# The Human Airway Epithelial Basal Cell Transcriptome

**DOI:** 10.1371/journal.pone.0018378

**Published:** 2011-05-04

**Authors:** Neil R. Hackett, Renat Shaykhiev, Matthew S. Walters, Rui Wang, Rachel K. Zwick, Barbara Ferris, Bradley Witover, Jacqueline Salit, Ronald G. Crystal

**Affiliations:** 1 Department of Genetic Medicine, Weill Cornell Medical College, New York, New York, United States of America; 2 Division of Pulmonary and Critical Care Medicine, Department of Medicine, Weill Cornell Medical College, New York, New York, United States of America; Comprehensive Pneumology Center, Germany

## Abstract

**Background:**

The human airway epithelium consists of 4 major cell types: ciliated, secretory, columnar and basal cells. During natural turnover and in response to injury, the airway basal cells function as stem/progenitor cells for the other airway cell types. The objective of this study is to better understand human airway epithelial basal cell biology by defining the gene expression signature of this cell population.

**Methodology/Principal Findings:**

Bronchial brushing was used to obtain airway epithelium from healthy nonsmokers. Microarrays were used to assess the transcriptome of basal cells purified from the airway epithelium in comparison to the transcriptome of the differentiated airway epithelium. This analysis identified the “human airway basal cell signature” as 1,161 unique genes with >5-fold higher expression level in basal cells compared to differentiated epithelium. The basal cell signature was suppressed when the basal cells differentiated into a ciliated airway epithelium *in vitro*. The basal cell signature displayed overlap with genes expressed in basal-like cells from other human tissues and with that of murine airway basal cells. Consistent with self-modulation as well as signaling to other airway cell types, the human airway basal cell signature was characterized by genes encoding extracellular matrix components, growth factors and growth factor receptors, including genes related to the EGF and VEGF pathways. Interestingly, while the basal cell signature overlaps that of basal-like cells of other organs, the human airway basal cell signature has features not previously associated with this cell type, including a unique pattern of genes encoding extracellular matrix components, G protein-coupled receptors, neuroactive ligands and receptors, and ion channels.

**Conclusion/Significance:**

The human airway epithelial basal cell signature identified in the present study provides novel insights into the molecular phenotype and biology of the stem/progenitor cells of the human airway epithelium.

## Introduction

The airway epithelium, a continuous pseudostratified population of cells lining the dichotomously branching airways, provides the barrier function that defends against inhaled gases, particulates, pathogens and other xenobiotics [1]–[3]. In humans, the airway epithelium is comprised of 4 major cell types, including ciliated, secretory, columnar and basal cells [1]–[3]. While the ciliated, secretory and columnar cells constitute the primary host defense barrier, it is the basal cells, a proliferating population of cuboidal-shaped cells, that provide the major stem/progenitor cell function from which other airway epithelial cells are derived [4]–[8]. As part of normal epithelial turnover and repair, the basal cells differentiate into the ciliated cells that help cleanse the surface of the airways, and secretory cells that produce mucins and other products that contribute to the extracellular apical barrier [1], [6], [8]. This process can be recapitulated by culture on air-liquid interface (ALI), where undifferentiated basal cells differentiate into ciliated and secretory cells [9]–[14]. The unique contribution of airway basal cells to the structural integrity of the airway epithelial barrier in the steady-state and during tissue injury suggests they have a distinct gene expression program enabling these cells to function in this manner.

In this context, it is the purpose of this study to characterize the human airway basal cell transcriptome. Taking advantage of the ability to culture pure populations of human airway basal cells from the complete airway epithelium obtained by brushing the airway epithelium of healthy nonsmokers, we characterized the “human airway basal cell signature” by comparing the transcriptome of the cultured airway basal cells to that of the complete differentiated airway epithelium from which the basal cells were isolated. Interestingly, while human basal cells express many of the genes and pathways expected from a basal cell population, the human basal cell signature includes several unique gene categories/pathways that likely play a significant role in human airway basal cell biology.

## Methods

### Sampling the Airway Epithelium

Healthy, nonsmoking subjects were recruited under a protocol approved by the Weill Cornell Medical College Institutional Review Board. For 12 individuals, the complete differentiated airway epithelium was evaluated. For 8 individuals, the epithelium was cultured under conditions to obtain pure populations of basal cells. All subjects were confirmed to be nonsmokers by urine levels of nicotine (<2 ng/ml) and cotinine (<5 ng/ml) with normal pulmonary functions tests and chest X-ray. The demographics of the individuals from whom the basal cells and the differentiated airway epithelium were assessed were similar (p>0.05) for gender and ancestry (by Chi-square test) and age (by t-test).

After obtaining written informed consent, flexible bronchoscopy was used to collect large airway epithelial cells by brushing the epithelium as previously described [15]–[17]. Cells were detached from the brush by flicking into 5 ml of ice-cold Bronchial Epithelium Basal Medium (BEGM, Lonza, Basel, Switzerland). An aliquot of 0.5 ml was used for differential cell count. The remainder (4.5 ml) was processed immediately for either immediate RNA extraction (n = 12) or basal cell culture followed by RNA extraction (n = 5) or culture on ALI (n = 3). The number of cells recovered by brushing was determined by counting on a hemocytometer. To quantify the percentage of epithelial and inflammatory cells and the proportions of basal, ciliated, secretory and columnar cells recovered, cells were prepared by centrifugation (Cytospin 11, Shandon Instruments, Pittsburgh, PA) and stained with Diff-Quik (Baxter Healthcare, Miami, FL). In all samples the epithelial cells represented >97% of the cell population; the proportions of epithelial cells were as previously reported [15], [17].

### Culture and Characterization of Basal Cells

Airway epithelial cells collected by brushing were pelleted by centrifugation (250× g, 5 min) and disaggregated by resuspension in 0.05% trypsin-ethylenediaminetetraacetic acid (EDTA) for 5 min at 37°C. Trypsinization was stopped by addition of HEPES buffered saline (Lonza, Basel, Switzerland) supplemented with 15% fetal bovine serum (FBS; GIBCO-Invitrogen, Carlsbad, CA), and the cells were again pelleted at 250× g, 5 min. The pellet was resuspended with 5 ml of phosphate buffered saline, pH 7.4 (PBS), at room temperature, then centrifuged at 250× g, 5 min. Following centrifugation, the PBS was removed, the cells resuspended in 5 ml of BEGM and 5×10^5^ cells were cultured in T25 flasks in BEGM, supplemented with growth factors according to the manufacturer's instructions. The antibiotics supplied by the manufacturer of BEGM were replaced with gentamycin (50 µg/ml; Sigma, St Louis, MO), amphotericin B (1.25 µg/ml; Invitrogen, Carlsbad, CA), and penicillin-streptomycin (50 µg/ml; Invitrogen, Carlsbad, CA). Cultures were maintained in a humidified atmosphere of 5% CO_2_ at 37°C. Unattached cells were removed by changing medium after 12 hr. Thereafter, media was changed every 2 days with characterization and analysis at 7 to 8 days, when the cells were 70% confluent.

To characterize the basal cell cultures by immunohistochemistry, the cells were trypsinized, and cytospin slide preparation fixed in 4% paraformaldehyde for 15 min. To enhance staining, an antigen recovery step was carried out by steaming the samples for 15 min in citrate buffer solution (Labvision, Fremont, CA) followed by cooling at 23°C, 20 min. Endogenous peroxidase activity was quenched using 0.3% H_2_O_2_, and normal serum matched secondary antibody was used for 20 min to reduce background staining. Samples were incubated overnight at 4°C with primary antibodies, including rabbit polyclonal anti-human cytokeratin 5 antibody (1/50; Thermo Scientific, Rockford, IL), mouse monoclonal anti-human p63 (1/50; Santa Cruz Biotechnology, Inc., Santa Cruz, CA), mouse monoclonal anti-human CD151 (1/200; Leica Microsystems, Inc., Bannockburn, IL) as markers for basal cells; mouse monoclonal anti-human N-cadherin antibody (1/2500; Invitrogen, Carlsbad, CA) for mesenchymal cells; mouse monoclonal anti-human mucin 5AC antibody (1/50; Vector Laboratories, Burlingame, CA) and mouse monoclonal anti-TFF3 (0.1 µg/ml; Santa Cruz) for secretory cells; and mouse monoclonal anti-human β-tubulin IV antibody (1/2000 dilution; Biogenex, San Ramon, CA) for ciliated cells and mouse monoclonal anti-human chromagranin A (1/5000; Thermo Scientific, Rockford, IL) and mouse anti-CGRP (0.2 µg/ml; Sigma, St Loius MO) for neuroendocrine cells. Isotype matched IgG (Jackson Immunoresearch Laboratories, Inc, West Grove, PA) was the negative control. Vectastain Elite ABC kit and AEC substrate kit (Dako North America, Inc, Carpinteria, CA) were used to visualize antibody binding. The sections were counterstained with Mayer's hematoxylin (Polysciences, Inc, Warrington, PA) and mounted using Faramount mounting medium (Dako North America, Inc.). Brightfield microscopy was done using a Nikon Microphot microscope equipped with a Plan ×40 numerical aperture (NA) 0.70 objective lens. Images were captured with an Olympus DP70 CCD camera.

To characterize the basal cell cultures by Western analsysis, the cells were trypsinized and lysed in radioimmunoprecipitation lysis (RIPA) buffer plus Complete Protease Inhibitor Cocktail (Roche, Mannheim, Germany), and incubated on ice for 30 min. Lysates were clarified by centrifugation at 22,500× *g* for 10 min in an Eppendorf 5415C microcentrifuge at 4°C. The total protein concentration was measured using the Bio-Rad (Hercules, CA) protein assay to the manufacturer's guidelines. For samples of large airway epithelium, the cells were obtained directly from brushing and following two washes with PBS, processed in an identical manner to the cultured basal cells. NuPAGE® LDS Sample Buffer (4×) (supplemented with 200 mM dithiothreitol) was added to each sample before boiling for 10 min and SDS-polyacrylamide gel electrophoresis (PAGE) analysis using NuPAGE® 4 to 12% Bis-Tris gradient gels (Invitrogen). Proteins were transferred onto nitrocellulose membranes with a Bio-Rad Semi-Dry apparatus before Western analysis. After blocking membranes overnight at 4°C in 4% nonfat milk in PBS containing 0.1% Tween-20 (PBST), immobilized proteins were reacted with cell type specific antibodies in 4% nonfat milk in PBST for 1 hr, 23°C with shaking, including: rabbit polyclonal anti-human cytokeratin 5 (1/3000; Thermo Scientific); mouse monoclonal anti-human cytokeratin 14 (1/3000; R&D Biosystems, Minneapolis, MN); and mouse monoclonal anti-human p63 (1/1000; Santa Cruz Biotechnology, Inc.) for basal cells; mouse monoclonal anti-human mucin 1 (1/500; Santa Cruz Biotechnology, Inc.); mouse monoclonal anti-human mucin 5AC (1/500; Vector Laboratories, Burlingame, CA); and mouse monoclonal anti-human trefoil factor 3 (TFF3/ITF; 1/500; Santa Cruz Biotechnology, Inc.) for secretory cells; rabbit polyclonal anti-human dynein intermediate chain 1 (DNAI1; 1/3000; Sigma, St Louis, MO) for ciliated cells and mouse monoclonal anti-human glyceraldehyde dehydrogenase (GAPDH; 1/5000; Santa Cruz Biotechnology, Inc.) as a loading control. Following the primary antibody incubation, membranes were washed three times for 5 min each with PBST, incubated with an anti-rabbit or anti-mouse antibody conjugated to horseradish peroxidase in 4% nonfat milk in PBST for 1 hr, 23°C with shaking. Upon completion of secondary antibody incubation, the membranes were washed again three times for 5 min with PBST and twice with PBS, and antibodies were visualized after the addition of ECL Western Blotting Detection Reagents (GE Healthcare Biosciences, Pittsburgh, PA) by exposure to X-ray film.

### Airway Epithelium Differentiation in Air-liquid Interface Culture

To demonstrate that the cultured population of basal cells could function as stem/progenitors for differentiated airway epithelial cells, the pure population of basal cells for n = 3 subjects were grown as ALI cultures [18]. The basal cells were trypsinized and seeded at a density of 6×10^5^ cells/cm^2^ onto a 0.4 µm pore-sized Costar Transwells inserts (Corning Incorporated, Corning, NY) pre-coated with type IV collagen (Sigma, St Louis, MO). The initial culture medium consisted of a 1∶1 mixture of DMEM and Ham's F-12 medium (GIBCO-Invitrogen, Carlsbad, CA) containing 100 U/ml penicillin, 5% fetal bovine serum 100 µg/ml streptomycin, 0.1% gentamycin, and 0.5% amphotericin B. On the next day, the medium was changed to 1∶1 DMEM/Ham's F12 (including antibiotics described above) with 2% Ultroser G serum substitute (BioSerpa S.A., Cergy-Saint-Christophe, France). Once the cells had reached confluence (typically following 2 days of culturing on the membrane) the media was removed from the upper chamber to expose the apical surface to air and establish the ALI (referred to as ALI “day 0”). The cells were then grown at 37°C, 8% CO_2_, and the culture medium was changed every other day. Following 5 days on ALI, the cells were grown at 37°C, 5% CO_2_ until harvested.

To assess cell differentiation, the ALI membranes were processed for immunofluorescence with an anti-cytokeratin 5 and anti-β-tubulin IV antibody and scanning electron microscopy. For immunofluorescence the samples were processed by two methods. For whole membrane analysis, the membrane was fixed in 4% paraformaldehyde for 15 min inside the ALI transwell. Following fixation, the cells were permeabilized with 0.1% triton X-100 in PBS and then blocked with normal serum matched to the secondary antibody for 20 min to reduce background staining. The samples were stained for the presence of ciliated cells using the primary antibody mouse monoclonal anti-human β-tubulin IV (1/2000; red channel, Biogenex, San Ramon, CA) incubated at 23°C, 30 min. Isotype matched IgG (Jackson Immunoresearch Laboratories, West Grove, PA) was the negative control. Cy3-conjugated AffiniPure Donkey anti-mouse IgG (1/200; Jackson Immunoresearch Laboratories, West Grove, PA) was used as a secondary antibody to visualize antibody binding. The sections were counterstained with DAPI to identify cell nuclei (blue channel). Upon completion of staining, the membrane was cut from the well and mounted using SlowFade Antifade (Invitrogen, Carlsbad, CA). Immunofluorescent microscopy was performed using an Olympus IX70 body microscope equipped with a 60× oil immersion lens. Images were captured with a Photometrics, Quantix model camera. For analysis of paraffin embedded sections, samples were first cleaned in xylene and rehydrated with graded ethanol. To unmask the antigens, samples were steamed for 15 min in citrate buffer solution (Labvision, Fremont, CA) followed by cooling at 23°C, 20 min. The sections were then blocked with normal serum matched secondary to the secondary antibody for 30 min to reduce background staining. The samples were stained for the presence of basal cells using the primary antibody rabbit polyclonal anti-human cytokeratin 5 (1/50; green channel, Thermo Scientific, Rockford, IL) and ciliated cells using the primary antibody mouse monoclonal anti-human β-tubulin IV (1/2000; red channel, Biogenex, San Ramon, CA) incubated at 4°C overnight. Isotype matched IgG (Jackson Immunoresearch Laboratories, West Grove, PA) was the negative control. Cy3 conjugated AffiniPure Donkey anti-rabbit IgG (1/200; Jackson Immunoresearch Laboratories) and FITC-conjugated AffiniPure Donkey anti-mouse IgG (1/200; Jackson Immunoresearch Laboratories) were used as the secondary antibodies to visualize antibody binding. The sections were counterstained with DAPI to identify cell nuclei (blue channel). Upon completion of staining, the slides were coverslipped with SlowFade GOLD (Invitrogen, Carlsbad, CA). Immunofluorescent microscopy was performed using a Zeiss Axioplan body microscope with a 100× oil immersion lens. The images were captured with a Zeiss hrM (High resolution monochrome) camera and false colored. For analysis by electron microscopy, the membranes were removed from the well and fixed in a modified Karnovsky's fix [19], post-fixed with osmium tetroxide, dehydrated through graded ethanols, critical point dried through CO_2_, and sputtered with Au-Pd. Samples were subsequently examined and images collected in an FEI Quanta 600 SEM.

### RNA and Microarray Processing

Gene expression was assessed using the HG-U133 Plus 2.0 array (Affymetrix, Santa Clara, CA), which includes probes for more than 47,000 genome-wide transcripts as previously described [20], [21]. Total RNA was extracted using a modified version of the TRIzol method (Invitrogen, Carlsbad, CA), in which RNA is purified directly from the aqueous phase (RNeasy MinElute RNA purification kit, Qiagen, Valencia, CA). RNA samples were stored in RNA Secure (Ambion, Austin, TX) at −80°C. RNA integrity was determined by assessing an aliquot of each RNA sample on an Agilent Bioanalyzer (Agilent Technologies, Palo Alto, CA). The concentration was determined using a NanoDrop ND-1000 spectrophotometer (NanoDrop Technologies, Wilmington, DE). Double-stranded cDNA was synthesized from 1 to 2 µg total RNA using the GeneChip One-Cycle cDNA Synthesis Kit, followed by cleanup with GeneChip Sample Cleanup Module, *in vitro* transcription (IVT) reaction using the GeneChip IVT Labeling Kit, and cleanup and quantification of the biotin-labeled cDNA yield by spectrophotometry. All kits were from Affymetrix (Santa Clara, CA). All HG-U133 Plus 2.0 microarrays were processed according to Affymetrix protocols, hardware and software, processed by the Affymetrix fluidics station 450 and hybridization oven 640 and scanned with an Affymetrix Gene Array Scanner 3000 7G. Overall microarray quality was verified by the following criteria: (1) RNA Integrity Number (RIN) >7.0; (2) 3′/5′ ratio for GAPDH <3; and (3) scaling factor <10.0 [22]. All MIAME-compliant raw data have been deposited in the Gene Expression Omnibus (GEO) site (http://www.ncbi.nlm.nih.gov/geo), curated by the National Center for Bioinformatics. Accession number for the data is GSE24337.

### Analysis and Statistics

For microarrays passing QC, the expression levels for all probe sets were extracted using GeneSpring 11 after normalization by array only rejecting those probes that were not expressed in any sample (No Affymetrix “P” call). Significant gene expression differences between the basal cells and differentiated epithelium were determined with Benjamini-Hochberg correction for multiple testing [23]. Unsupervised hierarchical cluster analysis of the normalized expression levels of the differentiated epithelium and basal cell cultures was done using GeneSpring GX 7.3. Two independent sets of 1,000 random genes were selected using the Excel RANDBETWEEN function on all HG-U133 Plus 2.0 probe sets. The clustering was done with Spearman correlation as a similarity measure and average linkage as a clustering algorithm for both genes and samples. Genes expressed above the average are represented in red, below average in blue, and average in white. To compare the present data with data from other cell types, Gene Expression Omnibus datasets from HG-U133 Plus 2.0 microarray were used as a source of cel files, which were imported into Partek Genomic Suite version 6.5.2 (Partek, St. Louis, MO), by Robust Multiarray Analysis normalization simultaneously with the cel files from the current study. Principal component analysis (PCA) used normalized expression data in the Partek Genomic Suite using all probe sets or probe sets filtered for the basal cell signature genes. Genes were assigned to functional categories with online utilities, including GATHER (http://gather.genome.duke.edu/) [24], GoSurfer (http://bioinformatics.bioen.illinois.edu/gosurfer/) [25] and Ingenuity Pathway Analysis (Ingenuity Systems, Redwood City, CA).

## Results

### Culture and Characterization of Airway Epithelial Basal Cells

**Figure 1:**
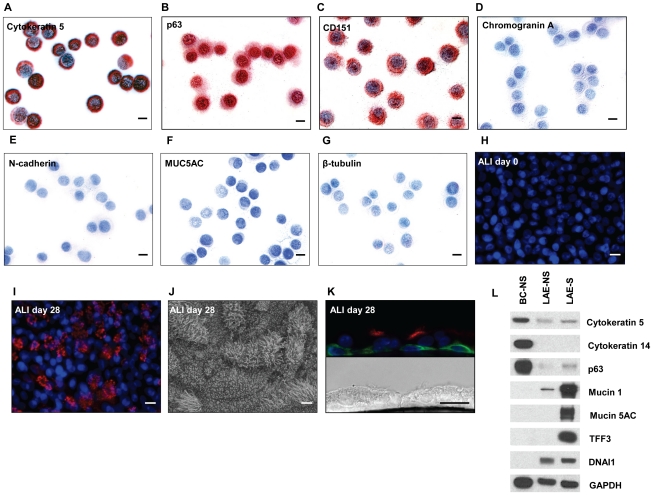
Characterization of cultured human airway epithelial basal cells. Large airway epithelial cells were collected by bronchoscopy brushing of healthy nonsmokers and cultured under basal cell-selective conditions for 7 to 8 days until 70% confluent. **A–G.** Confirmation of basal cell identity and purity by immunohistochemistry of cytospin preparations using cell type-specific markers. **A.** cytokeratin 5 (basal cells); **B.** TP63 (basal cells); **C.** CD151 (basal cells); **D.** chromagranin A (neuroendocrine cells) **E.** N-cadherin (mesenchymal cells); **F.** mucin (MUC) 5AC (secretory cells) and **G.** β-tubulin IV (ciliated cells). All cells were counterstained with Mayer's hematoxylin. **H–K.** Differentiation of basal cells on air-liquid interface cultures. **H.** Immunofluorscent staining for β-tubulin IV, day 0 (DAPI, nucleus). **I.** β-tubulin IV, day 28 (DAPI, nucleus; red, β-tubulin). **J.** Scanning electron microscopy at day 28. Scale bar for all panels A–J = 10 µm. **K.** Immunofluorescent staining of section of 28 day air liquid interface culture for β-tubulin (red) and cytokeratin 5 (green). **L.** Western analysis of basal and differentiated cell proteins probing equal amounts of extracts of basal cells from nonsmoker (BC-NS), large airway epithelium from a nonsmoker (LAE-NS), and large airway epithelium from a smoker (LAE-S) with antibodies as described in Methods section.

Human airway epithelial basal cells purified from airway brushings of healthy nonsmokers were assessed by immunohistochemistry. The basal cell markers cytokeratin 5, tumor protein 63, and CD151 were expressed in >95% of cells (Figure 1A–C) but the cells were negative for the neuroendocrine cell marker chromogranin A, the mesenchymal cell marker N-cadherin, the secretory cell marker mucin 5AC and the ciliated cell marker β-tubulin IV (Figure 1 D–G). Cell counting confirmed that differentiated cells were undetectable in cytospins of basal cells stained with markers for various cell types including two markers for ciliated cells (β-tubulin IV, 0/500 positively stained cells; dynein intermediate chain 1, 0/500), secretory cell marker (trefoil factor 3, 0/1000), mesenchymal cell marker (N-cadherin, 0/500) and two neuroendocrine cell markers [chromogranin A, 0/1000; calcitonin-related polypeptide alpha (CGRP), 0/1000]. The potential of the pure population of human airway basal cells to differentiate was confirmed by culturing the basal cells on ALI. Over 28 days, there was a progressive increase in the number of ciliated cells as indicated by β-tubulin IV immunofluorescence staining (Figure 1H–I) and by scanning electron microscopy (Figure 1J). In the air liquid interface culture, cells with basal-like morphology abutting the substratum and staining positive for cytokeratin 5 remained after 28 days simultaneous with the presence of ciliated cells staining positive for β-tubulin IV (Figure 1K). Identity and absence of differentiated cells was also confirmed by Western analysis using antibodies against three basal cell specific proteins, cytokeratin 14, cytokeratin 5 and p63, which were expressed at higher levels in basal cells than large airway epithelium (Figure 1L). Western analysis also showed the absence of expression of secretory cell proteins mucin 1, mucin 5AC and trefoil factor 3 which were expressed in the large airway epithelium especially of smokers. Similarly, Western analysis showed the absence of the cilia cell specific protein dynein intermediate chain 1 in the basal cells while it was expressed in large airway epithelium. We also used Western analysis for the neuroendocrine protein chromagranin, demonstrating no expression in the basal cells at the protein level (not shown).

### Human Airway Basal Cell-enriched Genes

**Figure 2:**
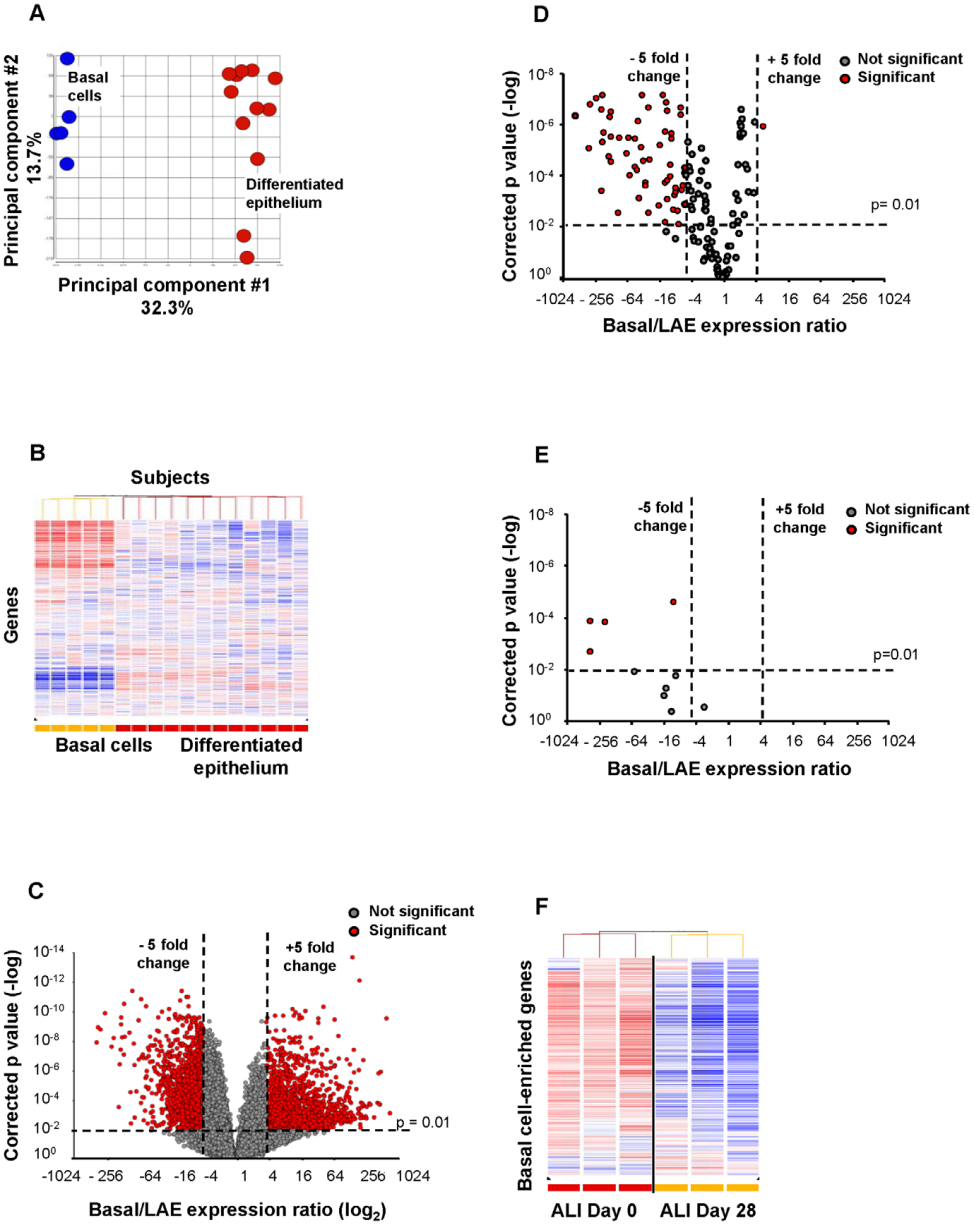
Identification of basal cell-enriched transcripts. **A.** Principal component analysis of gene expression of basal cells (n = 5; blue circles) and differentiated airway epithelium samples (n = 12; red circles) using all expressed gene probe sets (n = 39,324) as an input dataset. **B.** Hierarchical cluster analysis of basal cells (n = 5) compared to complete airway epithelium samples (n = 12) based on the expression of 1,000 randomly chosen probe sets detected in either of groups. Genes expressed above the average are represented in red, below average in blue, and average in white. The genes are represented vertically, and individual samples horizontally. **C.** Volcano plot comparing the transcriptomes of basal cells (n = 5) and complete airway epithelium (n = 12). In both panels, the y-axis corresponds to the negative log of p value and the x-axis corresponds to the log2-transformed fold-change. Red dots represent significant differentially expressed probe sets (fold-change >5; p value<0.01 with Benjamini-Hochberg correction); grey dots represent nonsignificant gene probe sets. **D.** Volcano plot assessing the transcriptome of basal cells *vs* complete large airway epithelium using a list of only ciliogenesis-related genes [29], [30]. **E.** Volcano plot assessing the transcriptomes of basal cell *vs* complete large airway epithelium using a list of only secretory cell-related genes [29]. **F.** Suppression of the basal cell-enriched transcriptome when basal cells are induced to differentiate into specialized airway cells in air-liquid interface culture. Pure populations of basal cells were plated onto air-liquid interface cultures and RNA was prepared on day 0 and day 28. The gene expression profile was determined and a cluster built using the genes of human airway basal cell-enriched transcriptome.

Gene expression microarrays were used to compare the transcriptomes of the human airway basal cells and the differentiated airway epithelium. When assessed by principal component analysis (PCA), the basal cell samples were clearly separated from the differentiated epithelium (Figure 2A). Clustering with 1,000 random genes also gave complete separation of basal and differentiated epithelium samples with a clear group of genes overexpressed in basal cells relative to differentiated epithelium, and another group of genes underexpressed in basal cells relative to differentiated epithelium (Figure 2B). Clustering with another independent set of 1,000 randomly picked genes gave a very similar pattern (not shown). A volcano plot revealed a large number of probe sets significantly (p<0.01) overexpressed (basal/differentiated epithelium ratio >5) or underexpressed (basal/differentiated epithelium ratio <0.2) in the basal cells compared to the differentiated epithelium (Figure 2C). This cut off is based on the knowledge that the differentiated human airway epithelium contains ∼20% basal cells [17], [26]–[28], i.e, we expect a basal cell-enriched gene to have a basal cell/differentiated epithelium expression ratio of >5. The subset of genes up-regulated in basal cells, as compared to the complete differentiated airway epithelium (ratio >5, p<0.01), included 1,828 probe sets representing 1,161 unique genes. These genes (see Table 1 for top 45; Table S1 for the complete list) will be further referred to as the “basal cell signature.”

**Table 1:** Top 45 Human Airway Basal Cell-enriched Genes.^1^

Category	ProbeSetID	Gene symbol	Gene title	Mean expression in differentiated epithelium	Mean expression in basal cells	Basal/differentiated epithelium expression ratio	p value^2^
Cytoskeleton	209125_at	KRT6A	keratin 6A	1.1	724.4	667.2	3.2×10^−9^
	209800_at	KRT16	keratin 16	0.1	88.7	635.4	2.7×10^−8^
	209126_x_at	KRT6B	keratin 6B	1.8	353.0	196.2	3.6×10^10^
	209191_at	TUBB6	tubulin, beta 6	1.1	142.5	127.8	4.0×10^−4^
	221870_at	EHD2	EH-domain containing 2	0.3	33.1	123.7	1.1×10^−8^
	212077_at	CALD1	caldesmon 1	1.2	135.8	109.6	1.2×10^−5^
	201564_s_at	FSCN1	fascin homolog 1, actin-bundling protein	0.3	26.9	89.0	1.3×10^−5^
Extracellular matrix	204636_at	COL17A1	collagen, type XVII, alpha 1	0.1	31.0	353.0	6.5×10^−6^
	201656_at	ITGA6	integrin, alpha 6	2.6	129.8	50.8	5.9×10^−9^
Protease/antiprotease	222162_s_at	ADAMTS1	ADAM metallopeptidase with thrombospondin type 1 motif, 1	0.1	43.1	327.7	4.8×10^−5^
	205778_at	KLK7	kallikrein-related peptidase 7	0.1	24.2	221.3	3.6×10^−5^
	205479_s_at	PLAU	plasminogen activator, urokinase	0.9	113.5	132.0	3.8×10^−3^
	202628_s_at	SERPINE1	serpin peptidase inhibitor, clade E, member 1	0.4	46.8	116.5	7.7×10^−3^
	212190_at	SERPINE2	serpin peptidase inhibitor, clade E, member 2	0.9	85.0	91.4	1.9×10^−9^
Epidermal function	206884_s_at	SCEL	sciellin	0.4	73.3	194.6	2.0×10^−7^
	213796_at	SPRR1A	small proline-rich protein 1A	0.2	83.9	371.4	2.2×10^−5^
	205064_at	SPRR1B	small proline-rich protein 1B (cornifin)	0.7	234.3	345.5	5.4×10^−8^
	205239_at	AREG	amphiregulin	2.7	354.7	133.9	8.5×10^−10^
	232082_x_at	SPRR3	small proline-rich protein 3	0.8	48.2	60.1	5.0×10^−4^
Signaling ligand	205767_at	EREG	epiregulin	0.1	34.6	246.0	8.0×10^−7^
	206343_s_at	NRG1	neuregulin 1	0.4	19.8	53.4	7.4×10^−7^
Signal transduction	218309_at	CAMK2N1	calcium/calmodulin-dependent protein kinase II inhibitor 1	0.6	51.0	85.4	3.0×10^−7^
	204602_at	DKK1	dickkopf homolog	1.1	91.4	82.6	2.2×10^−3^
	210138_at	RGS20	regulator of G-protein signaling 20	0.2	16.1	82.3	3.5×10^−7^
	203438_at	STC2	stanniocalcin 2	0.1	21.1	277.1	2.6×10^−7^
	212097_at	CAV1	caveolin 1, caveolae protein, 22 kDa	1.0	177.4	169.5	1.0×10^−3^
	201109_s_at	THBS1	thrombospondin 1	0.5	59.4	110.4	7.3×10^−3^
Transcription	1555788_a_at	TRIB3	tribbles homolog 3 (Drosophila)	0.2	28.9	161.5	1.0×10^−8^
	1552487_a_at	BNC1	basonuclin 1	0.5	35.1	69.7	9.4×10^−5^
Metabolism	203234_at	UPP1	uridine phosphorylase 1	0.9	254.7	268.9	8.5×10^−9^
	219181_at	LIPG	lipase, endothelial	0.3	16.0	62.8	3.0×10^−4^
	231202_at	ALDH1L2	Aldehyde dehydrogenase 1 family, member L2	0.3	24.3	79.9	2.1×10^−5^
	223062_s_at	PSAT1	phosphoserine aminotransferase 1	4.3	233.0	54.5	1.0×10^−8^
Oxidation/reduction	218717_s_at	LEPREL1	leprecan-like 1	0.1	6.8	57.0	1.8×10^−3^
Gap junction	223278_at	GJB2	gap junction protein, beta 2, 26 kDa	3.3	183.1	56.2	4.0×10^−3^
	231771_at	GJB6	gap junction protein, beta 6, 30 kDa	0.2	18.7	75.3	8.9×10^−3^
Cell adhesion	235075_at	DSG3	desmoglein 3 (pemphigus vulgaris antigen)	0.5	114.9	222.2	5.5×10^−5^
Apoptosis	217996_at	PHLDA1	pleckstrin homology-like domain, family A, member 1	1.8	112.3	61.2	1.8×10^−5^
Immune responses	207526_s_at	IL1RL1	interleukin 1 receptor-like 1	0.2	106.3	449.9	7.0×10^−4^
	206172_at	IL13RA2	interleukin 13 receptor, alpha 2	0.2	32.9	200.8	9.0×10^−4^
	212657_s_at	IL1RN	interleukin 1 receptor antagonist	2.1	162.7	77.6	3.0×10^−4^
Ion transport	209900_s_at	SLC16A1	solute carrier family 16, member 1	0.3	23.8	69.8	1.9×10^−7^
	201195_s_at	SLC7A5	solute carrier family 7, member 5	0.6	201.0	314.7	6.7×10^−8^
Unknown	220620_at	CRCT1	cysteine-rich C-terminal 1	0.2	16.9	97.4	7.0×10^−4^

1 The probe set with the highest basal/differentiated epithelium expression ratio for the top 45 enriched named genes.

2 BH – Benjamini-Hochberg.

By definition, the basal cell signature should exclude genes expressed selectively or more abundantly in ciliated and secretory cell types. To ensure this was the case, the basal cell and differentiated epithelium expression levels were assessed for a cilia-specific gene list derived from proteomic studies [29], [30]. The analysis revealed that 41% (58 of 141) of the expressed ciliated cell-specific probe sets were significantly down-regulated (basal/differentiated epithelium ratio <0.2) in the basal cells (Figure 2D) and that only 1 of 141 probe sets corresponding to ciliated genes met the criteria for inclusion in basal cell signature. Similarly, 40% of the probe sets corresponding to a secretory cell gene list [29] showed significant underexpression in the basal cells relative to differentiated epithelium with a basal/differentiated epithelium expression ratio of <0.2 (Figure 2E). The dataset was also assessed for expression of 11 neuroendocrine genes [31]. Of the 21 probesets representing these genes, only three (14%) were expressed in basal cell samples based on Affymetrix call of “Present”.

To confirm that the cultured basal cells maintained their “*in vivo*” capacity to function as stem/progenitor cells capable of generating differentiated airway epithelial cell types, the basal cells were plated on ALI culture. Transcriptome-wide microarray analysis of these cultures at day 0 and day 28 (before and after differentiation) showed that the expression of the human airway basal cell signature genes was markedly suppressed as the basal cells differentiated (Figure 2F). The median expression ratio for all of the genes of the basal cell signature between day 0 and day 28 of culture in air liquid interface was 0.49 indicating reduction in the expression of basal cell signature upon differentiation into specialized epithelial cell types.

Among the top 45 basal cell signature genes were genes coding for the cytoskeleton, extracellular matrix, proteases/antiproteases, epidermal function, signaling ligands, signal transduction, transcription, metabolism, oxidation reduction, gap junctions, cell adhesion, immune responses, ion transport and apoptosis (Table 1). Although classical basal cell genes such as cytokeratin 5, transcription factors p63 and basonuclin, and hemidesmosome component integrin ITGA6 were included in the human airway basal cell signature, the top 5 genes most highly expressed in the basal cells were 2 cytokeratins (KRT6A, KRT16), interleukin 1 receptor-like 1, small proline-rich protein 1A and collagen type XVII alpha1. Other classic basal cell genes [32] were overexpressed in basal cells relative to differentiated epithelium but fell short of the p value cutoff or 5 fold expression ratio cut off to be included in the basal cell signature. These included CD151 (expression ratio = 3.6, p<0.01, tissue factor (3.2, p<0.01), cytokertain 13 (10.6, p = 0.04) and cytokertain 14 (ratio = 200, p = 0.02).

### Comparative Analyses

**Figure 3:**
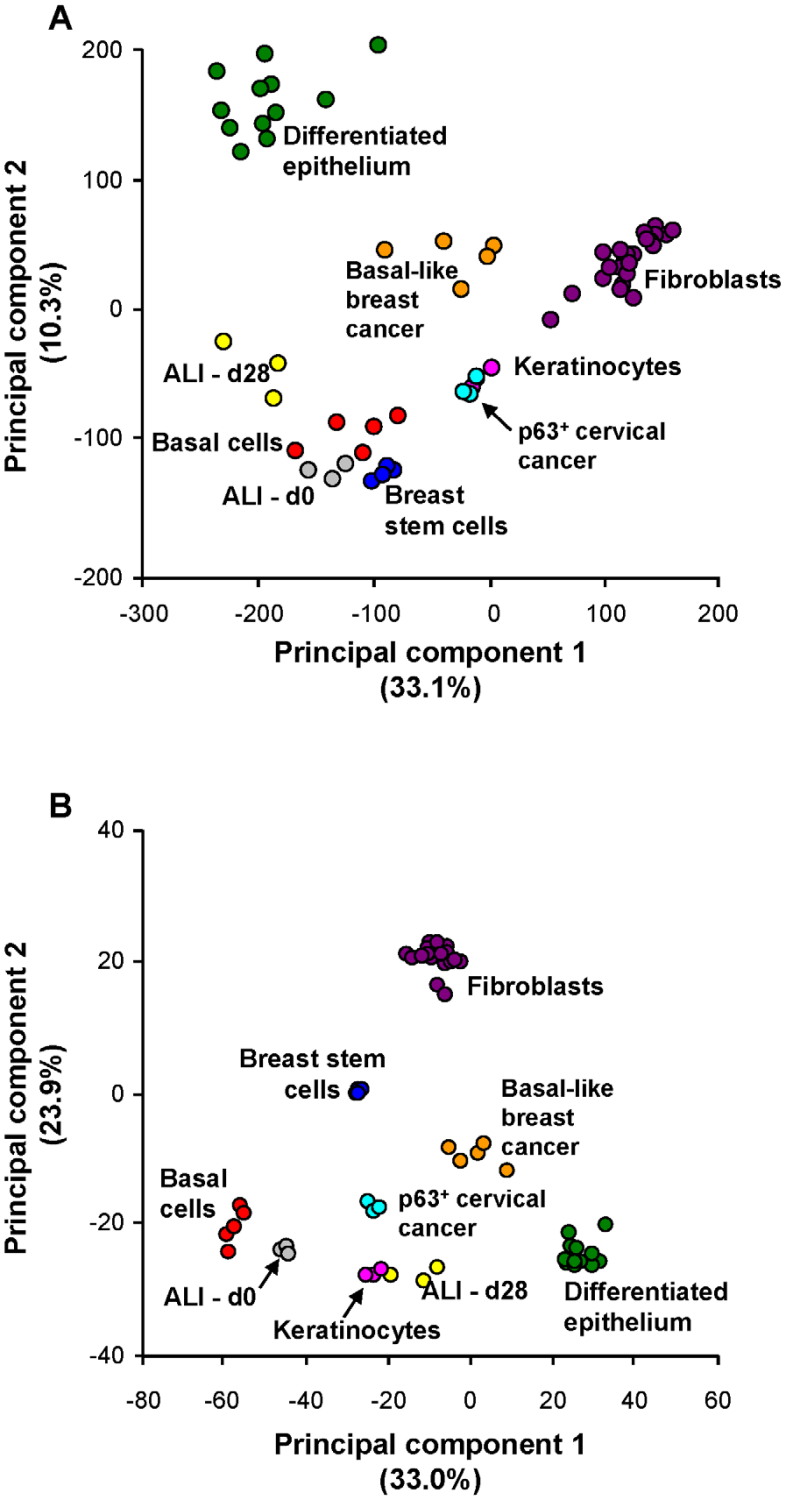
Principal component analysis-based comparison of airway basal cells to other human tissues and cells. Compared tissues and cell types were all of human origin and included: basal cells (human airway basal cells, red; n = 5); differentiated epithelium (complete large airway epithelium obtained by brushing, green; n = 12); ALI-d0 (basal cells cultured on ALI until confluent, ∼2 days after plating (see Methods), grey; n = 3); ALI-d28 (the same airway basal cells after 28 days of differentiation in air-liquid interface, yellow; n = 3); breast stem cells (from Gene Expression Omnibus GSE15192: CD44+ CD24− stem-like fraction of MCF-10A immortalized breast epithelial cells, dark blue; n = 4), basal-like breast cancer (GSE3744: orange; n = 5); keratinocytes (GSE7216: primary neonatal foreskin epidermal keratinocytes, pink; n = 3); cervical cancer (GSE5993: p63-overexpressing cervical cancer cell line ME180; light blue; n = 3); and fibroblasts (GSE17032: human skin and lung fibroblasts, purple; n = 20). **A.** Analysis based on the entire transcriptome. **B.** Analysis based on the 1,161 genes of basal cell signature.

Principal component analysis was used to visualize the differences between airway basal cells and other human cell types and tissues including those having basal cell-like characteristics (Figure 3). The complete transcriptome and the airway basal cell signature were compared for the basal cells, the differentiated airway epithelium and the human airway basal cells placed in ALI cultures on days 0 and 28. In addition, publically available external datasets imported from Gene Expression Omnibus were compared, including the datasets of keratinocytes [33], cervical cancer cell line ME180 overexpressing basal cell-associated transcription factor p63 [34], a CD44+CD24- stem/progenitor-like immortalized breast epithelial cell [35], basal-like breast carcinoma [36] and skin and lung fibroblasts [37]. Based on the analysis of the whole transcriptomes (Figure 3A) as well as in the analysis restricted to airway basal cell signature genes (Figure 3B), there was a clear vector in the PCA space from basal cells to differentiated epithelium. A parallel vector linked the day 0 ALI cultures to the day 28 ALI cultures. In both genome-wide and airway basal cell signature-restricted analyses, airway epithelium basal cells exhibited similarity to cells with basal cell characteristics, such as CD44+ breast epithelial stem cells, and p63-overexpressing cervical cells and keratinocytes, but had more distant relationships with basal-like breast cancers and fibroblasts (Figures 3A, 3B). The gene expression profile for keratinocytes and p63+ cervical basal cells were more similar to each other and more distantly related to the airway basal cells than were the breast basal cells. Although based on the whole transcriptome analysis, CD44+CD24- basal-like breast epithelial stem cells demonstrated the highest degree of phenotypic similarity to airway basal cells compared to all other cells/tissues analyzed (Figure 3A), PCA based on the airway basal cell signature genes segregated airway basal cells from all cells/tissues (Figure 3B). This observation suggests that the airway basal cell signature harbors transcriptome features that are unique to airway basal cells not only in comparison to other airway epithelial cell types, but also compared to basal-like stem/progenitor cells of other organs.

Interestingly, comparison of the human airway basal cell signature with the recently characterized transcriptome of mouse airway basal cells [7] revealed that, despite differences in the methodologies utilized for isolation and characterization of airway basal cells in humans in our study and in mice, there was a considerable overlap between the mouse and human basal cell signatures (Table S2). Overall, even though there were some differences between the airway basal cell transcriptomes of humans and mice, there were many cross-species similarities. The dataset of 105 overlapping genes included well-established airway basal cell-associated genes, such as those encoding cytokeratin 5, basonuclin, and p63. Although differing in some details, a number of enriched gene families were common to human and mouse airway basal cell signatures, including cytokeratins, integrins, and genes encoding various G protein-coupled receptors. Keratins 6A, 6B and 16, which had the highest degree of enrichment in the human airway basal cells (Table S1), were not enriched in murine basal cells. In contrast, the mouse basal cell transcriptome included keratins 5, 14, 17 and 31, of which only keratin 5 and 17 were present in the human airway basal cell signature. Further, the mouse basal cell transcriptome included the signaling ligands Wnt3A, Wnt5B and Wnt9A, whereas the human basal cell signature contained only WNT7A. Although the major basal cell-specific integrin ITGA6, encoding hemidesmosomes and relevant to stem/progenitor cell function, was present in both human and mouse airway basal cell signatures (Table S2), the genes encoding integrins ITGA5 and ITGB6 were enriched in human, but not mouse basal cells.

The specific genes expressed in basal cells from various human tissues were also compared to the genes of the airway epithelium basal cells of mice and humans. No consistent patterns were detected in which gene expression level in human basal cells from all tissues always differed from that in mouse airway epithelium basal cells. For example, the WNT7A gene that is preferentiality expressed in human but not mouse airway basal cells, was not highly expressed in basal cells of any other human tissue. Also with respect to cytokeratins, there was no human-specific expression pattern for basal cells from all tissues. For example, cytokeratin 13 which is highly up regulated in human airway basal cells was expressed only in cervical basal like cells and not in breast basal cells nor keratinocytes. By contrast cytokeratin 16, another highly expressed human airway basal cell gene, was expressed only in kertainocytes and to a much lesser extent in breast or cervical basal cells.

### Global Functional Characterization of the Human Airway Basal Cell Signature

**Figure 4:**
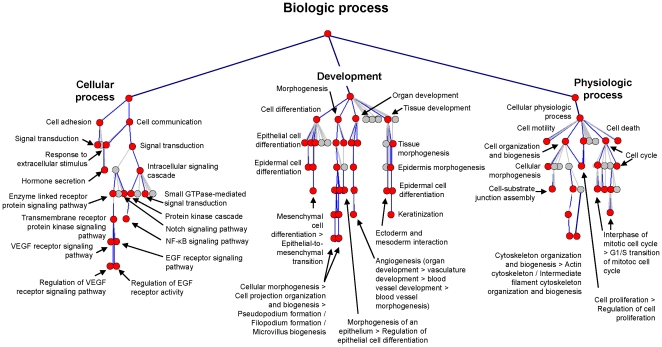
Hierarchical mapping of Gene Ontology (GO) categories enriched in the human basal cell transcriptome. Basal cell-enriched gene probe sets (n = 1,828) were used to generate a GO tree using GoSurfer software to display “biologic process” categories related to “cellular process” (left branch), “development” (middle branch) and “physiologic process” (right branch). Significantly enriched categories (p<10^−10^) are represented as red nodes; grey nodes represent mostly closely mapped nonsignificant categories; edges represent “parent-child” relationships of GO terms.

The GoSurfer tool was used to provide a global view of the functional characteristics of the 1,161-gene human airway basal cell signature by identifying enriched functional categories with their subsequent mapping to the hierarchical Gene Ontology tree (Figure 4). Consistent with the anatomic location of basal cells close to the extracellular matrix-rich tissue compartment and their established role in providing attachment of the airway epithelium to the basement membrane and physical interaction between various cell types [4], [8], GoSurfer analysis revealed enrichment of cellular processes related to cell adhesion, cell-cell interaction and tissue morphogenesis. Hierarchical analysis of enriched categories from the parent GO terms (levels 1–2) down to categories describing more specific biologic processes (levels 3–9) revealed a number of functions and pathways relevant to the biology of airway basal cells.

Among the categories enriched in the airway basal cell signature, a considerable number represented well-known signal transduction molecular pathways implicated in the regulation of tissue homeostasis and stem/progenitor cell function, including the NF-κB, Notch, EGFR, G protein-coupled receptor and VEGFR signaling pathways (Figure 4, left branch). This was accompanied by overexpression of genes belonging to “hormone secretion,” a category downstream of “response to extracellular stimulus,” suggesting the presence of putative self-regulating ligand-receptor interactions that operate in human airway basal cells in a cell-autonomous manner. Also consistent with stem/progenitor cell function, the airway basal cell signature was enriched in categories related to tissue development and differentiation (Figure 4, middle branch). The analysis revealed directionality of enriched differentiation-related categories, with a bias toward the ectoderm development pathway, providing an explanation for the similarity of the basal cell signature to keratinocytes, as shown in the PCA (Figure 3). Other enriched differentiation pathways included angiogenesis and mesenchymal cell differentiation, suggesting that airway basal cells might affect morphogenesis and differentiation of neighboring cell populations. Finally, the multifunctional role of airway basal cells in maintaining airway epithelial integrity was supported by the enrichment of functional categories related to cell motility, cell organization and biogenesis, cell-substrate junction assembly, cell cycle and proliferation (Figure 4, right branch).

### Gene Expression Patterns and Pathways Enriched in the Human Airway Basal Cell Signature

Specific gene expression patterns and molecular pathways enriched in the human airway basal cell signature were analyzed by means of the Gene Annotation Tool to Help Explain Relationships (GATHER) tool. Five Kyoto Encyclopedia of Genes and Genomes (KEGG) pathways were identified that were significantly overrepresented in the human airway basal cell signature (Table 2) and the genes of the basal cell signature corresponding to these KEGG pathways were identified (Table S3). In addition, GATHER analysis was used to identify a total of 29 Gene Ontology categories that were significantly overrepresented in the human airway basal cell signature (Table S4) and the top 10 (Table 3) were examined for the genes overlapping with the basal cell signature (Table S5). Finally, Ingenuity Pathway Analysis was used to identify canonical pathways overrepresented in the basal cell signature (Table 4) and the component genes identified (Table S6). These analyses were combined to identify key functions potentially related to basal cell functions.

**Table 2:** GATHER KEGG Pathway Analysis of the Human Airway Basal Cell-enriched Transcriptome.^1^

KEGG pathways	Annotation	Gene number/total^2^	% genes enriched	p value	Bayes factor^3^
hsa04510	Focal adhesion	44/201	22	2×10^−8^	13
hsa04512	Extracellular matrix-receptor interaction	18/84	21	0.0002	4
hsa04520	Adherens junction	16/75	21	0.0006	3
hsa04010	MAPK signaling pathway	32/271	12	0.004	1
hsa04810	Regulation of actin cytoskeleton	29/216	13	0.005	1

1 GATHER (Gene Annotation Tool to Help Explain Relationships, http://gather.genome.duke.edu/) was used to assist in functional annotation of the human basal cell-specific transcriptome. Input data consisted of the unique named genes from probe sets with basal/large airway epithelium expression ratio >5, p<0.01 following Benjamini-Hochberg multiple test correction. For the KEGG pathways analysis, the significant pathways are shown.

2 Number of genes in that category represented in the human airway basal cell-enriched transcriptome/total genes in pathway.

3 A measure of false discovery rate; a Bayes factor ≥1 is significant.

**Table 3:** GATHER GO Analysis of the Human Airway Basal Cell-enriched Transcriptome.^1^

Gene ontology	Annotation	Gene number/total^2^	% genes enriched	p value	Bayes factor^3^
GO:0007398	ectoderm development	22/218	10.1	6.5×10^−11^	17.1
GO:0008544	epidermis development	19/202	9.4	1.7×10^−9^	13.8
GO:0000074	regulation of cell cycle	52/448	11.6	4.1×10^−8^	11.0
GO:0009887	organogenesis	90/650	13.8	9.4×10^−7^	8.1
GO:0009653	morphogenesis	106/1338	7.9	1.3×10^−6^	7.9
GO:0008283	cell proliferation	98/1169	8.4	2.8×10^−6^	7.1
GO:0009113	purine base biosynthesis	4/8	50.0	1.2×10^−5^	4.8
GO:0030216	keratinocyte differentiation	5/75	6.7	3.3×10^−5^	3.8
GO:0006144	purine base metabolism	4/15	26.7	5.6×10^−5^	3.3
GO:0007049	cell cycle	66/1000	6.6	1.4×10^−4^	3.2

1 GATHER (Gene Annotation Tool to Help Explain Relationships, http://gather.genome.duke.edu/) was used to assist in functional annotation of the human basal cell-specific transcriptome. Input data consisted of the unique named genes from probe sets with basal/large airway epithelium expression ratio >5, p<0.01 following Benjamini-Hochberg multiple test correction. For the Gene Ontology analysis the top 10 genes are shown.

2 Number of genes in that category represented in the human airway basal cell-enriched transcriptome.

3 A measure of false discovery rate in which a positive number is significant; the GATHER analysis identified (n = 71) categories with Bayes factor ≥1; shown are the top 10 categories.

**Table 4:** Canonical Pathways Dominant in Human Airway Epithelium Basal Cells Compared to the Differentiated Complete Airway Epithelium.^1^

Pathway	Ratio (% of the total genes in pathway)^2^	p value
Neuregulin signaling	22/103 (21.4)	1.9×10^−7^
Integrin signaling	33/202 (16.3)	2.0×10^−6^
ILK signaling	31/186 (16.7)	2.5×10^−6^
Ephrin receptor signaling	30/19 (18.2)	6.3×10^−6^
Virus entry via endocytic pathway	19/96 (19.8)	9.3×10^−6^
Glioblastoma multiforme signaling	26/163 (16.0)	1.7×10^−5^
HER2 signaling in breast cancer	17/79 (21.5)	2.2×10^−5^
Renal cell carcinoma signaling	16/72 (22.2)	2.3×10^−5^
Clatharin-mediated endocytosis signaling	26/167 (15.6)	4.9×10^−5^
Agrin interactions at neuromuscular junction	15/69 (21.7)	7.6×10^−5^

1 Functional pathway analysis was carried out using Ingenuity Pathway Analysis (http://www.ingenuity.com) on all basal cell-enriched (fold basal/large airway epithelium ratio >5, p<0.01 following Benjamini-Hochberg multiple test correction).

The top 10 canonical pathways were selected on the basis of significance.

2 Ratio refers to the number of pathway genes in the basal cell-enriched signature dataset compared to the total number of genes in the curated pathway.

The dominant GO categories included ectoderm development, epidermis development, regulation of cell cycle, organogenesis, morphogenesis and cell proliferation. The most significantly enriched genes encoding structural proteins were those related to ectoderm development, a GO category characterized by 218 genes, 22 of which overlapped with the human airway basal cell signature (Table S5). The top GO category of ectoderm development included genes coding for components of the cornified envelope such as 3 small proline-rich peptides, SPRR1A (expression ratio 371), SPRR1B (expression ratio 345) and SPRR2B (expression ratio 35), as well as sciellin (expression ratio 194). This is consistent with the results of PCA, which revealed similarities between the airway basal cell and keratinocyte transcriptomes (Figure 3), and the GoSurfer analysis, which detected categories related to ectoderm development, epidermal cell differentiation, epidermis morphogenesis and keratinazation as significantly enriched categories in the human airway basal cell signature (Figure 4).

#### Extracellular matrix (ECM) and Structural Cellular Proteins

The foremost KEGG pathway identified by the GATHER analysis (Table 2) was the focal adhesion pathway, a pathway characterized by 210 genes, of which 41 overlapped with the human airway basal cell signature (Table S3). All of the top significantly enriched KEGG pathways represented closely related structural/functional categories of genes, including adherens junction, extracellular matrix-receptor interactions and regulation of actin cytoskeleton, together with the focal adhesion pathway. These categories constitute fundamental molecular machineries essential for communication of cells with extracellular microenvironment and with each other and also necessary for regulation of cell migration. Consistently, functional categories related to cell motility, such as cytoskeleton organization and biogenesis, cell adhesion, cell projection organization and biogenesis, including pseudopodium and filopodium formation, were among significantly enriched GO annotations in the GoSurfer analysis (Figure 4).

Among extracellular matrix components, 5 laminin subunits corresponding to the α, β1, β3, γ1 and γ2 chains as well as 3 subunits of type IV collagen were overexpressed from 12 to 35-fold in the basal cell signature compared to the differentiated epithelium (Table 5). Extracellular matrix components signal through integrins of which 6 subunits (α3, α5, α6, β1, β4, β6) were overexpressed in basal cells by a factor of up to 51-fold. The initial signaling events from extracellular matrix through integrins result in remodeling of the cytoskeleton [38]. The adaptor proteins actinin, vinculin and filamin were prominent in the human airway basal cell signature as were the signal transduction GTPases rhoC, D, and F. With respect to the cytoskeleton, there were multiple cytokeratin genes in the basal cell signature, including KRT5, 6A, 6B, 7, 16, 17 and 34 with basal/differentiated epithelium expression ratios between 7.8 and 667. Of the classic basal-specific cytokeratins, KRT5 and KRT14, KRT5 was in the human airway basal cell signature (expression ratio 8.6). Although KRT14 was up-regulated in basal cells as compared to the complete differentiated airway epithelium (expression ratio 203), the borderline significance (p = 0.016) precluded it from inclusion in the human airway basal cell signature.

**Table 5:** Extracellular Matrix and Cytoplasmic Structural Proteins in the Human Airway Basal Cell-enriched Signature.^1^

Category	Genes	Mean expression in differentiated epithelium	Mean expression in basal cells	Basal/differentiated epithelium expression ratio
**Extracellular matrix**				
Collagens	COL4A1	0.5	4.3	7.9
	COL4A2	0.7	5.1	7.2
	COL4A6	5.1	39.3	7.6
	COL7A1	1.1	15.9	15.1
	COL12A1	0.4	4.0	9.7
	COL17A1	0.1	31.0	353.0
Thrombospondin	ADAMTS1	0.1	43.1	327.7
	THBS1	0.5	59.4	110.4
Laminins	LAMA3	5.4	148.4	27.6
	LAMB1	1.8	31.3	17.3
	LAMB3	15.9	297.3	18.7
	LAMC1	3.5	45.0	12.9
	LAMC2	11.8	417.2	35.2
Other	TNC	8.6	118.0	13.7
	HSPG2	4.5	26.8	6.0
	SDC1	12.0	110.9	9.3
	CD44	5.9	58.2	9.9
**Receptors**				
Integrins	ITGA3	3.2	50.1	15.8
	ITGA5	0.7	18.3	27.3
	ITGA6	2.6	129.8	50.8
	ITGB1	16.9	216.8	12.8
	ITGB4	11.0	106.2	9.6
	ITGB6	5.4	44.6	8.3
**Adaptors**				
Filamin and related	FLNA	2.0	38.0	19.1
	FLNB	13.4	103.5	7.7
	FBLIM1	0.6	9.2	14.9
	FILIP1L	0.5	3.8	8.2
Vinculin	VCL	9.6	55.8	5.8
Actinins	ACTN1	4.2	70.8	17.0
**Signal transduction**				
G-proteins	RAC2	6.9	50.2	7.3
	RALA	5.5	47.9	8.6
	RAP2A	6.9	37.0	5.4
	RAP2B	6.5	55.8	8.5
	RHOC	18.8	100.3	5.3
	RHOD	2.5	32.6	13.0
	RHOF	0.3	3.1	11.5
	RRAS	1.9	59.5	30.8
**Cytoskeletal components**				
Keratins	KRT34	0.1	0.6	7.8
	KRT17	10.7	537.9	50.3
	KRT16	0.1	88.7	635.4
	KRT7	15.6	117.8	7.6
	KRT6B	1.8	353.0	196.2
	KRT6A	1.1	724.4	667.2
	KRT5	44.5	382.6	8.6
Other	ARPC2	27.7	187.1	6.8
	ARPC5L	9.0	52.2	5.8
	DSP	69.6	359.2	5.2
	MSN	8.3	87.2	10.5
	PLS3	24.5	221.7	9.1
	DBN1	1.5	19.1	12.4

1 Genes identified by GATHER KEGG categories (Table 2, Table S3), Gene Ontology categories (Table 3, Table S5), and/or Canonical Pathways (Table 4, Table S6).

#### Receptors and Ligands

The Ingenuity Pathway Analysis revealed synchronous enrichment of a variety of ligands (Table 6) and their cognate transmembrane receptors (Table 7) in the human airway basal cell signature. In addition to the extracellular matrix protein – integrin interactions described above, the basal cell signature included several growth factor – receptor interactions, as shown by the enrichment of a surprisingly broad spectrum of the epidermal growth factor (EGF) family ligands such as epiregulin (246-fold up-regulated compared to the differentiated epithelium), amphiregulin (133-fold), neuregulin (54-fold), heparin-binding EGF-like growth factor (176-fold) and the classic EGF receptor (EGFR; 10.7-fold). By contrast, other EGFR family receptors such as ERBB2, ERBB3 and ERBB4 were expressed at lower levels in basal cells compared to the intact epithelium. As a further example, genes encoding both transforming growth factor beta (TGF-β) and its receptor were present in the human airway basal cell signature (Tables 6, 7).

**Table 6:** Ligands in the Human Airway Basal Cell-enriched Transcriptome.^1^

Ligand family	Genes	Mean expression in differentiated epithelium	Mean expression in basal cells	Basal/differentiated epithelium expression ratio
Vascular endothelial growth factors	VEGFA	4.2	35.1	8.3
	VEGFC	1.3	9.1	7.2
Angiopoietin	ANGPTL4	0.8	4.6	5.8
Platelet derived growth factors	PDGFA	3.0	19.4	6.4
	PDGFC	12.7	78.6	6.2
Placental growth factor	PGF	0.4	7.3	17.9
Wingless-type MMTV integration site family	WNT7A	0.2	2.9	15.7
Bone morphogenic proteins	BMP1	0.4	5.2	12.3
	BMP2	0.3	8.2	30.7
	GDF15	13.6	135.2	10.0
Transforming growth factors	TGFB1	2.6	14.9	5.7
	TGFB2	0.8	4.6	6.0
	TGFA	7.4	59.7	8.1
	ARTN	0.4	9.0	21.7
Epidermal growth factors	EREG	0.1	34.6	246.0
	NRG1	0.4	19.8	53.4
	AREG	2.7	354.7	133.9
	HBEGF	3.8	65.4	17.3
	AREGB	0.5	10.9	20.4
Dickkopf homologs	DKK1	1.1	91.4	82.6
	DKK3	0.3	7.3	27.9
Fibroblast growth factors	FGF2	0.2	1.8	7.7
	FGF11	1.3	8.3	6.2
Insulin growth factors	IGFL1	0.1	2.7	18.5
Cholecystokinin	CCK	0.2	2.8	18.4
Endothelin	EDN1	0.2	7.5	31.7
Galanin	GAL	0.3	5.1	18.6
Neuromedin	NMU	1.0	11.7	11.4
Peptide Y	PYY	0.1	1.0	7.1
Follistatin/inhibin	FST	0.6	27.1	44.2
	INHBE	0.1	0.7	7.1
Cytokines	IL1B	1.8	20.9	11.7
	IL18	4.0	26.5	6.6
	IFNE	0.5	2.4	5.2
Adrenomedullin	ADM	1.9	53.3	28.3
Endothelin	EDN1	0.2	7.5	31.7
Granulin	GRN	6.2	37.6	6.1
Jagged	JAG1	23.3	249.3	10.7
Parathyroid hormone like hormone	PTHLH	0.6	18.6	32.3

1 Genes identified by GATHER KEGG categories (Table 2, Table S3), Gene Ontology categories (Table 3, Table S5) and/or Canonical Pathways (Table 4, Table S6).

**Table 7:** Receptors in the Human Airway Basal Cell-enriched Transcriptome.^1^

Receptor family	Genes	Mean expression in differentiated epithelium	Mean expression in basal cells	Basal/differentiated epithelium expression ratio
EGF receptor	EGFR	9.2	93.6	10.2
TGFb receptor	TGFBR1	7.3	54.4	7.5
TNF receptors	TNFRSF10B	8.5	130.0	15.3
	TNFRSF10D	1.5	23.0	15.3
	TNFRSF12A	2.7	69.4	25.5
	TNFRSF10A	1.1	5.9	5.4
Ephrin receptors	EPHB4	3.2	19.1	6.0
	EPHB2	0.8	6.0	7.7
Adrenergic receptor	ADRB2	6.6	38.4	5.9
Leptin receptor	LEPR	0.6	4.5	7.6
Vasopressin receptor	AVPR1B	0.4	1.9	5.3
Histamine receptor	HRH1	0.5	2.3	5.1
Serotonin receptor	HTR7	0.3	2.3	6.6
G protein-coupled receptors	GPR37	0.5	3.0	5.9
	GPR87	5.5	47.4	8.7
	GPR115	0.3	9.5	32.3
	GPR126	1.5	15.0	10.2
	GPR153	1.2	9.4	8.2
	GPRC5A	8.6	66.6	7.7
Light sensor	OPN3	4.7	26.6	5.6
Cytokine receptors	IL13RA2	0.2	32.9	200.8
	IL1RL1	0.2	106.3	449.9
	OSMR	1.4	16.2	12.0
	IL20RB	2.3	67.3	28.7
IGF receptors	IGF2R	11.3	57.8	5.1
Tyrosine kinase	AXL	5.7	34.6	6.0
HGF receptor	MET	30.9	195.3	6.3
VEGF receptor	NRP1	0.2	2.7	13.0
Opioid growth factor receptor	OGFRL1	2.4	14.8	6.2
Platelet endothelial aggregation receptor	PEAR1	0.4	2.8	7.4
LDL receptor	LDLR	21.1	138.2	6.6
Thrombin receptor	THBD	1.7	53.5	31.5
Protein C receptor	PROCR	0.8	26.1	33.1

1 Genes identified by GATHER KEGG categories (Table 2, Table S3), Gene Ontology categories (Table 3, Table S5) and/or Canonical Pathways (Table 4, Table S6).

Ingenuity Pathway Analysis pointed to several receptor/ligand combinations and signaling pathways that may be critical for basal cell function (Table 4). The most significant was EGFR-related neuregulin signaling pathway, which was markedly overrepresented (p<10^−7^) with 22 out of 103 genes in the pathway up-regulated in the basal cell transcriptome compared to differentiated epithelium (Table S6). As expected, the closely related HER2 signaling pathway, containing many of the same components as the neuregulin pathway, was also among the most significant canonical pathways (Table 4). Consistent with the KEGG data, Ingenuity Pathway Analysis also detected overrepresentation of members of the integrin signaling pathway, which overlaps extensively with the ephrin signaling pathway. Together, the data suggest that canonical pathways encoded by the human airway basal cell-enriched genes represent a network of functionally-related molecular features associated with a limited number of relatively specific signaling modules. The analysis suggests that such unifying signaling modules in human airway basal cells are most likely represented, at least in part, by the signature elements encoding the extracellular matrix-receptor and EGFR molecular pathways.

The compiled ligand/receptor list included a number of genes that are classically associated with the neuroendocrine system but have potential relevance to pharmacological effects on the lung, such as the adrenergic receptor (ADRB2; expression ratio 5.9-fold), and histamine receptor (HRH1; expression ratio 5.1-fold). The most striking basal-enriched genes in this category were galanin (expression ratio 18.6), a secreted peptide with diverse neuroendocrine functions, and cholecystokinin (expression ratio 18.4) classically thought of as a peptide involved in the functions of the gut [39]. Interestingly, none of these genes were enriched in mouse airway basal cell signature.

The basal cell signature included multiple transmembrane receptors, including those with transmembrane tyrosine kinase signaling elements such as the EGFR and VEGFR pathways, as well as the TGF-β and G protein-coupled receptors (Figure 4, Table 6). Among the G protein-coupled receptors in the basal cell signature were the arginine vasopressin receptor (AVPR1B, expression ratio 5.4), the non-retinal light-sensitive opsin 3 (OPN, expression ratio 5.6), the serotonin receptor (HTR7, expression ratio 6.6), as well as several orphan G protein-coupled receptors, including GRP115 (expression ratio 32) and GPR126 (expression ratio 10.5). The GATHER analysis also revealed significant enrichment of the MAPK signaling pathway in the human airway basal cell signature (Table 3). This included components signaling from the plasma membrane (TGF-β1 and its receptor enriched by 5.7 and 7.5-fold, respectively) through the cytoplasm to MAP2K1 (enriched 7.0-fold) and two 2 MAP kinases (MAPK6 and MAPK13, enriched 5.9 and 6.2-fold), and to the nucleus (transcription factor ATF4, enriched 7.1-fold). Notably, except for TGF-β, the elements of these signaling cascades were not among airway basal cell-enriched genes in mice (Table S2) [7].

#### Ion transport

The human airway basal cell signature was also enriched for at least 35 genes encoding various ion transporters including potassium channels and solute carrier proteins (Table 8). Three subunits of the cationic amino acid transporter SLC7A were highly enriched (SLC7A5 by 314-fold, SLC7A11 by 141-fold and SLC7A1 by 6.8-fold). Four subunits of the monocarboxylic acid transporter SLC16A were also overexpressed. Interestingly, CFTR, the cAMP-mediated Cl^−^ ion channel, which is central to the pathogenesis of cystic fibrosis [40], was not expressed in the human airway basal cell signature.

**Table 8:** Ion Transport-related Genes in the Human Airway Basal Cell-enriched Transcriptome.^1^

Symbol	Gene title	Mean expression in differentiated epithelium	Mean expression in basal cells	Basal/differentiated epithelium expression ratio
SLC7A5	solute carrier family 7 (cationic amino acid transporter, y+ system), member 5	0.6	201.0	314.7
SLC7A11	solute carrier family 7, (cationic amino acid transporter, y+ system) member 11	0.8	116.5	141.2
SLC16A1	solute carrier family 16, member 1 (monocarboxylic acid transporter 1)	0.3	23.8	69.8
SLC16A4	solute carrier family 16, member 4 (monocarboxylic acid transporter 5)	0.5	18.4	34.5
SLC4A5	Solute carrier family 4, sodium bicarbonate cotransporter, member 5	0.1	4.1	30.5
SLC3A2	solute carrier family 3 (activators of dibasic and neutral amino acid transport), member 2	2.9	84.6	29.1
SLC22A3	solute carrier family 22 (extraneuronal monoamine transporter), member 3	0.1	2.5	27.6
SLC6A15	solute carrier family 6 (neutral amino acid transporter), member 15	0.3	7.5	27.2
KCNG1	potassium voltage-gated channel, subfamily G, member 1	0.3	7.3	21.2
SLC39A14	solute carrier family 39 (zinc transporter), member 14	2.2	46.2	20.8
SLC2A1	solute carrier family 2 (facilitated glucose transporter), member 1	0.3	5.5	17.7
SLC38A1	solute carrier family 38, member 1	11.4	181.2	16.0
ABCA12	ATP-binding cassette, sub-family A (ABC1), member 12	0.6	7.1	12.8
CLIC4	chloride intracellular channel 4	1.3	15.8	12.2
SLC35F2	solute carrier family 35, member F2	1.2	13.5	11.7
AMMECR1	Alport syndrome, mental retardation, midface hypoplasia and elliptocytosis chromosomal region gene 1	5.2	57.8	11.1
SLC38A5	solute carrier family 38, member 5	0.8	6.6	8.6
SLC4A7	solute carrier family 4, sodium bicarbonate cotransporter, member 7	0.8	7.0	8.3
SLC25A15	solute carrier family 25 (mitochondrial carrier; ornithine transporter) member 15	0.2	1.2	8.0
KCNK6	potassium channel, subfamily K, member 6	1.3	9.8	7.3
SLC16A3	solute carrier family 16, member 3 (monocarboxylic acid transporter 4)	2.3	16.5	7.3
SLC7A1	solute carrier family 7 (cationic amino acid transporter, y+ system), member 1	19.2	130.4	6.8
SLC38A2	solute carrier family 38, member 2	46.6	314.2	6.7
SLC25A43	solute carrier family 25, member 43	3.4	22.2	6.6
KCMF1	potassium channel modulatory factor 1	30.2	190.0	6.3
SLC2A9	Solute carrier family 2 (facilitated glucose transporter), member 9	0.4	2.3	6.2
SLCO1B3	solute carrier organic anion transporter family, member 1B3	0.4	2.6	6.1
SLC9A1	solute carrier family 9 (sodium/hydrogen exchanger), member 1	2.8	16.9	5.9
KCNQ5	potassium voltage-gated channel, KQT-like subfamily, member 5	0.3	1.9	5.5
SLC6A8	solute carrier family 6 (neurotransmitter transporter, creatine), member 8	3.5	19.2	5.5
SLC35E4	solute carrier family 35, member E4	0.3	1.5	5.5
ABCC3	ATP-binding cassette, sub-family C (CFTR/MRP), member 3	3.3	17.6	5.4
SLC16A2	solute carrier family 16, member 2 (monocarboxylic acid transporter 8)	1.2	6.4	5.2
KCTD9	potassium channel tetramerisation domain containing 9	4.8	25.1	5.2
SLC10A3	solute carrier family 10 (sodium/bile acid cotransporter family), member 3	2.0	10.2	5.1

1 Genes identified by GATHER KEGG categories (Table 2, Table S3), Gene Ontology categories (Table 3, Table S5), and/or Canonical Pathways (Table 4, Table S6).

#### Transcription factors

The unique phenotypic and functional properties of airway basal cells suggest there are likely transcription factors specific for this cell type. Interestingly, the human airway basal cell signature included at least 70 transcription factors (Table 9). As expected, the classic basal cell-specific transcription factor basonuclin was the most overexpressed with a basal/differentiated epithelium expression ratio of 69.7. TP63 was another recognized basal cell-specific factor which was overexpressed, with a ratio of 8.9.

**Table 9:** Transcription Factors in the Human Airway Basal Cell-enriched Transcriptome.^1^

Symbol	Gene title	Mean expression in differentiated epithelium	Mean expression in basal cells	Basal/differentiated epithelium expression ratio
BNC1	basonuclin 1	0.5	35.1	69.7
ARNTL2	aryl hydrocarbon receptor nuclear translocator-like 2	0.8	38.1	44.9
FOSL1	FOS-like antigen 1	0.7	22.9	30.7
HMGA2	high mobility group AT-hook 2	0.1	2.8	27.9
SOX7	SRY (sex determining region Y)-box 7	2.1	41.7	19.7
ETV5	Ets variant 5	0.3	6.0	18.3
KLF7	Kruppel-like factor 7 (ubiquitous)	2.0	34.1	17.3
HSF2BP	heat shock transcription factor 2 binding protein	0.6	9.9	15.3
ETS1	v-ets erythroblastosis virus E26 oncogene homolog 1 (avian)	6.4	94.6	14.8
SOX15	SRY (sex determining region Y)-box 15	1.9	27.0	14.0
CEBPG	CCAAT/enhancer binding protein (C/EBP), gamma	4.3	54.0	12.6
SNAI2	snail homolog 2 (Drosophila)	6.0	71.9	12.0
FOXA2	forkhead box A2	2.1	24.0	11.4
ID3	inhibitor of DNA binding 3, dominant negative helix-loop-helix protein	1.7	19.3	11.1
ETV4	ets variant 4	0.3	3.5	10.8
NPAS2	neuronal PAS domain protein 2	0.4	4.7	10.7
MXD1	MAX dimerization protein 1	3.3	35.4	10.7
ZNF185	zinc finger protein 185 (LIM domain)	8.7	89.2	10.3
KLF9	Kruppel-like factor 9	2.5	23.9	9.7
KLF9	Kruppel-like factor 9	2.5	23.9	9.7
ZNF215	zinc finger protein 215	0.3	2.9	9.5
MYC	v-myc myelocytomatosis viral oncogene homolog (avian)	9.6	90.1	9.4
DR1	down-regulator of transcription 1, TBP-binding (negative cofactor 2)	3.0	28.2	9.3
PAWR	PRKC, apoptosis, WT1, regulator	5.2	47.8	9.2
TAF1D	TATA box binding protein (TBP)-associated factor, RNA polymerase I, D, 41 kDa	7.7	70.4	9.1
CBX4	chromobox homolog 4 (Pc class homolog, Drosophila)	17.1	154.6	9.0
TP63	tumor protein p63	14.9	132.2	8.9
HIF1α	hypoxia inducible factor 1, alpha subunit (basic helix-loop-helix transcription factor)	30.0	258.6	8.6
HES2	hairy and enhancer of split 2 (Drosophila)	1.6	13.2	8.5
E2F7	E2F transcription factor 7	2.5	20.7	8.2
CCRN4L	CCR4 carbon catabolite repression 4-like (S. cerevisiae)	0.4	3.3	8.1
ZNF83	zinc finger protein 83	1.3	10.0	8.0

1 Genes identified by GATHER KEGG categories (Table 2, Table S3), Gene Ontology categories (Table 3, Table S5) and/or Canonical Pathways (Table 4, Table S6). The top genes based on expression ratio are shown.

Other transcription factor-encoding genes identified in the human airway basal cell signature not previously associated with basal cells included ARNTL2 (also known as MOP9/BMAL2, 44.9-fold enrichment), a transcription factor implicated in circadian transcription [41]. Another was FOSL1/FRA-1 (30.7-fold enrichment), a transcription factor activated in a c-Fos-dependent manner during cellular transformation and osteoclast differentiation [42], [43]. Both of these transcription factor genes are also enriched in mouse airway basal cells (Table S2).

Consistent with the stem/progenitor function of basal cells, the airway basal cell signature included 2 transcription factors critical for the regulation of embryonic stem cell functions. The high-mobility group protein A2 (HMGA2), known to regulate key developmental pathways in human embryonic stem cells and participate in transformation in lung cancer [44]–[46], was enriched in the human airway basal cells (27.9-fold enrichment), but not in the murine counterpart. Intriguingly, also included was the oncogenic transcription factor MYC, known to suppress differentiation of embryonic stem cells (ESC) while increasing their pluripotency and self-renewal [47]. SNAI2/SLUG, a transcription factor driving epithelial-mesenchymal transition (EMT) [48], was enriched in both the human and mouse airway basal cell signatures (Table S2) consistent with the overrepresentation of functional categories related to mesenchymal cell differentiation and EMT in the human airway basal cell signature (Table 9, Table S2, Figure 4).

In addition to individual transcription factor-encoding genes, a number of transcription factor families were enriched in the human airway basal cell signature, including the forkhead box (FOX) and SRY-related HMG-box (SOX) family genes (Table 9). The pattern of enriched genes belonging to the FOX family (FOXA2, FOXL2, FOXN2, FOXD1, FOXQ1) and SOX family (SOX7, SOX15, SOX4) was distinct from the mouse airway basal cell signature (only FOXO1 and SOX6). Among other highly basal cell-enriched transcription factors were several members of the ETS family, including ETV5 (18.3-fold expression ratio), ETS1 (14.8-fold), ETV4 (10.8-fold), TCF3 (6.6-fold) and ELF4 (6.2-fold). Multiple Kruppel-like factors were overexpressed in basal cells including KLF7, KLF9, KLF8, KLF13, KLF6, with expression ratios of 17.3, 9.7, 7.7, 5.9 and 5.5 respectively, but these transcription factors are not in the mouse basal cell gene list.

## Discussion

Basal cells play a central role in airway epithelial biology [1], [4]–[8]. The basal cell population includes stem/progenitor cells capable of self-renewal, and with the appropriate signals, differentiation into specialized ciliated and secretory cells during physiologic turnover and repair [4], [7], [8], [26], [32], [49]. The airway basal cells directly interact with the extracellular matrix, but are also capable of extending elements to sample the airway epithelial surface, and are adept at migrating into injured areas [4], [50], [51]. The focus of the present study was to characterize the human airway basal cell transcriptome. To accomplish this, the transcriptome of well characterized cultured human airway basal cells was compared to that of the differentiated airway epithelium from which they were derived. From this analysis we identified 1,161 named genes with expression ratios (basal cell/differentiated epithelium) of greater than 5, which we defined as the “human airway basal cell signature.” While some of the differences between differentiated epithelium and cultured basal cells may be attributed to the culture conditions, analysis of the human airway basal cell signature identified a number of genes/pathways that are clearly relevant to the biology and function of airway epithelial basal cells.

The legitimacy of the human airway basal cell signature identified by this analysis was supported by multiple lines of evidence. First, there was a dramatic decline in expression levels of the basal cell-enriched genes upon induction of differentiation following the culture on ALI in parallel with acquisition of the morphologic phenotype of a ciliated airway epithelium. Second, the genes specific for ciliated and secretory airway epithelial cells were down-regulated in the basal cell population compared to the complete differentiated airway. Third, genome-wide PCA revealed a high degree of similarity of the human airway basal cell transcriptome to that of cell lines with basal cell-like features. Fourth, comparison of the human airway basal cell signature with that recently characterized for mouse airway basal cells [7] revealed a considerable overlap of genes between the 2 species. This is remarkable given the differences in the methodologies utilized for isolation and characterization of airway basal cells in both studies, and the known differences in human *vs* murine airway epithelial populations [1], [7], [8]. Indeed, many of the differences between the mouse and human basal cell transcriptomes involve different members of gene families (e.g., WNT and KRT) where overlapping species-specific roles may be critical.

### Overall Characteristics of Human Airway Basal Cells

Despite similarities to basal-like cells of other organs and murine basal cells, the human airway basal cell signature has several unique features. PCA analysis demonstrated that the human airway basal cell signature entirely segregated airway basal cells from all other cell types analyzed, including the basal-like CD44+ breast epithelial stem cells and p63-overexpressing cervical cancer cells, the transcriptomes of which were similar to human airway basal cells at the genome-wide level. In both genome-wide and airway basal cell signature-restricted analyses, the airway basal cells clustered very distantly from basal-like breast carcinoma. Interestingly, the airway basal cells distributed more closely to skin keratinocytes, with a higher degree of transcriptome similarity compared to the complete differentiated large airway epithelium the basal cells were derived from. Several molecular features detected in the airway basal cell signature were responsible for this similarity, including the unique pattern of cytokeratin-encoding genes and elements of the cornified envelope normally expressed by the stratified epithelia of the skin [52]. Consistent with these findings, functional analysis revealed significant overrepresentation of genes related to ectoderm development, epidermis morphogenesis and keratinization in the human airway basal cell signature. Enrichment of these categories is likely indicative of the phenotypic plasticity of airway basal cells, which, under certain conditions, such as those related to tissue injury and regeneration, might temporarily acquire the phenotype of squamous cell-like reparatory progenitor cells [53]. These characteristics can be recapitulated in vitro, when signals necessary for the differentiation of airway basal cells towards the mucociliary epithelium are not present [54], [55].

The human airway basal cell signature included a group of genes encoding for components of the cornified envelope belonging to the epidermal differentiation complex, including 3 small proline-rich peptides (SPRR1A, SPRR1B, SPRR2B) and sciellin contributing to the gene ontology category related to ectoderm development. Expression of these genes in the non-stratified epithelia is usually associated with acquisition of the squamous phenotype [56], as SPRR1A is overexpressed in the airway epithelium in association with squamous metaplasia [57]. Enrichment of these genes in the airway basal cells is consistent with data suggesting squamous metaplastic changes is the airway epithelium is of basal cell origin [53], [55], [58]. In the absence of signals critical for mucociliary differentiation such as retinoic acid, certain growth factors and exposure of the apical surface to air, airway basal cells can acquire a squamous cell phenotype as a default differentiation pathway [54], [59]. It is possible that such a phenotype was partially acquired by human airway basal cells in the *in vitro* system. Although the basal cell cultures were not passaged in the present study, a recent study revealed that after several passages human airway epithelial cells express some molecular features of squamous cells [55].

The expression pattern of cytokeratin-encoding genes in human airway basal cells was different from that of murine airway basal cells. The mouse basal cell transcriptome includes keratins 5, 14, 17 and 31, of which only keratin 5 and 17, members of the cytokeratin family classically associated with the basal cell phenotype [7], [56], were in human airway basal cell signature. Relatively high variability of the cytokeratin 14 gene expression in the human airway basal cells, reflected by higher p value of the significance of enrichment as compared to other basal cell-specific genes of this family, is consistent with studies showing that cytokeratin 14 is expressed in only a subset of airway basal cells but is up-regulated during epithelial pathological processes such as squamous metaplasia and tumorigenesis [8], [53]. Interestingly, the genes encoding cytokeratins 6 and 16, found in the proliferating cell compartments of various epithelia [56], [60], [61], were the top human airway basal cell signature genes but were not present among the mouse basal cell-enriched genes.

### Basal Cell – Extracellular Matrix Relationships

Functional analysis of the human airway basal cell signature using a diverse set of analytic tools identified a number of gene categories relevant to function of the cells to maintain the structural integrity of the airway epithelium. Consistent with their role in establishing contacts with the various extracellular components as well as between airway epithelial cells [4], the human airway basal cell signature was enriched in functional categories related to the extracellular matrix-receptor interactions and cell-cell communications. Included in this category were biological functions relevant to anchorage of the epithelium to the extracellular matrix. This function requires specific interactions that are mediated by integrins through specialized protein domains of the basal membrane compartment, which bind to the corresponding components of extracellular matrix, activating binding of the intracellular domain of integrin to the cytoskeleton via adapter proteins [38], [62], [63]. All components of this pathway are expressed in the basal cell signature including the extracellular laminins and collagen, the integrins as well as the actinin, vinculin and filamin adapter proteins. Notably, the major basal cell-specific integrin ITGA6, encoding hemidesmosomes, structures critical for the anchorage of the intermediate and luminal cells to the basal cell layer [64], and relevant to the stem/progenitor cell phenotype of basal cells in tissues such as the breast and skin [65]–[67], was present in the human basal cell signature, as it is in mouse [7]. Likewise, the surface antigen CD44, encoding the receptor for various plasma membrane-associated and extracellular components, including hyaluronic acid [68], and associated with the phenotype of tumor-initiating basal cells in the prostate and breast [69]–[71], was expressed in the airway basal cells of both species. In support of the role of CD44 in the function of airway basal cells as stem/progenitor cells, CD44 is up-regulated during airway epithelial repair [72].

Interestingly, genes encoding integrins ITGA5 and ITGB6 were enriched in the human, but not mouse, airway basal cell signature, suggesting that the surface phenotype of airway basal cells likely differs between these 2 species. The integrin gene expression profile of human airway basal cells in the present study is similar to that described for basal cells based on immunohistochemical analysis [73]. Although the relevance of ITGA5 and ITGB6 to airway basal cell biology is largely unknown, several independent lines of evidence indicate their potential relevance to the stem/progenitor cell and tissue repair functions of airway basal cells. ITGA5 mediates fibronectin-dependent epithelial cell proliferation through activation of EGFR [74], activates a NF-κB-dependent transcriptional program regulating angiogenesis [75], and promotes cell migration in a HIF1α-dependent manner [76]. Consistent with this data, genes encoding elements of the EFGR and NF-κB signaling pathways, functional categories related to cell proliferation, migration and angiogenesis, as well as transcriptional factor HIF1α, were enriched in the human airway basal cell signature. Integrin alpha 5 beta 6, encoded by the ITGA5 and ITGB6 subunit genes, is required for spacially-restricted activation of latent TGF-β in lung [77]. Given that both ITGA5 and ITGB6 genes, as well as the TGFB1 gene are components of the human airway basal cell signature, it is possible that ITGA5+ ITGB6+ cells represent a airway basal cell population that regulate their own TGF-β signaling in an autocrine manner, potentially contributing to local control of inflammation and lung fibrosis [77]. Altered expression of CD44 as well elements of TGF-β and EGFR signaling play a role in airway remodeling in asthma [78], [79].

### Ligands and Receptors

Another remarkable feature of the human airway basal cell signature is that it includes the genes encoding biologically active ligands and, in some cases, the corresponding receptors. This provides the basis for a model in which airway basal cells regulate their own stem/progenitor capacity in a cell-autonomous manner as well as the activities of adjacent differentiated epithelial cells. The most striking example was the enrichment of EGFR expression, paralleled by overexpression of a broad spectrum of the epidermal growth factor family ligands, including epiregulin, amphiregulin, neuregulin and heparin-binding EGF-like growth factor (HB-EGF). The relevance of amphiregulin signaling to epithelial self renewal is well established. Amphiregulin mediates self-renewal in stem cell-like mammary epithelial cells [80], and has been implicated in epithelial remodeling in asthma, with elevated serum levels immediately following asthma attacks and in mediating proliferation of human bronchial epithelium [81]. In a murine bleomycin lung injury model, amphiregulin expression increased following injury, and administration of exogenous amphiregulin improved survival [82]. By contrast, the receptors for neuregulin (ERBB2 and ERBB3) [83], are expressed at lower levels in basal cells that in differentiated epithelium so neuregulin may be a secreted by basal cells and signal to differentiated cells.

### Ion Channels

Another notable feature of the basal cell signature was the overexpression of a number of ion channels. The most basal-enriched ion transporter gene, SLC7A5/LAT1, is a cationic amino acid transporter that has previously been used to distinguish squamous lung cancer from adenocarcinoma [84]. In addition to transporting amino acids, SLC7A5 may transport thyroxine derivatives, although the implications for epithelial biology are not clear [85]. Interestingly, CFTR, the cAMP Cl^−^ gene responsible for cystic fibrosis [40], is not part of the human airway basal cell signature. This is consistent with the location of native CFTR protein at the apical surface of ciliated cells [86] and suggests CFTR is not critical to renewal functions in airway epithelium.

### Transcription Factors

Transcriptome analysis of the basal cell and differentiated airway epithelium identified at least 70 transcription factors in the basal cell signature, including transcription factors implicated in the regulation of cell proliferation, differentiation and maintenance of the stem cell phenotype. The zinc finger transcription factor basonuclin 1, a known basal cell-specific transcription factor which plays a role in epithelial cell differentiation and proliferation [87], had the highest degree of enrichment among transcription factors. Inclusion of the basal cell-specific transcription factor p63, known to be essential for the proliferation potential of stem cells in stratified epithelium [88], helps to explain why airway basal cells exhibit a number of molecular features typical for the squamous phenotype.

The role of Kruppel and Kruppel-like factors in basal cell biology is well established [89], [90]. The human airway basal cell signature included expression of KLF5 but not KLF4. KLF5 is a basal-specific factor in squamous epithelium that mediates a proliferative gene expression profile [90]. Among the targets of KLF5 are the EGFR gene and the MEK/ERK pathway, components of the human airway basal cell signature. The absence of KLF4 in the basal cell signature is consistent with its action to directly antagonize KLF5, and with murine data that deletion of KLF4 results in basal cell hyperplasia [89].

The human airway basal cell signature also included the genes for transcription factors related to stem cell function including MYC, known to suppress differentiation of embryonic stem cells, while increasing their pluripotency and self-renewal [47] and HIF1α, a hypoxia-sensitive transcription factor which modulates telomerase function of embryonic stem cells [91]. In addition, the SOX family of transcription factors are known to play a key role in the regulation of embryonic development and cell fate [92]. SOX4, SOX7 and SOX15 are known to be highly expressed in adult lung [17] and were all highly enriched in basal cells, an observation relevant to the function of SOX4 interacting with β-catenin to control gene expression [93]. Interestingly, β-catenin signaling has been shown to play a role in the regulation of the expansion of both ESC and tissue stem cells [94], [95].

### Conclusion

In summary, we have characterized the human airway basal cell transcriptome, identifying genes and pathways enriched in this cell population. Functional annotation of the human airway basal cell signature points to molecular pathways likely important for known and potentially novel aspects of basal cell biology. The data presented here provide an important tool for future analyses of human airway basal cell functions and may help elucidate the origins and mechanisms of respiratory diseases associated with altered structural and functional integrity of the airway epithelial barrier.

## Supporting Information

Table S1 **The Human Airway Epithelium Basal Cell Signature.**
(DOC)

Table S2 **Basal Cell-specific Genes Shared by Human and Mouse.**
(DOC)

Table S3 **Genes Comprising the Significant GATHER KEGG Categories of the Human Basal Cell Signature.**
(DOC)

Table S4 **All Significant Gene Ontology Categories of the Human Airway Basal Cell Signature.**
(DOC)

Table S5 **Genes Comprising Top Gene Ontology Categories in the Human Basal Cell Signature.**
(DOC)

Table S6 **Genes Comprising the Significant Canonical Pathways of the Human Basal Cell Signature.**
(DOC)
