# Supplementary material for: The Human Airway Epithelial Basal Cell Transcriptome
Source: PLoS One. 2011 May 4;6(5):e18378. doi: 10.1371/journal.pone.0018378 (PMC3087716; doi:10.1371/journal.pone.0018378)
Supplement: Table S1 — The Human Airway Epithelium Basal Cell Signature. (DOC) [file pone.0018378.s001.doc]

| **ProbeSetID** | **Gene symbol** | **Gene title** | **Mean expression in differentiated epithelium** | **Mean expression in basal cells** | **Basal / differentiated epithelium expression ratio** | **p value^2^** | **GO biological function^3^** | **GO cellular compartment^4^** |
| --- | --- | --- | --- | --- | --- | --- | --- | --- |
|  |  |  |  |  |  |  |  |  |
| 214580_x_at | KRT6A/// KRT6B/// KRT6C | keratin 6A///keratin 6B///keratin 6C | 0.44 | 311.4 | 707.9 | 3.3 x 10^-7^ | cytoskeleton organization | intermediate filament |
| 209125_at | KRT6A | keratin 6A | 1.09 | 724.4 | 667.2 | 3.2 x 10^-9^ | ectoderm development | intermediate filament |
| 209800_at | KRT16 | keratin 16 | 0.14 | 88.7 | 635.4 | 2.7 x 10^-8^ | cytoskeleton organization | intermediate filament |
| 207526_s_at | IL1RL1 | interleukin 1 receptor-like 1 | 0.24 | 106.3 | 449.9 | 7.0 x 10^-4^ | immune response | extracellular region |
| 213796_at | SPRR1A | small proline-rich protein 1A | 0.23 | 83.9 | 371.4 | 2.2 x 10^-6^ | epidermis development | cornified envelope |
| 204636_at | COL17A1 | collagen, type XVII, alpha 1 | 0.09 | 31.0 | 353.0 | 6.5 x 10^-6^ | cell-matrix adhesion | extracellular region |
| 205064_at | SPRR1B | small proline-rich protein 1B (cornifin) | 0.68 | 234.3 | 345.5 | 5.4 x 10^-8^ | epidermis development | cornified envelope |
| 222162_s_at | ADAMTS1 | ADAM metallopeptidase with thrombospondin type 1 motif, 1 | 0.13 | 43.1 | 327.7 | 4.8 x 10^-6^ | ovulation from ovarian follicle | extracellular region |
| 201195_s_at | SLC7A5 | solute carrier family 7 (cationic amino acid transporter, y+ system), member 5 | 0.64 | 201.0 | 314.7 | 6.7 x 10^-8^ | amino acid metabolic process | cytoplasm |
| 203438_at | STC2 | stanniocalcin 2 | 0.08 | 21.1 | 277.1 | 2.6 x 10^-7^ | cell surface receptor linked signal transduction | extracellular region |
| 203234_at | UPP1 | uridine phosphorylase 1 | 0.95 | 254.7 | 268.9 | 8.5 x 10^-9^ | nucleobase, nucleoside, nucleotide and nucleic acid metabolic process | cytoplasm |
| 205767_at | EREG | epiregulin | 0.14 | 34.6 | 246.0 | 8.0 x 10^-7^ | angiogenesis | extracellular region |
| 235075_at | DSG3 | desmoglein 3 (pemphigus vulgaris antigen) | 0.52 | 114.9 | 222.2 | 5.5 x 10^-5^ | cell adhesion | cytosol |
| 205778_at | KLK7 | kallikrein-related peptidase 7 | 0.11 | 24.2 | 221.3 | 3.6 x 10^-6^ | proteolysis | extracellular region |
| 206172_at | IL13RA2 | interleukin 13 receptor, alpha 2 | 0.16 | 32.9 | 200.8 | 9.0 x 10^-4^ |  | extracellular space |
| 209126_x_at | KRT6B | keratin 6B | 1.80 | 353.0 | 196.2 | 3.6 x 10^-10^ | ectoderm development | intermediate filament |
| 206884_s_at | SCEL | sciellin | 0.38 | 73.3 | 194.6 | 2.0 x 10^-7^ | epidermis development | cornified envelope |
| 212097_at | CAV1 | caveolin 1, caveolae protein, 22kDa | 1.05 | 177.4 | 169.5 | 1.0 x 10^-3^ | MAPKKK cascade | Golgi membrane |
| 1555788_a_at | TRIB3 | tribbles homolog 3 (Drosophila) | 0.18 | 28.9 | 161.5 | 1.0 x 10^-8^ | transcription | nucleus |
| 203065_s_at | CAV1 | caveolin 1, caveolae protein, 22kDa | 0.49 | 75.4 | 154.1 | 2.0 x 10^-4^ | MAPKKK cascade | Golgi membrane |
| 217678_at | SLC7A11 | solute carrier family 7, (cationic amino acid transporter, y+ system) member 11 | 0.83 | 116.5 | 141.2 | 4.1 x 10^-5^ | transport | membrane |
| 205239_at | AREG | amphiregulin | 2.65 | 354.7 | 133.9 | 8.5 x 10^-10^ | epidermal growth factor receptor signaling pathway | extracellular space |
| 205479_s_at | PLAU | plasminogen activator, urokinase | 0.86 | 113.5 | 132.0 | 3.8 x 10^-3^ | response to hypoxia | extracellular region |
| 209191_at | TUBB6 | tubulin, beta 6 | 1.12 | 142.5 | 127.8 | 4.0 x 10^-4^ | microtubule-based process | microtubule |
| 209921_at | SLC7A11 | solute carrier family 7, (cationic amino acid transporter, y+ system) member 11 | 1.10 | 138.5 | 125.8 | 1.6 x 10^-5^ | transport | membrane |
| 218145_at | TRIB3 | tribbles homolog 3 (Drosophila) | 1.51 | 188.3 | 124.9 | 6.0 x 10^-10^ | transcription | nucleus |
| 221870_at | EHD2 | EH-domain containing 2 | 0.27 | 33.1 | 123.7 | 1.1 x 10^-8^ | translation | intracellular |
| 205595_at | DSG3 | desmoglein 3 (pemphigus vulgaris antigen) | 1.07 | 128.4 | 120.1 | 3.0 x 10^-4^ | cell adhesion | cytosol |
| 202628_s_at | SERPINE1 | serpin peptidase inhibitor, clade E (nexin, plasminogen activator inhibitor type 1), member 1 | 0.40 | 46.8 | 116.5 | 7.7 x 10^-3^ | chronological cell aging | extracellular region |
| 201109_s_at | THBS1 | thrombospondin 1 | 0.54 | 59.4 | 110.4 | 7.3 x 10^-3^ | activation of MAPK activity | extracellular region |
| 212077_at | CALD1 | caldesmon 1 | 1.24 | 135.8 | 109.6 | 1.2 x 10^-6^ | cell motion | membrane fraction |
| 220620_at | CRCT1 | cysteine-rich C-terminal 1 | 0.17 | 16.9 | 97.42 | 7.0 x 10^-4^ |  |  |
| 201110_s_at | THBS1 | thrombospondin 1 | 0.71 | 68.6 | 96.41 | 7.3 x 10^-3^ | activation of MAPK activity | extracellular region |
| 201108_s_at | THBS1 | thrombospondin 1 | 0.62 | 56.9 | 91.96 | 1.0 x 10^-4^ | activation of MAPK activity | extracellular region |
| 201617_x_at | CALD1 | caldesmon 1 | 0.28 | 25.6 | 91.50 | 8.9 x 10^-7^ | cell motion | membrane fraction |
| 212190_at | SERPINE2 | serpin peptidase inhibitor, clade E (nexin, plasminogen activator inhibitor type 1), member 2 | 0.93 | 85.0 | 91.36 | 1.9 x 10^-9^ | multicellular organismal development | extracellular region |
| 214549_x_at | SPRR1A | small proline-rich protein 1A | 1.17 | 104.6 | 89.45 | 8.5 x 10^-6^ | epidermis development | cornified envelope |
| 201564_s_at | FSCN1 | fascin homolog 1, actin-bundling protein (Strongylocentrotus purpuratus) | 0.30 | 26.9 | 88.95 | 1.3 x 10^-5^ | cell proliferation | cytoplasm |
| 218309_at | CAMK2N1 | calcium/calmodulin-dependent protein kinase II inhibitor 1 | 0.60 | 51.0 | 85.39 | 3.0 x 10^-7^ |  | plasma membrane |
| 204602_at | DKK1 | dickkopf homolog 1 (Xenopus laevis) | 1.11 | 91.4 | 82.64 | 2.2 x 10^-3^ | multicellular organismal development | extracellular region |
| 210138_at | RGS20 | regulator of G-protein signaling 20 | 0.20 | 16.1 | 82.27 | 3.5 x 10^-7^ | regulation of G-protein coupled receptor protein signaling pathway | membrane |
| 231202_at | ALDH1L2 | Aldehyde dehydrogenase 1 family, member L2 | 0.30 | 24.3 | 79.86 | 2.1 x 10^-5^ | one-carbon compound metabolic process | cytoplasm |
| 212657_s_at | IL1RN | interleukin 1 receptor antagonist | 2.10 | 162.7 | 77.64 | 3.0 x 10^-4^ | inflammatory response | extracellular region |
| 231771_at | GJB6 | gap junction protein, beta 6, 30kDa | 0.25 | 18.7 | 75.34 | 8.9 x 10^-3^ | apoptosis | cytosol |
| 232056_at | SCEL | sciellin | 0.30 | 22.4 | 74.14 | 9.9 x 10^-6^ | epidermis development | cornified envelope |
| 239381_at | KLK7 | kallikrein-related peptidase 7 | 0.60 | 42.9 | 71.98 | 2.0 x 10^-6^ | proteolysis | extracellular region |
| 209900_s_at | SLC16A1 | solute carrier family 16, member 1 (monocarboxylic acid transporter 1) | 0.34 | 23.8 | 69.75 | 1.9 x 10^-7^ | transport | membrane fraction |
| 1552487_a_at | BNC1 | basonuclin 1 | 0.50 | 35.1 | 69.70 | 9.4 x 10^-5^ | transcription | intracellular |
| 211668_s_at | PLAU | plasminogen activator, urokinase | 0.75 | 50.8 | 67.96 | 5.5 x 10^-3^ | response to hypoxia | extracellular region |
| 1554921_a_at | SCEL | sciellin | 0.27 | 17.9 | 67.40 | 1.6 x 10^-6^ | epidermis development | cornified envelope |
| 202627_s_at | SERPINE1 | serpin peptidase inhibitor, clade E (nexin, plasminogen activator inhibitor type 1), member 1 | 0.74 | 48.6 | 65.74 | 1.5 x 10^-3^ | chronological cell aging | extracellular region |
| 219181_at | LIPG | lipase, endothelial | 0.25 | 16.0 | 62.80 | 3.0 x 10^-4^ | lipid metabolic process | extracellular region |
| 217996_at | PHLDA1 | pleckstrin homology-like domain, family A, member 1 | 1.84 | 112.3 | 61.15 | 1.8 x 10^-5^ | apoptosis | nucleus |
| 232082_x_at | SPRR3 | small proline-rich protein 3 | 0.80 | 48.2 | 60.07 | 5.0 x 10^-4^ | epidermis development | cytoplasm |
| 228302_x_at | CAMK2N1 | calcium/calmodulin-dependent protein kinase II inhibitor 1 | 0.07 | 4.0 | 57.66 | 6.1 x 10^-7^ |  | plasma membrane |
| 218717_s_at | LEPREL1 | leprecan-like 1 | 0.12 | 6.8 | 56.98 | 1.8 x 10^-3^ | protein metabolic process | endoplasmic reticulum |
| 223278_at | GJB2 | gap junction protein, beta 2, 26kDa | 3.26 | 183.1 | 56.24 | 4.0 x 10^-3^ | transport | Golgi membrane |
| 213680_at | KRT6B | keratin 6B | 2.06 | 114.8 | 55.71 | 2.5 x 10^-5^ | ectoderm development | intermediate filament |
| 223062_s_at | PSAT1 | phosphoserine aminotransferase 1 | 4.27 | 233.0 | 54.53 | 1.0 x 10^-8^ | L-serine biosynthetic process |  |
| 206343_s_at | NRG1 | neuregulin 1 | 0.37 | 19.8 | 53.38 | 7.4 x 10^-7^ | cell communication | extracellular region |
| 202234_s_at | SLC16A1 | solute carrier family 16, member 1 (monocarboxylic acid transporter 1) | 0.30 | 15.6 | 52.86 | 2.0 x 10^-8^ | transport | membrane fraction |
| 201616_s_at | CALD1 | caldesmon 1 | 0.51 | 26.6 | 52.52 | 4.1 x 10^-8^ | cell motion | membrane fraction |
| 201656_at | ITGA6 | integrin, alpha 6 | 2.56 | 129.8 | 50.77 | 5.9 x 10^-9^ | cell-substrate junction assembly | integrin complex |
| 205157_s_at | KRT17 | keratin 17 | 10.68 | 537.9 | 50.34 | 1.9 x 10^-8^ | epidermis development | nucleus |
| 200632_s_at | NDRG1 | N-myc downstream regulated 1 | 6.67 | 335.8 | 50.32 | 4.3 x 10^-9^ | response to metal ion | nucleus |
| 227806_at | C16orf74 | chromosome 16 open reading frame 74 | 0.26 | 12.5 | 48.27 | 1.3 x 10^-8^ |  |  |
| 206400_at | LGALS7 /// LGALS7B | lectin, galactoside-binding, soluble, 7 /// lectin, galactoside-binding, soluble, 7B | 5.82 | 280.8 | 48.26 | 1.0 x 10^-6^ | apoptosis | extracellular region |
| 216243_s_at | IL1RN | interleukin 1 receptor antagonist | 0.78 | 36.0 | 46.27 | 1.9 x 10^-3^ | inflammatory response | extracellular region |
| 220892_s_at | PSAT1 | phosphoserine aminotransferase 1 | 2.16 | 99.7 | 46.22 | 5.3 x 10^-6^ | L-serine biosynthetic process |  |
| 225481_at | FRMD6 | FERM domain containing 6 | 2.13 | 96.9 | 45.40 | 2.3 x 10^-7^ |  | cytoplasm |
| 224204_x_at | ARNTL2 | aryl hydrocarbon receptor nuclear translocator-like 2 | 0.85 | 38.1 | 44.89 | 9.2 x 10^-7^ | transcription | nucleus |
| 226847_at | FST | follistatin | 0.61 | 27.1 | 44.25 | 7.4 x 10^-5^ | negative regulation of transcription from RNA polymerase II promoter | extracellular region |
| 1558871_at | --- | --- | 0.10 | 4.3 | 43.62 | 1.5 x 10^-3^ |  |  |
| 210933_s_at | FSCN1 | fascin homolog 1, actin-bundling protein (Strongylocentrotus purpuratus) | 0.64 | 27.5 | 43.37 | 2.0 x 10^-4^ | cell proliferation | cytoplasm |
| 242809_at | IL1RL1 | Interleukin 1 receptor-like 1 | 0.52 | 22.1 | 42.73 | 2.0 x 10^-4^ | immune response | extracellular region |
| 218990_s_at | SPRR3 | small proline-rich protein 3 | 1.88 | 80.1 | 42.68 | 7.0 x 10^-4^ | epidermis development | cytoplasm |
| 230188_at | NIPAL4 | NIPA-like domain containing 4 | 0.17 | 7.1 | 42.47 | 1.0 x 10^-4^ |  | membrane |
| 203562_at | FEZ1 | fasciculation and elongation protein zeta 1 (zygin I) | 1.27 | 53.4 | 42.18 | 2.2 x 10^-6^ | cell adhesion | cytoplasm |
| 1557321_a_at | CAPN14 | calpain 14 | 0.17 | 7.2 | 42.02 | 5.8 x 10^-5^ | proteolysis | intracellular |
| 201761_at | MTHFD2 | methylenetetrahydrofolate dehydrogenase (NADP+ dependent) 2, methenyltetrahydrofolate cyclohydrolase | 4.08 | 171.2 | 41.93 | 8.4 x 10^-10^ | one-carbon compound metabolic process | mitochondrion |
| 202235_at | SLC16A1 | solute carrier family 16, member 1 (monocarboxylic acid transporter 1) | 0.18 | 7.7 | 41.89 | 4.4 x 10^-7^ | transport | membrane fraction |
| 209803_s_at | PHLDA2 | pleckstrin homology-like domain, family A, member 2 | 3.98 | 165.9 | 41.71 | 1.0 x 10^-7^ | apoptosis | cytoplasm |
| 210517_s_at | AKAP12 | A kinase (PRKA) anchor protein 12 | 1.55 | 64.7 | 41.62 | 1.8 x 10^-3^ | protein targeting | nucleus |
| 201105_at | LGALS1 | lectin, galactoside-binding, soluble, 1 | 8.48 | 343.3 | 40.47 | 8.9 x 10^-6^ | apoptosis | extracellular region |
| 218000_s_at | PHLDA1 | pleckstrin homology-like domain, family A, member 1 | 0.30 | 12.0 | 40.10 | 5.0 x 10^-4^ | apoptosis | nucleus |
| 225842_at | PHLDA1 | pleckstrin homology-like domain, family A, member 1 | 0.78 | 31.4 | 40.06 | 8.7 x 10^-8^ | apoptosis | nucleus |
| 1555758_a_at | CDKN3 | cyclin-dependent kinase inhibitor 3 | 0.43 | 17.0 | 39.70 | 4.8 x 10^-3^ | regulation of cyclin-dependent protein kinase activity | cytoplasm |
| 227529_s_at | AKAP12 | A kinase (PRKA) anchor protein 12 | 0.22 | 8.5 | 38.84 | 4.6 x 10^-3^ | protein targeting | nucleus |
| 218888_s_at | NETO2 | neuropilin (NRP) and tolloid (TLL)-like 2 | 0.72 | 27.6 | 38.41 | 1.4 x 10^-6^ |  | membrane |
| 204733_at | KLK6 | kallikrein-related peptidase 6 | 0.43 | 16.2 | 38.07 | 8.2 x 10^-5^ | proteolysis | extracellular region |
| 222486_s_at | ADAMTS1 | ADAM metallopeptidase with thrombospondin type 1 motif, 1 | 0.25 | 9.3 | 37.29 | 2.4 x 10^-6^ | ovulation from ovarian follicle | extracellular region |
| 217997_at | PHLDA1 | pleckstrin homology-like domain, family A, member 1 | 1.23 | 45.7 | 37.18 | 4.4 x 10^-3^ | apoptosis | nucleus |
| 234700_s_at | RNASE7 | ribonuclease, RNase A family, 7 | 0.19 | 7.2 | 37.17 | 1.5 x 10^-7^ | response to bacterium | extracellular region |
| 205691_at | SYNGR3 | synaptogyrin 3 | 0.18 | 6.4 | 36.33 | 8.8 x 10^-3^ |  | integral to plasma membrane |
| 201506_at | TGFBI | transforming growth factor, beta-induced, 68kDa | 6.75 | 243.7 | 36.09 | 2.0 x 10^-4^ | cell adhesion | extracellular region |
| 227530_at | AKAP12 | A kinase (PRKA) anchor protein 12 | 0.70 | 25.0 | 35.81 | 7.2 x 10^-3^ | protein targeting | nucleus |
| 222774_s_at | NETO2 | neuropilin (NRP) and tolloid (TLL)-like 2 | 0.50 | 17.8 | 35.68 | 5.7 x 10^-6^ |  | membrane |
| 209260_at | SFN | stratifin | 3.99 | 141.0 | 35.38 | 2.0 x 10^-4^ | regulation of cyclin-dependent protein kinase activity | extracellular region |
| 212816_s_at | CBS | cystathionine-beta-synthase | 0.33 | 11.5 | 35.25 | 1.3 x 10^-7^ | cysteine metabolic process | nucleus |
| 208539_x_at | SPRR2B | small proline-rich protein 2B | 0.86 | 30.4 | 35.25 | 3.0 x 10^-4^ | epidermis development | cornified envelope |
| 202267_at | LAMC2 | laminin, gamma 2 | 11.85 | 417.2 | 35.22 | 1.6 x 10^-6^ | cell adhesion | extracellular region |
| 205047_s_at | ASNS | asparagine synthetase | 5.91 | 207.4 | 35.13 | 3.7 x 10^-8^ | asparagine biosynthetic process | soluble fraction |
| 226817_at | DSC2 | desmocollin 2 | 1.12 | 39.3 | 35.10 | 2.2 x 10^-5^ | cell adhesion | plasma membrane |
| 204948_s_at | FST | follistatin | 1.19 | 41.2 | 34.56 | 8.8 x 10^-5^ | negative regulation of transcription from RNA polymerase II promoter | extracellular region |
| 205234_at | SLC16A4 | solute carrier family 16, member 4 (monocarboxylic acid transporter 5) | 0.53 | 18.4 | 34.47 | 2.4 x 10^-6^ | transport | membrane fraction |
| 225688_s_at | PHLDB2 | pleckstrin homology-like domain, family B, member 2 | 2.51 | 85.0 | 33.87 | 1.0 x 10^-8^ |  | cytoplasm |
| 230973_at | SH2D5 | SH2 domain containing 5 | 0.31 | 10.3 | 33.32 | 2.0 x 10^-4^ |  |  |
| 204214_s_at | RAB32 | RAB32, member RAS oncogene family | 2.45 | 81.4 | 33.23 | 9.6 x 10^-7^ | small GTPase mediated signal transduction | mitochondrion |
| 213848_at | --- | --- | 2.15 | 71.3 | 33.13 | 3.7 x 10^-8^ |  |  |
| 203650_at | PROCR | protein C receptor, endothelial (EPCR) | 0.79 | 26.1 | 33.06 | 2.7 x 10^-7^ | immune response | centrosome |
| 222646_s_at | ERO1L | ERO1-like (S. cerevisiae) | 4.99 | 164.2 | 32.91 | 8.4 x 10^-5^ | protein folding | membrane fraction |
| 201667_at | GJA1 | gap junction protein, alpha 1, 43kDa | 2.55 | 83.8 | 32.86 | 5.0 x 10^-4^ | transport | plasma membrane |
| 237690_at | GPR115 | G protein-coupled receptor 115 | 0.29 | 9.5 | 32.32 | 4.4 x 10^-3^ | signal transduction | plasma membrane |
| 1559393_at | ALDH1L2 | aldehyde dehydrogenase 1 family, member L2 | 0.10 | 3.3 | 32.31 | 9.0 x 10^-6^ | one-carbon compound metabolic process | cytoplasm |
| 210355_at | PTHLH | parathyroid hormone-like hormone | 0.58 | 18.6 | 32.26 | 3.0 x 10^-4^ | skeletal system development | extracellular region |
| 222449_at | PMEPA1 | prostate transmembrane protein, androgen induced 1 | 1.79 | 57.6 | 32.15 | 4.6 x 10^-6^ | androgen receptor signaling pathway | plasma membrane |
| 215177_s_at | ITGA6 | integrin, alpha 6 | 2.83 | 90.2 | 31.83 | 2.7 x 10^-6^ | cell-substrate junction assembly | integrin complex |
| 202236_s_at | SLC16A1 | solute carrier family 16, member 1 (monocarboxylic acid transporter 1) | 1.12 | 35.6 | 31.71 | 2.6 x 10^-6^ | transport | membrane fraction |
| 1564630_at | EDN1 | endothelin 1 | 0.24 | 7.5 | 31.69 | 5.2 x 10^-5^ | patterning of blood vessels | extracellular region |
| 203887_s_at | THBD | thrombomodulin | 1.70 | 53.5 | 31.48 | 3.1 x 10^-3^ | female pregnancy | extracellular space |
| 214967_at | --- | --- | 0.10 | 3.2 | 31.21 | 3.4 x 10^-6^ |  |  |
| 225464_at | FRMD6 | FERM domain containing 6 | 3.14 | 97.6 | 31.10 | 3.3 x 10^-7^ |  | cytoplasm |
| 212647_at | RRAS | related RAS viral (r-ras) oncogene homolog | 1.93 | 59.5 | 30.84 | 1.5 x 10^-7^ | small GTPase mediated signal transduction | intracellular |
| 204348_s_at | AK3L1 | adenylate kinase 3-like 1 | 0.83 | 25.5 | 30.75 | 3.2 x 10^-8^ | nucleobase, nucleoside, nucleotide and nucleic acid metabolic process | mitochondrion |
| 204420_at | FOSL1 | FOS-like antigen 1 | 0.75 | 22.9 | 30.68 | 6.0 x 10^-4^ | regulation of transcription, DNA-dependent | nucleus |
| 205289_at | BMP2 | bone morphogenetic protein 2 | 0.27 | 8.2 | 30.66 | 1.2 x 10^-9^ | skeletal system development | extracellular region |
| 234976_x_at | SLC4A5 | Solute carrier family 4, sodium bicarbonate cotransporter, member 5 | 0.13 | 4.1 | 30.52 | 5.9 x 10^-11^ | transport | plasma membrane |
| 216080_s_at | FADS3 | fatty acid desaturase 3 | 0.60 | 18.1 | 30.29 | 2.5 x 10^-6^ | lipid metabolic process | membrane fraction |
| 216918_s_at | DST | dystonin | 3.29 | 99.3 | 30.22 | 2.0 x 10^-8^ | mesoderm formation | basement membrane |
| 210015_s_at | MAP2 | microtubule-associated protein 2 | 0.43 | 13.0 | 29.96 | 9.6 x 10^-5^ | microtubule bundle formation | cytoplasm |
| 220658_s_at | ARNTL2 | aryl hydrocarbon receptor nuclear translocator-like 2 | 1.36 | 40.6 | 29.94 | 4.9 x 10^-8^ | transcription | nucleus |
| 200924_s_at | SLC3A2 | solute carrier family 3 (activators of dibasic and neutral amino acid transport), member 2 | 2.91 | 84.6 | 29.12 | 2.4 x 10^-8^ | carbohydrate metabolic process | plasma membrane |
| 233737_s_at | LOC284561 | hypothetical protein LOC284561 | 0.19 | 5.4 | 28.99 | 2.1 x 10^-5^ |  |  |
| 206100_at | CPM | carboxypeptidase M | 0.42 | 12.2 | 28.78 | 1.0 x 10^-4^ | proteolysis | plasma membrane |
| 228575_at | IL20RB | interleukin 20 receptor beta | 2.35 | 67.3 | 28.70 | 4.7 x 10^-11^ |  | membrane |
| 222450_at | PMEPA1 | prostate transmembrane protein, androgen induced 1 | 1.69 | 48.3 | 28.66 | 3.5 x 10^-7^ | androgen receptor signaling pathway | plasma membrane |
| 229622_at | FAM132B | family with sequence similarity 132, member B | 0.30 | 8.6 | 28.49 | 2.0 x 10^-5^ |  | extracellular region |
| 215495_s_at | SAMD4A | sterile alpha motif domain containing 4A | 0.15 | 4.3 | 28.30 | 1.9 x 10^-5^ | positive regulation of translation | synaptosome |
| 202912_at | ADM | adrenomedullin | 1.89 | 53.3 | 28.26 | 1.9 x 10^-10^ | cAMP biosynthetic process | extracellular region |
| 213524_s_at | G0S2 | G0/G1switch 2 | 3.52 | 99.0 | 28.10 | 2.0 x 10^-4^ | cell cycle |  |
| 238542_at | ULBP2 | UL16 binding protein 2 | 0.51 | 14.4 | 28.04 | 1.3 x 10^-6^ | immune response | extracellular region |
| 1561633_at | HMGA2 | high mobility group AT-hook 2 | 0.10 | 2.8 | 27.92 | 6.0 x 10^-10^ | establishment or maintenance of chromatin architecture | nuclear chromosome |
| 230508_at | DKK3 | dickkopf homolog 3 (Xenopus laevis) | 0.26 | 7.3 | 27.91 | 2.5 x 10^-6^ | multicellular organismal  development | extracellular region |
| 205421_at | SLC22A3 | solute carrier family 22 (extraneuronal monoamine transporter), member 3 | 0.09 | 2.5 | 27.62 | 2.0 x 10^-4^ | transport | membrane fraction |
| 203726_s_at | LAMA3 | laminin, alpha 3 | 5.39 | 148.4 | 27.55 | 1.3 x 10^-5^ | cell adhesion | extracellular region |
| 202437_s_at | CYP1B1 | cytochrome P450, family 1, subfamily B, polypeptide 1 | 0.74 | 20.4 | 27.38 | 5.9 x 10^-3^ | cellular aromatic compound metabolic process | endoplasmic reticulum |
| 201389_at | ITGA5 | integrin, alpha 5 (fibronectin receptor, alpha polypeptide) | 0.67 | 18.3 | 27.28 | 2.9 x 10^-6^ | cell-substrate junction assembly | ruffle |
| 232263_at | SLC6A15 | solute carrier family 6 (neutral amino acid transporter), member 15 | 0.28 | 7.5 | 27.22 | 6.8 x 10^-7^ | transport | integral to plasmamembrane |
| 202998_s_at | LOXL2 | lysyl oxidase-like 2 | 0.32 | 8.6 | 27.09 | 1.8 x 10^-3^ | protein modification process | extracellular region |
| 207528_s_at | SLC7A11 | solute carrier family 7, (cationic amino acid transporter, y+ system) member 11 | 0.42 | 11.2 | 26.87 | 2.4 x 10^-6^ | transport | membrane |
| 204750_s_at | DSC2 | desmocollin 2 | 0.16 | 4.2 | 26.24 | 2.0 x 10^-4^ | cell adhesion | plasma membrane |
| 200951_s_at | CCND2 | cyclin D2 | 0.48 | 12.4 | 26.15 | 2.5 x 10^-7^ | G1 | cyclin-dependent protein kinase holoenzyme complex |
| 239370_at | --- | --- | 2.46 | 62.9 | 25.58 | 2.0 x 10^-4^ |  |  |
| 218368_s_at | TNFRSF12A | tumor necrosis factor receptor superfamily, member 12A | 2.72 | 69.4 | 25.50 | 1.3 x 10^-5^ | angiogenesis | ruffle |
| 213421_x_at | PRSS3 | protease, serine, 3 | 0.31 | 7.9 | 25.46 | 5.3 x 10^-7^ | proteolysis | extracellular region |
| 202558_s_at | HSPA13 | heat shock protein 70kDa family, member 13 | 3.01 | 76.5 | 25.41 | 2.0 x 10^-4^ |  | endoplasmic reticulum |
| 203592_s_at | FSTL3 | follistatin-like 3 (secreted glycoprotein) | 0.65 | 16.4 | 25.40 | 1.1 x 10^-6^ | negative regulation of BMP signaling pathway | extracellular region |
| 203439_s_at | STC2 | stanniocalcin 2 | 0.53 | 13.5 | 25.40 | 2.1 x 10^-8^ | cell surface receptor linked signal transduction | extracellular region |
| 219270_at | CHAC1 | ChaC, cation transport regulator homolog 1 (E. coli) | 0.87 | 21.9 | 25.22 | 1.7 x 10^-5^ |  |  |
| 237252_at | THBD | thrombomodulin | 0.27 | 6.7 | 25.20 | 1.0 x 10^-4^ | female pregnancy | extracellular space |
| 214095_at | SHMT2 | serine hydroxymethyltransferase 2 (mitochondrial) | 0.60 | 15.0 | 24.86 | 3.1 x 10^-12^ | glycine metabolic process | mitochondrion |
| 228653_at | SAMD5 | sterile alpha motif domain containing 5 | 0.27 | 6.6 | 24.71 | 2.8 x 10^-3^ |  |  |
| 212012_at | PXDN | peroxidasin homolog (Drosophila) | 1.16 | 28.5 | 24.65 | 1.4 x 10^-10^ | immune response | extracellular region |
| 225459_at | AMOTL1 | angiomotin like 1 | 0.24 | 5.8 | 24.53 | 5.0 x 10^-6^ |  | cytoplasm |
| 229900_at | CD109 | CD109 molecule | 0.68 | 16.7 | 24.43 | 2.6 x 10^-5^ |  | extracellular region |
| 201147_s_at | TIMP3 | TIMP metallopeptidase inhibitor 3 | 0.90 | 21.7 | 24.08 | 2.0 x 10^-4^ | transmembrane receptor protein tyrosine kinase signaling pathway | extracellular region |
| 204686_at | IRS1 | insulin receptor substrate 1 | 1.79 | 43.2 | 24.07 | 1.2 x 10^-5^ | positive regulation of mesenchymal cell proliferation | nucleus |
| 212013_at | PXDN | peroxidasin homolog (Drosophila) | 0.40 | 9.5 | 24.02 | 2.2 x 10^-8^ | immune response | extracellular region |
| 204455_at | DST | dystonin | 10.09 | 240.7 | 23.87 | 7.4 x 10^-8^ | mesoderm formation | basement membrane |
| 206008_at | TGM1 | transglutaminase 1 (K polypeptide epidermal type I, protein-glutamine-gamma-glutamyltransferase) | 0.47 | 11.1 | 23.82 | 1.0 x 10^-4^ | protein modification process | cornified envelope |
| 226545_at | CD109 | CD109 molecule | 1.76 | 41.8 | 23.70 | 1.0 x 10^-4^ |  | extracellular region |
| 243818_at | SFTA1P | surfactant associated 1 (pseudogene) | 0.19 | 4.5 | 23.47 | 1.3 x 10^-5^ |  |  |
| 202436_s_at | CYP1B1 | cytochrome P450, family 1, subfamily B, polypeptide 1 | 1.35 | 31.7 | 23.46 | 9.5 x 10^-3^ | cellular aromatic compound metabolic process | endoplasmic reticulum |
| 200831_s_at | SCD | stearoyl-CoA desaturase (delta-9-desaturase) | 3.35 | 78.5 | 23.39 | 2.2 x 10^-3^ | lipid metabolic process | endoplasmic reticulum |
| 239823_at | --- | --- | 0.10 | 2.3 | 23.38 | 7.8 x 10^-5^ |  |  |
| 210869_s_at | MCAM | melanoma cell adhesion molecule | 0.64 | 14.8 | 23.27 | 9.2 x 10^-3^ | cell adhesion | nucleus |
| 207463_x_at | PRSS3 | protease, serine, 3 | 0.31 | 7.1 | 23.18 | 8.2 x 10^-8^ | proteolysis | extracellular region |
| 204256_at | ELOVL6 | ELOVL family member 6, elongation of long chain fatty acids (FEN1/Elo2, SUR4/Elo3-like, yeast) | 1.31 | 30.3 | 23.17 | 7.7 x 10^-5^ | fatty acid biosynthetic process | mitochondrion |
| 212236_x_at | KRT17 | keratin 17 | 22.25 | 514.8 | 23.14 | 1.9 x 10^-8^ | epidermis development | nucleus |
| 234066_at | IL1RL1 | Interleukin 1 receptor-like 1 | 0.45 | 10.5 | 23.12 | 1.7 x 10^-5^ | immune response | extracellular region |
| 225540_at | MAP2 | microtubule-associated protein 2 | 1.64 | 37.8 | 22.97 | 3.0 x 10^-7^ | microtubule bundle formation | cytoplasm |
| 239825_at | --- | --- | 0.32 | 7.2 | 22.74 | 2.8 x 10^-3^ |  |  |
| 1567107_s_at | TPM4 | tropomyosin 4 | 1.07 | 24.3 | 22.73 | 1.1 x 10^-6^ | cell motion | cytoplasm |
| 214599_at | IVL | involucrin | 0.24 | 5.4 | 22.66 | 2.8 x 10^-3^ | response to UV-B | cornified envelope |
| 229163_at | CAMK2N1 | calcium/calmodulin-dependent protein kinase II inhibitor 1 | 0.17 | 3.8 | 22.60 | 2.3 x 10^-5^ |  | plasma membrane |
| 231094_s_at | MTHFD1L | methylenetetrahydrofolate dehydrogenase (NADP+ dependent) 1-like | 0.45 | 10.2 | 22.52 | 1.9 x 10^-6^ | one-carbon compound metabolic process | mitochondrion |
| 209714_s_at | CDKN3 | cyclin-dependent kinase inhibitor 3 | 0.71 | 15.9 | 22.49 | 5.9 x 10^-3^ | regulation of cyclin dependent protein kinase activity | cytoplasm |
| 206376_at | SLC6A15 | solute carrier family 6 (neutral amino acid transporter), member 15 | 0.68 | 15.3 | 22.36 | 5.5 x 10^-9^ | transport | integral to plasma membrane |
| 204614_at | SERPINB2 | serpin peptidase inhibitor, clade B (ovalbumin), member 2 | 4.69 | 104.2 | 22.24 | 1.0 x 10^-4^ | anti-apoptosis | extracellular region |
| 207030_s_at | CSRP2 | cysteine and glycine-rich protein 2 | 2.34 | 51.6 | 22.06 | 7.4 x 10^-7^ | multicellular organismal development | nucleus |
| 221539_at | EIF4EBP1 | eukaryotic translation initiation factor 4E binding protein 1 | 2.84 | 62.7 | 22.05 | 1.3 x 10^-10^ | regulation of translation | cytosol |
| 209278_s_at | TFPI2 | tissue factor pathway inhibitor 2 | 1.91 | 41.9 | 21.94 | 6.5 x 10^-3^ | blood coagulation | extracellular region |
| 227949_at | PHACTR3 | phosphatase and actin regulator 3 | 0.63 | 13.8 | 21.76 | 7.3 x 10^-5^ |  | nucleus |
| 210237_at | ARTN | artemin | 0.41 | 9.0 | 21.73 | 3.0 x 10^-4^ | signal transduction | extracellular region |
| 204347_at | AK3L1 | Adenylate kinase 3-like 1 | 0.47 | 10.1 | 21.69 | 3.7 x 10^-8^ | nucleobase, nucleoside, nucleotide and nucleic acid metabolic process | mitochondrion |
| 212659_s_at | IL1RN | interleukin 1 receptor antagonist | 1.05 | 22.8 | 21.64 | 1.3 x 10^-3^ | inflammatory response | extracellular region |
| 209086_x_at | MCAM | melanoma cell adhesion molecule | 0.41 | 8.8 | 21.46 | 2.1 x 10^-3^ | cell adhesion | nucleus |
| 222945_x_at | OLAH | oleoyl-ACP hydrolase | 0.52 | 11.0 | 21.19 | 9.6 x 10^-3^ | fatty acid biosynthetic process |  |
| 200832_s_at | SCD | stearoyl-CoA desaturase (delta-9-desaturase) | 10.57 | 223.8 | 21.17 | 6.8 x 10^-5^ | lipid metabolic process | endoplasmic reticulum |
| 214595_at | KCNG1 | potassium voltage-gated channel, subfamily G, member 1 | 0.34 | 7.3 | 21.15 | 1.7 x 10^-7^ | transport | voltage-gated potassium channel complex |
| 219554_at | RHCG | Rh family, C glycoprotein | 0.26 | 5.4 | 21.09 | 6.0 x 10^-4^ | transport | plasma membrane |
| 223533_at | LRRC8C | leucine rich repeat containing 8 family, member C | 0.68 | 14.3 | 21.05 | 7.9 x 10^-6^ |  | membrane |
| 225520_at | MTHFD1L | methylenetetrahydrofolate dehydrogenase (NADP+ dependent) 1-like | 2.33 | 48.9 | 21.03 | 1.8 x 10^-9^ | one-carbon compound metabolic process | mitochondrion |
| 203695_s_at | DFNA5 | deafness, autosomal dominant 5 | 3.55 | 74.2 | 20.92 | 1.1 x 10^-9^ | sensory perception of sound |  |
| 238151_at | --- | --- | 0.12 | 2.5 | 20.92 | 3.4 x 10^-7^ |  |  |
| 228314_at | LRRC8C | leucine rich repeat containing 8 family, member C | 0.79 | 16.4 | 20.90 | 4.0 x 10^-4^ |  | membrane |
| 233413_at | --- | --- | 0.14 | 2.9 | 20.84 | 1.0 x 10^-3^ |  |  |
| 212110_at | SLC39A14 | solute carrier family 39 (zinc transporter), member 14 | 2.22 | 46.2 | 20.83 | 1.9 x 10^-9^ | transport | plasma membrane |
| 233487_s_at | LRRC8A | leucine rich repeat containing 8 family, member A | 2.10 | 43.7 | 20.79 | 9.9 x 10^-12^ |  | membrane |
| 204952_at | LYPD3 | LY6/PLAUR domain containing 3 | 0.69 | 14.4 | 20.77 | 2.6 x 10^-3^ | cell motion | plasma membrane |
| 55081_at | MICALL1 | MICAL-like 1 | 4.18 | 86.7 | 20.76 | 1.3 x 10^-6^ |  | cytoplasm |
| 1556841_a_at | ALDH1L2 | aldehyde dehydrogenase 1 family, member L2 | 0.12 | 2.5 | 20.52 | 6.0 x 10^-8^ | one-carbon compound metabolic process | cytoplasm |
| 204165_at | WASF1 | WAS protein family, member 1 | 0.59 | 12.1 | 20.47 | 2.4 x 10^-7^ | protein complex assembly | cytoplasm |
| 219058_x_at | TINAGL1 | tubulointerstitial nephritis antigen-like 1 | 0.34 | 7.1 | 20.47 | 1.0 x 10^-4^ | proteolysis | extracellular region |
| 217999_s_at | PHLDA1 | pleckstrin homology-like domain, family A, member 1 | 0.50 | 10.1 | 20.37 | 3.0 x 10^-7^ | apoptosis | nucleus |
| 1557285_at | AREGB | Amphiregulin B | 0.54 | 10.9 | 20.37 | 2.0 x 10^-6^ |  |  |
| 208962_s_at | FADS1 | fatty acid desaturase 1 | 1.06 | 21.5 | 20.34 | 1.0 x 10^-3^ | lipid metabolic process | membrane fraction |
| 214793_at | DUSP7 | dual specificity phosphatase 7 | 0.24 | 4.8 | 20.22 | 3.1 x 10^-7^ | protein amino acid dephosphorylation | nucleoplasm |
| 217466_x_at | RPS2 | ribosomal protein S2 | 14.20 | 286.8 | 20.19 | 8.4 x 10^-9^ | translation | intracellular |
| 228840_at | AMOTL1 | angiomotin like 1 | 0.23 | 4.7 | 20.10 | 7.2 x 10^-5^ |  | cytoplasm |
| 204855_at | SERPINB5 | serpin peptidase inhibitor, clade B (ovalbumin), member 5 | 10.43 | 209.5 | 20.09 | 1.0 x 10^-4^ | cell motion | extracellular region |
| 235392_at | IRS1 | Insulin receptor substrate 1 | 0.32 | 6.3 | 20.08 | 6.0 x 10^-4^ | positive regulation of mesenchymal cell proliferation | nucleus |
| 209369_at | ANXA3 | annexin A3 | 11.62 | 233.2 | 20.07 | 3.3 x 10^-6^ | signal transduction | cytoplasm |
| 239853_at | KLC3 | kinesin light chain 3 | 0.45 | 8.9 | 19.88 | 4.2 x 10^-8^ |  | cytoplasm |
| 1555673_at | KRTAP2-4 | keratin associated protein 2-4 | 0.22 | 4.4 | 19.76 | 2.2 x 10^-6^ |  | intermediate filament |
| 225065_x_at | C17orf45 | chromosome 17 open reading frame 45 | 5.28 | 103.9 | 19.69 | 2.1 x 10^-7^ |  | mitochondrion |
| 228698_at | SOX7 | SRY (sex determining region Y)-box 7 | 2.12 | 41.7 | 19.68 | 7.4 x 10^-5^ | transcription | nucleus |
| 1553972_a_at | CBS | cystathionine-beta-synthase | 0.91 | 17.9 | 19.67 | 1.5 x 10^-7^ | cysteine metabolic process | nucleus |
| 214437_s_at | SHMT2 | serine hydroxymethyltransferase 2 (mitochondrial) | 2.12 | 41.6 | 19.57 | 2.5 x 10^-8^ | glycine metabolic process | mitochondrion |
| 219532_at | ELOVL4 | elongation of very long chain fatty acids (FEN1/Elo2, SUR4/Elo3, yeast)-like 4 | 0.30 | 6.0 | 19.56 | 8.8 x 10^-10^ | fatty acid biosynthetic process | endoplasmic reticulum |
| 222242_s_at | KLK5 | kallikrein-related peptidase 5 | 0.51 | 9.8 | 19.35 | 3.0 x 10^-4^ | proteolysis | extracellular region |
| 33323_r_at | SFN | stratifin | 31.80 | 609.6 | 19.17 | 5.4 x 10^-8^ | regulation of cyclin-dependent protein kinase activity | extracellular region |
| 235086_at | THBS1 | Thrombospondin 1 | 0.16 | 3.1 | 19.09 | 2.0 x 10^-4^ | activation of MAPK activity | extracellular region |
| 213746_s_at | FLNA | filamin A, alpha (actin binding protein 280) | 1.99 | 38.0 | 19.08 | 2.1 x 10^-5^ | inhibition of adenylate cyclase activity by dopamine receptor signaling pathway | nucleus |
| 202218_s_at | FADS2 | fatty acid desaturase 2 | 0.34 | 6.4 | 18.90 | 4.2 x 10^-3^ | lipid metabolic process | membrane fraction |
| 214866_at | PLAUR | plasminogen activator, urokinase receptor | 0.81 | 15.4 | 18.89 | 5.9 x 10^-3^ | cell motion | extracellular region |
| 203967_at | CDC6 | cell division cycle 6 homolog (S. cerevisiae) | 0.23 | 4.4 | 18.77 | 7.8 x 10^-3^ | DNA replication checkpoint | nucleus |
| 204058_at | ME1 | malic enzyme 1, NADP(+)-dependent, cytosolic | 2.38 | 44.6 | 18.76 | 3.3 x 10^-6^ | carbohydrate metabolic process | nucleus |
| 209270_at | LAMB3 | laminin, beta 3 | 15.86 | 297.3 | 18.74 | 2.8 x 10^-9^ | cell adhesion | extracellular region |
| 45297_at | EHD2 | EH-domain containing 2 | 1.29 | 24.2 | 18.69 | 3.5 x 10^-9^ | translation | intracellular |
| 214240_at | GAL | galanin prepropeptide | 0.27 | 5.1 | 18.62 | 2.1 x 10^-8^ | smooth muscle contraction | extracellular region |
| 217127_at | CTH | cystathionase (cystathionine gamma-lyase) | 2.19 | 40.6 | 18.51 | 7.3 x 10^-6^ | amino acid metabolic process | cytoplasm |
| 239430_at | IGFL1 | IGF-like family member 1 | 0.15 | 2.7 | 18.46 | 1.0 x 10^-4^ |  | extracellular region |
| 205827_at | CCK | cholecystokinin | 0.15 | 2.8 | 18.39 | 3.0 x 10^-4^ | neuron migration | extracellular region |
| 230102_at | ETV5 | Ets variant 5 | 0.33 | 6.0 | 18.34 | 5.5 x 10^-8^ | regulation of transcription, DNA-dependent | nucleus |
| 203888_at | THBD | thrombomodulin | 1.27 | 23.2 | 18.21 | 6.9 x 10^-3^ | female pregnancy | extracellular space |
| 211708_s_at | SCD | stearoyl-CoA desaturase (delta-9-desaturase) | 1.03 | 18.7 | 18.16 | 2.0 x 10^-3^ | lipid metabolic process | endoplasmic reticulum |
| 238460_at | FAM83A | family with sequence similarity 83, member A | 0.81 | 14.7 | 18.15 | 7.3 x 10^-5^ |  |  |
| 1555862_s_at | MICALL2 | MICAL-like 2 | 0.23 | 4.1 | 18.12 | 5.3 x 10^-7^ |  | cytoplasm |
| 201324_at | EMP1 | epithelial membrane protein 1 | 5.41 | 97.9 | 18.10 | 2.5 x 10^-5^ | multicellular organismal development | membrane fraction |
| 228745_at | SGTB | small glutamine-rich tetratricopeptide repeat (TPR)-containing, beta | 1.55 | 28.1 | 18.08 | 1.4 x 10^-5^ |  |  |
| 214247_s_at | DKK3 | dickkopf homolog 3 (Xenopus laevis) | 5.56 | 100.4 | 18.07 | 1.0 x 10^-3^ | multicellular organismal development | extracellular region |
| 224469_s_at | INF2 | inverted formin, FH2 and WH2 domain containing | 0.28 | 5.0 | 18.07 | 1.5 x 10^-3^ | cellular component organization | nucleus |
| 33322_i_at | SFN | stratifin | 31.93 | 575.5 | 18.02 | 2.0 x 10^-8^ | regulation of cyclin-dependent protein kinase activity | extracellular region |
| 227211_at | PHF19 | PHD finger protein 19 | 0.31 | 5.6 | 17.96 | 9.6 x 10^-5^ | transcription | nucleus |
| 203324_s_at | CAV2 | caveolin 2 | 10.27 | 183.9 | 17.91 | 9.9 x 10^-11^ | neuron migration | Golgi membrane |
| 209652_s_at | PGF | placental growth factor | 0.41 | 7.3 | 17.86 | 7.2 x 10^-7^ | angiogenesis | extracellular region |
| 202575_at | CRABP2 | cellular retinoic acid binding protein 2 | 2.04 | 36.4 | 17.78 | 1.9 x 10^-4^ | regulation of transcription, DNA-dependent | nucleus |
| 228274_at | SDSL | serine dehydratase-like | 0.16 | 2.9 | 17.77 | 2.4 x 10^-5^ | amino acid metabolic process |  |
| 206204_at | GRB14 | growth factor receptor-bound protein 14 | 0.09 | 1.7 | 17.74 | 1.3 x 10^-5^ | signal transduction | Golgi membrane |
| 201249_at | SLC2A1 | solute carrier family 2 (facilitated glucose transporter), member 1 | 0.31 | 5.5 | 17.67 | 3.9 x 10^-7^ | transport | intracellular |
| 220638_s_at | CBLC | Cas-Br-M (murine) ecotropic retroviral transforming sequence c | 0.58 | 10.3 | 17.54 | 3.6 x 10^-6^ | protein amino acid phosphorylation | nucleus |
| 201150_s_at | TIMP3 | TIMP metallopeptidase inhibitor 3 | 1.87 | 32.8 | 17.50 | 3.7 x 10^-4^ | transmembrane receptor protein tyrosine kinase signaling pathway | extracellular region |
| 223839_s_at | SCD | stearoyl-CoA desaturase (delta-9-desaturase) | 2.30 | 40.0 | 17.44 | 9.3 x 10^-6^ | lipid metabolic process | endoplasmic reticulum |
| 204334_at | KLF7 | Kruppel-like factor 7 (ubiquitous) | 1.97 | 34.1 | 17.32 | 2.4 x 10^-7^ | transcription | intracellular |
| 201505_at | LAMB1 | laminin, beta 1 | 1.81 | 31.3 | 17.30 | 2.6 x 10^-5^ | cell adhesion | extracellular region |
| 1569886_a_at | GLB1L3 | galactosidase, beta 1-like 3 | 0.37 | 6.5 | 17.29 | 1.3 x 10^-4^ | carbohydrate metabolic process | beta-galactosidase complex |
| 38037_at | HBEGF | heparin-binding EGF-like growth factor | 3.78 | 65.4 | 17.28 | 6.0 x 10^-7^ | signal transduction | extracellular region |
| 226517_at | BCAT1 | branched chain aminotransferase 1, cytosolic | 0.57 | 9.8 | 17.25 | 8.7 x 10^-5^ | G1 | cytoplasm |
| 224657_at | ERRFI1 | ERBB receptor feedback inhibitor 1 | 16.25 | 280.0 | 17.23 | 4.4 x 10^-11^ | response to stress | cytoplasm |
| 204693_at | CDC42EP1 | CDC42 effector protein (Rho GTPase binding) 1 | 0.44 | 7.5 | 17.18 | 1.7 x 10^-4^ | Rho protein signal transduction | cytoplasm |
| 224995_at | SPIRE1 | spire homolog 1 (Drosophila) | 4.36 | 74.7 | 17.12 | 1.2 x 10^-4^ | transport | cytoplasm |
| 206085_s_at | CTH | cystathionase (cystathionine gamma-lyase) | 0.87 | 14.9 | 17.08 | 7.9 x 10^-6^ | amino acid metabolic process | cytoplasm |
| 1554594_at | SH3D20 | SH3 domain containing 20 | 0.34 | 5.7 | 17.06 | 1.2 x 10^-7^ | endocytosis | intracellular |
| 216052_x_at | ARTN | artemin | 0.40 | 6.8 | 17.04 | 9.2 x 10^-5^ | signal transduction | extracellular region |
| 232689_at | LOC284561 | hypothetical protein LOC284561 | 0.34 | 5.8 | 17.01 | 5.5 x 10^-5^ |  |  |
| 60474_at | FERMT1 | fermitin family homolog 1 (Drosophila) | 3.65 | 62.0 | 16.98 | 2.0 x 10^-4^ | cell adhesion | cytoplasm |
| 211160_x_at | ACTN1 | actinin, alpha 1 | 4.17 | 70.8 | 16.97 | 5.8 x 10^-3^ | regulation of apoptosis | nucleolus |
| 209365_s_at | ECM1 | extracellular matrix protein 1 | 1.08 | 18.4 | 16.96 | 2.7 x 10^-5^ | positive regulation of I-kappaB kinase | extracellular region |
| 238419_at | PHLDB2 | pleckstrin homology-like domain, family B, member 2 | 0.24 | 4.1 | 16.95 | 7.3 x 10^-4^ |  | cytoplasm |
| 204268_at | S100A2 | S100 calcium binding protein A2 | 39.11 | 662.8 | 16.95 | 1.3 x 10^-7^ | endothelial cell migration |  |
| 206032_at | DSC3 | desmocollin 3 | 2.69 | 45.5 | 16.92 | 1.1 x 10^-8^ | cell adhesion | membrane fraction |
| 223586_at | ARNTL2 | aryl hydrocarbon receptor nuclear translocator-like 2 | 1.17 | 19.7 | 16.86 | 8.1 x 10^-10^ | transcription | nucleus |
| 203821_at | HBEGF | heparin-binding EGF-like growth factor | 6.70 | 112.9 | 16.85 | 6.8 x 10^-6^ | signal transduction | extracellular region |
| 1558045_a_at | LOC441528 | hypothetical protein LOC441528 | 0.22 | 3.7 | 16.84 | 2.5 x 10^-4^ |  |  |
| 225342_at | AK3L1 | adenylate kinase 3-like 1 | 1.61 | 27.1 | 16.82 | 1.9 x 10^-8^ | nucleobase, nucleoside, nucleotide and nucleic acid metabolic process | mitochondrion |
| 206461_x_at | MT1H | metallothionein 1H | 17.94 | 301.4 | 16.80 | 8.7 x 10^-5^ |  |  |
| 204647_at | HOMER3 | homer homolog 3 (Drosophila) | 1.07 | 17.9 | 16.77 | 4.0 x 10^-11^ | protein targeting | cytoplasm |
| 219654_at | PTPLA | protein tyrosine phosphatase-like (proline instead of catalytic arginine), member A | 1.55 | 25.6 | 16.56 | 4.6 x 10^-11^ | multicellular organismal development | membrane |
| 233488_at | RNASE7 | ribonuclease, RNase A family, 7 | 0.42 | 6.9 | 16.50 | 3.0 x 10^-5^ | response to bacterium | extracellular region |
| 1557918_s_at | SLC16A1 | solute carrier family 16, member 1 (monocarboxylic acid transporter 1) | 0.45 | 7.5 | 16.50 | 5.8 x 10^-4^ | transport | membrane fraction |
| 225018_at | SPIRE1 | spire homolog 1 (Drosophila) | 1.86 | 30.4 | 16.38 | 2.8 x 10^-6^ | transport | cytoplasm |
| 218677_at | S100A14 | S100 calcium binding protein A14 | 9.18 | 150.2 | 16.36 | 2.5 x 10^-7^ |  | cytoplasm |
| 240877_x_at | --- | --- | 0.19 | 3.0 | 16.34 | 3.7 x 10^-5^ |  |  |
| 204726_at | CDH13 | cadherin 13, H-cadherin (heart) | 0.64 | 10.4 | 16.31 | 1.1 x 10^-4^ | positive regulation of endothelial cell proliferation | extracellular space |
| 1557578_at | PHLDB2 | Pleckstrin homology-like domain, family B, member 2 | 0.27 | 4.4 | 16.19 | 2.4 x 10^-7^ |  | cytoplasm |
| 206866_at | CDH4 | cadherin 4, type 1, R-cadherin (retinal) | 0.23 | 3.7 | 16.19 | 3.4 x 10^-6^ | cell adhesion | plasma membrane |
| 203348_s_at | ETV5 | ets variant 5 | 0.61 | 9.8 | 16.14 | 1.1 x 10^-5^ | regulation of transcription, DNA-dependent | nucleus |
| 1558517_s_at | LRRC8C | leucine rich repeat containing 8 family, member C | 0.51 | 8.3 | 16.12 | 9.6 x 10^-5^ |  | membrane |
| 202847_at | PCK2 | phosphoenolpyruvate carboxykinase 2 (mitochondrial) | 3.79 | 61.1 | 16.12 | 1.0 x 10^-8^ | gluconeogenesis | mitochondrion |
| 220664_at | SPRR2C | small proline-rich protein 2C (pseudogene) | 0.11 | 1.8 | 16.10 | 3.0 x 10^-5^ | epidermis development | cornified envelope |
| 227486_at | NT5E | 5'-nucleotidase, ecto (CD73) | 0.37 | 5.9 | 16.07 | 8.1 x 10^-5^ | purine nucleotide biosynthetic process | membrane fraction |
| 227963_at | --- | --- | 0.51 | 8.2 | 15.98 | 2.3 x 10^-3^ |  |  |
| 224579_at | SLC38A1 | solute carrier family 38, member 1 | 11.36 | 181.2 | 15.95 | 1.0 x 10^-7^ | transport | membrane fraction |
| 200859_x_at | FLNA | filamin A, alpha (actin binding protein 280) | 3.41 | 54.4 | 15.95 | 3.0 x 10^-5^ | inhibition of adenylate cyclase activity by dopamine receptor signaling pathway | nucleus |
| 204748_at | PTGS2 | prostaglandin-endoperoxide synthase 2 (prostaglandin G/H synthase and cyclooxygenase) | 2.78 | 44.1 | 15.89 | 2.9 x 10^-4^ | prostaglandin biosynthetic process | nucleus |
| 205404_at | HSD11B1 | hydroxysteroid (11-beta) dehydrogenase 1 | 0.54 | 8.6 | 15.88 | 8.6 x 10^-5^ | lipid metabolic process | endoplasmic reticulum |
| 221667_s_at | HSPB8 | heat shock 22kDa protein 8 | 0.81 | 12.9 | 15.85 | 6.6 x 10^-6^ | response to stress | intracellular |
| 201474_s_at | ITGA3 | integrin, alpha 3 (antigen CD49C, alpha 3 subunit of VLA-3 receptor) | 3.16 | 50.1 | 15.85 | 5.0 x 10^-9^ | neuron migration | plasma membrane |
| 204955_at | SRPX | sushi-repeat-containing protein, X-linked | 0.29 | 4.6 | 15.83 | 1.9 x 10^-3^ | cell adhesion | cell surface |
| 1569183_a_at | CHM | choroideremia (Rab escort protein 1) | 0.12 | 1.9 | 15.76 | 2.3 x 10^-4^ | blood vessel development | nucleus |
| 210248_at | WNT7A | wingless-type MMTV integration site family, member 7A | 0.19 | 2.9 | 15.72 | 1.0 x 10^-5^ | signal transduction | extracellular region |
| 229296_at | LOC100128501 | hypothetical protein LOC100128501 | 2.47 | 38.8 | 15.72 | 3.4 x 10^-7^ |  |  |
| 239586_at | FAM83A | family with sequence similarity 83, member A | 2.03 | 31.9 | 15.72 | 4.4 x 10^-4^ |  |  |
| 235085_at | PRAGMIN | homolog of rat pragma of Rnd2 | 3.93 | 61.7 | 15.70 | 3.4 x 10^-4^ | protein amino acid phosphorylation |  |
| 202284_s_at | CDKN1A | cyclin-dependent kinase inhibitor 1A (p21, Cip1) | 10.69 | 167.8 | 15.70 | 2.0 x 10^-6^ | regulation of cyclin-dependent protein kinase activity | cyclin-dependent protein kinase holoenzyme complex |
| 223723_at | MFI2 | antigen p97 (melanoma associated) identified by monoclonal antibodies 133.2 and 96.5 | 0.16 | 2.5 | 15.64 | 6.9 x 10^-3^ | transport | extracellular region |
| 242979_at | IRS1 | insulin receptor substrate 1 | 0.35 | 5.4 | 15.61 | 3.1 x 10^-5^ | positive regulation of mesenchymal cell proliferation | nucleus |
| 208693_s_at | GARS | glycyl-tRNA synthetase | 13.90 | 216.8 | 15.60 | 4.3 x 10^-12^ | translation | soluble fraction |
| 210357_s_at | SMOX | spermine oxidase | 0.57 | 8.9 | 15.59 | 1.9 x 10^-8^ | spermine catabolic process | cytoplasm |
| 225303_at | KIRREL | kin of IRRE like (Drosophila) | 0.52 | 8.1 | 15.50 | 5.1 x 10^-4^ | excretion | plasma membrane |
| 201615_x_at | CALD1 | caldesmon 1 | 0.30 | 4.6 | 15.41 | 2.0 x 10^-4^ | cell motion | membrane fraction |
| 214752_x_at | FLNA | filamin A, alpha (actin binding protein 280) | 2.24 | 34.4 | 15.36 | 1.8 x 10^-6^ | inhibition of adenylate cyclase activity by dopamine receptor signaling pathway | nucleus |
| 207020_at | HSF2BP | heat shock transcription factor 2 binding protein | 0.64 | 9.9 | 15.33 | 7.1 x 10^-6^ | transcription from RNA polymerase II promoter | cytosol |
| 227345_at | TNFRSF10D | tumor necrosis factor receptor superfamily, member 10d, decoy with truncated death domain | 1.50 | 23.0 | 15.32 | 6.0 x 10^-4^ | apoptosis | membrane |
| 208964_s_at | FADS1 | fatty acid desaturase 1 | 1.89 | 28.8 | 15.29 | 6.6 x 10^-4^ | lipid metabolic process | membrane fraction |
| 209295_at | TNFRSF10B | tumor necrosis factor receptor superfamily, member 10b | 8.50 | 130.0 | 15.29 | 2.2 x 10^-9^ | apoptosis | plasma membrane |
| 217875_s_at | PMEPA1 | prostate transmembrane protein, androgen induced 1 | 0.44 | 6.7 | 15.24 | 1.7 x 10^-6^ | androgen receptor signaling pathway | plasma membrane |
| 238133_at | --- | --- | 0.31 | 4.7 | 15.20 | 2.0 x 10^-3^ |  |  |
| 217312_s_at | COL7A1 | collagen, type VII, alpha 1 | 1.05 | 15.9 | 15.12 | 9.4 x 10^-6^ | cell adhesion | extracellular region |
| 205490_x_at | GJB3 | gap junction protein, beta 3, 31kDa | 3.65 | 55.2 | 15.12 | 2.5 x 10^-6^ | placenta development | plasma membrane |
| 231572_at | LOC136242 | Peptidase S1 domain-containing protein LOC136242 | 0.28 | 4.2 | 15.11 | 1.2 x 10^-4^ | proteolysis | extracellular region |
| 223391_at | SGPP1 | sphingosine-1-phosphate phosphatase 1 | 2.18 | 32.8 | 15.02 | 1.6 x 10^-7^ | sphingolipid metabolic process | membrane fraction |
| 214096_s_at | SHMT2 | serine hydroxymethyltransferase 2 (mitochondrial) | 7.59 | 113.3 | 14.93 | 5.4 x 10^-11^ | glycine metabolic process | mitochondrion |
| 201148_s_at | TIMP3 | TIMP metallopeptidase inhibitor 3 | 0.63 | 9.4 | 14.91 | 5.3 x 10^-5^ | transmembrane receptor protein tyrosine kinase signaling pathway | extracellular region |
| 206237_s_at | NRG1 | neuregulin 1 | 0.38 | 5.6 | 14.89 | 7.3 x 10^-7^ | cell communication | extracellular region |
| 1554795_a_at | FBLIM1 | filamin binding LIM protein 1 | 0.62 | 9.2 | 14.85 | 1.2 x 10^-6^ | cell adhesion | cytoplasm |
| 224624_at | LRRC8A | leucine rich repeat containing 8 family, member A | 15.26 | 225.5 | 14.78 | 2.0 x 10^-7^ |  | membrane |
| 224833_at | ETS1 | v-ets erythroblastosis virus E26 oncogene homolog 1 (avian) | 6.40 | 94.6 | 14.78 | 3.1 x 10^-6^ | transcription | nucleus |
| 221827_at | RBCK1 | RanBP-type and C3HC4-type zinc finger containing 1 | 9.96 | 146.5 | 14.71 | 2.0 x 10^-6^ | protein modification process | intracellular |
| 209301_at | CA2 | carbonic anhydrase II | 0.67 | 9.8 | 14.70 | 4.9 x 10^-5^ | morphogenesis of an epithelium | nucleus |
| 222383_s_at | ALOXE3 | arachidonate lipoxygenase 3 | 0.11 | 1.7 | 14.69 | 2.5 x 10^-5^ | leukotriene metabolic process |  |
| 215243_s_at | GJB3 | gap junction protein, beta 3, 31kDa | 2.77 | 40.5 | 14.63 | 3.5 x 10^-6^ | placenta development | plasma membrane |
| 202196_s_at | DKK3 | dickkopf homolog 3 (Xenopus laevis) | 1.38 | 20.1 | 14.60 | 2.0 x 10^-4^ | multicellular organismal development | extracellular region |
| 209651_at | TGFB1I1 | transforming growth factor beta 1 induced transcript 1 | 0.75 | 10.9 | 14.59 | 6.1 x 10^-5^ | transcription from RNA polymerase II promoter | intracellular |
| 210868_s_at | ELOVL6 | ELOVL family member 6, elongation of long chain fatty acids (FEN1/Elo2, SUR4/Elo3-like, yeast) | 0.74 | 10.7 | 14.57 | 8.3 x 10^-7^ | fatty acid biosynthetic process | mitochondrion |
| 218995_s_at | EDN1 | endothelin 1 | 0.89 | 13.0 | 14.55 | 1.3 x 10^-4^ | skeletal system development | extracellular region |
| 1554020_at | BICD1 | bicaudal D homolog 1 (Drosophila) | 0.43 | 6.3 | 14.55 | 1.4 x 10^-5^ | RNA processing | Golgi apparatus |
| 213865_at | DCBLD2 | discoidin, CUB and LCCL domain containing 2 | 0.38 | 5.6 | 14.53 | 3.9 x 10^-6^ | cell adhesion | integral to plasma membrane |
| 218796_at | FERMT1 | fermitin family homolog 1 (Drosophila) | 5.63 | 81.5 | 14.50 | 2.6 x 10^-4^ | cell adhesion | cytoplasm |
| 204751_x_at | DSC2 | desmocollin 2 | 0.49 | 7.1 | 14.45 | 6.7 x 10^-5^ | cell adhesion | plasma membrane |
| 202949_s_at | FHL2 | four and a half LIM domains 2 | 6.70 | 96.4 | 14.40 | 1.3 x 10^-10^ | transcription | nucleus |
| 217196_s_at | CAMSAP1L1 | calmodulin regulated spectrin-associated protein 1-like 1 | 4.18 | 60.1 | 14.37 | 1.2 x 10^-9^ |  |  |
| 204411_at | KIF21B | kinesin family member 21B | 0.24 | 3.5 | 14.25 | 4.0 x 10^-5^ | microtubule-based movement | cytoplasm |
| 208963_x_at | FADS1 | fatty acid desaturase 1 | 1.45 | 20.6 | 14.23 | 2.2 x 10^-4^ | lipid metabolic process | membrane fraction |
| 237435_at | --- | --- | 0.10 | 1.5 | 14.22 | 9.8 x 10^-4^ |  |  |
| 227998_at | S100A16 | S100 calcium binding protein A16 | 18.45 | 261.8 | 14.19 | 3.3 x 10^-6^ |  |  |
| 205105_at | MAN2A1 | mannosidase, alpha, class 2A, member 1 | 4.66 | 65.9 | 14.16 | 3.6 x 10^-5^ | in utero embryonic development | Golgi membrane |
| 218237_s_at | SLC38A1 | solute carrier family 38, member 1 | 10.58 | 149.7 | 14.15 | 1.6 x 10^-8^ | transport | membrane fraction |
| 227614_at | HKDC1 | hexokinase domain containing 1 | 0.21 | 2.9 | 14.07 | 1.0 x 10^-3^ | carbohydrate metabolic process |  |
| 211162_x_at | SCD | stearoyl-CoA desaturase (delta-9-desaturase) | 1.10 | 15.5 | 14.05 | 2.7 x 10^-3^ | lipid metabolic process | endoplasmic reticulum |
| 225799_at | LOC541471 //NCRNA00152 | hypothetical LOC541471 /// non-protein coding RNA 152 | 3.29 | 46.2 | 14.04 | 3.3 x 10^-6^ |  |  |
| 212242_at | TUBA4A | tubulin, alpha 4a | 7.50 | 105.2 | 14.02 | 1.7 x 10^-3^ | microtubule-based process | microtubule |
| 1555420_a_at | KLF7 | Kruppel-like factor 7 (ubiquitous) | 1.63 | 22.9 | 14.02 | 2.2 x 10^-9^ | transcription | intracellular |
| 206122_at | SOX15 | SRY (sex determining region Y)-box 15 | 1.93 | 27.0 | 13.97 | 2.9 x 10^-8^ | negative regulation of transcription from RNA polymerase II promoter | nucleus |
| 208510_s_at | PPARG | peroxisome proliferator-activated receptor gamma | 0.59 | 8.2 | 13.95 | 1.2 x 10^-3^ | negative regulation of transcription from RNA polymerase II promoter | nucleus |
| 212845_at | SAMD4A | sterile alpha motif domain containing 4A | 1.41 | 19.7 | 13.93 | 7.4 x 10^-7^ | positive regulation of translation | synaptosome |
| 244107_at | --- | --- | 0.68 | 9.4 | 13.93 | 2.3 x 10^-7^ |  |  |
| 236599_at | --- | --- | 0.40 | 5.6 | 13.92 | 6.6 x 10^-6^ |  |  |
| 219412_at | RAB38 | RAB38, member RAS oncogene family | 5.82 | 80.3 | 13.80 | 2.4 x 10^-7^ | transport | soluble fraction |
| 208025_s_at | HMGA2 | high mobility group AT-hook 2 | 2.65 | 36.5 | 13.78 | 1.3 x 10^-3^ | establishment or maintenance of chromatin architecture | nuclear chromosome |
| 201645_at | TNC | tenascin C | 8.60 | 118.0 | 13.73 | 3.2 x 10^-6^ | cell adhesion | extracellular region |
| 214853_s_at | SHC1 | SHC (Src homology 2 domain containing) transforming protein 1 | 14.86 | 204.0 | 13.73 | 3.3 x 10^-8^ | MAPKKK cascade | cytoplasm |
| 219926_at | POPDC3 | popeye domain containing 3 | 0.09 | 1.3 | 13.70 | 6.5 x 10^-7^ |  | membrane |
| 222802_at | EDN1 | endothelin 1 | 1.30 | 17.7 | 13.70 | 3.5 x 10^-3^ | skeletal system development | extracellular region |
| 205829_at | HSD17B1 | hydroxysteroid (17-beta) dehydrogenase 1 | 0.60 | 8.2 | 13.65 | 1.2 x 10^-7^ | steroid biosynthetic process | cytoplasm |
| 208785_s_at | MAP1LC3B | microtubule-associated protein 1 light chain 3 beta | 15.37 | 209.4 | 13.62 | 2.5 x 10^-5^ | autophagy | intracellular |
| 203349_s_at | ETV5 | ets variant 5 | 0.92 | 12.5 | 13.61 | 1.6 x 10^-7^ | regulation of transcription, DNA-dependent | nucleus |
| 222753_s_at | SPCS3 | signal peptidase complex subunit 3 homolog (S. cerevisiae) | 4.18 | 56.7 | 13.59 | 2.4 x 10^-6^ | signal peptide processing | endoplasmic reticulum |
| 210113_s_at | NLRP1 | NLR family, pyrin domain containing 1 | 0.54 | 7.3 | 13.56 | 6.0 x 10^-6^ | apoptosis | intracellular |
| 224927_at | KIAA1949 | KIAA1949 | 1.87 | 25.3 | 13.52 | 1.1 x 10^-5^ |  | cytoplasm |
| 1557779_at | --- | --- | 0.17 | 2.3 | 13.45 | 3.0 x 10^-5^ |  |  |
| 218018_at | PDXK | pyridoxal (pyridoxine, vitamin B6) kinase | 4.18 | 56.2 | 13.45 | 2.1 x 10^-4^ | cell proliferation | cytoplasm |
| 204059_s_at | ME1 | malic enzyme 1, NADP(+)-dependent, cytosolic | 6.71 | 89.8 | 13.38 | 2.4 x 10^-6^ | carbohydrate metabolic process | nucleus |
| 212662_at | PVR | poliovirus receptor | 0.73 | 9.7 | 13.37 | 7.1 x 10^-12^ | positive regulation of natural killer cell mediated cytotoxicity directed against tumor cell target | extracellular region |
| 217975_at | WBP5 | WW domain binding protein 5 | 8.62 | 114.8 | 13.32 | 4.7 x 10^-7^ |  |  |
| 1559517_a_at | SPIRE1 | spire homolog 1 (Drosophila) | 0.62 | 8.2 | 13.32 | 5.0 x 10^-7^ | transport | cytoplasm |
| 234699_at | RNASE7 | ribonuclease, RNase A family, 7 | 0.27 | 3.6 | 13.32 | 4.7 x 10^-8^ | response to bacterium | extracellular region |
| 208789_at | PTRF | polymerase I and transcript release factor | 2.87 | 38.2 | 13.31 | 2.5 x 10^-4^ | transcription | nucleus |
| 219901_at | FGD6 | FYVE, RhoGEF and PH domain containing 6 | 0.97 | 12.9 | 13.29 | 5.0 x 10^-5^ | cytoskeleton organization | ruffle |
| 200650_s_at | LDHA | lactate dehydrogenase A | 37.89 | 502.7 | 13.27 | 3.5 x 10^-8^ | carbohydrate metabolic process | cytoplasm |
| 201787_at | FBLN1 LOC100133843 | fibulin 1 similar to Fibulin 1 | 0.69 | 9.0 | 13.20 | 2.7 x 10^-7^ | interspecies interaction between organisms | extracellular region |
| 231880_at | FAM40B | family with sequence similarity 40, member B | 0.34 | 4.5 | 13.20 | 1.5 x 10^-6^ |  |  |
| 225045_at | CCDC88A | coiled-coil domain containing 88A | 0.56 | 7.3 | 13.16 | 2.6 x 10^-7^ | regulation of protein amino acid phosphorylation | cytoplasm |
| 242893_at | --- | --- | 0.22 | 2.8 | 13.11 | 4.1 x 10^-3^ |  |  |
| 234954_at | --- | --- | 1.05 | 13.7 | 13.09 | 1.7 x 10^-7^ |  |  |
| 235117_at | CHAC2 | ChaC, cation transport regulator homolog 2 (E. coli) | 1.02 | 13.4 | 13.07 | 7.9 x 10^-6^ |  |  |
| 209885_at | RHOD | ras homolog gene family, member D | 2.50 | 32.6 | 13.05 | 9.2 x 10^-8^ | small GTPase mediated signal transduction | intracellular |
| 220289_s_at | AIM1L | absent in melanoma 1-like | 0.44 | 5.7 | 13.02 | 3.8 x 10^-7^ |  |  |
| 200733_s_at | PTP4A1 | protein tyrosine phosphatase type IVA, member 1 | 13.14 | 171.0 | 13.01 | 1.1 x 10^-4^ | protein amino acid dephosphorylation | nucleus |
| 219496_at | ANKRD57 | ankyrin repeat domain 57 | 5.42 | 70.5 | 13.00 | 3.9 x 10^-8^ |  |  |
| 225285_at | BCAT1 | branched chain aminotransferase 1, cytosolic | 0.75 | 9.8 | 12.99 | 1.3 x 10^-4^ | G1 | cytoplasm |
| 210510_s_at | NRP1 | neuropilin 1 | 0.21 | 2.7 | 12.98 | 2.4 x 10^-7^ | angiogenesis | extracellular region |
| 219555_s_at | CENPN | centromere protein N | 0.67 | 8.6 | 12.97 | 5.8 x 10^-3^ |  | chromosome, centromeric region |
| 231894_at | --- | --- | 1.36 | 17.7 | 12.94 | 2.5 x 10^-7^ |  |  |
| 1554026_a_at | MYO10 | myosin X | 0.68 | 8.7 | 12.92 | 6.4 x 10^-5^ | signal transduction | cytoskeleton |
| 229432_at | NAGS | N-acetylglutamate synthase | 0.76 | 9.8 | 12.90 | 9.5 x 10^-5^ | urea cycle | mitochondrion |
| 200770_s_at | LAMC1 | laminin, gamma 1 (formerly LAMB2) | 3.49 | 45.0 | 12.89 | 5.5 x 10^-8^ | protein complex assembly | extracellular region |
| 225878_at | KIF1B | kinesin family member 1B | 1.63 | 21.0 | 12.89 | 3.8 x 10^-8^ | microtubule-based movement | cytoplasm |
| 209087_x_at | MCAM | melanoma cell adhesion molecule | 1.16 | 14.9 | 12.87 | 9.8 x 10^-3^ | cell adhesion | nucleus |
| 1553530_a_at | ITGB1 | integrin, beta 1 (fibronectin receptor, beta polypeptide, antigen CD29 includes MDF2, MSK12) | 16.89 | 216.8 | 12.83 | 7.4 x 10^-7^ | G1 | ruffle |
| 215465_at | ABCA12 | ATP-binding cassette, sub-family A (ABC1), member 12 | 0.55 | 7.1 | 12.83 | 3.4 x 10^-6^ | transport | mitochondrial inner membrane |
| 202679_at | NPC1 | Niemann-Pick disease, type C1 | 5.23 | 66.8 | 12.78 | 1.1 x 10^-8^ | endocytosis | nuclear envelope |
| 238933_at | IRS1 | Insulin receptor substrate 1 | 0.37 | 4.8 | 12.74 | 1.4 x 10^-3^ | positive regulation of mesenchymal cell proliferation | nucleus |
| 223195_s_at | SESN2 | sestrin 2 | 2.34 | 29.8 | 12.72 | 1.1 x 10^-4^ | cell cycle arrest | nucleus |
| 210999_s_at | GRB10 | growth factor receptor-bound protein 10 | 1.86 | 23.6 | 12.70 | 2.1 x 10^-7^ | signal transduction | cytoplasm |
| 219973_at | ARSJ | arylsulfatase family, member J | 1.62 | 20.6 | 12.69 | 1.5 x 10^-9^ | metabolic process | extracellular region |
| 205796_at | TCP11L1 | t-complex 11 (mouse)-like 1 | 0.38 | 4.9 | 12.69 | 8.0 x 10^-5^ |  | cytoplasm |
| 223625_at | FAM126A | family with sequence similarity 126, member A | 0.30 | 3.8 | 12.68 | 3.5 x 10^-6^ |  | cytoplasm |
| 1564651_at | LOC221710 | hypothetical protein LOC221710 | 0.45 | 5.7 | 12.67 | 1.0 x 10^-4^ | regulation of transcription |  |
| 240432_x_at | --- | --- | 1.22 | 15.4 | 12.65 | 8.1 x 10^-7^ |  |  |
| 204760_s_at | NR1D1 /// THRA | nuclear receptor subfamily 1, group D, member 1 /// thyroid hormone receptor, alpha (erythroblastic leukemia viral (v-erb-a) oncogene homolog, avian) | 0.31 | 4.0 | 12.65 | 1.5 x 10^-4^ | cartilage condensation | nucleus |
| 205290_s_at | BMP2 | bone morphogenetic protein 2 | 0.48 | 6.0 | 12.62 | 7.0 x 10^-12^ | skeletal system development | extracellular region |
| 204475_at | MMP1 | matrix metallopeptidase 1 (interstitial collagenase) | 2.29 | 28.8 | 12.60 | 9.8 x 10^-5^ | proteolysis | extracellular region |
| 204203_at | CEBPG | CCAAT/enhancer binding protein (C/EBP), gamma | 4.29 | 54.0 | 12.60 | 3.0 x 10^-6^ | liver development | nucleus |
| 209825_s_at | UCK2 | uridine-cytidine kinase 2 | 2.15 | 27.0 | 12.54 | 1.7 x 10^-5^ | metabolic process | cytosol |
| 212298_at | NRP1 | neuropilin 1 | 0.97 | 12.1 | 12.53 | 3.1 x 10^-6^ | angiogenesis | extracellular region |
| 224794_s_at | CERCAM | cerebral endothelial cell adhesion molecule | 0.49 | 6.1 | 12.50 | 5.7 x 10^-8^ | cell motion | endoplasmic reticulum |
| 213112_s_at | SQSTM1 | sequestosome 1 | 1.13 | 14.0 | 12.46 | 6.6 x 10^-5^ | ubiquitin-dependent protein catabolic process | nucleus |
| 206074_s_at | HMGA1 | high mobility group AT-hook 1 | 7.64 | 95.0 | 12.44 | 1.3 x 10^-6^ | DNA unwinding during replication | chromatin |
| 206371_at | FOLR3 | folate receptor 3 (gamma) | 0.33 | 4.1 | 12.43 | 4.6 x 10^-4^ | folic acid transport | extracellular region |
| 212680_x_at | PPP1R14B | protein phosphatase 1, regulatory (inhibitor) subunit 14B | 3.81 | 47.3 | 12.43 | 1.1 x 10^-8^ | regulation of phosphorylation | cytoplasm |
| 202806_at | DBN1 | drebrin 1 | 1.54 | 19.1 | 12.42 | 5.9 x 10^-8^ | actin filament organization | intracellular |
| 208786_s_at | MAP1LC3B | microtubule-associated protein 1 light chain 3 beta | 17.65 | 218.5 | 12.39 | 1.6 x 10^-5^ | autophagy | intracellular |
| 219909_at | MMP28 | matrix metallopeptidase 28 | 0.26 | 3.2 | 12.36 | 1.3 x 10^-3^ | proteolysis | extracellular region |
| 235103_at | MAN2A1 | mannosidase, alpha, class 2A, member 1 | 2.60 | 32.2 | 12.36 | 5.0 x 10^-5^ | in utero embryonic development | Golgi membrane |
| 205574_x_at | BMP1 | bone morphogenetic protein 1 | 0.42 | 5.2 | 12.32 | 1.7 x 10^-5^ | skeletal system development | extracellular space |
| 244025_at | --- | --- | 0.45 | 5.5 | 12.32 | 5.9 x 10^-7^ |  |  |
| 239272_at | MMP28 | matrix metallopeptidase 28 | 1.10 | 13.6 | 12.29 | 3.8 x 10^-9^ | proteolysis | extracellular region |
| 201263_at | TARS | threonyl-tRNA synthetase | 9.46 | 115.9 | 12.25 | 3.6 x 10^-12^ | translation | cytoplasm |
| 229641_at | --- | --- | 0.40 | 4.9 | 12.25 | 4.8 x 10^-7^ |  |  |
| 225450_at | AMOTL1 | angiomotin like 1 | 2.28 | 27.9 | 12.23 | 3.6 x 10^-6^ |  | cytoplasm |
| 205330_at | MN1 | meningioma (disrupted in balanced translocation) 1 | 0.46 | 5.6 | 12.20 | 8.6 x 10^-8^ | cell cycle | nucleus |
| 221881_s_at | CLIC4 | chlorideintracellular channel 4 | 1.30 | 15.8 | 12.17 | 2.8 x 10^-6^ | transport | intracellular |
| 227180_at | ELOVL7 | ELOVL family member 7, elongation of long chain fatty acids (yeast) | 2.31 | 28.1 | 12.16 | 2.9 x 10^-4^ | fatty acid biosynthetic process | endoplasmic reticulum |
| 203287_at | LAD1 | ladinin 1 | 2.23 | 27.0 | 12.13 | 7.2 x 10^-6^ |  | extracellular region |
| 1561741_at | --- | --- | 0.37 | 4.4 | 12.09 | 2.1 x 10^-6^ |  |  |
| 212765_at | CAMSAP1L1 | calmodulin regulated spectrin-associated protein 1-like 1 | 6.49 | 78.4 | 12.08 | 1.1 x 10^-9^ |  |  |
| 204560_at | FKBP5 | FK506 binding protein 5 | 0.56 | 6.8 | 12.07 | 3.1 x 10^-3^ | protein folding | nucleus |
| 224580_at | SLC38A1 | solute carrier family 38, member 1 | 1.27 | 15.3 | 12.01 | 7.8 x 10^-6^ | transport | membrane fraction |
| 213139_at | SNAI2 | snail homolog 2 (Drosophila) | 5.99 | 71.9 | 12.00 | 2.0 x 10^-4^ | negative regulation of transcription from RNA polymerase II promoter | intracellular |
| 1554008_at | OSMR | oncostatin M receptor | 1.35 | 16.2 | 12.00 | 3.0 x 10^-6^ | positive regulation of acute inflammatory response | oncostatin-M receptor complex |
| 227856_at | C4orf32 | chromosome 4 open reading frame 32 | 2.18 | 26.2 | 12.00 | 1.3 x 10^-10^ |  | membrane |
| 211340_s_at | MCAM | melanoma cell adhesion molecule | 1.54 | 18.4 | 11.99 | 4.4 x 10^-3^ | cell adhesion | nucleus |
| 218084_x_at | FXYD5 | FXYD domain containing ion transport regulator 5 | 3.81 | 45.6 | 11.98 | 6.5 x 10^-8^ | transport | membrane |
| 200771_at | LAMC1 | laminin, gamma 1 (formerly LAMB2) | 6.08 | 72.8 | 11.97 | 3.2 x 10^-7^ | protein complex assembly | extracellular region |
| 226390_at | STARD4 | StAR-related lipid transfer (START) domain containing 4 | 2.61 | 31.3 | 11.96 | 3.1 x 10^-7^ | steroid biosynthetic process |  |
| 227006_at | PPP1R14A | protein phosphatase 1, regulatory (inhibitor) subunit 14A | 0.06 | 0.7 | 11.96 | 2.4 x 10^-4^ | regulation of phosphorylation | cytoplasm |
| 206125_s_at | KLK8 | kallikrein-related peptidase 8 | 0.70 | 8.4 | 11.92 | 2.8 x 10^-6^ | proteolysis | extracellular region |
| 235419_at | --- | --- | 3.42 | 40.7 | 11.91 | 5.0 x 10^-4^ |  |  |
| 211071_s_at | MLLT11 | myeloid/lymphoid or mixed-lineage leukemia (trithorax homolog, Drosophila); translocated to, 11 | 1.46 | 17.3 | 11.89 | 4.9 x 10^-4^ |  | extracellular region |
| 227034_at | ANKRD57 | ankyrin repeat domain 57 | 7.81 | 92.2 | 11.80 | 5.8 x 10^-8^ |  |  |
| 210018_x_at | MALT1 | mucosa associated lymphoid tissue lymphoma translocation gene 1 | 1.58 | 18.6 | 11.80 | 9.5 x 10^-11^ | positive regulation of T cell cytokine production | nucleus |
| 203071_at | SEMA3B | sema domain, immunoglobulin domain (Ig), short basic domain, secreted, (semaphorin) 3B | 0.31 | 3.6 | 11.75 | 2.3 x 10^-5^ | cell-cell signaling | extracellular region |
| 218826_at | SLC35F2 | solute carrier family 35, member F2 | 1.15 | 13.5 | 11.74 | 2.5 x 10^-5^ | transport | membrane |
| 205067_at | IL1B | interleukin 1, beta | 1.79 | 20.9 | 11.72 | 6.3 x 10^-3^ | activation of MAPK activity | extracellular region |
| 208002_s_at | ACOT7 | acyl-CoA thioesterase 7 | 3.74 | 43.6 | 11.67 | 9.9 x 10^-6^ | lipid metabolic process | cytoplasm |
| 238482_at | KLF7 | Kruppel-like factor 7 (ubiquitous) | 1.06 | 12.3 | 11.67 | 2.3 x 10^-6^ | transcription | intracellular |
| 228892_at | SH3RF2 | SH3 domain containing ring finger 2 | 0.39 | 4.6 | 11.64 | 5.2 x 10^-3^ | modification-dependent protein catabolic process |  |
| 201325_s_at | EMP1 | epithelial membrane protein 1 | 2.59 | 30.2 | 11.62 | 9.4 x 10^-6^ | multicellular organismal development | membrane fraction |
| 216336_x_at | MT1E /// MT1H /// MT1M /// MT1P2 | metallothionein 1E /// metallothionein 1H /// metallothionein 1M /// metallothionein 1 pseudogene 2 | 22.18 | 257.6 | 11.61 | 5.5 x 10^-7^ |  | cytoplasm |
| 217165_x_at | MT1F | metallothionein 1F | 17.01 | 197.6 | 11.61 | 3.8 x 10^-7^ |  | cytoplasm |
| 203256_at | CDH3 | cadherin 3, type 1, P-cadherin (placental) | 8.19 | 95.0 | 11.60 | 3.5 x 10^-5^ | cell adhesion | plasma membrane |
| 228161_at | RAB32 | RAB32, member RAS oncogene family | 0.30 | 3.5 | 11.55 | 9.0 x 10^-6^ | small GTPase mediated signal transduction | mitochondrion |
| 206581_at | BNC1 | basonuclin 1 | 0.65 | 7.5 | 11.51 | 2.9 x 10^-6^ | transcription | intracellular |
| 235417_at | SPOCD1 | SPOC domain containing 1 | 0.22 | 2.6 | 11.51 | 1.2 x 10^-3^ | transcription |  |
| 242509_at | --- | --- | 0.68 | 7.8 | 11.50 | 1.0 x 10^-5^ |  |  |
| 222812_s_at | RHOF | ras homolog gene family, member F (in filopodia) | 0.27 | 3.1 | 11.50 | 5.2 x 10^-8^ | actin filament organization | intracellular |
| 1555292_at | FAM40B | family with sequence similarity 40, member B | 0.23 | 2.7 | 11.48 | 3.5 x 10^-5^ |  |  |
| 207345_at | FST | follistatin | 0.57 | 6.5 | 11.45 | 3.0 x 10^-5^ | negative regulation of transcription from RNA polymerase II promoter | extracellular region |
| 238964_at | FIGN | fidgetin | 0.09 | 1.0 | 11.44 | 9.4 x 10^-4^ |  |  |
| 203786_s_at | TPD52L1 | tumor protein D52-like 1 | 7.35 | 84.0 | 11.43 | 1.4 x 10^-4^ | G2 | cytoplasm |
| 216620_s_at | ARHGEF10 | Rho guanine nucleotide exchange factor (GEF) 10 | 1.62 | 18.5 | 11.42 | 1.1 x 10^-10^ | regulation of Rho protein signal transduction | intracellular |
| 1552277_a_at | C9orf30 | chromosome 9 open reading frame 30 | 3.01 | 34.3 | 11.39 | 2.3 x 10^-9^ | multicellular organismal development | plasma membrane |
| 206023_at | NMU | neuromedin U | 1.03 | 11.7 | 11.39 | 8.4 x 10^-6^ | regulation of smooth muscle contraction | extracellular region |
| 208309_s_at | MALT1 | mucosa associated lymphoid tissue lymphoma translocation gene 1 | 1.86 | 21.2 | 11.39 | 7.2 x 10^-9^ | positive regulation of T cell cytokine production | nucleus |
| 240991_at | --- | --- | 2.37 | 27.0 | 11.38 | 1.4 x 10^-6^ |  |  |
| 1555137_a_at | FGD6 | FYVE, RhoGEF and PH domain containing 6 | 0.70 | 7.9 | 11.37 | 4.9 x 10^-4^ | cytoskeleton organization | ruffle |
| 242626_at | SAMD5 | sterile alpha motif domain containing 5 | 0.29 | 3.3 | 11.36 | 7.4 x 10^-3^ |  |  |
| 40284_at | FOXA2 | forkhead box A2 | 2.11 | 24.0 | 11.35 | 5.7 x 10^-3^ | ectoderm formation | nucleus |
| 227212_s_at | PHF19 | PHD finger protein 19 | 0.69 | 7.8 | 11.26 | 8.6 x 10^-4^ | transcription | nucleus |
| 218644_at | PLEK2 | pleckstrin 2 | 4.37 | 49.2 | 11.25 | 4.6 x 10^-3^ | intracellular signaling cascade | nucleus |
| 227443_at | C9orf150 | chromosome 9 open reading frame 150 | 3.49 | 39.2 | 11.23 | 4.1 x 10^-6^ |  |  |
| 225662_at | ZAK | sterile alpha motif and leucine zipper containing kinase AZK | 3.37 | 37.8 | 11.22 | 3.6 x 10^-7^ | cell cycle checkpoint | nucleus |
| 200953_s_at | CCND2 | cyclin D2 | 6.19 | 69.4 | 11.22 | 2.4 x 10^-4^ | G1 | cyclin-dependent protein kinase holoenzyme complex |
| 221059_s_at | COTL1 | coactosin-like 1 (Dictyostelium) | 4.55 | 51.0 | 11.20 | 1.1 x 10^-4^ | carbohydrate metabolic process | Golgi membrane |
| 224952_at | TANC2 | tetratricopeptide repeat, ankyrin repeat and coiled-coil containing 2 | 4.24 | 47.4 | 11.18 | 2.4 x 10^-12^ |  |  |
| 221510_s_at | GLS | glutaminase | 2.66 | 29.6 | 11.11 | 2.9 x 10^-7^ | glutamine metabolic process | mitochondrion |
| 229545_at | FERMT1 | fermitin family homolog 1 (Drosophila) | 1.94 | 21.6 | 11.10 | 1.9 x 10^-4^ | cell adhesion | cytoplasm |
| 204976_s_at | AMMECR1 | Alport syndrome, mental retardation, midface hypoplasia and elliptocytosis chromosomal region gene 1 | 5.21 | 57.8 | 11.09 | 2.8 x 10^-6^ | potassium ion transport | voltage-gated potassium channel complex |
| 207826_s_at | ID3 | inhibitor of DNA binding 3, dominant negative helix-loop-helix protein | 1.74 | 19.3 | 11.08 | 6.9 x 10^-3^ | negative regulation of transcription from RNA polymerase II promoter | nucleus |
| 222892_s_at | TMEM40 | transmembrane protein 40 | 5.30 | 58.7 | 11.08 | 5.7 x 10^-9^ |  | membrane |
| 223832_s_at | CAPNS2 | calpain, small subunit 2 | 2.35 | 26.0 | 11.06 | 3.2 x 10^-7^ |  | cytoplasm |
| 226750_at | LARP2 | La ribonucleoprotein domain family, member 2 | 2.40 | 26.5 | 11.03 | 1.4 x 10^-6^ |  |  |
| 235272_at | SBSN | suprabasin | 0.34 | 3.8 | 11.03 | 8.3 x 10^-3^ |  | extracellular region |
| 235706_at | CPM | carboxypeptidase M | 0.61 | 6.8 | 11.02 | 1.4 x 10^-5^ | proteolysis | plasma membrane |
| 212481_s_at | TPM4 | tropomyosin 4 | 3.15 | 34.7 | 11.02 | 1.5 x 10^-6^ | cell motion | cytoplasm |
| 209053_s_at | WHSC1 | Wolf-Hirschhorn syndrome candidate 1 | 1.42 | 15.7 | 11.02 | 4.9 x 10^-6^ | transcription | nucleus |
| 237563_s_at | LOC440731 | similar to hCG1817424 | 1.38 | 15.1 | 10.99 | 2.2 x 10^-8^ |  |  |
| 228284_at | TLE1 | transducin-like enhancer of split 1 (E(sp1) homolog, Drosophila) | 1.68 | 18.4 | 10.95 | 2.0 x 10^-11^ | transcription | nucleus |
| 201250_s_at | SLC2A1 | solute carrier family 2 (facilitated glucose transporter), member 1 | 6.90 | 75.5 | 10.94 | 2.0 x 10^-6^ | transport | intracellular |
| 219366_at | AVEN | apoptosis, caspase activation inhibitor | 2.35 | 25.7 | 10.94 | 3.4 x 10^-4^ | apoptosis | intracellular |
| 234985_at | LDLRAD3 | low density lipoprotein receptor class A domain containing 3 | 1.22 | 13.3 | 10.93 | 9.2 x 10^-11^ |  | membrane |
| 222409_at | CORO1C | coronin, actin binding protein, 1C | 4.73 | 51.7 | 10.93 | 9.1 x 10^-6^ | phagocytosis | actin cytoskeleton |
| 225387_at | TSPAN5 | tetraspanin 5 | 0.92 | 10.1 | 10.91 | 4.9 x 10^-4^ |  | membrane |
| 230398_at | TNS4 | tensin 4 | 2.57 | 28.1 | 10.91 | 9.3 x 10^-6^ | apoptosis | cytoplasm |
| 226881_at | GRPEL2 | GrpE-like 2, mitochondrial (E. coli) | 3.22 | 35.0 | 10.88 | 8.8 x 10^-7^ | protein folding | mitochondrion |
| 205797_s_at | TCP11L1 | t-complex 11 (mouse)-like 1 | 0.30 | 3.3 | 10.87 | 8.9 x 10^-8^ |  | cytoplasm |
| 1555355_a_at | ETS1 | v-ets erythroblastosis virus E26 oncogene homolog 1 (avian) | 1.21 | 13.2 | 10.86 | 7.3 x 10^-3^ | transcription | nucleus |
| 201540_at | FHL1 | four and a half LIM domains 1 | 5.14 | 55.9 | 10.86 | 1.9 x 10^-4^ | multicellular organismal development | nucleus |
| 1555786_s_at | C14orf34 | chromosome 14 open reading frame 34 | 2.91 | 31.6 | 10.83 | 1.7 x 10^-4^ |  |  |
| 212887_at | SEC23A | Sec23 homolog A (S. cerevisiae) | 5.75 | 62.3 | 10.83 | 2.8 x 10^-7^ | transport | Golgi membrane |
| 206033_s_at | DSC3 | desmocollin 3 | 3.39 | 36.7 | 10.82 | 2.0 x 10^-7^ | cell adhesion | membrane fraction |
| 1554131_at | KIAA1128 | KIAA1128 | 0.05 | 0.6 | 10.82 | 4.5 x 10^-3^ |  |  |
| 201560_at | CLIC4 | chlorideintracellular channel 4 | 9.74 | 105.2 | 10.81 | 2.7 x 10^-7^ | transport | intracellular |
| 235205_at | LOC100128259 /// LOC346887 | hypothetical protein LOC100128259 /// similar to solute carrier family 16 (monocarboxylic acid transporters), member 14 | 0.73 | 7.9 | 10.81 | 1.2 x 10^-4^ |  |  |
| 1554576_a_at | ETV4 | ets variant 4 | 0.32 | 3.5 | 10.81 | 8.1 x 10^-5^ | transcription | nucleus |
| 217025_s_at | DBN1 | drebrin 1 | 0.86 | 9.3 | 10.78 | 8.9 x 10^-7^ | actin filament organization | intracellular |
| 218451_at | CDCP1 | CUB domain containing protein 1 | 4.85 | 52.2 | 10.76 | 6.1 x 10^-9^ |  | extracellular region |
| 205460_at | NPAS2 | neuronal PAS domain protein 2 | 0.43 | 4.7 | 10.74 | 1.2 x 10^-3^ | transcription | nucleus |
| 230361_at | HEATR7A | HEAT repeat containing 7A | 0.83 | 8.9 | 10.72 | 9.7 x 10^-4^ |  |  |
| 1555841_at | C9orf30 | Chromosome 9 open reading frame 30 | 2.62 | 28.1 | 10.72 | 1.1 x 10^-9^ | multicellular organismal development | plasma membrane |
| 210372_s_at | TPD52L1 | tumor protein D52-like 1 | 3.02 | 32.3 | 10.71 | 7.5 x 10^-5^ | G2 | cytoplasm |
| 1553678_a_at | ITGB1 | integrin, beta 1 (fibronectin receptor, beta polypeptide, antigen CD29 includes MDF2, MSK12) | 20.52 | 219.7 | 10.71 | 8.4 x 10^-8^ | G1 | ruffle |
| 216268_s_at | JAG1 | jagged 1 (Alagille syndrome) | 23.30 | 249.3 | 10.70 | 4.0 x 10^-7^ | angiogenesis | extracellular region |
| 204971_at | CSTA | cystatin A (stefin A) | 35.56 | 380.5 | 10.70 | 9.1 x 10^-9^ | negative regulation of peptidase activity | cornified envelope |
| 203323_at | CAV2 | caveolin 2 | 8.22 | 87.7 | 10.68 | 3.7 x 10^-5^ | neuron migration | Golgi membrane |
| 221127_s_at | DKK3 | dickkopf homolog 3 (Xenopus laevis) | 0.36 | 3.9 | 10.68 | 2.3 x 10^-4^ | multicellular organismal development | extracellular region |
| 203535_at | S100A9 | S100 calcium binding protein A9 | 8.31 | 88.7 | 10.67 | 2.9 x 10^-5^ | inflammatory response | nucleus |
| 218817_at | SPCS3 | signal peptidase complex subunit 3 homolog (S. cerevisiae) | 2.24 | 23.9 | 10.67 | 8.0 x 10^-8^ | signal peptide processing | endoplasmic reticulum |
| 209444_at | RAP1GDS1 | RAP1, GTP-GDP dissociation stimulator 1 | 4.22 | 45.0 | 10.67 | 7.9 x 10^-10^ |  |  |
| 231183_s_at | JAG1 | Jagged 1 (Alagille syndrome) | 2.18 | 23.2 | 10.66 | 5.0 x 10^-8^ | angiogenesis | extracellular region |
| 205618_at | PRRG1 | proline rich Gla (G-carboxyglutamic acid) 1 | 0.84 | 8.9 | 10.66 | 2.4 x 10^-10^ |  | extracellular region |
| 228846_at | MXD1 | MAX dimerization protein 1 | 3.33 | 35.4 | 10.65 | 2.6 x 10^-6^ | transcription | nucleus |
| 239719_at | CD109 | CD109 molecule | 0.39 | 4.2 | 10.65 | 3.9 x 10^-4^ |  | extracellular region |
| 208581_x_at | MT1X | metallothionein 1X | 43.35 | 461.1 | 10.64 | 1.1 x 10^-6^ | response to metal ion |  |
| 225582_at | ITPRIP | inositol 1,4,5-triphosphate receptor interacting protein | 1.64 | 17.4 | 10.62 | 7.7 x 10^-12^ |  | plasma membrane |
| 1554262_s_at | KLHL29 | kelch-like 29 (Drosophila) | 0.17 | 1.8 | 10.60 | 2.8 x 10^-8^ |  |  |
| 204257_at | FADS3 | fatty acid desaturase 3 | 1.28 | 13.5 | 10.57 | 2.7 x 10^-6^ | lipid metabolic process | membrane fraction |
| 200600_at | MSN | moesin | 8.31 | 87.2 | 10.49 | 2.6 x 10^-5^ | cell motion | uropod |
| 1555136_at | FGD6 | FYVE, RhoGEF and PH domain containing 6 | 0.28 | 2.9 | 10.48 | 2.0 x 10^-5^ | cytoskeleton organization | ruffle |
| 204944_at | PTPRG | protein tyrosine phosphatase, receptor type, G | 0.60 | 6.3 | 10.47 | 7.2 x 10^-9^ | protein amino acid dephosphorylation | integral to plasma membrane |
| 207302_at | SGCG | sarcoglycan, gamma (35kDa dystrophin-associated glycoprotein) | 0.06 | 0.7 | 10.46 | 2.3 x 10^-4^ | cytoskeleton organization | cytoplasm |
| 227239_at | FAM126A | family with sequence similarity 126, member A | 1.70 | 17.8 | 10.44 | 1.4 x 10^-6^ |  | cytoplasm |
| 230356_at | --- | --- | 0.50 | 5.2 | 10.44 | 1.7 x 10^-5^ |  |  |
| 218499_at | RP6-213H19.1 | serine/threonine protein kinase MST4 | 4.04 | 42.1 | 10.43 | 1.5 x 10^-8^ | protein amino acid phosphorylation | cytoplasm |
| 1553938_a_at | STK32A | serine/threonine kinase 32A | 0.31 | 3.2 | 10.42 | 5.4 x 10^-4^ | protein amino acid phosphorylation |  |
| 217094_s_at | ITCH | itchy E3 ubiquitin protein ligase homolog (mouse) | 2.21 | 23.0 | 10.40 | 7.3 x 10^-5^ | regulation of cell growth | intracellular |
| 218849_s_at | PPP1R13L | protein phosphatase 1, regulatory (inhibitor) subunit 13 like | 2.59 | 26.9 | 10.40 | 8.5 x 10^-9^ | transcription | nucleus |
| 214452_at | BCAT1 | branched chain aminotransferase 1, cytosolic | 0.18 | 1.9 | 10.40 | 1.2 x 10^-4^ | G1 | cytoplasm |
| 224841_x_at | GAS5 | growth arrest-specific 5 (non-protein coding) | 26.63 | 276.4 | 10.38 | 5.4 x 10^-7^ |  |  |
| 1554997_a_at | PTGS2 | prostaglandin-endoperoxide synthase 2 (prostaglandin G/H synthase and cyclooxygenase) | 1.21 | 12.5 | 10.37 | 8.1 x 10^-4^ | prostaglandin biosynthetic process | nucleus |
| 201300_s_at | PRNP | prion protein | 30.56 | 316.9 | 10.37 | 1.5 x 10^-8^ | cellular copper ion homeostasis | cytoplasm |
| 217998_at | LOC652993 /// PHLDA1 | hypothetical LOC652993 /// pleckstrin homology-like domain, family A, member 1 | 0.69 | 7.1 | 10.36 | 3.1 x 10^-6^ | apoptosis | nucleus |
| 224511_s_at | TXNDC17 | thioredoxin domain containing 17 | 17.34 | 179.7 | 10.36 | 7.9 x 10^-7^ | tumor necrosis factor-mediated signaling pathway | cytoplasm |
| 205082_s_at | AOX1 | aldehyde oxidase 1 | 0.21 | 2.1 | 10.34 | 4.4 x 10^-5^ | oxygen and reactive oxygen species metabolic process | cytoplasm |
| 202557_at | HSPA13 | heat shock protein 70kDa family, member 13 | 6.32 | 65.3 | 10.33 | 1.1 x 10^-7^ |  | endoplasmic reticulum |
| 209387_s_at | TM4SF1 | transmembrane 4 L six family member 1 | 4.00 | 41.2 | 10.30 | 7.4 x 10^-7^ |  | integral to plasma membrane |
| 203585_at | ZNF185 | zinc finger protein 185 (LIM domain) | 8.67 | 89.2 | 10.29 | 5.8 x 10^-10^ |  | nucleus |
| 212185_x_at | MT2A | metallothionein 2A | 55.76 | 573.5 | 10.28 | 2.3 x 10^-7^ | cellular copper ion homeostasis |  |
| 202934_at | HK2 | hexokinase 2 | 4.02 | 41.4 | 10.28 | 2.9 x 10^-5^ | carbohydrate metabolic process | mitochondrion |
| 226835_s_at | C20orf199 | chromosome 20 open reading frame 199 | 26.76 | 274.9 | 10.27 | 2.2 x 10^-6^ |  |  |
| 201149_s_at | TIMP3 | TIMP metallopeptidase inhibitor 3 | 1.30 | 13.4 | 10.25 | 4.1 x 10^-4^ | transmembrane receptor protein tyrosine kinase signaling pathway | extracellular region |
| 203476_at | TPBG | trophoblast glycoprotein | 25.64 | 262.4 | 10.23 | 3.3 x 10^-7^ | cell adhesion | cytoplasm |
| 203725_at | GADD45A | growth arrest and DNA-damage-inducible, alpha | 13.25 | 135.4 | 10.22 | 4.4 x 10^-10^ | regulation of cyclin-dependent protein kinase activity | nucleus |
| 203735_x_at | PPFIBP1 | PTPRF interacting protein, binding protein 1 (liprin beta 1) | 1.17 | 12.0 | 10.22 | 1.1 x 10^-6^ | cell adhesion | cytoplasm |
| 201984_s_at | EGFR | epidermal growth factor receptor (erythroblastic leukemia viral (v-erb-b) oncogene homolog, avian) | 9.17 | 93.6 | 10.20 | 9.1 x 10^-7^ | ossification | extracellular region |
| 221268_s_at | SGPP1 | sphingosine-1-phosphate phosphatase 1 | 1.28 | 13.0 | 10.17 | 3.0 x 10^-7^ | sphingolipid metabolic process | membrane fraction |
| 205830_at | CLGN | calmegin | 5.72 | 58.2 | 10.17 | 8.1 x 10^-3^ | protein folding | endoplasmic reticulum |
| 213094_at | GPR126 | G protein-coupled receptor 126 | 1.48 | 15.0 | 10.16 | 3.0 x 10^-6^ | signal transduction | plasma membrane |
| 218484_at | NDUFA4L2 | NADH dehydrogenase (ubiquinone) 1 alpha subcomplex, 4-like 2 | 0.38 | 3.8 | 10.14 | 6.4 x 10^-3^ |  |  |
| 1554807_a_at | SPIRE1 | spire homolog 1 (Drosophila) | 1.76 | 17.8 | 10.14 | 1.1 x 10^-6^ | transport | cytoplasm |
| 201559_s_at | CLIC4 | chlorideintracellular channel 4 | 2.67 | 27.0 | 10.13 | 3.3 x 10^-5^ | transport | intracellular |
| 235834_at | CALD1 | Caldesmon 1 | 0.17 | 1.7 | 10.06 | 1.5 x 10^-6^ | cell motion | membrane fraction |
| 210654_at | TNFRSF10D | tumor necrosis factor receptor superfamily, member 10d, decoy with truncated death domain | 0.31 | 3.1 | 10.05 | 1.5 x 10^-5^ | apoptosis | membrane |
| 209744_x_at | ITCH | itchy E3 ubiquitin protein ligase homolog (mouse) | 2.59 | 26.0 | 10.04 | 2.8 x 10^-5^ | regulation of cell growth | intracellular |
| 224252_s_at | FXYD5 | FXYD domain containing ion transport regulator 5 | 2.91 | 29.2 | 10.03 | 2.8 x 10^-8^ | transport | membrane |
| 217755_at | HN1 | hematological and neurological expressed 1 | 8.33 | 83.5 | 10.02 | 1.2 x 10^-7^ |  | nucleus |
| 229778_at | C12orf39 | chromosome 12 open reading frame 39 | 0.27 | 2.7 | 10.02 | 6.6 x 10^-7^ |  | extracellular region |
| 221577_x_at | GDF15 | growth differentiation factor 15 | 13.56 | 135.2 | 9.97 | 4.0 x 10^-3^ | signal transduction | extracellular region |
| 225806_at | JUB | jub, ajuba homolog (Xenopus laevis) | 7.90 | 78.7 | 9.96 | 3.8 x 10^-5^ | cell cycle | nucleus |
| 215489_x_at | HOMER3 | homer homolog 3 (Drosophila) | 1.28 | 12.7 | 9.95 | 1.8 x 10^-4^ | protein targeting | cytoplasm |
| 228205_at | TKT | transketolase | 0.59 | 5.9 | 9.95 | 1.9 x 10^-6^ | metabolic process | cytosol |
| 233478_at | --- | --- | 0.39 | 3.9 | 9.94 | 1.1 x 10^-5^ |  |  |
| 1554097_a_at | LOC554202 | hypothetical LOC554202 | 1.59 | 15.8 | 9.94 | 3.1 x 10^-3^ |  |  |
| 213201_s_at | TNNT1 | troponin T type 1 (skeletal, slow) | 0.45 | 4.5 | 9.94 | 1.3 x 10^-4^ | skeletal muscle contraction | troponin complex |
| 205402_x_at | PRSS2 | protease, serine, 2 (trypsin 2) | 0.28 | 2.7 | 9.94 | 4.7 x 10^-6^ | proteolysis | extracellular region |
| 1557938_s_at | PTRF | polymerase I and transcript release factor | 1.58 | 15.7 | 9.93 | 1.3 x 10^-4^ | transcription | nucleus |
| 224013_s_at | SOX7 | SRY (sex determining region Y)-box 7 | 0.75 | 7.5 | 9.91 | 1.5 x 10^-4^ | transcription | nucleus |
| 227107_at | --- | --- | 2.87 | 28.4 | 9.90 | 5.4 x 10^-9^ |  |  |
| 201850_at | CAPG | capping protein (actin filament), gelsolin-like | 6.31 | 62.3 | 9.87 | 1.6 x 10^-7^ | protein complex assembly | nucleus |
| 204490_s_at | CD44 | CD44 molecule (Indian blood group) | 5.89 | 58.2 | 9.87 | 1.4 x 10^-8^ | regulation of cell growth | cytoplasm |
| 223151_at | DCUN1D5 | DCN1, defective in cullin neddylation 1, domain containing 5 (S. cerevisiae) | 8.27 | 81.4 | 9.85 | 1.0 x 10^-8^ |  |  |
| 221050_s_at | GTPBP2 | GTP binding protein 2 | 2.92 | 28.8 | 9.85 | 4.9 x 10^-5^ |  |  |
| 224790_at | ASAP1 | ArfGAP with SH3 domain, ankyrin repeat and PH domain 1 | 0.97 | 9.5 | 9.85 | 2.5 x 10^-9^ | regulation of ARF GTPase activity | cytoplasm |
| 224915_x_at | C20orf199 | chromosome 20 open reading frame 199 | 33.60 | 330.3 | 9.83 | 4.9 x 10^-8^ |  |  |
| 229644_at | PREP | Prolyl endopeptidase | 0.98 | 9.6 | 9.81 | 2.5 x 10^-5^ | proteolysis | cytoplasm |
| 209099_x_at | JAG1 | jagged 1 (Alagille syndrome) | 27.09 | 264.8 | 9.77 | 2.1 x 10^-6^ | angiogenesis | extracellular region |
| 212014_x_at | CD44 | CD44 molecule (Indian blood group) | 7.66 | 74.8 | 9.77 | 1.2 x 10^-7^ | regulation of cell growth | cytoplasm |
| 224767_at | RPL37 | Ribosomal protein L37 | 2.47 | 24.1 | 9.76 | 1.1 x 10^-5^ | translation | intracellular |
| 201631_s_at | IER3 | immediate early response 3 | 43.57 | 424.8 | 9.75 | 6.3 x 10^-9^ | apoptosis | caveola |
| 225195_at | DPH3 | DPH3, KTI11 homolog (S. cerevisiae) | 4.38 | 42.7 | 9.74 | 2.6 x 10^-5^ | peptidyl-diphthamide biosynthetic process from peptidyl-histidine | nucleus |
| 219045_at | RHOF | ras homolog gene family, member F (in filopodia) | 1.61 | 15.7 | 9.74 | 5.9 x 10^-7^ | actin filament organization | intracellular |
| 205899_at | CCNA1 | cyclin A1 | 4.40 | 42.8 | 9.73 | 6.2 x 10^-3^ | cell cycle | nucleus |
| 203543_s_at | KLF9 | Kruppel-like factor 9 | 2.46 | 23.9 | 9.70 | 2.6 x 10^-3^ | transcription | intracellular |
| 232995_at | --- | --- | 0.26 | 2.5 | 9.68 | 8.7 x 10^-8^ |  |  |
| 225314_at | OCIAD2 | OCIA domain containing 2 | 18.11 | 175.0 | 9.66 | 3.5 x 10^-10^ |  | mitochondrion |
| 231766_s_at | COL12A1 | collagen, type XII, alpha 1 | 0.42 | 4.0 | 9.65 | 2.9 x 10^-4^ | skeletal system development | extracellular region |
| 1554464_a_at | CRTAP | cartilage associated protein | 2.98 | 28.7 | 9.65 | 9.7 x 10^-10^ |  | extracellular region |
| 204990_s_at | ITGB4 | integrin, beta 4 | 11.03 | 106.2 | 9.63 | 3.0 x 10^-7^ | cell communication | integrin complex |
| 204326_x_at | MT1X | metallothionein 1X | 35.73 | 343.7 | 9.62 | 1.5 x 10^-7^ | response to metal ion |  |
| 219987_at | --- | --- | 0.22 | 2.1 | 9.59 | 6.4 x 10^-4^ | transposition | membrane |
| 216383_at | RPL18A /// RPL18AP3 /// RPL18AP6 | ribosomal protein L18a /// ribosomal protein L18a pseudogene 3 /// ribosomal protein L18a pseudogene 6 | 1.55 | 14.8 | 9.58 | 9.1 x 10^-6^ | translation | intracellular |
| 212160_at | XPOT | exportin, tRNA (nuclear export receptor for tRNAs) | 16.34 | 156.4 | 9.57 | 7.7 x 10^-9^ | tRNA export from nucleus | nucleus |
| 215198_s_at | CALD1 | caldesmon 1 | 0.23 | 2.2 | 9.55 | 1.6 x 10^-5^ | cell motion | membrane fraction |
| 208790_s_at | PTRF | polymerase I and transcript release factor | 0.85 | 8.1 | 9.54 | 3.1 x 10^-4^ | transcription | nucleus |
| 218871_x_at | CSGALNACT2 | chondroitin sulfate N-acetylgalactosaminyltransferase 2 | 3.35 | 31.9 | 9.52 | 1.4 x 10^-7^ | proteoglycan biosynthetic process | Golgi apparatus |
| 220214_at | ZNF215 | zinc finger protein 215 | 0.31 | 2.9 | 9.49 | 5.2 x 10^-4^ | transcription | intracellular |
| 226408_at | TEAD2 | TEA domain family member 2 | 0.32 | 3.1 | 9.48 | 1.2 x 10^-5^ | transcription | nucleus |
| 223452_s_at | ATL3 | atlastin GTPase 3 | 1.58 | 14.9 | 9.48 | 7.6 x 10^-7^ |  | membrane |
| 223519_at | ZAK | sterile alpha motif and leucine zipper containing kinase AZK | 2.98 | 28.2 | 9.47 | 3.4 x 10^-8^ | cell cycle checkpoint | nucleus |
| 226227_x_at | C20orf199 | chromosome 20 open reading frame 199 | 37.73 | 357.3 | 9.47 | 1.2 x 10^-7^ |  |  |
| 232132_at | PARD6G | par-6 partitioning defective 6 homolog gamma (C. elegans) | 0.16 | 1.5 | 9.47 | 8.6 x 10^-6^ | cell cycle | cytoplasm |
| 209264_s_at | TSPAN4 | tetraspanin 4 | 1.00 | 9.4 | 9.46 | 1.3 x 10^-4^ | protein complex assembly | plasma membrane |
| 204977_at | DDX10 | DEAD (Asp-Glu-Ala-Asp) box polypeptide 10 | 4.76 | 45.0 | 9.45 | 1.5 x 10^-3^ |  | nucleolus |
| 1568813_at | --- | --- | 0.24 | 2.2 | 9.45 | 3.0 x 10^-4^ |  |  |
| 221854_at | PKP1 | plakophilin 1 (ectodermal dysplasia/skin fragility syndrome) | 2.22 | 21.0 | 9.44 | 2.2 x 10^-5^ | cell adhesion | nucleus |
| 230930_at | LOC338620 | hypothetical protein LOC338620 | 0.55 | 5.2 | 9.44 | 1.6 x 10^-4^ |  |  |
| 217457_s_at | RAP1GDS1 | RAP1, GTP-GDP dissociation stimulator 1 | 2.17 | 20.5 | 9.44 | 3.6 x 10^-10^ |  |  |
| 218498_s_at | ERO1L | ERO1-like (S. cerevisiae) | 4.28 | 40.4 | 9.44 | 2.8 x 10^-6^ | protein folding | membrane fraction |
| 211651_s_at | LAMB1 | laminin, beta 1 | 1.72 | 16.2 | 9.43 | 7.8 x 10^-5^ | cell adhesion | extracellular region |
| 221779_at | MICALL1 | MICAL-like 1 | 3.41 | 32.1 | 9.42 | 4.8 x 10^-7^ |  | cytoplasm |
| 56197_at | PLSCR3 | phospholipid scramblase 3 | 5.38 | 50.7 | 9.42 | 1.5 x 10^-7^ | phospholipid scrambling | plasma membrane |
| 243805_at | CCBE1 | collagen and calcium binding EGF domains 1 | 0.22 | 2.1 | 9.41 | 3.1 x 10^-7^ |  | extracellular region |
| 213734_at | WSB2 | WD repeat and SOCS box-containing 2 | 4.60 | 43.3 | 9.41 | 5.1 x 10^-6^ | intracellular signaling cascade |  |
| 211456_x_at | MT1P2 | metallothionein 1 pseudogene 2 | 38.61 | 363.4 | 9.41 | 4.9 x 10^-5^ |  |  |
| 222309_at | C6orf62 | Chromosome 6 open reading frame 62 | 0.63 | 5.9 | 9.39 | 5.1 x 10^-4^ |  | intracellular |
| 235707_at | LOC221710 | hypothetical protein LOC221710 | 0.50 | 4.7 | 9.39 | 7.6 x 10^-6^ | regulation of transcription |  |
| 202381_at | ADAM9 | ADAM metallopeptidase domain 9 (meltrin gamma) | 25.59 | 240.0 | 9.38 | 3.5 x 10^-9^ | activation of MAPKK activity | extracellular region |
| 203968_s_at | CDC6 | cell division cycle 6 homolog (S. cerevisiae) | 0.38 | 3.6 | 9.38 | 7.5 x 10^-3^ | DNA replication checkpoint | nucleus |
| 202431_s_at | MYC | v-myc myelocytomatosis viral oncogene homolog (avian) | 9.62 | 90.1 | 9.37 | 8.1 x 10^-5^ | B cell apoptosis | nucleus |
| 1558683_a_at | HMGA2 | high mobility group AT-hook 2 | 0.32 | 3.0 | 9.35 | 4.3 x 10^-6^ | establishment or maintenance of chromatin architecture | nuclear chromosome |
| 216283_s_at | PVR | poliovirus receptor | 0.33 | 3.1 | 9.35 | 6.8 x 10^-6^ | positive regulation of natural killer cell mediated cytotoxicity directed against tumor cell target | extracellular region |
| 234971_x_at | PLCD3 | phospholipase C, delta 3 | 0.58 | 5.4 | 9.34 | 2.2 x 10^-8^ | angiogenesis | cytoplasm |
| 224468_s_at | C19orf48 | chromosome 19 open reading frame 48 | 1.16 | 10.8 | 9.33 | 5.6 x 10^-4^ |  |  |
| 210026_s_at | CARD10 | caspase recruitment domain family, member 10 | 2.84 | 26.5 | 9.33 | 1.1 x 10^-3^ | protein complex assembly | intracellular |
| 201853_s_at | CDC25B | cell division cycle 25 homolog B (S. pombe) | 2.32 | 21.6 | 9.33 | 5.2 x 10^-6^ | M phase of mitotic cell cycle | intracellular |
| 221269_s_at | SH3BGRL3 | SH3 domain binding glutamic acid-rich protein like 3 | 12.59 | 117.3 | 9.32 | 2.5 x 10^-7^ | cell redox homeostasis | nucleus |
| 216652_s_at | DR1 | down-regulator of transcription 1, TBP-binding (negative cofactor 2) | 3.03 | 28.2 | 9.31 | 1.1 x 10^-6^ | negative regulation of transcription from RNA polymerase II promoter | intracellular |
| 210008_s_at | MRPS12 | mitochondrial ribosomal protein S12 | 0.24 | 2.2 | 9.30 | 1.1 x 10^-6^ | translation | intracellular |
| 214073_at | CTTN | cortactin | 1.40 | 13.0 | 9.30 | 1.2 x 10^-6^ | receptor-mediated endocytosis | ruffle |
| 222411_s_at | SSR3 | signal sequence receptor, gamma (translocon-associated protein gamma) | 15.33 | 142.4 | 9.29 | 1.1 x 10^-10^ | cotranslational protein targeting to membrane | endoplasmic reticulum |
| 201286_at | SDC1 | syndecan 1 | 11.95 | 110.9 | 9.28 | 9.5 x 10^-8^ |  | integral to plasma membrane |
| 227249_at | NDE1 | NudE nuclear distribution gene E homolog 1 (A. nidulans) | 3.19 | 29.6 | 9.27 | 4.8 x 10^-11^ | cell cycle | kinetochore |
| 228234_at | TICAM2 | toll-like receptor adaptor molecule 2 | 1.89 | 17.5 | 9.27 | 3.4 x 10^-7^ | regulation of cytokine production | cytoplasm |
| 1567224_at | HMGA2 | high mobility group AT-hook 2 | 0.74 | 6.9 | 9.26 | 2.2 x 10^-3^ | establishment or maintenance of chromatin architecture | nuclear chromosome |
| 226421_at | AMMECR1 | Alport syndrome, mental retardation, midface hypoplasia and elliptocytosis chromosomal region gene 1 | 2.98 | 27.6 | 9.26 | 8.7 x 10^-6^ |  |  |
| 234675_x_at | --- | --- | 9.18 | 84.5 | 9.20 | 5.3 x 10^-6^ |  |  |
| 238654_at | LOC147645 | hypothetical protein LOC147645 | 0.23 | 2.1 | 9.18 | 3.0 x 10^-4^ |  |  |
| 201469_s_at | SHC1 | SHC (Src homology 2 domain containing) transforming protein 1 | 2.40 | 22.1 | 9.18 | 4.2 x 10^-6^ | MAPKKK cascade | cytoplasm |
| 208095_s_at | SRP72 | signal recognition particle 72kDa | 14.66 | 134.4 | 9.17 | 1.8 x 10^-7^ | protein amino acid phosphorylation | nucleolus |
| 204005_s_at | PAWR | PRKC, apoptosis, WT1, regulator | 5.23 | 47.8 | 9.15 | 3.1 x 10^-6^ | negative regulation of transcription from RNA polymerase II promoter | nucleus |
| 239809_at | --- | --- | 0.53 | 4.8 | 9.15 | 2.4 x 10^-4^ |  |  |
| 220227_at | CDH4 | cadherin 4, type 1, R-cadherin (retinal) | 0.37 | 3.4 | 9.14 | 6.1 x 10^-6^ | cell adhesion | plasma membrane |
| 215034_s_at | TM4SF1 | transmembrane 4 L six family member 1 | 5.45 | 49.8 | 9.13 | 2.7 x 10^-6^ |  | integral to plasma membrane |
| 222728_s_at | TAF1D | TATA box binding protein (TBP)-associated factor, RNA polymerase I, D, 41kDa | 7.72 | 70.4 | 9.12 | 5.2 x 10^-6^ | transcription | nucleus |
| 229518_at | FAM46B | family with sequence similarity 46, member B | 1.27 | 11.6 | 9.12 | 5.9 x 10^-7^ |  |  |
| 202671_s_at | PDXK | pyridoxal (pyridoxine, vitamin B6) kinase | 8.47 | 77.1 | 9.10 | 2.5 x 10^-7^ | cell proliferation | cytoplasm |
| 221676_s_at | CORO1C | coronin, actin binding protein, 1C | 7.22 | 65.7 | 9.10 | 5.6 x 10^-6^ | phagocytosis | actin cytoskeleton |
| 223666_at | SNX5 | sorting nexin 5 | 0.65 | 6.0 | 9.09 | 2.3 x 10^-3^ | transport |  |
| 209098_s_at | JAG1 | jagged 1 (Alagille syndrome) | 3.75 | 34.1 | 9.09 | 2.1 x 10^-5^ | angiogenesis | extracellular region |
| 241353_s_at | LOC100129105 | similar to hCG1821214 | 2.25 | 20.4 | 9.08 | 5.9 x 10^-8^ |  |  |
| 206877_at | MXD1 | MAX dimerization protein 1 | 0.27 | 2.4 | 9.08 | 4.5 x 10^-5^ | transcription | nucleus |
| 238332_at | ANKRD29 | ankyrin repeat domain 29 | 1.30 | 11.8 | 9.08 | 1.5 x 10^-3^ |  |  |
| 201215_at | PLS3 | plastin 3 (T isoform) | 24.47 | 221.7 | 9.06 | 9.9 x 10^-7^ | actin filament organization | cytoplasm |
| 221291_at | ULBP2 | UL16 binding protein 2 | 0.67 | 6.1 | 9.05 | 1.9 x 10^-5^ | immune response | extracellular region |
| 223789_s_at | GTPBP2 | GTP binding protein 2 | 0.68 | 6.1 | 9.04 | 7.4 x 10^-6^ |  |  |
| 205567_at | CHST1 | carbohydrate (keratan sulfate Gal-6) sulfotransferase 1 | 0.49 | 4.4 | 9.04 | 6.3 x 10^-4^ | carbohydrate metabolic process | Golgi membrane |
| 204435_at | NUPL1 | nucleoporin like 1 | 4.13 | 37.3 | 9.03 | 6.9 x 10^-4^ | transport | nucleus |
| 227072_at | RTTN | rotatin | 4.94 | 44.6 | 9.03 | 6.3 x 10^-4^ | multicellular organismal development |  |
| 227558_at | CBX4 | chromobox homolog 4 (Pc class homolog, Drosophila) | 17.13 | 154.6 | 9.03 | 1.1 x 10^-5^ | negative regulation of transcription from RNA polymerase II promoter | chromatin |
| 231428_at | --- | --- | 0.20 | 1.8 | 9.02 | 4.5 x 10^-4^ |  |  |
| 205138_s_at | UST | uronyl-2-sulfotransferase | 0.60 | 5.4 | 9.00 | 1.3 x 10^-3^ | protein amino acid sulfation | Golgi membrane |
| 1553105_s_at | DSG2 | desmoglein 2 | 7.11 | 64.0 | 9.00 | 5.0 x 10^-7^ | cell adhesion | plasma membrane |
| 226282_at | --- | --- | 5.32 | 47.9 | 9.00 | 6.4 x 10^-10^ |  |  |
| 229927_at | LEMD1 | LEM domain containing 1 | 0.37 | 3.4 | 9.00 | 4.6 x 10^-5^ |  | nuclear envelope |
| 210233_at | IL1RAP | interleukin 1 receptor accessory protein | 0.59 | 5.3 | 8.98 | 4.1 x 10^-6^ | protein complex assembly | extracellular region |
| 201069_at | MMP2 | matrix metallopeptidase 2 (gelatinase A, 72kDa gelatinase, 72kDa type IV collagenase) | 0.77 | 6.9 | 8.98 | 2.5 x 10^-3^ | response to hypoxia | extracellular region |
| 204034_at | ETHE1 | ethylmalonic encephalopathy 1 | 6.29 | 56.4 | 8.97 | 4.6 x 10^-8^ |  | nucleus |
| 205493_s_at | DPYSL4 | dihydropyrimidinase-like 4 | 0.20 | 1.8 | 8.96 | 6.6 x 10^-3^ | nervous system development | cytoplasm |
| 209410_s_at | GRB10 | growth factor receptor-bound protein 10 | 1.36 | 12.1 | 8.96 | 1.1 x 10^-7^ | signal transduction | cytoplasm |
| 204286_s_at | PMAIP1 | phorbol-12-myristate-13-acetate-induced protein 1 | 4.88 | 43.7 | 8.96 | 5.7 x 10^-3^ | release of cytochrome c from mitochondria | mitochondrion |
| 219390_at | FKBP14 | FK506 binding protein 14, 22 kDa | 2.21 | 19.8 | 8.95 | 2.2 x 10^-7^ | protein folding | endoplasmic reticulum |
| 212048_s_at | YARS | tyrosyl-tRNA synthetase | 8.45 | 75.6 | 8.94 | 1.9 x 10^-9^ | translation | extracellular space |
| 205525_at | CALD1 | caldesmon 1 | 0.36 | 3.2 | 8.94 | 1.7 x 10^-8^ | cell motion | membrane fraction |
| 204527_at | MYO5A | myosin VA (heavy chain 12, myoxin) | 2.27 | 20.3 | 8.93 | 7.0 x 10^-6^ | ubiquitin-dependent protein catabolic process | ruffle |
| 201587_s_at | IRAK1 | interleukin-1 receptor-associated kinase 1 | 10.62 | 94.8 | 8.93 | 3.7 x 10^-7^ | regulation of cytokine-mediated signaling pathway | cytosol |
| 228107_at | LOC100127983 | hypothetical protein LOC100127983 | 0.42 | 3.8 | 8.89 | 1.4 x 10^-5^ |  |  |
| 210074_at | CTSL2 | cathepsin L2 | 2.62 | 23.3 | 8.88 | 7.8 x 10^-4^ | proteolysis | lysosome |
| 209863_s_at | TP63 | tumor protein p63 | 14.89 | 132.2 | 8.88 | 6.2 x 10^-6^ | response to tumor cell | nucleus |
| 235534_at | --- | --- | 1.15 | 10.2 | 8.87 | 2.5 x 10^-4^ |  |  |
| 226452_at | PDK1 | pyruvate dehydrogenase kinase, isozyme 1 | 1.74 | 15.5 | 8.87 | 1.0 x 10^-5^ | carbohydrate metabolic process | mitochondrion |
| 230348_at | LATS2 | LATS, large tumor suppressor, homolog 2 (Drosophila) | 0.17 | 1.5 | 8.86 | 3.3 x 10^-4^ | G1 | spindle pole |
| 231881_at | CALD1 | caldesmon 1 | 0.11 | 0.9 | 8.86 | 4.1 x 10^-6^ | cell motion | membrane fraction |
| 240353_s_at | C12orf54 | chromosome 12 open reading frame 54 | 0.46 | 4.1 | 8.85 | 4.9 x 10^-3^ |  |  |
| 1554539_a_at | RHOF | ras homolog gene family, member F (in filopodia) | 0.75 | 6.6 | 8.84 | 3.7 x 10^-9^ | actin filament organization | intracellular |
| 211043_s_at | CLTB | clathrin, light chain (Lcb) | 6.96 | 61.5 | 8.83 | 9.0 x 10^-5^ | intracellular protein transport | coated pit |
| 239431_at | TICAM2 | toll-like receptor adaptor molecule 2 | 0.55 | 4.8 | 8.83 | 9.7 x 10^-7^ | regulation of cytokine production | cytoplasm |
| 235295_at | --- | --- | 1.01 | 8.9 | 8.82 | 2.9 x 10^-8^ |  |  |
| 213419_at | APBB2 | amyloid beta (A4) precursor protein-binding, family B, member 2 | 0.91 | 8.0 | 8.80 | 5.9 x 10^-7^ | neuron migration | nucleus |
| 219522_at | FJX1 | four jointed box 1 (Drosophila) | 1.71 | 15.0 | 8.77 | 6.9 x 10^-4^ |  | extracellular region |
| 219256_s_at | SH3TC1 | SH3 domain and tetratricopeptide repeats 1 | 0.89 | 7.8 | 8.76 | 2.4 x 10^-4^ |  |  |
| 222235_s_at | CSGALNACT2 | chondroitin sulfate N-acetylgalactosaminyltransferase 2 | 4.08 | 35.6 | 8.74 | 1.8 x 10^-8^ | proteoglycan biosynthetic process | Golgi apparatus |
| 226538_at | MAN2A1 | mannosidase, alpha, class 2A, member 1 | 8.48 | 73.8 | 8.70 | 9.5 x 10^-7^ | in utero embryonic development | Golgi membrane |
| 209758_s_at | MFAP5 | microfibrillar associated protein 5 | 0.36 | 3.2 | 8.70 | 3.7 x 10^-4^ |  | microfibril |
| 205123_s_at | TMEFF1 | transmembrane protein with EGF-like and two follistatin-like domains 1 | 0.17 | 1.5 | 8.69 | 4.6 x 10^-7^ | multicellular organismal development | plasma membrane |
| 219936_s_at | GPR87 | G protein-coupled receptor 87 | 5.46 | 47.4 | 8.68 | 2.1 x 10^-8^ | signal transduction | plasma membrane |
| 227209_at | CNTN1 | Contactin 1 | 0.84 | 7.3 | 8.65 | 2.4 x 10^-3^ | cell adhesion | membrane fraction |
| 200999_s_at | CKAP4 | cytoskeleton-associated protein 4 | 12.26 | 106.0 | 8.65 | 2.8 x 10^-7^ |  | membrane fraction |
| 224654_at | DDX21 | DEAD (Asp-Glu-Ala-Asp) box polypeptide 21 | 17.37 | 150.2 | 8.65 | 5.8 x 10^-9^ | transcription | intracellular |
| 214435_x_at | RALA | v-ral simian leukemia viral oncogene homolog A (ras related) | 5.55 | 47.9 | 8.64 | 1.3 x 10^-6^ | chemotaxis | intracellular |
| 210705_s_at | TRIM5 | tripartite motif-containing 5 | 1.49 | 12.8 | 8.64 | 1.2 x 10^-5^ | response to virus | intracellular |
| 226769_at | FIBIN | fin bud initiation factor homolog (zebrafish) | 1.31 | 11.3 | 8.62 | 4.5 x 10^-4^ |  | extracellular region |
| 242828_at | FIGN | fidgetin | 0.45 | 3.9 | 8.62 | 4.3 x 10^-4^ |  |  |
| 216806_at | --- | --- | 1.20 | 10.4 | 8.62 | 1.3 x 10^-5^ |  |  |
| 233085_s_at | OBFC2A | oligonucleotide/oligosaccharide-binding fold containing 2A | 1.79 | 15.4 | 8.62 | 3.7 x 10^-7^ |  | nucleus |
| 206247_at | MICB | MHC class I polypeptide-related sequence B | 1.28 | 11.0 | 8.62 | 7.7 x 10^-3^ | immune response-activating cell surface receptor signaling pathway | plasma membrane |
| 230630_at | AK3L1 | Adenylate kinase 3-like 1 | 0.65 | 5.6 | 8.62 | 6.4 x 10^-9^ | nucleobase, nucleoside, nucleotide and nucleic acid metabolic process | mitochondrion |
| 200989_at | HIF1A | hypoxia inducible factor 1, alpha subunit (basic helix-loop-helix transcription factor) | 30.02 | 258.6 | 8.61 | 8.1 x 10^-10^ | angiogenesis | nucleus |
| 231402_at | LOC100129105 | similar to hCG1821214 | 0.17 | 1.4 | 8.61 | 3.5 x 10^-5^ |  |  |
| 201820_at | KRT5 | keratin 5 | 44.51 | 382.6 | 8.60 | 1.6 x 10^-5^ | epidermis development | intermediate filament |
| 225258_at | FBLIM1 | filamin binding LIM protein 1 | 4.97 | 42.6 | 8.58 | 3.9 x 10^-7^ | cell adhesion | cytoplasm |
| 234973_at | SLC38A5 | solute carrier family 38, member 5 | 0.77 | 6.6 | 8.57 | 2.1 x 10^-6^ |  | plasma membrane |
| 206034_at | SERPINB8 | serpin peptidase inhibitor, clade B (ovalbumin), member 8 | 1.93 | 16.5 | 8.57 | 2.5 x 10^-5^ |  | extracellular region |
| 201666_at | TIMP1 | TIMP metallopeptidase inhibitor 1 | 25.15 | 215.4 | 8.56 | 1.8 x 10^-6^ | cell activation | extracellular region |
| 219648_at | MREG | melanoregulin | 2.76 | 23.6 | 8.56 | 2.0 x 10^-8^ | melanocyte differentiation | plasma membrane |
| 213895_at | EMP1 | epithelial membrane protein 1 | 0.44 | 3.8 | 8.55 | 8.4 x 10^-3^ | multicellular organismal development | membrane fraction |
| 236718_at | MYO10 | myosin X | 0.38 | 3.3 | 8.55 | 1.8 x 10^-3^ | signal transduction | cytoskeleton |
| 225665_at | ZAK | sterile alpha motif and leucine zipper containing kinase AZK | 4.95 | 42.3 | 8.54 | 2.6 x 10^-9^ | cell cycle checkpoint | nucleus |
| 213923_at | RAP2B | RAP2B, member of RAS oncogene family | 6.55 | 55.8 | 8.52 | 2.7 x 10^-7^ | signal transduction | intracellular |
| 230503_at | --- | --- | 0.71 | 6.0 | 8.52 | 1.1 x 10^-4^ |  |  |
| 1558014_s_at | FAR1 | fatty acyl CoA reductase 1 | 1.96 | 16.7 | 8.51 | 5.5 x 10^-5^ | lipid metabolic process | peroxisome |
| 231928_at | HES2 | hairy and enhancer of split 2 (Drosophila) | 1.55 | 13.2 | 8.51 | 1.4 x 10^-10^ | transcription | nucleus |
| 201287_s_at | SDC1 | syndecan 1 | 19.76 | 168.2 | 8.51 | 1.6 x 10^-7^ |  | integral to plasma membrane |
| 209344_at | TPM4 | tropomyosin 4 | 2.95 | 25.1 | 8.51 | 1.6 x 10^-4^ | cell motion | cytoplasm |
| 220484_at | MCOLN3 | mucolipin 3 | 0.33 | 2.8 | 8.50 | 1.1 x 10^-4^ | transport | membrane |
| 212473_s_at | MICAL2 | microtubule associated monoxygenase, calponin and LIM domain containing 2 | 5.58 | 47.5 | 8.50 | 5.3 x 10^-4^ | metabolic process | cytoplasm |
| 203851_at | IGFBP6 | insulin-like growth factor binding protein 6 | 1.63 | 13.8 | 8.50 | 1.7 x 10^-4^ | regulation of cell growth | extracellular region |
| 211824_x_at | NLRP1 | NLR family, pyrin domain containing 1 | 0.62 | 5.2 | 8.50 | 8.9 x 10^-4^ | apoptosis | intracellular |
| 224840_at | FKBP5 | FK506 binding protein 5 | 7.96 | 67.6 | 8.50 | 3.5 x 10^-5^ | protein folding | nucleus |
| 239273_s_at | MMP28 | matrix metallopeptidase 28 | 0.87 | 7.4 | 8.49 | 3.4 x 10^-8^ | proteolysis | extracellular region |
| 204136_at | COL7A1 | collagen, type VII, alpha 1 | 3.84 | 32.6 | 8.48 | 6.6 x 10^-6^ | cell adhesion | extracellular region |
| 207173_x_at | CDH11 | cadherin 11, type 2, OB-cadherin (osteoblast) | 2.73 | 23.1 | 8.47 | 1.8 x 10^-5^ | skeletal system development | cytoplasm |
| 213012_at | NEDD4 | neural precursor cell expressed, developmentally down-regulated 4 | 1.34 | 11.4 | 8.47 | 8.8 x 10^-8^ | protein modification process | ubiquitin ligase complex |
| 202620_s_at | PLOD2 | procollagen-lysine, 2-oxoglutarate 5-dioxygenase 2 | 4.90 | 41.5 | 8.46 | 1.4 x 10^-6^ | response to hypoxia | endoplasmic reticulum |
| 205393_s_at | CHEK1 | CHK1 checkpoint homolog (S. pombe) | 0.50 | 4.3 | 8.45 | 8.8 x 10^-4^ | DNA damage checkpoint | condensed nuclear chromosome |
| 225252_at | SRXN1 | sulfiredoxin 1 homolog (S. cerevisiae) | 5.21 | 43.9 | 8.43 | 1.8 x 10^-8^ | transcription | intracellular |
| 229555_at | GALNT5 | UDP-N-acetyl-alpha-D-galactosamine:polypeptide N-acetylgalactosaminyltransferase 5 (GalNAc-T5) | 3.40 | 28.7 | 8.43 | 2.5 x 10^-5^ | glycosaminoglycan biosynthetic process | Golgi membrane |
| 213166_x_at | FAM128A | family with sequence similarity 128, member A | 7.99 | 67.2 | 8.42 | 1.4 x 10^-9^ |  |  |
| 209792_s_at | KLK10 | kallikrein-related peptidase 10 | 9.20 | 77.4 | 8.42 | 3.8 x 10^-5^ | proteolysis | extracellular region |
| 209835_x_at | CD44 | CD44 molecule (Indian blood group) | 9.85 | 82.9 | 8.42 | 2.6 x 10^-7^ | regulation of cell growth | cytoplasm |
| 1557257_at | BCL10 | B-cell CLL/lymphoma 10 | 0.77 | 6.5 | 8.39 | 3.0 x 10^-3^ | neural tube closure | immunological synapse |
| 238741_at | FAM83A | family with sequence similarity 83, member A | 1.47 | 12.3 | 8.38 | 8.0 x 10^-5^ |  |  |
| 224791_at | ASAP1 | ArfGAP with SH3 domain, ankyrin repeat and PH domain 1 | 3.22 | 26.9 | 8.37 | 7.9 x 10^-9^ | regulation of ARF GTPase activity | cytoplasm |
| 206080_at | PLCH2 | phospholipase C, eta 2 | 0.49 | 4.1 | 8.36 | 5.2 x 10^-6^ | lipid metabolic process | cytoplasm |
| 228565_at | KIAA1804 | mixed lineage kinase 4 | 1.14 | 9.6 | 8.36 | 2.9 x 10^-8^ | protein amino acid phosphorylation | cytoplasm |
| 212245_at | MCFD2 | multiple coagulation factor deficiency 2 | 9.83 | 82.1 | 8.35 | 1.1 x 10^-5^ | transport | extracellular region |
| 200872_at | S100A10 | S100 calcium binding protein A10 | 50.44 | 420.8 | 8.34 | 1.8 x 10^-6^ | signal transduction | mitochondrion |
| 211527_x_at | VEGFA | vascular endothelial growth factor A | 4.22 | 35.1 | 8.32 | 1.6 x 10^-5^ | angiogenesis | extracellular region |
| 200808_s_at | ZYX | zyxin | 3.15 | 26.2 | 8.32 | 2.6 x 10^-6^ | cell adhesion | stress fiber |
| 204489_s_at | CD44 | CD44 molecule (Indian blood group) | 7.80 | 64.8 | 8.31 | 1.7 x 10^-7^ | regulation of cell growth | cytoplasm |
| 202701_at | BMP1 | bone morphogenetic protein 1 | 1.16 | 9.6 | 8.29 | 1.3 x 10^-6^ | skeletal system development | extracellular space |
| 235019_at | CPM | carboxypeptidase M | 0.73 | 6.0 | 8.28 | 5.3 x 10^-6^ | proteolysis | plasma membrane |
| 207357_s_at | GALNT10 | UDP-N-acetyl-alpha-D-galactosamine:polypeptide N-acetylgalactosaminyltransferase 10 (GalNAc-T10) | 0.52 | 4.3 | 8.28 | 6.4 x 10^-6^ | protein amino acid O-linked glycosylation | Golgi membrane |
| 226621_at | --- | --- | 6.59 | 54.5 | 8.27 | 4.1 x 10^-8^ |  |  |
| 226535_at | ITGB6 | integrin, beta 6 | 5.40 | 44.6 | 8.26 | 6.3 x 10^-3^ | inflammatory response | integrin complex |
| 210286_s_at | SLC4A7 | solute carrier family 4, sodium bicarbonate cotransporter, member 7 | 0.85 | 7.0 | 8.26 | 9.1 x 10^-6^ | transport | plasma membrane |
| 1553764_a_at | JUB | jub, ajuba homolog (Xenopus laevis) | 4.77 | 39.4 | 8.26 | 2.2 x 10^-5^ | cell cycle | nucleus |
| 1554966_a_at | FILIP1L | filamin A interacting protein 1-like | 0.46 | 3.8 | 8.24 | 1.1 x 10^-4^ |  | nucleus |
| 227070_at | GLT8D2 | glycosyltransferase 8 domain containing 2 | 0.16 | 1.3 | 8.24 | 7.5 x 10^-3^ |  | membrane |
| 202347_s_at | UBE2K | ubiquitin-conjugating enzyme E2K (UBC1 homolog, yeast) | 12.02 | 99.0 | 8.24 | 6.5 x 10^-7^ | ubiquitin-dependent protein catabolic process | cytoplasm |
| 202779_s_at | LOC731049 /// UBE2S | similar to Ubiquitin-conjugating enzyme E2S (Ubiquitin-conjugating enzyme E2-24 kDa) (Ubiquitin-protein ligase) (Ubiquitin carrier protein) (E2-EPF5) /// ubiquitin-conjugating enzyme E2S | 3.25 | 26.8 | 8.23 | 6.4 x 10^-4^ | protein modification process |  |
| 213671_s_at | MARS | methionyl-tRNA synthetase | 13.20 | 108.3 | 8.20 | 2.4 x 10^-8^ | translation | cytoplasm |
| 215707_s_at | PRNP | prion protein | 8.06 | 66.0 | 8.20 | 1.0 x 10^-5^ | cellular copper ion homeostasis | cytoplasm |
| 217553_at | MGC87042 | similar to Six transmembrane epithelial antigen of prostate | 0.19 | 1.6 | 8.19 | 8.6 x 10^-5^ |  | membrane |
| 201397_at | PHGDH | phosphoglycerate dehydrogenase | 4.08 | 33.4 | 8.19 | 2.3 x 10^-9^ | L-serine biosynthetic process |  |
| 207714_s_at | SERPINH1 | serpin peptidase inhibitor, clade H (heat shock protein 47), member 1, (collagen binding protein 1) | 2.27 | 18.5 | 8.18 | 1.6 x 10^-4^ | response to stress | endoplasmic reticulum |
| 215706_x_at | ZYX | zyxin | 2.82 | 23.1 | 8.18 | 4.3 x 10^-5^ | cell adhesion | stress fiber |
| 219431_at | ARHGAP10 | Rho GTPase activating protein 10 | 0.60 | 4.9 | 8.17 | 6.3 x 10^-5^ | cytoskeleton organization | intracellular |
| 64942_at | GPR153 | G protein-coupled receptor 153 | 1.15 | 9.4 | 8.17 | 1.0 x 10^-6^ | signal transduction | plasma membrane |
| 228033_at | E2F7 | E2F transcription factor 7 | 2.54 | 20.7 | 8.16 | 1.2 x 10^-4^ | transcription | nucleus |
| 244533_at | --- | --- | 2.51 | 20.4 | 8.16 | 1.9 x 10^-6^ |  |  |
| 219892_at | TM6SF1 | transmembrane 6 superfamily member 1 | 0.89 | 7.3 | 8.15 | 2.5 x 10^-4^ |  | membrane |
| 216641_s_at | LAD1 | ladinin 1 | 2.10 | 17.1 | 8.14 | 9.9 x 10^-6^ |  | extracellular region |
| 203074_at | ANXA8 /// ANXA8L1 /// ANXA8L2 | annexin A8 /// annexin A8-like 1 /// annexin A8-like 2 | 16.89 | 137.6 | 8.14 | 7.4 x 10^-3^ | blood coagulation |  |
| 222937_s_at | MMP28 | matrix metallopeptidase 28 | 0.34 | 2.8 | 8.14 | 1.1 x 10^-3^ | proteolysis | extracellular region |
| 224741_x_at | GAS5 | growth arrest-specific 5 (non-protein coding) | 33.51 | 272.0 | 8.12 | 4.6 x 10^-7^ |  |  |
| 216470_x_at | PRSS1 | protease, serine, 1 (trypsin 1) | 0.35 | 2.8 | 8.12 | 1.5 x 10^-8^ | proteolysis | extracellular region |
| 218336_at | PFDN2 | prefoldin subunit 2 | 12.51 | 101.4 | 8.11 | 1.0 x 10^-8^ | protein folding | prefoldin complex |
| 203693_s_at | E2F3 | E2F transcription factor 3 | 1.04 | 8.5 | 8.09 | 4.6 x 10^-10^ | transcription | nucleus |
| 1560066_at | --- | --- | 0.95 | 7.7 | 8.09 | 1.7 x 10^-4^ |  |  |
| 225541_at | RPL22L1 | ribosomal protein L22-like 1 | 15.80 | 127.8 | 8.09 | 8.2 x 10^-6^ | translation | intracellular |
| 205016_at | TGFA | transforming growth factor, alpha | 7.38 | 59.7 | 8.09 | 4.5 x 10^-11^ | activation of MAPK activity | extracellular region |
| 219483_s_at | PORCN | porcupine homolog (Drosophila) | 2.04 | 16.5 | 8.08 | 1.3 x 10^-7^ | glycoprotein metabolic process | endoplasmic reticulum |
| 1552575_a_at | C6orf141 | chromosome 6 open reading frame 141 | 0.64 | 5.2 | 8.08 | 4.7 x 10^-6^ |  |  |
| 221489_s_at | SPRY4 | sprouty homolog 4 (Drosophila) | 0.55 | 4.4 | 8.07 | 1.7 x 10^-4^ | multicellular organismal development | cytoplasm |
| 243515_at | --- | --- | 0.97 | 7.8 | 8.06 | 9.4 x 10^-5^ |  |  |
| 1558305_at | GIGYF2 | GRB10 interacting GYF protein 2 | 0.55 | 4.4 | 8.06 | 1.9 x 10^-4^ |  |  |
| 1554283_at | CCRN4L | CCR4 carbon catabolite repression 4-like (S. cerevisiae) | 0.41 | 3.3 | 8.05 | 1.7 x 10^-10^ | transcription from RNA polymerase II promoter | nucleus |
| 231293_at | --- | --- | 0.08 | 0.6 | 8.04 | 4.5 x 10^-4^ |  |  |
| 238759_at | CCDC88A | coiled-coil domain containing 88A | 0.35 | 2.8 | 8.03 | 4.0 x 10^-6^ | regulation of protein amino acid phosphorylation | cytoplasm |
| 228559_at | CENPN | centromere protein N | 0.97 | 7.8 | 8.03 | 5.2 x 10^-3^ |  | chromosome, centromeric region |
| 1570402_at | KLC3 | kinesin light chain 3 | 0.62 | 5.0 | 8.02 | 6.5 x 10^-5^ |  | cytoplasm |
| 219099_at | C12orf5 | chromosome 12 open reading frame 5 | 3.81 | 30.5 | 8.01 | 3.6 x 10^-8^ | metabolic process | intracellular |
| 219959_at | MOCOS | molybdenum cofactor sulfurase | 7.00 | 56.0 | 8.00 | 6.4 x 10^-6^ | Mo-molybdopterin cofactor biosynthetic process |  |
| 201016_at | EIF1AX | eukaryotic translation initiation factor 1A, X-linked | 5.67 | 45.4 | 7.99 | 5.5 x 10^-6^ | translation | cytoplasm |
| 202543_s_at | GMFB | glia maturation factor, beta | 5.12 | 40.9 | 7.99 | 1.3 x 10^-6^ | protein amino acid phosphorylation | intracellular |
| 236346_at | --- | --- | 2.66 | 21.2 | 7.99 | 2.4 x 10^-4^ |  |  |
| 236429_at | ZNF83 | zinc finger protein 83 | 1.26 | 10.0 | 7.99 | 1.4 x 10^-3^ | transcription | intracellular |
| 222705_s_at | SLC25A15 | solute carrier family 25 (mitochondrial carrier; ornithine transporter) member 15 | 0.15 | 1.2 | 7.98 | 2.3 x 10^-4^ | urea cycle | mitochondrion |
| 1570482_at | --- | --- | 0.30 | 2.4 | 7.98 | 5.8 x 10^-6^ |  |  |
| 226968_at | KIF1B | kinesin family member 1B | 2.34 | 18.6 | 7.96 | 1.2 x 10^-6^ | microtubule-based movement | cytoplasm |
| 204745_x_at | MT1G | metallothionein 1G | 15.19 | 120.8 | 7.95 | 5.7 x 10^-7^ |  |  |
| 202696_at | OXSR1 | oxidative-stress responsive 1 | 5.32 | 42.3 | 7.95 | 5.9 x 10^-7^ | protein amino acid phosphorylation | cytoplasm |
| 220789_s_at | TBRG4 | transforming growth factor beta regulator 4 | 1.32 | 10.5 | 7.95 | 9.0 x 10^-5^ | G1 phase of mitotic cell cycle | mitochondrion |
| 203939_at | NT5E | 5'-nucleotidase, ecto (CD73) | 8.53 | 67.6 | 7.93 | 5.0 x 10^-4^ | purine nucleotide biosynthetic process | membrane fraction |
| 216705_s_at | ADA | adenosine deaminase | 0.50 | 3.9 | 7.93 | 2.1 x 10^-3^ | response to hypoxia | cytoplasm |
| 202201_at | BLVRB | biliverdin reductase B (flavin reductase (NADPH)) | 17.71 | 140.3 | 7.92 | 2.2 x 10^-6^ | metabolic process | cytoplasm |
| 211980_at | COL4A1 | collagen, type IV, alpha 1 | 0.54 | 4.3 | 7.92 | 3.6 x 10^-3^ |  | extracellular region |
| 201760_s_at | WSB2 | WD repeat and SOCS box-containing 2 | 13.50 | 106.7 | 7.91 | 2.4 x 10^-8^ | intracellular signaling cascade |  |
| 242944_at | FAM83A | family with sequence similarity 83, member A | 0.54 | 4.3 | 7.91 | 2.2 x 10^-3^ |  |  |
| 221039_s_at | ASAP1 | ArfGAP with SH3 domain, ankyrin repeat and PH domain 1 | 1.24 | 9.8 | 7.90 | 1.5 x 10^-9^ | regulation of ARF GTPase activity | cytoplasm |
| 214297_at | CSPG4 | chondroitin sulfate proteoglycan 4 | 0.22 | 1.7 | 7.90 | 7.2 x 10^-3^ | angiogenesis | plasma membrane |
| 209884_s_at | SLC4A7 | solute carrier family 4, sodium bicarbonate cotransporter, member 7 | 3.41 | 26.9 | 7.89 | 2.1 x 10^-5^ | transport | plasma membrane |
| 218656_s_at | LHFP | lipoma HMGIC fusion partner | 0.55 | 4.3 | 7.88 | 2.6 x 10^-6^ |  | membrane |
| 201416_at | SOX4 | SRY (sex determining region Y)-box 4 | 8.02 | 63.2 | 7.88 | 4.4 x 10^-3^ | pro-B cell differentiation | nucleus |
| 237159_x_at | AP1S3 | adaptor-related protein complex 1, sigma 3 subunit | 1.34 | 10.6 | 7.88 | 2.7 x 10^-7^ | transport | Golgi apparatus |
| 229228_at | CREB5 | cAMP responsive element binding protein 5 | 0.43 | 3.4 | 7.88 | 1.1 x 10^-4^ | transcription | intracellular |
| 40148_at | APBB2 | amyloid beta (A4) precursor protein-binding, family B, member 2 | 0.86 | 6.8 | 7.88 | 1.6 x 10^-7^ | neuron migration | nucleus |
| 227193_at | --- | --- | 0.51 | 4.0 | 7.86 | 1.6 x 10^-7^ |  |  |
| 1553581_s_at | SFRS12IP1 | SFRS12-interacting protein 1 | 6.65 | 52.3 | 7.85 | 3.3 x 10^-7^ | mRNA processing |  |
| 227897_at | --- | --- | 9.95 | 78.1 | 7.85 | 4.1 x 10^-5^ |  |  |
| 219225_at | LOC100134440 /// PGBD5 | similar to PGBD5 protein /// piggyBac transposable element derived 5 | 0.26 | 2.1 | 7.83 | 1.9 x 10^-5^ |  | membrane |
| 202755_s_at | GPC1 | glypican 1 | 0.93 | 7.3 | 7.83 | 4.7 x 10^-5^ |  | extracellular region |
| 203180_at | ALDH1A3 | aldehyde dehydrogenase 1 family, member A3 | 21.76 | 170.2 | 7.82 | 6.5 x 10^-9^ | optic cup morphogenesis involved in camera-type eye development | cytoplasm |
| 210299_s_at | FHL1 | four and a half LIM domains 1 | 1.36 | 10.6 | 7.82 | 2.3 x 10^-3^ | multicellular organismal development | nucleus |
| 225807_at | JUB | jub, ajuba homolog (Xenopus laevis) | 1.30 | 10.1 | 7.82 | 3.9 x 10^-5^ | cell cycle | nucleus |
| 213937_s_at | FTSJ1 | FtsJ homolog 1 (E. coli) | 2.36 | 18.5 | 7.81 | 8.4 x 10^-9^ | rRNA processing |  |
| 223196_s_at | SESN2 | sestrin 2 | 2.12 | 16.6 | 7.81 | 2.1 x 10^-5^ | cell cycle arrest | nucleus |
| 209263_x_at | TSPAN4 | tetraspanin 4 | 2.52 | 19.6 | 7.80 | 1.1 x 10^-6^ | protein complex assembly | plasma membrane |
| 227769_at | --- | --- | 1.87 | 14.6 | 7.78 | 5.7 x 10^-4^ |  |  |
| 206571_s_at | MAP4K4 | mitogen-activated protein kinase kinase kinase kinase 4 | 3.41 | 26.5 | 7.78 | 1.3 x 10^-6^ | protein amino acid phosphorylation |  |
| 206421_s_at | SERPINB7 | serpin peptidase inhibitor, clade B (ovalbumin), member 7 | 5.88 | 45.7 | 7.78 | 1.5 x 10^-6^ |  | cytoplasm |
| 223225_s_at | SEH1L | SEH1-like (S. cerevisiae) | 1.35 | 10.5 | 7.77 | 5.3 x 10^-8^ | transport | nucleus |
| 1555889_a_at | CRTAP | cartilage associated protein | 10.54 | 81.9 | 7.77 | 3.7 x 10^-8^ |  | extracellular region |
| 241813_at | MBD1 | methyl-CpG binding domain protein 1 | 0.96 | 7.5 | 7.77 | 1.3 x 10^-7^ | transcription | nucleus |
| 203258_at | DRAP1 | DR1-associated protein 1 (negative cofactor 2 alpha) | 3.63 | 28.2 | 7.77 | 1.0 x 10^-7^ | negative regulation of transcription from RNA polymerase II promoter | intracellular |
| 227202_at | CNTN1 | Contactin 1 | 0.18 | 1.4 | 7.76 | 1.0 x 10^-3^ | cell adhesion | membrane fraction |
| 206969_at | KRT34 | keratin 34 | 0.08 | 0.6 | 7.76 | 4.7 x 10^-3^ | epidermis development | intermediate filament |
| 1569150_x_at | PDLIM7 | PDZ and LIM domain 7 (enigma) | 0.24 | 1.9 | 7.75 | 2.5 x 10^-3^ | ossification | cytoplasm |
| 1555480_a_at | FBLIM1 | filamin binding LIM protein 1 | 1.69 | 13.1 | 7.75 | 2.4 x 10^-5^ | cell adhesion | cytoplasm |
| 243591_at | --- | --- | 0.89 | 6.9 | 7.74 | 5.8 x 10^-8^ |  |  |
| 239959_x_at | --- | --- | 0.28 | 2.1 | 7.73 | 3.2 x 10^-6^ |  |  |
| 226241_s_at | MRPL52 | mitochondrial ribosomal protein L52 | 4.99 | 38.5 | 7.72 | 9.6 x 10^-7^ | translation | mitochondrion |
| 229404_at | TWIST2 | twist homolog 2 (Drosophila) | 0.47 | 3.7 | 7.72 | 4.0 x 10^-3^ | transcription | nucleus |
| 203108_at | GPRC5A | G protein-coupled receptor, family C, group 5, member A | 8.63 | 66.6 | 7.72 | 1.5 x 10^-5^ | signal transduction | endoplasmic reticulum |
| 208613_s_at | FLNB | filamin B, beta (actin binding protein 278) | 13.41 | 103.5 | 7.72 | 6.9 x 10^-6^ | cytoskeletal anchoring at plasma membrane | nucleus |
| 219930_at | KLF8 | Kruppel-like factor 8 | 0.21 | 1.6 | 7.71 | 2.2 x 10^-7^ | transcription | intracellular |
| 222231_s_at | LRRC59 | leucine rich repeat containing 59 | 18.62 | 143.6 | 7.71 | 3.1 x 10^-7^ |  | endoplasmic reticulum |
| 206284_x_at | CLTB | clathrin, light chain (Lcb) | 6.38 | 49.2 | 7.71 | 5.5 x 10^-4^ | intracellular protein transport | coated pit |
| 210017_at | MALT1 | mucosa associated lymphoid tissue lymphoma translocation gene 1 | 1.53 | 11.8 | 7.71 | 7.7 x 10^-7^ | positive regulation of T cell cytokine production | nucleus |
| 228977_at | LOC729680 | hypothetical protein LOC729680 | 1.24 | 9.5 | 7.69 | 7.7 x 10^-7^ |  |  |
| 244350_at | MYO10 | myosin X | 0.48 | 3.7 | 7.68 | 3.3 x 10^-4^ | signal transduction | cytoskeleton |
| 242321_at | --- | --- | 1.16 | 8.9 | 7.68 | 2.5 x 10^-6^ |  |  |
| 35820_at | GM2A | GM2 ganglioside activator | 6.45 | 49.5 | 7.68 | 7.3 x 10^-9^ | ganglioside metabolic process | cytoplasm |
| 220362_at | PSORS1C1 | psoriasis susceptibility 1 candidate 1 | 0.25 | 1.9 | 7.68 | 1.5 x 10^-4^ |  |  |
| 209589_s_at | EPHB2 | EPH receptor B2 | 0.78 | 6.0 | 7.67 | 2.5 x 10^-4^ | protein amino acid phosphorylation | membrane |
| 204422_s_at | FGF2 | fibroblast growth factor 2 (basic) | 0.23 | 1.8 | 7.66 | 2.7 x 10^-3^ | activation of MAPKK activity | extracellular region |
| 213992_at | COL4A6 | collagen, type IV, alpha 6 | 5.14 | 39.3 | 7.65 | 6.6 x 10^-8^ | cell adhesion | extracellular region |
| 231396_s_at | FAM126A | family with sequence similarity 126, member A | 0.28 | 2.1 | 7.63 | 3.0 x 10^-5^ |  | cytoplasm |
| 218205_s_at | MKNK2 | MAP kinase interacting serine/threonine kinase 2 | 55.83 | 425.8 | 7.63 | 6.5 x 10^-6^ | regulation of translation |  |
| 209409_at | GRB10 | growth factor receptor-bound protein 10 | 3.40 | 25.9 | 7.62 | 3.4 x 10^-8^ | signal transduction | cytoplasm |
| 218611_at | IER5 | immediate early response 5 | 11.92 | 90.7 | 7.61 | 1.6 x 10^-6^ |  |  |
| 216532_x_at | LOC728344 | similar to hCG1978918 | 2.96 | 22.5 | 7.61 | 3.0 x 10^-5^ |  |  |
| 1552256_a_at | SCARB1 | scavenger receptor class B, member 1 | 1.49 | 11.3 | 7.61 | 4.9 x 10^-4^ | transport | plasma membrane |
| 214880_x_at | CALD1 | caldesmon 1 | 0.54 | 4.1 | 7.61 | 3.6 x 10^-6^ | cell motion | membrane fraction |
| 208230_s_at | NRG1 | neuregulin 1 | 0.37 | 2.8 | 7.61 | 3.9 x 10^-6^ | cell communication | extracellular region |
| 215728_s_at | ACOT7 | acyl-CoA thioesterase 7 | 1.23 | 9.4 | 7.60 | 1.9 x 10^-6^ | lipid metabolic process | cytoplasm |
| 211354_s_at | LEPR | leptin receptor | 0.59 | 4.5 | 7.60 | 1.2 x 10^-5^ | angiogenesis | extracellular region |
| 223361_at | C6orf115 | chromosome 6 open reading frame 115 | 7.16 | 54.4 | 7.60 | 2.6 x 10^-6^ |  |  |
| 1555970_at | --- | --- | 0.64 | 4.9 | 7.59 | 8.2 x 10^-8^ |  |  |
| 212763_at | CAMSAP1L1 | calmodulin regulated spectrin-associated protein 1-like 1 | 1.81 | 13.7 | 7.59 | 4.6 x 10^-7^ |  |  |
| 212171_x_at | VEGFA | vascular endothelial growth factor A | 9.20 | 69.9 | 7.59 | 8.0 x 10^-7^ | angiogenesis | extracellular region |
| 209016_s_at | KRT7 | keratin 7 | 15.55 | 117.8 | 7.58 | 8.1 x 10^-7^ | DNA replication | cytoplasm |
| 215729_s_at | VGLL1 | vestigial like 1 (Drosophila) | 0.23 | 1.7 | 7.57 | 2.5 x 10^-4^ | transcription | nucleus |
| 212995_x_at | FAM128B | family with sequence similarity 128, member B | 10.86 | 82.1 | 7.56 | 1.9 x 10^-10^ | L-serine biosynthetic process |  |
| 212527_at | PPPDE2 | PPPDE peptidase domain containing 2 | 1.76 | 13.3 | 7.55 | 2.5 x 10^-7^ |  |  |
| 1555731_a_at | AP1S3 | adaptor-related protein complex 1, sigma 3 subunit | 3.61 | 27.3 | 7.55 | 1.5 x 10^-6^ | transport | Golgi apparatus |
| 221078_s_at | CCDC88A | coiled-coil domain containing 88A | 0.46 | 3.5 | 7.54 | 1.9 x 10^-7^ | regulation of protein amino acid phosphorylation | cytoplasm |
| 232092_at | MCART1 | mitochondrial carrier triple repeat 1 | 1.08 | 8.2 | 7.54 | 4.9 x 10^-5^ | transport | mitochondrion |
| 205122_at | TMEFF1 | transmembrane protein with EGF-like and two follistatin-like domains 1 | 0.40 | 3.0 | 7.54 | 1.2 x 10^-6^ | multicellular organismal development | plasma membrane |
| 217775_s_at | RDH11 | retinol dehydrogenase 11 (all-trans/9-cis/11-cis) | 3.53 | 26.6 | 7.53 | 6.9 x 10^-7^ | transport | intracellular |
| 242037_at | ASPH | Aspartate beta-hydroxylase | 0.30 | 2.3 | 7.53 | 8.4 x 10^-6^ | muscle contraction | endoplasmic reticulum |
| 1555326_a_at | ADAM9 | ADAM metallopeptidase domain 9 (meltrin gamma) | 5.32 | 40.0 | 7.53 | 1.3 x 10^-5^ | activation of MAPKK activity | extracellular region |
| 232254_at | --- | --- | 0.57 | 4.3 | 7.53 | 3.4 x 10^-6^ |  |  |
| 201387_s_at | UCHL1 | ubiquitin carboxyl-terminal esterase L1 (ubiquitin thiolesterase) | 1.10 | 8.3 | 7.52 | 3.7 x 10^-5^ | ubiquitin-dependent protein catabolic process | intracellular |
| 39402_at | IL1B | interleukin 1, beta | 2.37 | 17.8 | 7.51 | 3.9 x 10^-3^ | activation of MAPK activity | extracellular region |
| 221902_at | GPR153 | G protein-coupled receptor 153 | 0.85 | 6.4 | 7.51 | 1.1 x 10^-4^ | signal transduction | plasma membrane |
| 208637_x_at | ACTN1 | actinin, alpha 1 | 9.88 | 74.2 | 7.51 | 3.1 x 10^-4^ | regulation of apoptosis | nucleolus |
| 214444_s_at | PVR | poliovirus receptor | 0.44 | 3.3 | 7.50 | 3.0 x 10^-5^ | positive regulation of natural killer cell mediated cytotoxicity directed against tumor cell target | extracellular region |
| 201818_at | LPCAT1 | lysophosphatidylcholine acyltransferase 1 | 1.37 | 10.3 | 7.49 | 4.7 x 10^-7^ | metabolic process | Golgi membrane |
| 224793_s_at | TGFBR1 | transforming growth factor, beta receptor 1 | 7.27 | 54.4 | 7.49 | 7.4 x 10^-8^ | activation of MAPKK activity | plasma membrane |
| 224367_at | BEX2 | brain expressed X-linked 2 | 4.98 | 37.2 | 7.47 | 7.5 x 10^-4^ |  | nucleus |
| 205394_at | CHEK1 | CHK1 checkpoint homolog (S. pombe) | 0.68 | 5.1 | 7.45 | 9.8 x 10^-3^ | DNA damage checkpoint | condensed nuclear chromosome |
| 229801_at | C10orf47 | chromosome 10 open reading frame 47 | 6.14 | 45.7 | 7.45 | 4.9 x 10^-6^ |  |  |
| 204639_at | ADA | adenosine deaminase | 0.92 | 6.8 | 7.44 | 8.1 x 10^-5^ | response to hypoxia | cytoplasm |
| 209373_at | MALL | mal, T-cell differentiation protein-like | 6.89 | 51.2 | 7.44 | 3.5 x 10^-6^ | cholesterol homeostasis | Golgi membrane |
| 202619_s_at | PLOD2 | procollagen-lysine, 2-oxoglutarate 5-dioxygenase 2 | 3.50 | 26.0 | 7.43 | 4.1 x 10^-5^ | response to hypoxia | endoplasmic reticulum |
| 235198_at | OSTM1 | osteopetrosis associated transmembrane protein 1 | 2.78 | 20.6 | 7.42 | 2.3 x 10^-7^ | osteoclast differentiation | cytosol |
| 207675_x_at | ARTN | Artemin | 0.94 | 7.0 | 7.41 | 1.2 x 10^-4^ | signal transduction | extracellular region |
| 207382_at | TP63 | tumor protein p63 | 1.48 | 11.0 | 7.41 | 3.8 x 10^-7^ | response to tumor cell | nucleus |
| 238477_at | --- | --- | 2.00 | 14.8 | 7.41 | 6.6 x 10^-8^ |  |  |
| 219944_at | CLIP4 | CAP-GLY domain containing linker protein family, member 4 | 1.82 | 13.4 | 7.40 | 1.5 x 10^-4^ |  |  |
| 212094_at | PEG10 | paternally expressed 10 | 1.92 | 14.2 | 7.40 | 3.5 x 10^-3^ | proteolysis | nucleus |
| 209386_at | TM4SF1 | transmembrane 4 L six family member 1 | 14.80 | 109.5 | 7.40 | 2.4 x 10^-4^ |  | integral to plasma membrane |
| 225723_at | C6orf129 | chromosome 6 open reading frame 129 | 1.99 | 14.7 | 7.39 | 1.6 x 10^-5^ |  | membrane |
| 228618_at | PEAR1 | platelet endothelial aggregation receptor 1 | 0.39 | 2.8 | 7.39 | 9.1 x 10^-5^ |  | plasma membrane |
| 228499_at | PFKFB4 | 6-phosphofructo-2-kinase/fructose-2,6-biphosphatase 4 | 0.74 | 5.5 | 7.38 | 9.8 x 10^-5^ | fructose metabolic process | cytosol |
| 203736_s_at | PPFIBP1 | PTPRF interacting protein, binding protein 1 (liprin beta 1) | 0.45 | 3.3 | 7.37 | 3.0 x 10^-7^ | cell adhesion | cytoplasm |
| 208152_s_at | DDX21 | DEAD (Asp-Glu-Ala-Asp) box polypeptide 21 | 10.54 | 77.6 | 7.37 | 5.7 x 10^-9^ |  | nucleus |
| 219550_at | ROBO3 | roundabout, axon guidance receptor, homolog 3 (Drosophila) | 0.57 | 4.2 | 7.36 | 9.4 x 10^-6^ | chemotaxis | membrane |
| 204421_s_at | FGF2 | fibroblast growth factor 2 (basic) | 0.29 | 2.2 | 7.35 | 1.8 x 10^-3^ | activation of MAPKK activity | extracellular region |
| 210244_at | CAMP | cathelicidin antimicrobial peptide | 0.50 | 3.7 | 7.35 | 3.4 x 10^-5^ | defense response | extracellular region |
| 239233_at | CCDC88A | coiled-coil domain containing 88A | 0.29 | 2.2 | 7.35 | 1.2 x 10^-5^ | regulation of protein amino acid phosphorylation | cytoplasm |
| 212072_s_at | CSNK2A1 | casein kinase 2, alpha 1 polypeptide | 12.73 | 93.5 | 7.34 | 5.9 x 10^-7^ | regulation of cell growth | nucleus |
| 212263_at | QKI | quaking homolog, KH domain RNA binding (mouse) | 7.58 | 55.6 | 7.34 | 1.4 x 10^-6^ | mRNA processing | nucleus |
| 223658_at | KCNK6 | potassium channel, subfamily K, member 6 | 1.34 | 9.8 | 7.34 | 7.0 x 10^-8^ | transport | voltage-gated potassium channel complex |
| 200790_at | ODC1 | ornithine decarboxylase 1 | 32.39 | 237.4 | 7.33 | 1.7 x 10^-7^ | kidney development | nucleus |
| 220253_s_at | LRP12 | low density lipoprotein-related protein 12 | 1.17 | 8.5 | 7.33 | 2.1 x 10^-4^ | endocytosis | integral to plasma membrane |
| 208456_s_at | RRAS2 | related RAS viral (r-ras) oncogene homolog 2 | 6.01 | 44.0 | 7.32 | 5.9 x 10^-6^ | small GTPase mediated signal transduction | intracellular |
| 209283_at | CRYAB | crystallin, alpha B | 0.69 | 5.0 | 7.31 | 3.5 x 10^-5^ | protein folding | soluble fraction |
| 204135_at | FILIP1L | filamin A interacting protein 1-like | 0.83 | 6.1 | 7.31 | 1.9 x 10^-5^ |  | nucleus |
| 229442_at | C18orf54 | chromosome 18 open reading frame 54 | 0.60 | 4.4 | 7.31 | 6.9 x 10^-3^ |  | extracellular region |
| 213603_s_at | RAC2 | ras-related C3 botulinum toxin substrate 2 (rho family, small GTP binding protein Rac2) | 6.87 | 50.2 | 7.30 | 1.8 x 10^-4^ | chemotaxis | intracellular |
| 206857_s_at | FKBP1B | FK506 binding protein 1B, 12.6 kDa | 3.38 | 24.7 | 7.30 | 9.6 x 10^-10^ | protein folding | cytoplasm |
| 1558152_at | LOC100131262 | hypothetical LOC100131262 | 0.97 | 7.1 | 7.30 | 1.5 x 10^-4^ |  |  |
| 200634_at | PFN1 | profilin 1 | 33.01 | 240.8 | 7.29 | 5.1 x 10^-6^ | neural tube closure | nucleus |
| 203002_at | AMOTL2 | angiomotin like 2 | 4.58 | 33.4 | 7.29 | 3.5 x 10^-4^ |  | tight junction |
| 214431_at | GMPS | guanine monphosphate synthetase | 4.14 | 30.2 | 7.29 | 1.3 x 10^-6^ | purine nucleotide biosynthetic process | cytoplasm |
| 202147_s_at | IFRD1 | interferon-related developmental regulator 1 | 3.91 | 28.4 | 7.28 | 2.9 x 10^-4^ | multicellular organismal development | nucleus |
| 229310_at | KLHL29 | kelch-like 29 (Drosophila) | 3.74 | 27.2 | 7.27 | 3.2 x 10^-7^ |  |  |
| 202855_s_at | SLC16A3 | solute carrier family 16, member 3 (monocarboxylic acid transporter 4) | 2.27 | 16.5 | 7.27 | 3.2 x 10^-3^ | transport | membrane fraction |
| 1555905_a_at | C3orf23 | chromosome 3 open reading frame 23 | 1.62 | 11.8 | 7.26 | 8.1 x 10^-5^ |  | mitochondrion |
| 243581_at | LOC646470 | similar to proteaseome (prosome, macropain) 28 subunit, 3 | 0.21 | 1.5 | 7.25 | 1.8 x 10^-3^ |  |  |
| 218092_s_at | AGFG1 | ArfGAP with FG repeats 1 | 5.18 | 37.5 | 7.25 | 4.0 x 10^-7^ | acrosome assembly | nucleus |
| 225400_at | TSEN15 | tRNA splicing endonuclease 15 homolog (S. cerevisiae) | 1.75 | 12.7 | 7.24 | 2.5 x 10^-7^ | mRNA processing | nucleus |
| 216088_s_at | PSMA7 | proteasome (prosome, macropain) subunit, alpha type, 7 | 6.79 | 49.1 | 7.24 | 6.1 x 10^-8^ | ubiquitin-dependent protein catabolic process | proteasome complex |
| 222396_at | HN1 | hematological and neurological expressed 1 | 5.98 | 43.3 | 7.23 | 9.3 x 10^-6^ |  | nucleus |
| 211964_at | COL4A2 | collagen, type IV, alpha 2 | 0.71 | 5.1 | 7.23 | 6.7 x 10^-3^ | negative regulation of angiogenesis | extracellular region |
| 203889_at | SCG5 | secretogranin V (7B2 protein) | 0.23 | 1.7 | 7.23 | 1.3 x 10^-4^ | transport | extracellular region |
| 213847_at | PRPH | Peripherin | 0.21 | 1.5 | 7.23 | 5.6 x 10^-4^ |  | intermediate filament |
| 236179_at | CDH11 | cadherin 11, type 2, OB-cadherin (osteoblast) | 0.42 | 3.0 | 7.22 | 7.2 x 10^-3^ | skeletal system development | cytoplasm |
| 219702_at | PLAC1 | placenta-specific 1 | 0.21 | 1.5 | 7.22 | 1.5 x 10^-3^ | placenta development | extracellular region |
| 209946_at | VEGFC | vascular endothelial growth factor C | 1.26 | 9.1 | 7.21 | 2.2 x 10^-5^ | angiogenesis | extracellular region |
| 1569583_at | EREG | Epiregulin | 0.25 | 1.8 | 7.21 | 7.8 x 10^-3^ | angiogenesis | extracellular region |
| 213100_at | UNC5B | Unc-5 homolog B (C. elegans) | 1.51 | 10.8 | 7.20 | 3.2 x 10^-5^ | apoptosis | membrane |
| 203370_s_at | PDLIM7 | PDZ and LIM domain 7 (enigma) | 0.95 | 6.9 | 7.19 | 2.3 x 10^-4^ | ossification | cytoplasm |
| 210405_x_at | TNFRSF10B | tumor necrosis factor receptor superfamily, member 10b | 1.81 | 13.0 | 7.19 | 6.9 x 10^-6^ | apoptosis | plasma membrane |
| 220431_at | TMPRSS11E /// TMPRSS11E2 | transmembrane protease, serine 11E /// transmembrane protease, serine 11E2 | 0.41 | 2.9 | 7.19 | 1.1 x 10^-3^ | proteolysis | extracellular region |
| 211945_s_at | ITGB1 | integrin, beta 1 (fibronectin receptor, beta polypeptide, antigen CD29 includes MDF2, MSK12) | 58.32 | 418.7 | 7.18 | 3.3 x 10^-8^ | G1 | ruffle |
| 235756_at | --- | --- | 0.68 | 4.8 | 7.18 | 1.5 x 10^-4^ |  |  |
| 209294_x_at | TNFRSF10B | tumor necrosis factor receptor superfamily, member 10b | 1.86 | 13.4 | 7.18 | 1.2 x 10^-5^ | apoptosis | plasma membrane |
| 224583_at | COTL1 | coactosin-like 1 (Dictyostelium) | 3.24 | 23.3 | 7.17 | 1.2 x 10^-4^ | defense response to fungus | intracellular |
| 226899_at | UNC5B | unc-5 homolog B (C. elegans) | 5.24 | 37.6 | 7.17 | 4.2 x 10^-4^ | apoptosis | membrane |
| 204989_s_at | ITGB4 | integrin, beta 4 | 5.02 | 36.0 | 7.17 | 1.3 x 10^-3^ | cell communication | integrin complex |
| 217943_s_at | MAP7D1 | MAP7 domain containing 1 | 3.63 | 26.0 | 7.16 | 7.0 x 10^-4^ |  | spindle |
| 209894_at | LEPR | leptin receptor | 2.22 | 15.9 | 7.15 | 1.0 x 10^-5^ | angiogenesis | extracellular region |
| 225399_at | TSEN15 | tRNA splicing endonuclease 15 homolog (S. cerevisiae) | 5.92 | 42.3 | 7.15 | 5.6 x 10^-9^ | mRNA processing | nucleus |
| 226342_at | SPTBN1 | spectrin, beta, non-erythrocytic 1 | 1.61 | 11.5 | 7.14 | 1.1 x 10^-6^ | common-partner SMAD protein phosphorylation | nucleus |
| 211597_s_at | HOPX | HOP homeobox | 5.08 | 36.3 | 7.14 | 2.1 x 10^-5^ | negative regulation of transcription from RNA polymerase II promoter | nucleus |
| 200802_at | SARS | seryl-tRNA synthetase | 17.40 | 124.1 | 7.13 | 3.9 x 10^-11^ | translation | cytoplasm |
| 202148_s_at | PYCR1 | pyrroline-5-carboxylate reductase 1 | 2.29 | 16.4 | 7.13 | 5.4 x 10^-6^ | proline biosynthetic process | mitochondrion |
| 226863_at | FAM110C | family with sequence similarity 110, member C | 3.16 | 22.6 | 7.13 | 1.2 x 10^-8^ |  | cytoplasm |
| 217256_x_at | RPL36A /// RPL36AP51 | ribosomal protein L36a /// ribosomal protein L36a pseudogene 51 | 18.61 | 132.5 | 7.12 | 8.6 x 10^-8^ | translation | intracellular |
| 208800_at | SRP72 | signal recognition particle 72kDa | 7.04 | 50.0 | 7.10 | 6.2 x 10^-8^ | protein amino acid phosphorylation | nucleolus |
| 221027_s_at | PLA2G12A | phospholipase A2, group XIIA | 4.23 | 30.1 | 7.10 | 2.2 x 10^-3^ | phospholipid metabolic process | extracellular region |
| 232202_at | --- | --- | 3.13 | 22.2 | 7.10 | 7.9 x 10^-10^ |  |  |
| 209877_at | SNCG | synuclein, gamma (breast cancer-specific protein 1) | 0.28 | 2.0 | 7.10 | 4.7 x 10^-10^ |  | cytoplasm |
| 210587_at | INHBE | inhibin, beta E | 0.10 | 0.7 | 7.10 | 2.6 x 10^-4^ |  | extracellular region |
| 228355_s_at | NDUFAF2 | NADH dehydrogenase (ubiquinone) 1 alpha subcomplex, assembly factor 2 | 5.77 | 40.9 | 7.09 | 3.5 x 10^-10^ |  | mitochondrion |
| 238909_at | S100A10 | S100 calcium binding protein A10 | 4.01 | 28.4 | 7.08 | 5.7 x 10^-3^ | signal transduction | mitochondrion |
| 1561969_at | ZPLD1 | zona pellucida-like domain containing 1 | 0.26 | 1.9 | 7.08 | 8.2 x 10^-4^ |  | membrane |
| 242786_at | --- | --- | 0.61 | 4.3 | 7.08 | 1.2 x 10^-4^ |  |  |
| 211031_s_at | CLIP2 | CAP-GLY domain containing linker protein 2 | 0.35 | 2.5 | 7.08 | 1.3 x 10^-4^ |  | cytoplasm |
| 203946_s_at | ARG2 | arginase, type II | 7.85 | 55.6 | 7.08 | 1.7 x 10^-5^ | urea cycle | mitochondrion |
| 1554314_at | C6orf141 | chromosome 6 open reading frame 141 | 0.38 | 2.7 | 7.07 | 5.9 x 10^-5^ |  |  |
| 200779_at | ATF4 | activating transcription factor 4 (tax-responsive enhancer element B67) | 42.62 | 301.5 | 7.07 | 1.3 x 10^-10^ | gluconeogenesis | nucleus |
| 223316_at | CCDC3 | coiled-coil domain containing 3 | 0.95 | 6.7 | 7.07 | 3.5 x 10^-4^ |  | endoplasmic reticulum |
| 213358_at | KIAA0802 | KIAA0802 | 1.16 | 8.2 | 7.07 | 2.2 x 10^-9^ |  |  |
| 203298_s_at | JARID2 | jumonji, AT rich interactive domain 2 | 2.72 | 19.2 | 7.07 | 1.4 x 10^-7^ | negative regulation of transcription from RNA polymerase II promoter | intracellular |
| 207080_s_at | PYY | peptide YY | 0.14 | 1.0 | 7.06 | 7.6 x 10^-5^ | cell motion | extracellular region |
| 222989_s_at | UBQLN1 | ubiquilin 1 | 3.37 | 23.8 | 7.05 | 6.8 x 10^-6^ | response to hypoxia | nucleus |
| 225664_at | COL12A1 | collagen, type XII, alpha 1 | 1.32 | 9.3 | 7.05 | 2.8 x 10^-3^ | skeletal system development | extracellular region |
| 214205_x_at | GLRX3 | glutaredoxin 3 | 2.56 | 18.0 | 7.04 | 3.4 x 10^-7^ | cell redox homeostasis | cytoplasm |
| 222503_s_at | WDR41 | WD repeat domain 41 | 8.71 | 61.3 | 7.04 | 1.6 x 10^-6^ |  |  |
| 218401_s_at | ZNF281 | zinc finger protein 281 | 2.03 | 14.2 | 7.03 | 1.8 x 10^-5^ | negative regulation of transcription from RNA polymerase II promoter | intracellular |
| 201014_s_at | PAICS | phosphoribosylaminoimidazole carboxylase, phosphoribosylaminoimidazole succinocarboxamide synthetase | 5.42 | 38.1 | 7.03 | 1.6 x 10^-10^ | purine nucleotide biosynthetic process | phosphoribosylaminoimidazole carboxylase complex |
| 202771_at | FAM38A | family with sequence similarity 38, member A | 2.44 | 17.2 | 7.03 | 9.7 x 10^-7^ |  | endoplasmic reticulum |
| 205324_s_at | FTSJ1 | FtsJ homolog 1 (E. coli) | 11.23 | 78.9 | 7.02 | 2.2 x 10^-7^ | rRNA processing |  |
| 218193_s_at | GOLT1B | golgi transport 1 homolog B (S. cerevisiae) | 7.21 | 50.6 | 7.02 | 2.2 x 10^-5^ | transport | Golgi membrane |
| 218818_at | FHL3 | four and a half LIM domains 3 | 0.58 | 4.1 | 7.01 | 7.2 x 10^-4^ | muscle organ development | stress fiber |
| 37152_at | PPARD | peroxisome proliferator-activated receptor delta | 8.01 | 56.1 | 7.01 | 3.7 x 10^-4^ | negative regulation of transcription from RNA polymerase II promoter | nucleus |
| 202670_at | MAP2K1 | mitogen-activated protein kinase kinase 1 | 8.36 | 58.5 | 7.00 | 1.5 x 10^-9^ | protein amino acid phosphorylation | Golgi apparatus |
| 231925_at | --- | --- | 0.91 | 6.3 | 6.99 | 9.6 x 10^-5^ |  |  |
| 244665_at | --- | --- | 0.62 | 4.3 | 6.99 | 2.1 x 10^-6^ |  |  |
| 241333_x_at | --- | --- | 0.11 | 0.8 | 6.98 | 3.2 x 10^-3^ |  |  |
| 212566_at | MAP4 | microtubule-associated protein 4 | 7.85 | 54.8 | 6.98 | 5.4 x 10^-7^ | negative regulation of microtubule depolymerization | microtubule |
| 227204_at | PARD6G | par-6 partitioning defective 6 homolog gamma (C. elegans) | 2.34 | 16.4 | 6.98 | 2.0 x 10^-6^ | cell cycle | cytoplasm |
| 209646_x_at | ALDH1B1 | aldehyde dehydrogenase 1 family, member B1 | 0.42 | 2.9 | 6.97 | 9.5 x 10^-7^ | carbohydrate metabolic process | mitochondrion |
| 218728_s_at | CNIH4 | cornichon homolog 4 (Drosophila) | 9.35 | 65.1 | 6.97 | 4.1 x 10^-10^ | intracellular signaling cascade | endoplasmic reticulum |
| 205865_at | ARID3A | AT rich interactive domain 3A (BRIGHT-like) | 0.46 | 3.2 | 6.96 | 1.7 x 10^-6^ | transcription | intracellular |
| 237623_at | CST3 | cystatin C | 0.54 | 3.7 | 6.95 | 1.8 x 10^-4^ | defense response | extracellular region |
| 226276_at | TMEM167A | transmembrane protein 167A | 11.98 | 83.1 | 6.94 | 2.8 x 10^-8^ |  | membrane |
| 220102_at | FOXL2 | forkhead box L2 | 0.10 | 0.7 | 6.93 | 9.5 x 10^-4^ | ovarian follicle development | nucleus |
| 220239_at | KLHL7 | kelch-like 7 (Drosophila) | 1.76 | 12.2 | 6.93 | 5.1 x 10^-7^ |  | nucleus |
| 212992_at | AHNAK2 | AHNAK nucleoprotein 2 | 22.32 | 154.7 | 6.93 | 6.1 x 10^-8^ | keratinization | nucleus |
| 208540_x_at | S100A11 /// S100A11P | S100 calcium binding protein A11 /// S100 calcium binding protein A11 pseudogene | 31.39 | 217.0 | 6.91 | 1.8 x 10^-4^ | signal transduction | ruffle |
| 212737_at | GM2A | GM2 ganglioside activator | 13.38 | 92.5 | 6.91 | 4.6 x 10^-8^ | ganglioside metabolic process | cytoplasm |
| 226650_at | ZFAND2A | zinc finger, AN1-type domain 2A | 4.62 | 31.9 | 6.91 | 1.3 x 10^-5^ |  | nucleus |
| 201268_at | NME1 /// NME1-NME2 /// NME2 | non-metastatic cells 1, protein (NM23A) expressed in /// NME1-NME2 readthrough transcript /// non-metastatic cells 2, protein (NM23B) expressed in | 30.51 | 210.8 | 6.91 | 4.3 x 10^-11^ | GTP biosynthetic process | ruffle |
| 227759_at | PCSK9 | proprotein convertase subtilisin/kexin type 9 | 0.72 | 5.0 | 6.91 | 1.3 x 10^-3^ | kidney development | extracellular region |
| 206686_at | PDK1 | pyruvate dehydrogenase kinase, isozyme 1 | 0.42 | 2.9 | 6.91 | 3.2 x 10^-4^ | carbohydrate metabolic process | mitochondrion |
| 214164_x_at | CA12 | carbonic anhydrase XII | 4.66 | 32.2 | 6.90 | 3.8 x 10^-3^ | one-carbon compound metabolic process | cytoplasm |
| 203279_at | EDEM1 | ER degradation enhancer, mannosidase alpha-like 1 | 4.23 | 29.2 | 6.90 | 1.0 x 10^-6^ | response to unfolded protein | endoplasmic reticulum |
| 217901_at | DSG2 | desmoglein 2 | 23.64 | 163.0 | 6.90 | 3.7 x 10^-8^ | cell adhesion | plasma membrane |
| 234107_s_at | DTD1 | D-tyrosyl-tRNA deacylase 1 homolog (S. cerevisiae) | 10.03 | 69.2 | 6.89 | 1.0 x 10^-7^ | protein folding | nucleus |
| 231299_at | AGAP3 | ArfGAP with GTPase domain, ankyrin repeat and PH domain 3 | 0.44 | 3.0 | 6.89 | 3.5 x 10^-5^ | small GTPase mediated signal transduction | intracellular |
| 217948_at | FAM127B | family with sequence similarity 127, member B | 2.08 | 14.3 | 6.89 | 3.1 x 10^-5^ |  |  |
| 235453_at | TOR1AIP2 | torsin A interacting protein 2 | 0.16 | 1.1 | 6.89 | 7.9 x 10^-5^ |  | endoplasmic reticulum |
| 235296_at | EIF5A2 | eukaryotic translation initiation factor 5A2 | 1.29 | 8.9 | 6.88 | 3.2 x 10^-5^ | translation | cytosol |
| 219569_s_at | TMEM22 | transmembrane protein 22 | 0.48 | 3.3 | 6.87 | 9.2 x 10^-4^ |  | Golgi apparatus |
| 205724_at | PKP1 | plakophilin 1 (ectodermal dysplasia/skin fragility syndrome) | 0.48 | 3.3 | 6.87 | 9.5 x 10^-6^ | cell adhesion | nucleus |
| 1554703_at | ARHGEF10 | Rho guanine nucleotide exchange factor (GEF) 10 | 0.85 | 5.8 | 6.87 | 1.6 x 10^-4^ | regulation of Rho protein signal transduction | intracellular |
| 207654_x_at | DR1 | down-regulator of transcription 1, TBP-binding (negative cofactor 2) | 5.38 | 36.9 | 6.87 | 4.9 x 10^-8^ | negative regulation of transcription from RNA polymerase II promoter | intracellular |
| 209188_x_at | DR1 | down-regulator of transcription 1, TBP-binding (negative cofactor 2) | 5.52 | 37.9 | 6.86 | 1.2 x 10^-7^ | negative regulation of transcription from RNA polymerase II promoter | intracellular |
| 217584_at | --- | --- | 0.62 | 4.2 | 6.85 | 3.6 x 10^-7^ |  |  |
| 201231_s_at | ENO1 | enolase 1, (alpha) | 32.54 | 223.0 | 6.85 | 5.4 x 10^-4^ | negative regulation of transcription from RNA polymerase II promoter | phosphopyruvate hydratase complex |
| 1554256_a_at | PCNXL2 | pecanex-like 2 (Drosophila) | 0.68 | 4.7 | 6.85 | 8.6 x 10^-5^ |  | membrane |
| 208074_s_at | AP2S1 | adaptor-related protein complex 2, sigma 1 subunit | 11.61 | 79.5 | 6.85 | 9.3 x 10^-8^ | transport | cytosol |
| 230257_s_at | TSEN15 | tRNA splicing endonuclease 15 homolog (S. cerevisiae) | 2.85 | 19.5 | 6.84 | 1.2 x 10^-6^ | mRNA processing | nucleus |
| 210512_s_at | VEGFA | vascular endothelial growth factor A | 33.43 | 228.3 | 6.83 | 2.6 x 10^-7^ | angiogenesis | extracellular region |
| 242993_at | --- | --- | 0.58 | 3.9 | 6.82 | 1.0 x 10^-3^ |  |  |
| 218668_s_at | RAP2C | RAP2C, member of RAS oncogene family | 4.10 | 27.9 | 6.82 | 1.1 x 10^-6^ | small GTPase mediated signal transduction | intracellular |
| 239903_at | --- | --- | 1.12 | 7.6 | 6.82 | 4.1 x 10^-3^ |  |  |
| 205429_s_at | MPP6 | membrane protein, palmitoylated 6 (MAGUK p55 subfamily member 6) | 0.83 | 5.6 | 6.81 | 1.1 x 10^-9^ | protein complex assembly | plasma membrane |
| 212859_x_at | MT1E | metallothionein 1E | 52.77 | 359.5 | 6.81 | 1.4 x 10^-7^ |  | cytoplasm |
| 212295_s_at | SLC7A1 | solute carrier family 7 (cationic amino acid transporter, y+ system), member 1 | 19.15 | 130.4 | 6.81 | 3.2 x 10^-10^ | transport | integral to plasma membrane |
| 219855_at | NUDT11 | nudix (nucleoside diphosphate linked moiety X)-type motif 11 | 0.34 | 2.3 | 6.81 | 7.0 x 10^-3^ |  | intracellular |
| 202693_s_at | STK17A | serine/threonine kinase 17a | 16.75 | 114.0 | 6.81 | 7.6 x 10^-9^ | protein amino acid phosphorylation | nucleus |
| 223234_at | MAD2L2 | MAD2 mitotic arrest deficient-like 2 (yeast) | 2.42 | 16.5 | 6.80 | 4.5 x 10^-9^ | cell cycle |  |
| 214845_s_at | CALU | Calumenin | 4.11 | 27.9 | 6.80 | 6.9 x 10^-7^ |  | extracellular region |
| 211126_s_at | CSRP2 | cysteine and glycine-rich protein 2 | 1.78 | 12.1 | 6.80 | 3.0 x 10^-8^ | multicellular organismal development | nucleus |
| 224407_s_at | RP6-213H19.1 | serine/threonine protein kinase MST4 | 1.51 | 10.3 | 6.79 | 1.0 x 10^-5^ | protein amino acid phosphorylation | cytoplasm |
| 224598_at | MGAT4B | mannosyl (alpha-1,3-)-glycoprotein beta-1,4-N-acetylglucosaminyltransferase, isozyme B | 10.91 | 74.1 | 6.79 | 1.9 x 10^-6^ | carbohydrate metabolic process | Golgi membrane |
| 239908_at | --- | --- | 0.90 | 6.1 | 6.79 | 2.4 x 10^-6^ |  |  |
| 225367_at | PGM2 | phosphoglucomutase 2 | 7.18 | 48.7 | 6.79 | 1.5 x 10^-5^ | carbohydrate metabolic process | cytoplasm |
| 201692_at | SIGMAR1 | sigma non-opioidintracellular receptor 1 | 3.12 | 21.1 | 6.78 | 2.8 x 10^-6^ | ergosterol biosynthetic process | nucleus |
| 211356_x_at | LEPR | leptin receptor | 0.98 | 6.6 | 6.78 | 2.3 x 10^-5^ | angiogenesis | extracellular region |
| 200886_s_at | PGAM1 | phosphoglycerate mutase 1 (brain) | 33.93 | 230.0 | 6.78 | 8.4 x 10^-9^ | glycolysis | cytosol |
| 224851_at | CDK6 | cyclin-dependent kinase 6 | 7.85 | 53.2 | 6.78 | 1.1 x 10^-6^ | G1 phase of mitotic cell cycle | cyclin-dependent protein kinase holoenzyme complex |
| 224762_at | SERINC2 | serine incorporator 2 | 4.33 | 29.4 | 6.78 | 8.9 x 10^-6^ | phosphatidylserine metabolic process | membrane |
| 240176_at | --- | --- | 0.41 | 2.8 | 6.77 | 2.7 x 10^-7^ |  |  |
| 203741_s_at | ADCY7 | adenylate cyclase 7 | 1.08 | 7.3 | 6.77 | 7.1 x 10^-5^ | cAMP biosynthetic process | plasma membrane |
| 223349_s_at | BOK | BCL2-related ovarian killer | 1.12 | 7.6 | 6.77 | 2.3 x 10^-4^ | apoptosis |  |
| 243916_x_at | UBLCP1 | ubiquitin-like domain containing CTD phosphatase 1 | 7.51 | 50.8 | 6.77 | 4.1 x 10^-5^ | protein modification process | nucleus |
| 206714_at | ALOX15B | arachidonate 15-lipoxygenase, type B | 0.42 | 2.8 | 6.76 | 2.2 x 10^-3^ | lipid metabolic process | intracellular |
| 242727_at | ARL5B | ADP-ribosylation factor-like 5B | 1.13 | 7.6 | 6.76 | 4.2 x 10^-3^ | small GTPase mediated signal transduction | intracellular |
| 205729_at | OSMR | oncostatin M receptor | 1.87 | 12.6 | 6.76 | 4.9 x 10^-6^ | positive regulation of acute inflammatory response | oncostatin-M receptor complex |
| 207988_s_at | ARPC2 | actin related protein 2/3 complex, subunit 2, 34kDa | 27.69 | 187.1 | 6.76 | 3.3 x 10^-8^ | cell motion | cytoplasm |
| 203744_at | HMGB3 | high-mobility group box 3 | 10.43 | 70.4 | 6.75 | 8.5 x 10^-5^ | multicellular organismal development | nucleus |
| 213230_at | CDR2L | cerebellar degeneration-related protein 2-like | 0.73 | 4.9 | 6.75 | 1.9 x 10^-9^ |  |  |
| 220924_s_at | SLC38A2 | solute carrier family 38, member 2 | 46.58 | 314.2 | 6.75 | 4.0 x 10^-10^ | transport | plasma membrane |
| 209308_s_at | BNIP2 | BCL2/adenovirus E1B 19kDa interacting protein 2 | 5.45 | 36.7 | 6.74 | 9.9 x 10^-7^ | apoptosis | nuclear envelope |
| 239026_x_at | AGAP3 | ArfGAP with GTPase domain, ankyrin repeat and PH domain 3 | 0.70 | 4.7 | 6.74 | 2.5 x 10^-5^ | small GTPase mediated signal transduction | intracellular |
| 202843_at | DNAJB9 | DnaJ (Hsp40) homolog, subfamily B, member 9 | 2.88 | 19.4 | 6.74 | 2.1 x 10^-4^ | protein folding | nucleus |
| 212290_at | SLC7A1 | solute carrier family 7 (cationic amino acid transporter, y+ system), member 1 | 3.41 | 23.0 | 6.73 | 3.0 x 10^-5^ | transport | integral to plasma membrane |
| 213262_at | SACS | spastic ataxia of Charlevoix-Saguenay (sacsin) | 1.36 | 9.1 | 6.73 | 8.4 x 10^-6^ | protein folding |  |
| 203672_x_at | TPMT | thiopurine S-methyltransferase | 2.13 | 14.3 | 6.72 | 1.2 x 10^-6^ | nucleobase, nucleoside, nucleotide and nucleic acid metabolic process | cytoplasm |
| 212251_at | MTDH | Metadherin | 16.34 | 109.6 | 6.71 | 2.0 x 10^-7^ | negative regulation of transcription from RNA polymerase II promoter | nucleus |
| 224151_s_at | AK3 | adenylate kinase 3 | 4.93 | 33.1 | 6.71 | 7.7 x 10^-4^ | nucleobase, nucleoside, nucleotide and nucleic acid metabolic process | mitochondrion |
| 1557905_s_at | CD44 | CD44 molecule (Indian blood group) | 4.20 | 28.1 | 6.69 | 2.4 x 10^-4^ | regulation of cell growth | cytoplasm |
| 222936_s_at | PPPDE1 | PPPDE peptidase domain containing 1 | 0.17 | 1.1 | 6.69 | 7.2 x 10^-5^ |  |  |
| 224856_at | FKBP5 | FK506 binding protein 5 | 3.64 | 24.3 | 6.68 | 6.0 x 10^-5^ | protein folding | nucleus |
| 1563900_at | FAM83B | family with sequence similarity 83, member B | 0.53 | 3.6 | 6.67 | 1.4 x 10^-4^ |  |  |
| 220672_at | PPP4R4 | protein phosphatase 4, regulatory subunit 4 | 0.54 | 3.6 | 6.67 | 1.1 x 10^-8^ |  | cytoplasm |
| 212590_at | RRAS2 | related RAS viral (r-ras) oncogene homolog 2 | 12.03 | 80.2 | 6.66 | 1.4 x 10^-8^ | small GTPase mediated signal transduction | intracellular |
| 206482_at | PTK6 | PTK6 protein tyrosine kinase 6 | 1.96 | 13.0 | 6.66 | 7.8 x 10^-6^ | protein amino acid phosphorylation | nucleus |
| 214443_at | PVR | poliovirus receptor | 0.35 | 2.3 | 6.65 | 1.6 x 10^-4^ | positive regulation of natural killer cell mediated cytotoxicity directed against tumor cell target | extracellular region |
| 201481_s_at | PYGB | phosphorylase, glycogen; brain | 5.14 | 34.2 | 6.65 | 6.5 x 10^-8^ | carbohydrate metabolic process | cytoplasm |
| 219998_at | HSPC159 | galectin-related protein | 0.71 | 4.8 | 6.65 | 2.6 x 10^-6^ |  | intracellular |
| 1555551_at | SERPINB5 | serpin peptidase inhibitor, clade B (ovalbumin), member 5 | 0.65 | 4.3 | 6.65 | 5.8 x 10^-4^ | cell motion | extracellular region |
| 1553994_at | NT5E | 5'-nucleotidase, ecto (CD73) | 1.22 | 8.1 | 6.64 | 7.9 x 10^-4^ | purine nucleotide biosynthetic process | membrane fraction |
| 209152_s_at | TCF3 | transcription factor 3 (E2A immunoglobulin enhancer binding factors E12/E47) | 1.01 | 6.7 | 6.64 | 2.6 x 10^-7^ | B cell lineage commitment | nucleus |
| 228378_at | C12orf29 | chromosome 12 open reading frame 29 | 2.88 | 19.1 | 6.64 | 2.8 x 10^-7^ |  |  |
| 205180_s_at | ADAM8 | ADAM metallopeptidase domain 8 | 1.11 | 7.4 | 6.64 | 4.6 x 10^-8^ | proteolysis | plasma membrane |
| 1560407_at | MARK1 | MAP/microtubule affinity-regulating kinase 1 | 0.76 | 5.0 | 6.62 | 3.6 x 10^-4^ | protein amino acid phosphorylation | cytoplasm |
| 239710_at | FIGN | fidgetin | 0.23 | 1.5 | 6.61 | 1.2 x 10^-3^ |  |  |
| 212470_at | SPAG9 | sperm associated antigen 9 | 6.90 | 45.6 | 6.61 | 8.0 x 10^-8^ | activation of MAPK activity | acrosomal vesicle |
| 209743_s_at | ITCH | itchy E3 ubiquitin protein ligase homolog (mouse) | 1.48 | 9.8 | 6.61 | 5.8 x 10^-6^ | regulation of cell growth | intracellular |
| 229494_s_at | PHLDA2 | Pleckstrin homology-like domain, family A, member 2 | 0.10 | 0.6 | 6.60 | 4.4 x 10^-3^ | apoptosis | cytoplasm |
| 211822_s_at | NLRP1 | NLR family, pyrin domain containing 1 | 0.81 | 5.3 | 6.60 | 2.4 x 10^-4^ | apoptosis | intracellular |
| 206295_at | IL18 | interleukin 18 (interferon-gamma-inducing factor) | 4.02 | 26.5 | 6.59 | 5.2 x 10^-9^ | angiogenesis | extracellular region |
| 220198_s_at | EIF5A2 | eukaryotic translation initiation factor 5A2 | 0.62 | 4.1 | 6.59 | 2.1 x 10^-4^ | translation | cytosol |
| 222442_s_at | ARL8B | ADP-ribosylation factor-like 8B | 8.94 | 58.9 | 6.59 | 3.3 x 10^-6^ | chromosome segregation | intracellular |
| 220983_s_at | SPRY4 | sprouty homolog 4 (Drosophila) | 0.14 | 1.0 | 6.59 | 6.5 x 10^-4^ | multicellular organismal development | cytoplasm |
| 223738_s_at | PGM2 | phosphoglucomutase 2 | 3.08 | 20.3 | 6.58 | 1.1 x 10^-5^ | carbohydrate metabolic process | cytoplasm |
| 213062_at | NTAN1 | N-terminal asparagine amidase | 2.78 | 18.3 | 6.58 | 2.4 x 10^-8^ | memory | nucleus |
| 215867_x_at | CA12 | carbonic anhydrase XII | 5.07 | 33.4 | 6.58 | 3.1 x 10^-3^ | one-carbon compound metabolic process | cytoplasm |
| 235287_at | CDK6 | cyclin-dependent kinase 6 | 0.68 | 4.5 | 6.57 | 3.1 x 10^-7^ | G1 phase of mitotic cell cycle | cyclin-dependent protein kinase holoenzyme complex |
| 238002_at | --- | --- | 3.42 | 22.5 | 6.57 | 8.5 x 10^-9^ |  |  |
| 217379_at | RPL10 | ribosomal protein L10 | 15.57 | 102.2 | 6.57 | 8.1 x 10^-5^ | translation | intracellular |
| 226610_at | CENPV | centromere protein V | 0.70 | 4.6 | 6.57 | 7.3 x 10^-3^ | cell cycle | kinetochore |
| 218041_x_at | SLC38A2 | solute carrier family 38, member 2 | 48.56 | 318.6 | 6.56 | 2.4 x 10^-10^ | transport | plasma membrane |
| 226231_at | --- | --- | 1.74 | 11.4 | 6.56 | 1.8 x 10^-8^ |  |  |
| 213154_s_at | BICD2 | bicaudal D homolog 2 (Drosophila) | 6.83 | 44.8 | 6.56 | 1.0 x 10^-7^ | transport | cytoplasm |
| 236281_x_at | HTR7 | 5-hydroxytryptamine (serotonin) receptor 7 (adenylate cyclase-coupled) | 0.35 | 2.3 | 6.55 | 1.7 x 10^-4^ | signal transduction | plasma membrane |
| 202068_s_at | LDLR | low density lipoprotein receptor | 21.10 | 138.2 | 6.55 | 2.1 x 10^-8^ | protein amino acid O-linked glycosylation | endosome |
| 1557411_s_at | SLC25A43 | solute carrier family 25, member 43 | 3.38 | 22.2 | 6.55 | 1.2 x 10^-8^ | translation | intracellular |
| 205569_at | LAMP3 | lysosomal-associated membrane protein 3 | 9.64 | 63.1 | 6.55 | 1.9 x 10^-3^ | cell proliferation | lysosome |
| 209324_s_at | RGS16 | regulator of G-protein signaling 16 | 0.25 | 1.6 | 6.54 | 6.3 x 10^-3^ | visual perception |  |
| 209526_s_at | HDGFRP3 | hepatoma-derived growth factor, related protein 3 | 5.37 | 35.1 | 6.54 | 3.2 x 10^-8^ | cell proliferation | nucleus |
| 228865_at | C1orf116 | chromosome 1 open reading frame 116 | 10.76 | 70.4 | 6.54 | 2.6 x 10^-8^ |  | cytoplasm |
| 203373_at | SOCS2 | suppressor of cytokine signaling 2 | 1.53 | 10.0 | 6.54 | 1.2 x 10^-5^ | regulation of cell growth | cytoplasm |
| 226145_s_at | FRAS1 | Fraser syndrome 1 | 3.09 | 20.2 | 6.54 | 2.1 x 10^-5^ | morphogenesis of an epithelium | basement membrane |
| 209209_s_at | FERMT2 | fermitin family homolog 2 (Drosophila) | 2.09 | 13.6 | 6.54 | 5.0 x 10^-5^ | cell adhesion | stress fiber |
| 204818_at | HSD17B2 | hydroxysteroid (17-beta) dehydrogenase 2 | 1.85 | 12.1 | 6.53 | 1.4 x 10^-3^ | steroid biosynthetic process | endoplasmic reticulum membrane |
| 1561286_a_at | DIP2A | DIP2 disco-interacting protein 2 homolog A (Drosophila) | 0.57 | 3.7 | 6.53 | 6.1 x 10^-4^ | multicellular organismal development | nucleus |
| 217257_at | SH3BP2 | SH3-domain binding protein 2 | 0.40 | 2.6 | 6.53 | 2.2 x 10^-6^ | signal transduction |  |
| 203542_s_at | KLF9 | Kruppel-like factor 9 | 3.03 | 19.8 | 6.52 | 3.9 x 10^-3^ | transcription | intracellular |
| 209383_at | DDIT3 /// NR1H3 | DNA-damage-inducible transcript 3 /// nuclear receptor subfamily 1, group H, member 3 | 4.80 | 31.3 | 6.52 | 2.4 x 10^-3^ | response to amphetamine | intracellular |
| 219271_at | GALNT14 | UDP-N-acetyl-alpha-D-galactosamine:polypeptide N-acetylgalactosaminyltransferase 14 (GalNAc-T14) | 2.12 | 13.8 | 6.51 | 9.3 x 10^-5^ |  | Golgi membrane |
| 1553959_a_at | B3GALT6 | UDP-Gal:betaGal beta 1,3-galactosyltransferase polypeptide 6 | 4.86 | 31.6 | 6.51 | 3.4 x 10^-3^ | glycosaminoglycan biosynthetic process | Golgi apparatus |
| 208700_s_at | TKT | transketolase | 18.59 | 121.0 | 6.51 | 2.5 x 10^-4^ | metabolic process | cytosol |
| 213927_at | MAP3K9 | mitogen-activated protein kinase kinase kinase 9 | 1.85 | 12.0 | 6.51 | 9.3 x 10^-7^ | activation of JUN kinase activity |  |
| 227368_at | --- | --- | 1.46 | 9.5 | 6.50 | 2.0 x 10^-3^ |  |  |
| 229221_at | CD44 | CD44 molecule (Indian blood group) | 0.79 | 5.1 | 6.50 | 5.5 x 10^-8^ | regulation of cell growth | cytoplasm |
| 204819_at | FGD1 | FYVE, RhoGEF and PH domain containing 1 | 0.63 | 4.1 | 6.50 | 3.2 x 10^-5^ | cytoskeleton organization | ruffle |
| 229905_at | RAP1GDS1 | RAP1, GTP-GDP dissociation stimulator 1 | 4.49 | 29.2 | 6.50 | 1.6 x 10^-5^ |  |  |
| 222108_at | AMIGO2 | adhesion molecule with Ig-like domain 2 | 14.02 | 91.1 | 6.50 | 2.9 x 10^-3^ | cell adhesion | nucleus |
| 225331_at | CCDC50 | coiled-coil domain containing 50 | 11.59 | 75.2 | 6.49 | 5.2 x 10^-8^ | sensory perception of sound | cytoplasm |
| 225211_at | PVRL1 | poliovirus receptor-related 1 (herpesvirus entry mediator C) | 3.33 | 21.6 | 6.49 | 3.6 x 10^-5^ | lens morphogenesis in camera-type eye | extracellular region |
| 201790_s_at | DHCR7 | 7-dehydrocholesterol reductase | 3.26 | 21.1 | 6.49 | 2.8 x 10^-6^ | blood vessel development | nuclear outer membrane |
| 1557545_s_at | RNF165 | ring finger protein 165 | 0.74 | 4.8 | 6.49 | 3.1 x 10^-4^ |  |  |
| 223143_s_at | AKIRIN2 | akirin 2 | 0.80 | 5.2 | 6.48 | 2.3 x 10^-4^ | transcription | nucleus |
| 202856_s_at | SLC16A3 | solute carrier family 16, member 3 (monocarboxylic acid transporter 4) | 6.25 | 40.5 | 6.48 | 6.0 x 10^-4^ | transport | membrane fraction |
| 229952_at | --- | --- | 1.52 | 9.9 | 6.48 | 1.2 x 10^-4^ |  |  |
| 204653_at | TFAP2A | transcription factor AP-2 alpha (activating enhancer binding protein 2 alpha) | 9.94 | 64.2 | 6.46 | 2.8 x 10^-9^ | transcription | nucleus |
| 226726_at | MBOAT2 | membrane bound O-acyltransferase domain containing 2 | 6.62 | 42.7 | 6.46 | 1.0 x 10^-8^ | phospholipid biosynthetic process | membrane |
| 219911_s_at | LOC100134295 /// SLCO4A1 | similar to Solute carrier organic anion transporter family, member 4A1 /// solute carrier organic anion transporter family, member 4A1 | 0.32 | 2.1 | 6.45 | 4.3 x 10^-3^ | transport | membrane |
| 223231_at | TATDN1 | TatD DNase domain containing 1 | 7.69 | 49.6 | 6.45 | 5.1 x 10^-6^ |  | nucleus |
| 217253_at | SH3BP2 | SH3-domain binding protein 2 | 1.09 | 7.0 | 6.45 | 9.9 x 10^-3^ | signal transduction |  |
| 230574_at | LOC100130938 | hypothetical protein LOC100130938 | 0.44 | 2.8 | 6.44 | 1.2 x 10^-6^ |  |  |
| 218526_s_at | RANGRF | RAN guanine nucleotide release factor | 2.87 | 18.5 | 6.44 | 2.6 x 10^-7^ | transport | nucleus |
| 236045_x_at | --- | --- | 1.38 | 8.9 | 6.44 | 1.1 x 10^-3^ |  |  |
| 208892_s_at | DUSP6 | dual specificity phosphatase 6 | 13.57 | 87.4 | 6.44 | 3.0 x 10^-7^ | inactivation of MAPK activity | soluble fraction |
| 1559141_s_at | FAM87B | family with sequence similarity 87, member B | 0.12 | 0.8 | 6.43 | 4.6 x 10^-4^ |  | membrane |
| 206832_s_at | SEMA3F | sema domain, immunoglobulin domain (Ig), short basic domain, secreted, (semaphorin) 3F | 1.36 | 8.8 | 6.43 | 3.1 x 10^-6^ | multicellular organismal development | extracellular region |
| 219038_at | MORC4 | MORC family CW-type zinc finger 4 | 2.34 | 15.0 | 6.43 | 1.1 x 10^-7^ |  |  |
| 238996_x_at | ALDOA | aldolase A, fructose-bisphosphate | 5.26 | 33.8 | 6.42 | 4.1 x 10^-6^ | fructose metabolic process | nucleus |
| 202728_s_at | LTBP1 | latent transforming growth factor beta binding protein 1 | 0.20 | 1.3 | 6.42 | 5.1 x 10^-3^ |  | extracellular region |
| 224847_at | CDK6 | cyclin-dependent kinase 6 | 6.17 | 39.6 | 6.41 | 3.8 x 10^-5^ | G1 phase of mitotic cell cycle | cyclin-dependent protein kinase holoenzyme complex |
| 229011_at | --- | --- | 0.71 | 4.6 | 6.41 | 2.5 x 10^-3^ |  |  |
| 236088_at | NTNG1 | netrin G1 | 0.36 | 2.3 | 6.41 | 8.3 x 10^-5^ | multicellular organismal development | proteinaceousextracellular matrix |
| 219601_s_at | C10orf12 | chromosome 10 open reading frame 12 | 0.25 | 1.6 | 6.41 | 8.3 x 10^-4^ |  |  |
| 208425_s_at | TANC2 | tetratricopeptide repeat, ankyrin repeat and coiled-coil containing 2 | 0.97 | 6.2 | 6.41 | 1.5 x 10^-7^ |  |  |
| 206569_at | IL24 | interleukin 24 | 0.24 | 1.5 | 6.40 | 1.2 x 10^-3^ | apoptosis | extracellular region |
| 241994_at | XDH | xanthine dehydrogenase | 2.28 | 14.6 | 6.40 | 8.9 x 10^-5^ | lactation | peroxisome |
| 205463_s_at | PDGFA | platelet-derived growth factor alpha polypeptide | 3.05 | 19.4 | 6.39 | 5.8 x 10^-6^ | cell activation | extracellular region |
| 201791_s_at | DHCR7 | 7-dehydrocholesterol reductase | 2.15 | 13.7 | 6.38 | 5.8 x 10^-6^ | blood vessel development | nuclear outer membrane |
| 211905_s_at | ITGB4 | integrin, beta 4 | 1.57 | 10.0 | 6.37 | 8.8 x 10^-3^ | cell communication | integrin complex |
| 215780_s_at | hCG_1644608 /// SET | SET translocation (myeloid leukemia-associated) pseudogene /// SET nuclear oncogene | 10.44 | 66.5 | 6.37 | 6.8 x 10^-8^ | DNA replication | nucleus |
| 214266_s_at | PDLIM7 | PDZ and LIM domain 7 (enigma) | 0.41 | 2.6 | 6.37 | 3.3 x 10^-4^ | ossification | cytoplasm |
| 230831_at | FRMD5 | FERM domain containing 5 | 0.15 | 1.0 | 6.37 | 2.3 x 10^-3^ |  | cytoplasm |
| 210984_x_at | EGFR | epidermal growth factor receptor (erythroblastic leukemia viral (v-erb-b) oncogene homolog, avian) | 2.38 | 15.1 | 6.36 | 2.4 x 10^-3^ | ossification | extracellular region |
| 223950_s_at | FLYWCH1 | FLYWCH-type zinc finger 1 | 1.76 | 11.2 | 6.36 | 3.2 x 10^-4^ |  | nucleus |
| 213404_s_at | RHEB | Ras homolog enriched in brain | 12.10 | 77.0 | 6.36 | 7.7 x 10^-7^ | signal transduction | intracellular |
| 221784_at | WIZ | widely interspaced zinc finger motifs | 0.33 | 2.1 | 6.36 | 1.7 x 10^-3^ |  | intracellular |
| 238075_at | CHEK1 | CHK1 checkpoint homolog (S. pombe) | 0.75 | 4.8 | 6.35 | 9.2 x 10^-3^ | DNA damage checkpoint | condensed nuclearchromosome |
| 202734_at | TRIP10 | thyroid hormone receptor interactor 10 | 2.83 | 18.0 | 6.35 | 3.7 x 10^-7^ | endocytosis | cytoplasm |
| 202647_s_at | NRAS | neuroblastoma RAS viral (v-ras) oncogene homolog | 5.07 | 32.2 | 6.34 | 5.1 x 10^-6^ | endocytosis | Golgi membrane |
| 205341_at | EHD2 | EH-domain containing 2 | 0.88 | 5.6 | 6.34 | 2.9 x 10^-7^ | translation | intracellular |
| 202422_s_at | ACSL4 | acyl-CoA synthetase long-chain family member 4 | 1.54 | 9.8 | 6.34 | 5.8 x 10^-7^ | lipid metabolic process | cytoplasm |
| 219361_s_at | AEN | apoptosis enhancing nuclease | 0.82 | 5.2 | 6.33 | 5.0 x 10^-5^ | apoptosis | intracellular |
| 216640_s_at | PDIA6 | protein disulfide isomerase family A, member 6 | 25.90 | 164.0 | 6.33 | 1.5 x 10^-10^ | protein folding | endoplasmic reticulum |
| 219979_s_at | C11orf73 | chromosome 11 open reading frame 73 | 3.19 | 20.2 | 6.33 | 6.9 x 10^-7^ | Golgi organization | cytoplasm |
| 210041_s_at | PGM3 | phosphoglucomutase 3 | 4.41 | 27.9 | 6.33 | 1.8 x 10^-5^ | carbohydrate metabolic process |  |
| 1556429_a_at | WDR67 | WD repeat domain 67 | 0.12 | 0.8 | 6.33 | 5.9 x 10^-3^ | regulation of Rab GTPase activity | intracellular |
| 203510_at | MET | met proto-oncogene (hepatocyte growth factor receptor) | 30.90 | 195.3 | 6.32 | 3.8 x 10^-7^ | activation of MAPK activity | membrane fraction |
| 226225_at | MCC | mutated in colorectal cancers | 3.89 | 24.6 | 6.32 | 8.8 x 10^-6^ | cell cycle |  |
| 223145_s_at | AKIRIN2 | akirin 2 | 4.92 | 31.1 | 6.32 | 8.6 x 10^-5^ | transcription | nucleus |
| 207595_s_at | BMP1 | bone morphogenetic protein 1 | 0.72 | 4.5 | 6.31 | 5.3 x 10^-5^ | skeletal system development | extracellular space |
| 217938_s_at | KCMF1 | potassium channel modulatory factor 1 | 30.16 | 190.0 | 6.30 | 4.8 x 10^-6^ | modification-dependent protein catabolic process | intracellular |
| 225522_at | AAK1 | AP2 associated kinase 1 | 5.78 | 36.4 | 6.30 | 1.6 x 10^-5^ | protein amino acid phosphorylation | plasma membrane |
| 233565_s_at | SDCBP2 | syndecan binding protein (syntenin) 2 | 3.82 | 24.0 | 6.30 | 8.6 x 10^-6^ | intracellular signaling cascade | cytoplasm |
| 219155_at | PITPNC1 | phosphatidylinositol transfer protein, cytoplasmic 1 | 2.84 | 17.9 | 6.29 | 2.0 x 10^-5^ | transport | intracellular |
| 226099_at | ELL2 | elongation factor, RNA polymerase II, 2 | 15.76 | 99.1 | 6.29 | 2.6 x 10^-8^ | transcription | nucleus |
| 242883_at | OTOS | otospiralin | 0.12 | 0.8 | 6.29 | 8.9 x 10^-5^ | sensory perception of sound | extracellular region |
| 239845_at | --- | --- | 0.85 | 5.4 | 6.29 | 2.0 x 10^-6^ |  |  |
| 241044_x_at | --- | --- | 0.38 | 2.4 | 6.29 | 3.0 x 10^-3^ |  |  |
| 204285_s_at | PMAIP1 | phorbol-12-myristate-13-acetate-induced protein 1 | 8.50 | 53.4 | 6.29 | 1.8 x 10^-3^ | release of cytochrome c from mitochondria | mitochondrion |
| 218051_s_at | NT5DC2 | 5'-nucleotidase domain containing 2 | 3.07 | 19.3 | 6.28 | 6.3 x 10^-4^ |  |  |
| 203740_at | MPHOSPH6 | M-phase phosphoprotein 6 | 10.17 | 63.9 | 6.28 | 2.4 x 10^-9^ | M phase of mitotic cell cycle | nucleus |
| 225317_at | ACBD6 | acyl-Coenzyme A binding domain containing 6 | 3.66 | 23.0 | 6.28 | 2.0 x 10^-8^ |  | cytoplasm |
| 204508_s_at | CA12 | carbonic anhydrase XII | 1.42 | 8.9 | 6.27 | 1.7 x 10^-3^ | one-carbon compound metabolic process | cytoplasm |
| 201579_at | FAT1 | FAT tumor suppressor homolog 1 (Drosophila) | 10.67 | 66.9 | 6.27 | 1.3 x 10^-5^ | cell adhesion | plasma membrane |
| 232406_at | --- | --- | 2.01 | 12.6 | 6.27 | 5.1 x 10^-4^ |  |  |
| 204030_s_at | SCHIP1 | schwannomin interacting protein 1 | 2.50 | 15.7 | 6.26 | 2.5 x 10^-7^ |  | nucleus |
| 201849_at | BNIP3 | BCL2/adenovirus E1B 19kDa interacting protein 3 | 16.79 | 105.1 | 6.26 | 3.8 x 10^-8^ | response to hypoxia | nucleus |
| 222757_s_at | ZAK | sterile alpha motif and leucine zipper containing kinase AZK | 2.52 | 15.8 | 6.25 | 4.1 x 10^-7^ | cell cycle checkpoint | nucleus |
| 205503_at | PTPN14 | protein tyrosine phosphatase, non-receptor type 14 | 0.82 | 5.1 | 6.25 | 3.1 x 10^-9^ | protein amino acid dephosphorylation | cytoplasm |
| 238973_s_at | LOC440731 | Similar to hCG1817424 | 0.27 | 1.7 | 6.25 | 9.4 x 10^-6^ |  |  |
| 213513_x_at | ARPC2 | actin related protein 2/3 complex, subunit 2, 34kDa | 28.54 | 178.3 | 6.25 | 2.0 x 10^-8^ | cell motion | cytoplasm |
| 201475_x_at | MARS | methionyl-tRNA synthetase | 13.06 | 81.6 | 6.25 | 5.1 x 10^-11^ | translation | cytoplasm |
| 205376_at | INPP4B | inositol polyphosphate-4-phosphatase, type II, 105kDa | 3.47 | 21.7 | 6.25 | 7.9 x 10^-10^ | signal transduction |  |
| 200755_s_at | CALU | calumenin | 5.89 | 36.8 | 6.24 | 2.6 x 10^-7^ |  | extracellular region |
| 232158_x_at | NIPAL1 | NIPA-like domain containing 1 | 0.97 | 6.1 | 6.24 | 7.4 x 10^-3^ |  | membrane |
| 212607_at | AKT3 | v-akt murine thymoma viral oncogene homolog 3 (protein kinase B, gamma) | 1.49 | 9.3 | 6.24 | 6.8 x 10^-7^ | protein amino acid phosphorylation | cytoplasm |
| 227088_at | PDE5A | phosphodiesterase 5A, cGMP-specific | 0.44 | 2.7 | 6.24 | 1.7 x 10^-4^ | response to hypoxia | cytoplasm |
| 201088_at | KPNA2 | karyopherin alpha 2 (RAG cohort 1, importin alpha 1) | 13.27 | 82.7 | 6.24 | 2.2 x 10^-3^ | regulation of DNA recombination | nucleus |
| 219582_at | OGFRL1 | opioid growth factor receptor-like 1 | 2.37 | 14.8 | 6.23 | 3.3 x 10^-7^ |  | membrane |
| 213798_s_at | CAP1 | CAP, adenylate cyclase-associated protein 1 (yeast) | 28.01 | 174.5 | 6.23 | 3.8 x 10^-9^ | cell morphogenesis | cytoplasm |
| 1560848_at | --- | --- | 0.19 | 1.2 | 6.23 | 2.3 x 10^-4^ |  |  |
| 227761_at | MYO5A | myosin VA (heavy chain 12, myoxin) | 2.45 | 15.3 | 6.23 | 2.5 x 10^-5^ | ubiquitin-dependent protein catabolic process | ruffle |
| 1554149_at | CLDND1 | claudin domain containing 1 | 9.07 | 56.5 | 6.22 | 5.1 x 10^-9^ |  | membrane |
| 232467_at | KIRREL | kin of IRRE like (Drosophila) | 0.20 | 1.3 | 6.22 | 9.8 x 10^-4^ | excretion | plasma membrane |
| 206091_at | MATN3 | matrilin 3 | 0.16 | 1.0 | 6.22 | 3.5 x 10^-6^ | skeletal system development | extracellular region |
| 214078_at | --- | --- | 0.08 | 0.5 | 6.22 | 3.5 x 10^-3^ |  |  |
| 200744_s_at | GNB1 | guanine nucleotide binding protein (G protein), beta polypeptide 1 | 14.65 | 91.0 | 6.21 | 3.5 x 10^-7^ | signal transduction | photoreceptor outer segment |
| 208231_at | NRG1 | neuregulin 1 | 0.20 | 1.2 | 6.21 | 6.1 x 10^-5^ | cell communication | extracellular region |
| 31845_at | ELF4 | E74-like factor 4 (ets domain transcription factor) | 3.96 | 24.6 | 6.20 | 1.4 x 10^-4^ | natural killer cell proliferation | nucleus |
| 218718_at | PDGFC | platelet derived growth factor C | 12.67 | 78.6 | 6.20 | 2.6 x 10^-7^ | activation of transmembrane receptor protein tyrosine kinase activity | extracellular region |
| 211607_x_at | EGFR | epidermal growth factor receptor (erythroblastic leukemia viral (v-erb-b) oncogene homolog, avian) | 2.21 | 13.7 | 6.20 | 3.9 x 10^-3^ | ossification | extracellular region |
| 227124_at | LOC221710 | hypothetical protein LOC221710 | 4.78 | 29.7 | 6.20 | 2.0 x 10^-5^ | regulation of transcription |  |
| 231211_s_at | YIF1B | Yip1 interacting factor homolog B (S. cerevisiae) | 0.35 | 2.2 | 6.20 | 3.1 x 10^-4^ |  | membrane |
| 1556316_s_at | LOC284889 | hypothetical protein LOC284889 | 0.39 | 2.4 | 6.19 | 1.2 x 10^-4^ |  |  |
| 212472_at | MICAL2 | microtubule associated monoxygenase, calponin and LIM domain containing 2 | 2.16 | 13.4 | 6.19 | 1.4 x 10^-3^ | metabolic process | cytoplasm |
| 209080_x_at | GLRX3 | glutaredoxin 3 | 15.86 | 98.1 | 6.18 | 1.3 x 10^-11^ | cell redox homeostasis | cytoplasm |
| 1553219_a_at | AMMECR1 | Alport syndrome, mental retardation, midface hypoplasia and elliptocytosis chromosomal region gene 1 | 0.95 | 5.9 | 6.18 | 9.2 x 10^-7^ |  |  |
| 1553113_s_at | CDK8 | cyclin-dependent kinase 8 | 2.54 | 15.7 | 6.18 | 4.6 x 10^-7^ | transcription | mediator complex |
| 232678_at | SLC2A9 | Solute carrier family 2 (facilitated glucose transporter), member 9 | 0.37 | 2.3 | 6.18 | 5.7 x 10^-6^ | carbohydrate transport | membrane |
| 222269_at | APOOL | apolipoprotein O-like | 0.91 | 5.7 | 6.18 | 5.9 x 10^-6^ |  | extracellular region |
| 205263_at | BCL10 | B-cell CLL/lymphoma 10 | 14.09 | 87.0 | 6.18 | 4.9 x 10^-8^ | neural tube closure | immunological synapse |
| 219247_s_at | ZDHHC14 | zinc finger, DHHC-type containing 14 | 0.42 | 2.6 | 6.18 | 1.1 x 10^-4^ |  | membrane |
| 229845_at | MAPKAP1 | mitogen-activated protein kinase associated protein 1 | 1.58 | 9.7 | 6.17 | 1.6 x 10^-6^ |  | nucleus |
| 1555416_a_at | ALOX15B | arachidonate 15-lipoxygenase, type B | 0.22 | 1.4 | 6.17 | 3.1 x 10^-3^ | lipid metabolic process | intracellular |
| 222530_s_at | MKKS | McKusick-Kaufman syndrome | 6.67 | 41.1 | 6.16 | 1.2 x 10^-6^ | heart looping | intracellular |
| 216348_at | RPS17P5 | ribosomal protein S17 pseudogene 5 | 5.40 | 33.2 | 6.16 | 6.8 x 10^-5^ | translation | intracellular |
| 210058_at | MAPK13 | mitogen-activated protein kinase 13 | 4.03 | 24.8 | 6.16 | 1.4 x 10^-5^ | protein amino acid phosphorylation |  |
| 227271_at | FGF11 | fibroblast growth factor 11 | 1.35 | 8.3 | 6.16 | 2.5 x 10^-6^ | signal transduction |  |
| 233589_x_at | C9orf167 | chromosome 9 open reading frame 167 | 1.46 | 9.0 | 6.15 | 2.6 x 10^-7^ | chaperone mediated protein folding requiring cofactor | endoplasmic reticulum |
| 230944_at | --- | --- | 0.55 | 3.4 | 6.15 | 1.5 x 10^-5^ |  |  |
| 202655_at | ARMET | arginine-rich, mutated in early stage tumors | 15.14 | 93.1 | 6.15 | 1.8 x 10^-7^ |  | extracellular region |
| 201983_s_at | EGFR | epidermal growth factor receptor (erythroblastic leukemia viral (v-erb-b) oncogene homolog, avian) | 39.85 | 244.9 | 6.15 | 4.6 x 10^-9^ | ossification | extracellular region |
| 227278_at | --- | --- | 10.19 | 62.6 | 6.15 | 4.5 x 10^-6^ |  |  |
| 214521_at | HES2 | hairy and enhancer of split 2 (Drosophila) | 0.47 | 2.9 | 6.14 | 1.5 x 10^-6^ | transcription | nucleus |
| 211355_x_at | LEPR | leptin receptor | 0.91 | 5.6 | 6.14 | 3.4 x 10^-5^ | angiogenesis | extracellular region |
| 211047_x_at | AP2S1 | adaptor-related protein complex 2, sigma 1 subunit | 18.19 | 111.8 | 6.14 | 1.8 x 10^-7^ | transport | cytosol |
| 225239_at | --- | --- | 22.12 | 135.9 | 6.14 | 1.3 x 10^-3^ |  |  |
| 206173_x_at | GABPB1 | GA binding protein transcription factor, beta subunit 1 | 1.19 | 7.3 | 6.13 | 1.5 x 10^-8^ | transcription | nucleus |
| 232682_at | MREG | melanoregulin | 1.30 | 8.0 | 6.13 | 4.7 x 10^-6^ | melanocyte differentiation | plasma membrane |
| 208636_at | ACTN1 | actinin, alpha 1 | 24.47 | 150.1 | 6.13 | 7.6 x 10^-5^ | regulation of apoptosis | nucleolus |
| 203484_at | SEC61G | Sec61 gamma subunit | 20.17 | 123.7 | 6.13 | 1.3 x 10^-8^ | protein targeting | endoplasmic reticulum |
| 240100_at | --- | --- | 0.09 | 0.6 | 6.13 | 6.7 x 10^-4^ |  |  |
| 217730_at | TMBIM1 | transmembrane BAX inhibitor motif containing 1 | 14.26 | 87.4 | 6.13 | 1.6 x 10^-8^ |  | membrane |
| 226043_at | GPSM1 | G-protein signaling modulator 1 (AGS3-like, C. elegans) | 1.08 | 6.6 | 6.13 | 6.8 x 10^-10^ | signal transduction | Golgi membrane |
| 229070_at | C6orf105 | chromosome 6 open reading frame 105 | 1.46 | 8.9 | 6.12 | 1.8 x 10^-7^ |  | membrane |
| 1553505_at | A2ML1 | alpha-2-macroglobulin-like 1 | 0.16 | 1.0 | 6.12 | 5.8 x 10^-3^ | regulation of endopeptidase activity | extracellular region |
| 213338_at | TMEM158 | transmembrane protein 158 | 0.57 | 3.5 | 6.12 | 1.5 x 10^-5^ |  | membrane |
| 243_g_at | MAP4 | microtubule-associated protein 4 | 7.17 | 43.9 | 6.12 | 8.0 x 10^-9^ | negative regulation of microtubule depolymerization | microtubule |
| 213554_s_at | CDV3 | CDV3 homolog (mouse) | 4.32 | 26.4 | 6.12 | 3.0 x 10^-5^ | cell proliferation | cytoplasm |
| 211936_at | HSPA5 | heat shock 70kDa protein 5 (glucose-regulated protein, 78kDa) | 92.38 | 565.1 | 6.12 | 1.5 x 10^-6^ | anti-apoptosis | nucleus |
| 222229_x_at | RPL26P37 | ribosomal protein L26 pseudogene 37 | 53.76 | 328.7 | 6.11 | 4.6 x 10^-8^ |  |  |
| 206354_at | SLCO1B3 | solute carrier organic anion transporter family, member 1B3 | 0.42 | 2.6 | 6.11 | 2.9 x 10^-3^ | transport | plasma membrane |
| 1555734_x_at | AP1S3 | adaptor-related protein complex 1, sigma 3 subunit | 1.85 | 11.3 | 6.10 | 2.1 x 10^-5^ | transport | Golgi apparatus |
| 223172_s_at | MTP18 | mitochondrial protein 18 kDa | 2.30 | 14.0 | 6.09 | 1.6 x 10^-4^ | apoptosis | mitochondrion |
| 209203_s_at | BICD2 | bicaudal D homolog 2 (Drosophila) | 2.51 | 15.3 | 6.09 | 4.0 x 10^-10^ | transport | cytoplasm |
| 212086_x_at | LMNA | lamin A/C | 7.52 | 45.8 | 6.09 | 3.4 x 10^-6^ | nuclear envelope organization | insoluble fraction |
| 202006_at | PTPN12 | protein tyrosine phosphatase, non-receptor type 12 | 13.28 | 80.8 | 6.09 | 1.3 x 10^-9^ | protein amino acid dephosphorylation | soluble fraction |
| 1555943_at | PGAM5 | phosphoglycerate mutase family member 5 | 0.68 | 4.2 | 6.09 | 8.0 x 10^-6^ |  | nucleus |
| 1563348_at | --- | --- | 0.22 | 1.4 | 6.09 | 7.2 x 10^-5^ |  |  |
| 222781_s_at | C9orf40 | chromosome 9 open reading frame 40 | 1.59 | 9.7 | 6.08 | 4.4 x 10^-7^ |  |  |
| 209135_at | ASPH | aspartate beta-hydroxylase | 11.56 | 70.3 | 6.08 | 2.5 x 10^-7^ | muscle contraction | endoplasmic reticulum |
| 218038_at | ATP5SL | ATP5S-like | 2.02 | 12.3 | 6.08 | 1.5 x 10^-5^ |  |  |
| 212104_s_at | RBM9 | RNA binding motif protein 9 | 6.73 | 40.9 | 6.08 | 8.1 x 10^-10^ | mRNA processing | nucleus |
| 223461_at | TBC1D7 | TBC1 domain family, member 7 | 3.31 | 20.1 | 6.07 | 8.3 x 10^-7^ | regulation of Rab GTPase activity | intracellular |
| 225104_at | ZNF598 | zinc finger protein 598 | 1.42 | 8.6 | 6.07 | 2.8 x 10^-6^ |  | intracellular |
| 219921_s_at | DOCK5 | dedicator of cytokinesis 5 | 0.73 | 4.4 | 6.07 | 4.7 x 10^-7^ |  | cytoplasm |
| 211284_s_at | GRN | granulin | 6.19 | 37.6 | 6.07 | 9.4 x 10^-7^ | signal transduction | extracellular region |
| 202570_s_at | DLGAP4 | discs, large (Drosophila) homolog-associated protein 4 | 2.21 | 13.4 | 6.07 | 6.9 x 10^-6^ | cell-cell signaling | membrane |
| 230031_at | HSPA5 | heat shock 70kDa protein 5 (glucose-regulated protein, 78kDa) | 5.24 | 31.7 | 6.05 | 1.8 x 10^-3^ | anti-apoptosis | nucleus |
| 202369_s_at | TRAM2 | translocation associated membrane protein 2 | 2.80 | 17.0 | 6.05 | 2.8 x 10^-4^ | transport | membrane |
| 202686_s_at | AXL | AXL receptor tyrosine kinase | 5.71 | 34.6 | 6.05 | 4.5 x 10^-5^ | protein amino acid phosphorylation | integral to plasma membrane |
| 204222_s_at | GLIPR1 | GLI pathogenesis-related 1 | 1.75 | 10.6 | 6.05 | 2.5 x 10^-3^ |  | extracellular region |
| 221923_s_at | NPM1 | nucleophosmin (nucleolar phosphoprotein B23, numatrin) | 19.98 | 120.8 | 6.05 | 9.0 x 10^-8^ | nucleosome assembly | nucleus |
| 243807_at | NCOA7 | nuclear receptor coactivator 7 | 0.43 | 2.6 | 6.05 | 8.2 x 10^-6^ | transcription | intracellular |
| 230252_at | LPAR5 | lysophosphatidic acid receptor 5 | 1.16 | 7.0 | 6.04 | 1.4 x 10^-4^ | signal transduction | plasma membrane |
| 226707_at | NAPRT1 | nicotinate phosphoribosyltransferase domain containing 1 | 4.27 | 25.8 | 6.04 | 4.4 x 10^-5^ | response to oxidative stress | cytoplasm |
| 206561_s_at | AKR1B10 | aldo-keto reductase family 1, member B10 (aldose reductase) | 4.24 | 25.6 | 6.04 | 1.7 x 10^-3^ | cellular aldehyde metabolic process | cytoplasm |
| 209890_at | TSPAN5 | tetraspanin 5 | 1.14 | 6.9 | 6.04 | 2.5 x 10^-3^ |  | membrane |
| 214075_at | NENF | neuron derived neurotrophic factor | 1.33 | 8.0 | 6.03 | 3.1 x 10^-7^ | positive regulation of MAPKKK cascade | extracellular region |
| 209909_s_at | TGFB2 | transforming growth factor, beta 2 | 0.75 | 4.6 | 6.03 | 2.9 x 10^-4^ | cell morphogenesis | extracellular region |
| 223378_at | GLIS2 | GLIS family zinc finger 2 | 2.45 | 14.8 | 6.03 | 1.3 x 10^-10^ | transcription | intracellular |
| 218247_s_at | MEX3C | mex-3 homolog C (C. elegans) | 6.43 | 38.8 | 6.02 | 1.2 x 10^-8^ |  | nucleus |
| 243475_at | CBL | Cas-Br-M (murine) ecotropic retroviral transforming sequence | 0.38 | 2.3 | 6.02 | 1.7 x 10^-7^ | cell surface receptor linked signal transduction | nucleus |
| 222412_s_at | SSR3 | signal sequence receptor, gamma (translocon-associated protein gamma) | 4.54 | 27.3 | 6.02 | 5.4 x 10^-8^ | cotranslational protein targeting to membrane | endoplasmic reticulum |
| 225725_at | --- | --- | 2.99 | 18.0 | 6.01 | 3.5 x 10^-4^ |  |  |
| 208803_s_at | SRP72 | signal recognition particle 72kDa | 4.11 | 24.7 | 6.01 | 1.5 x 10^-6^ | protein amino acid phosphorylation | nucleolus |
| 242222_at | LOC440894 | hypothetical protein LOC440894 | 0.37 | 2.2 | 6.01 | 5.6 x 10^-5^ |  |  |
| 236957_at | CDCA2 | cell division cycle associated 2 | 0.46 | 2.8 | 6.01 | 9.7 x 10^-3^ | cell cycle | nucleus |
| 204120_s_at | ADK | adenosine kinase | 0.96 | 5.8 | 6.01 | 1.9 x 10^-5^ | purine ribonucleoside salvage | nucleus |
| 225150_s_at | RTKN | rhotekin | 1.35 | 8.1 | 6.01 | 7.8 x 10^-4^ | apoptosis | intracellular |
| 210735_s_at | CA12 | carbonic anhydrase XII | 2.12 | 12.7 | 6.01 | 3.8 x 10^-4^ | one-carbon compound metabolic process | cytoplasm |
| 200788_s_at | PEA15 | phosphoprotein enriched in astrocytes 15 | 11.56 | 69.4 | 6.00 | 1.1 x 10^-8^ | transport | cytoplasm |
| 217784_at | YKT6 | YKT6 v-SNARE homolog (S. cerevisiae) | 1.57 | 9.4 | 6.00 | 1.4 x 10^-6^ | transport | Golgi membrane |
| 225454_at | CCDC124 | coiled-coil domain containing 124 | 2.09 | 12.6 | 6.00 | 5.6 x 10^-8^ |  |  |
| 216241_s_at | TCEA1 | transcription elongation factor A (SII), 1 | 42.81 | 256.7 | 6.00 | 4.9 x 10^-6^ | transcription | nucleus |
| 41037_at | TEAD4 | TEA domain family member 4 | 0.44 | 2.7 | 6.00 | 4.3 x 10^-7^ | skeletal system development | nucleus |
| 202894_at | EPHB4 | EPH receptor B4 | 3.18 | 19.1 | 5.99 | 9.8 x 10^-8^ | protein amino acid phosphorylation | integral to plasma membrane |
| 212444_at | --- | --- | 6.94 | 41.6 | 5.99 | 6.1 x 10^-6^ |  |  |
| 234932_s_at | CDCP1 | CUB domain containing protein 1 | 1.95 | 11.7 | 5.99 | 3.9 x 10^-6^ |  | extracellular region |
| 225039_at | RPE | ribulose-5-phosphate-3-epimerase | 4.63 | 27.7 | 5.99 | 4.9 x 10^-8^ | carbohydrate metabolic process | cytosol |
| 210396_s_at | BOLA2 /// LOC440354 /// LOC595101 | bolA homolog 2 (E. coli) /// PI-3-kinase-related kinase SMG-1 pseudogene /// PI-3-kinase-related kinase SMG-1 pseudogene | 9.98 | 59.7 | 5.98 | 4.8 x 10^-6^ | amino acid transport | cytoplasm |
| 224866_at | FAR1 | fatty acyl CoA reductase 1 | 7.21 | 43.2 | 5.98 | 3.7 x 10^-7^ | lipid metabolic process | peroxisome |
| 209054_s_at | WHSC1 | Wolf-Hirschhorn syndrome candidate 1 | 2.37 | 14.2 | 5.98 | 6.5 x 10^-6^ | transcription | nucleus |
| 230492_s_at | RP5-1022P6.2 | hypothetical protein KIAA1434 | 3.75 | 22.4 | 5.98 | 1.2 x 10^-4^ | carbohydrate metabolic process |  |
| 221803_s_at | NRBF2 | nuclear receptor binding factor 2 | 5.11 | 30.5 | 5.97 | 3.0 x 10^-7^ | transcription | nucleus |
| 214487_s_at | RAP2A /// RAP2B | RAP2A, member of RAS oncogene family /// RAP2B, member of RAS oncogene family | 0.96 | 5.7 | 5.97 | 1.8 x 10^-6^ | signal transduction | intracellular |
| 224610_at | SNHG1 | small nucleolar RNA host gene 1 (non-protein coding) | 11.17 | 66.6 | 5.96 | 1.2 x 10^-5^ |  |  |
| 203963_at | CA12 | carbonic anhydrase XII | 6.51 | 38.8 | 5.96 | 2.5 x 10^-3^ | one-carbon compound metabolic process | cytoplasm |
| 213010_at | PRKCDBP | protein kinase C, delta binding protein | 3.09 | 18.4 | 5.96 | 5.1 x 10^-4^ | cell cycle |  |
| 217492_s_at | PTEN /// PTENP1 | phosphatase and tensin homolog /// phosphatase and tensin homolog pseudogene 1 | 10.27 | 61.2 | 5.96 | 7.9 x 10^-5^ | regulation of cyclin-dependent protein kinase activity | nucleus |
| 217027_x_at | KPNB1 | karyopherin (importin) beta 1 | 1.47 | 8.8 | 5.95 | 5.8 x 10^-6^ | protein import into nucleus, docking | nucleus |
| 201655_s_at | HSPG2 | heparan sulfate proteoglycan 2 | 4.50 | 26.8 | 5.95 | 1.4 x 10^-5^ | endochondral ossification | extracellular region |
| 31637_s_at | NR1D1 /// THRA | nuclear receptor subfamily 1, group D, member 1 /// thyroid hormone receptor, alpha (erythroblastic leukemia viral (v-erb-a) oncogene homolog, avian) | 2.08 | 12.4 | 5.95 | 1.3 x 10^-3^ | cartilage condensation | nucleus |
| 214091_s_at | GPX3 | glutathione peroxidase 3 (plasma) | 9.84 | 58.6 | 5.95 | 9.7 x 10^-7^ | glutathione metabolic process | extracellular region |
| 213696_s_at | MED8 | mediator complex subunit 8 | 3.15 | 18.7 | 5.95 | 5.2 x 10^-9^ | transcription | mediator complex |
| 221414_s_at | DEFB126 | defensin, beta 126 | 0.08 | 0.5 | 5.94 | 1.3 x 10^-4^ | defense response | extracellular region |
| 210105_s_at | FYN | FYN oncogene related to SRC, FGR, YES | 4.12 | 24.5 | 5.94 | 7.6 x 10^-6^ | neuron migration | endosome |
| 218077_s_at | ZDHHC3 | zinc finger, DHHC-type containing 3 | 2.10 | 12.5 | 5.94 | 2.6 x 10^-8^ |  | Golgi membrane |
| 203184_at | FBN2 | fibrillin 2 | 1.71 | 10.2 | 5.93 | 4.5 x 10^-3^ | anatomical structure morphogenesis | extracellular region |
| 202756_s_at | GPC1 | glypican 1 | 4.71 | 27.9 | 5.93 | 3.1 x 10^-7^ |  | extracellular region |
| 219878_s_at | KLF13 | Kruppel-like factor 13 | 0.64 | 3.8 | 5.93 | 3.0 x 10^-3^ | transcription | intracellular |
| 209453_at | SLC9A1 | solute carrier family 9 (sodium/hydrogen exchanger), member 1 | 2.84 | 16.9 | 5.93 | 7.1 x 10^-5^ | transport | membrane fraction |
| 231001_at | FIBIN | fin bud initiation factor homolog (zebrafish) | 0.41 | 2.4 | 5.93 | 4.9 x 10^-3^ |  | extracellular region |
| 201015_s_at | JUP | junction plakoglobin | 14.33 | 84.9 | 5.92 | 1.3 x 10^-7^ | cell adhesion | membrane fraction |
| 226711_at | FOXN2 | forkhead box N2 | 5.48 | 32.4 | 5.92 | 1.7 x 10^-6^ | transcription | nucleus |
| 1553995_a_at | NT5E | 5'-nucleotidase, ecto (CD73) | 2.21 | 13.1 | 5.92 | 2.6 x 10^-4^ | purine nucleotide biosynthetic process | membrane fraction |
| 218964_at | ARID3B | AT rich interactive domain 3B (BRIGHT-like) | 0.59 | 3.5 | 5.91 | 5.2 x 10^-7^ | transcription | intracellular |
| 226088_at | ZDHHC12 | zinc finger, DHHC-type containing 12 | 1.25 | 7.4 | 5.91 | 3.3 x 10^-6^ |  | membrane |
| 216380_x_at | RPS28P6 | ribosomal protein S28 pseudogene 6 | 7.39 | 43.7 | 5.91 | 2.0 x 10^-6^ |  |  |
| 219991_at | SLC2A9 | solute carrier family 2 (facilitated glucose transporter), member 9 | 2.47 | 14.6 | 5.91 | 2.6 x 10^-6^ | transport | membrane |
| 208726_s_at | EIF2S2 | eukaryotic translation initiation factor 2, subunit 2 beta, 38kDa | 45.52 | 268.8 | 5.90 | 6.0 x 10^-12^ | translation | cytosol |
| 229292_at | EPB41L5 | erythrocyte membrane protein band 4.1 like 5 | 4.82 | 28.5 | 5.90 | 1.8 x 10^-4^ |  | cytoplasm |
| 207121_s_at | MAPK6 | mitogen-activated protein kinase 6 | 27.69 | 163.3 | 5.90 | 3.8 x 10^-11^ | protein amino acid phosphorylation | cytoplasm |
| 212698_s_at | 40066 | septin 10 | 21.01 | 123.9 | 5.90 | 8.7 x 10^-10^ | cell cycle | nucleus |
| 209836_x_at | BOLA2 /// BOLA2B | bolA homolog 2 (E. coli) /// bolA homolog 2B (E. coli) | 6.09 | 35.9 | 5.90 | 1.2 x 10^-9^ |  |  |
| 221580_s_at | TAF1D | TATA box binding protein (TBP)-associated factor, RNA polymerase I, D, 41kDa | 13.37 | 78.9 | 5.90 | 1.1 x 10^-8^ | transcription | nucleus |
| 32699_s_at | PVR | poliovirus receptor | 0.66 | 3.9 | 5.90 | 3.0 x 10^-4^ | positive regulation of natural killer cell mediated cytotoxicity directed against tumor cell target | extracellular region |
| 1555301_a_at | DIP2A | DIP2 disco-interacting protein 2 homolog A (Drosophila) | 0.74 | 4.3 | 5.90 | 6.6 x 10^-5^ | multicellular organismal development | nucleus |
| 213457_at | MFHAS1 | malignant fibrous histiocytoma amplified sequence 1 | 2.47 | 14.5 | 5.89 | 2.9 x 10^-5^ |  |  |
| 219275_at | PDCD5 | programmed cell death 5 | 3.65 | 21.5 | 5.88 | 1.3 x 10^-10^ | apoptosis |  |
| 1555259_at | ZAK | sterile alpha motif and leucine zipper containing kinase AZK | 0.55 | 3.2 | 5.88 | 1.8 x 10^-6^ | cell cycle checkpoint | nucleus |
| 212075_s_at | CSNK2A1 | casein kinase 2, alpha 1 polypeptide | 7.57 | 44.5 | 5.88 | 5.1 x 10^-7^ | regulation of cell growth | nucleus |
| 235311_at | FKBP14 | FK506 binding protein 14, 22 kDa | 0.74 | 4.3 | 5.88 | 2.2 x 10^-6^ | protein folding | endoplasmic reticulum |
| 222783_s_at | SMOC1 | SPARC related modular calcium binding 1 | 0.25 | 1.4 | 5.88 | 4.8 x 10^-4^ |  | extracellular region |
| 213984_at | PDS5A | PDS5, regulator of cohesion maintenance, homolog A (S. cerevisiae) | 0.96 | 5.6 | 5.88 | 6.2 x 10^-6^ | cell cycle | nucleus |
| 226609_at | DCBLD1 | discoidin, CUB and LCCL domain containing 1 | 2.47 | 14.5 | 5.88 | 1.1 x 10^-5^ | cell adhesion | membrane |
| 229865_at | FNDC3B | fibronectin type III domain containing 3B | 0.78 | 4.6 | 5.88 | 5.9 x 10^-5^ |  | endoplasmic reticulum |
| 202146_at | IFRD1 | interferon-related developmental regulator 1 | 8.73 | 51.3 | 5.87 | 5.0 x 10^-3^ | multicellular organismal development | nucleus |
| 218055_s_at | WDR41 | WD repeat domain 41 | 4.80 | 28.2 | 5.87 | 1.4 x 10^-5^ |  |  |
| 214696_at | C17orf91 | chromosome 17 open reading frame 91 | 13.41 | 78.7 | 5.87 | 6.2 x 10^-3^ |  |  |
| 211966_at | COL4A2 | collagen, type IV, alpha 2 | 0.26 | 1.5 | 5.87 | 8.6 x 10^-4^ | negative regulation of angiogenesis | extracellular region |
| 238568_s_at | --- | --- | 0.68 | 4.0 | 5.87 | 7.4 x 10^-3^ |  |  |
| 218705_s_at | SNX24 | sorting nexin 24 | 2.95 | 17.3 | 5.87 | 3.9 x 10^-6^ | transport |  |
| 202067_s_at | LDLR | low density lipoprotein receptor | 3.23 | 18.9 | 5.87 | 1.4 x 10^-6^ | protein amino acid O-linked glycosylation | endosome |
| 238336_s_at | DNAJC21 | DnaJ (Hsp40) homolog, subfamily C, member 21 | 0.15 | 0.9 | 5.86 | 1.5 x 10^-4^ |  | intracellular |
| 235197_s_at | OSTM1 | osteopetrosis associated transmembrane protein 1 | 1.55 | 9.1 | 5.86 | 2.1 x 10^-7^ | osteoclast differentiation | cytosol |
| 209631_s_at | GPR37 | G protein-coupled receptor 37 (endothelin receptor type B-like) | 0.50 | 3.0 | 5.86 | 8.5 x 10^-7^ | signal transduction | endoplasmic reticulum |
| 206170_at | ADRB2 | adrenergic, beta-2-, receptor, surface | 6.57 | 38.4 | 5.85 | 2.6 x 10^-4^ | activation of MAPK activity | membrane fraction |
| 212556_at | SCRIB | scribbled homolog (Drosophila) | 5.32 | 31.1 | 5.85 | 3.6 x 10^-7^ |  | cytoplasm |
| 203297_s_at | JARID2 | jumonji, AT rich interactive domain 2 | 4.64 | 27.2 | 5.85 | 7.8 x 10^-7^ | negative regulation of transcription from RNA polymerase II promoter | intracellular |
| 225686_at | FAM33A | family with sequence similarity 33, member A | 2.31 | 13.5 | 5.85 | 2.2 x 10^-6^ | cell cycle | condensed chromosome kinetochore |
| 1554213_at | ARHGEF10 | Rho guanine nucleotide exchange factor (GEF) 10 | 0.39 | 2.3 | 5.85 | 6.9 x 10^-4^ | regulation of Rho protein signal transduction | intracellular |
| 201751_at | JOSD1 | Josephin domain containing 1 | 9.98 | 58.3 | 5.84 | 6.4 x 10^-7^ |  | nucleus |
| 210005_at | GART | phosphoribosylglycinamide formyltransferase, phosphoribosylglycinamide synthetase, phosphoribosylaminoimidazole synthetase | 1.22 | 7.1 | 5.84 | 6.2 x 10^-8^ | purine nucleotide biosynthetic process | nucleus |
| 202754_at | R3HDM1 | R3H domain containing 1 | 4.81 | 28.1 | 5.84 | 1.2 x 10^-6^ |  |  |
| 212640_at | PTPLB | protein tyrosine phosphatase-like (proline instead of catalytic arginine), member b | 9.75 | 56.9 | 5.84 | 2.9 x 10^-6^ |  | endoplasmic reticulum |
| 225787_at | UBE2F | ubiquitin-conjugating enzyme E2F (putative) | 8.90 | 51.9 | 5.83 | 1.3 x 10^-5^ | modification-dependent protein catabolic process |  |
| 200931_s_at | VCL | vinculin | 9.57 | 55.8 | 5.83 | 1.5 x 10^-6^ | cell motion | cytoplasm |
| 226915_s_at | ARPC5L | actin related protein 2/3 complex, subunit 5-like | 8.96 | 52.2 | 5.83 | 4.8 x 10^-10^ | regulation of actin filament polymerization | cytoplasm |
| 239186_at | MGC39372 | hypothetical protein MGC39372 | 0.35 | 2.1 | 5.83 | 3.1 x 10^-3^ |  |  |
| 1558212_at | FLJ35024 | hypothetical LOC401491 | 1.23 | 7.2 | 5.82 | 2.3 x 10^-4^ |  |  |
| 215167_at | MED14 | mediator complex subunit 14 | 0.55 | 3.2 | 5.82 | 6.7 x 10^-4^ | transcription | mediator complex |
| 204920_at | CPS1 | carbamoyl-phosphate synthetase 1, mitochondrial | 0.56 | 3.3 | 5.82 | 2.5 x 10^-4^ | urea cycle | intracellular |
| 201319_at | MYL12A | myosin, light chain 12A, regulatory, non-sarcomeric | 19.72 | 114.7 | 5.82 | 4.3 x 10^-6^ |  | myosin complex |
| 215464_s_at | TAX1BP3 | Tax1 (human T-cell leukemia virus type I) binding protein 3 | 8.29 | 48.1 | 5.81 | 4.8 x 10^-5^ | transport | nucleus |
| 221009_s_at | ANGPTL4 | angiopoietin-like 4 | 0.78 | 4.6 | 5.80 | 5.2 x 10^-3^ | angiogenesis | extracellular region |
| 226651_at | HOMER1 | homer homolog 1 (Drosophila) | 2.22 | 12.9 | 5.80 | 1.5 x 10^-6^ | skeletal muscle contraction | membrane fraction |
| 218618_s_at | FNDC3B | fibronectin type III domain containing 3B | 12.21 | 70.9 | 5.80 | 9.3 x 10^-11^ | positive regulation of fat cell differentiation | endoplasmic reticulum |
| 201595_s_at | ZC3H15 | zinc finger CCCH-type containing 15 | 13.64 | 79.1 | 5.80 | 1.4 x 10^-7^ | cytokine-mediated signaling pathway | nucleus |
| 235759_at | --- | --- | 0.39 | 2.3 | 5.80 | 1.5 x 10^-3^ |  |  |
| 219024_at | PLEKHA1 | pleckstrin homology domain containing, family A (phosphoinositide binding specific) member 1 | 5.12 | 29.7 | 5.79 | 1.9 x 10^-8^ |  | nucleus |
| 224796_at | ASAP1 | ArfGAP with SH3 domain, ankyrin repeat and PH domain 1 | 3.04 | 17.6 | 5.79 | 9.7 x 10^-9^ | regulation of ARF GTPase activity | cytoplasm |
| 218772_x_at | TMEM38B | transmembrane protein 38B | 2.24 | 13.0 | 5.79 | 3.6 x 10^-3^ | transport | endoplasmic reticulum |
| 217211_at | --- | --- | 1.66 | 9.6 | 5.79 | 5.9 x 10^-7^ |  |  |
| 218828_at | PLSCR3 | phospholipid scramblase 3 | 1.83 | 10.6 | 5.78 | 1.3 x 10^-5^ | phospholipid scrambling | plasma membrane |
| 220058_at | C17orf39 | chromosome 17 open reading frame 39 | 0.77 | 4.4 | 5.78 | 9.9 x 10^-6^ |  |  |
| 238613_at | ZAK | sterile alpha motif and leucine zipper containing kinase AZK | 1.18 | 6.8 | 5.78 | 2.1 x 10^-10^ | cell cycle checkpoint | nucleus |
| 239668_at | --- | --- | 0.27 | 1.6 | 5.78 | 2.6 x 10^-5^ |  |  |
| 222982_x_at | SLC38A2 | solute carrier family 38, member 2 | 66.63 | 384.5 | 5.77 | 8.8 x 10^-10^ | transport | plasma membrane |
| 201828_x_at | FAM127A | family with sequence similarity 127, member A | 6.53 | 37.7 | 5.77 | 1.3 x 10^-7^ |  | plasma membrane |
| 202579_x_at | HMGN4 | high mobility group nucleosomal binding domain 4 | 13.78 | 79.5 | 5.77 | 1.6 x 10^-9^ |  | chromatin |
| 209190_s_at | DIAPH1 | diaphanous homolog 1 (Drosophila) | 8.22 | 47.4 | 5.77 | 4.2 x 10^-6^ | sensory perception of sound | cytoplasm |
| 212739_s_at | NME4 | non-metastatic cells 4, protein expressed in | 2.74 | 15.8 | 5.77 | 3.6 x 10^-6^ | GTP biosynthetic process | mitochondrion |
| 202314_at | CYP51A1 | cytochrome P450, family 51, subfamily A, polypeptide 1 | 11.17 | 64.3 | 5.76 | 1.5 x 10^-6^ | steroid biosynthetic process | endoplasmic reticulum |
| 233757_x_at | --- | --- | 1.93 | 11.1 | 5.75 | 9.4 x 10^-6^ |  |  |
| 208801_at | SRP72 | signal recognition particle 72kDa | 18.45 | 106.1 | 5.75 | 4.1 x 10^-10^ | protein amino acid phosphorylation | nucleolus |
| 243000_at | CDK6 | cyclin-dependent kinase 6 | 1.31 | 7.5 | 5.74 | 8.9 x 10^-7^ | G1 phase of mitotic cell cycle | cyclin-dependent protein kinase holoenzyme complex |
| 209181_s_at | RABGGTB | Rab geranylgeranyltransferase, beta subunit | 24.36 | 139.9 | 5.74 | 4.2 x 10^-6^ | protein modification process |  |
| 200998_s_at | CKAP4 | cytoskeleton-associated protein 4 | 9.28 | 53.3 | 5.74 | 1.7 x 10^-7^ |  | membrane fraction |
| 204255_s_at | VDR | vitamin D (1,25- dihydroxyvitamin D3) receptor | 1.58 | 9.1 | 5.73 | 4.3 x 10^-7^ | skeletal system development | nucleus |
| 218647_s_at | YRDC | yrdC domain containing (E. coli) | 5.07 | 29.1 | 5.73 | 3.5 x 10^-6^ |  | mitochondrion |
| 204194_at | BACH1 | BTB and CNC homology 1, basic leucine zipper transcription factor 1 | 5.34 | 30.6 | 5.73 | 1.2 x 10^-7^ | transcription | nucleus |
| 1555501_s_at | RSRC1 | arginine/serine-rich coiled-coil 1 | 1.42 | 8.1 | 5.72 | 5.7 x 10^-8^ | alternative nuclear mRNA splicing, via spliceosome | nucleus |
| 52164_at | C11orf24 | chromosome 11 open reading frame 24 | 2.92 | 16.7 | 5.72 | 2.1 x 10^-6^ |  | membrane |
| 1554010_at | NDST1 | N-deacetylase/N-sulfotransferase (heparan glucosaminyl) 1 | 0.71 | 4.1 | 5.72 | 2.9 x 10^-5^ | inflammatory response | Golgi membrane |
| 209210_s_at | FERMT2 | fermitin family homolog 2 (Drosophila) | 5.94 | 33.9 | 5.71 | 2.4 x 10^-5^ | cell adhesion | stress fiber |
| 219437_s_at | ANKRD11 | ankyrin repeat domain 11 | 2.61 | 14.9 | 5.71 | 1.0 x 10^-9^ |  | nucleus |
| 223079_s_at | GLS | glutaminase | 1.69 | 9.7 | 5.70 | 2.9 x 10^-6^ | glutamine metabolic process | mitochondrion |
| 207738_s_at | NCKAP1 | NCK-associated protein 1 | 26.78 | 152.6 | 5.70 | 1.4 x 10^-6^ | apoptosis | plasma membrane |
| 203085_s_at | TGFB1 | transforming growth factor, beta 1 | 2.61 | 14.9 | 5.70 | 1.2 x 10^-4^ | skeletal system development | extracellular region |
| 220122_at | MCTP1 | multiple C2 domains, transmembrane 1 | 2.38 | 13.5 | 5.69 | 3.1 x 10^-6^ | calcium-mediated signaling | membrane fraction |
| 230265_at | SEL1L | Sel-1 suppressor of lin-12-like (C. elegans) | 2.85 | 16.2 | 5.69 | 4.0 x 10^-4^ | Notch signaling pathway | endoplasmic reticulum |
| 216005_at | TNC | Tenascin C | 0.89 | 5.0 | 5.68 | 7.2 x 10^-3^ | cell adhesion | extracellular region |
| 203405_at | PSMG1 | proteasome (prosome, macropain) assembly chaperone 1 | 7.53 | 42.8 | 5.68 | 1.2 x 10^-7^ | proteasome assembly | cytoplasm |
| 219121_s_at | ESRP1 | epithelial splicing regulatory protein 1 | 17.86 | 101.5 | 5.68 | 1.4 x 10^-5^ | regulation of RNA splicing | nucleus |
| 1553601_a_at | TMIE | transmembrane inner ear | 0.18 | 1.0 | 5.68 | 2.8 x 10^-3^ | sensory perception of sound | membrane |
| 230051_at | C10orf47 | chromosome 10 open reading frame 47 | 3.39 | 19.2 | 5.68 | 1.5 x 10^-5^ |  |  |
| 226087_at | LZIC | leucine zipper and CTNNBIP1 domain containing | 4.43 | 25.2 | 5.68 | 2.7 x 10^-6^ |  |  |
| 241966_at | MYO5A | myosin VA (heavy chain 12, myoxin) | 0.38 | 2.1 | 5.68 | 3.8 x 10^-6^ | ubiquitin-dependent protein catabolic process | ruffle |
| 214543_x_at | QKI | quaking homolog, KH domain RNA binding (mouse) | 2.46 | 13.9 | 5.67 | 3.6 x 10^-9^ | mRNA processing | nucleus |
| 201819_at | SCARB1 | scavenger receptor class B, member 1 | 0.91 | 5.2 | 5.67 | 5.8 x 10^-4^ | transport | plasma membrane |
| 217739_s_at | NAMPT | nicotinamide phosphoribosyltransferase | 14.19 | 80.5 | 5.67 | 2.5 x 10^-5^ | signal transduction | cytoplasm |
| 215717_s_at | FBN2 | fibrillin 2 | 0.19 | 1.1 | 5.67 | 1.1 x 10^-4^ | anatomical structure morphogenesis | extracellular region |
| 206412_at | FER | fer (fps/fes related) tyrosine kinase | 1.51 | 8.6 | 5.67 | 5.0 x 10^-7^ | protein amino acid phosphorylation | nucleus |
| 223373_s_at | PLA2G12A | phospholipase A2, group XIIA | 4.33 | 24.5 | 5.67 | 6.3 x 10^-5^ | phospholipid metabolic process | extracellular region |
| 202345_s_at | FABP5 | fatty acid binding protein 5 (psoriasis-associated) | 15.97 | 90.4 | 5.66 | 1.5 x 10^-3^ | lipid metabolic process | cytoplasm |
| 1554462_a_at | DNAJB9 | DnaJ (Hsp40) homolog, subfamily B, member 9 | 7.24 | 41.0 | 5.66 | 5.0 x 10^-4^ | protein folding | nucleus |
| 220199_s_at | AIDA | axin interactor, dorsalization associated | 7.72 | 43.7 | 5.66 | 1.1 x 10^-7^ | multicellular organismal development |  |
| 223376_s_at | BRI3 | brain protein I3 | 19.36 | 109.4 | 5.65 | 4.7 x 10^-5^ |  | membrane |
| 209595_at | GTF2F2 | general transcription factor IIF, polypeptide 2, 30kDa | 2.76 | 15.6 | 5.65 | 7.4 x 10^-8^ | nuclear mRNA splicing, via spliceosome | nucleus |
| 224985_at | NRAS | neuroblastoma RAS viral (v-ras) oncogene homolog | 10.67 | 60.3 | 5.65 | 4.1 x 10^-7^ | endocytosis | Golgi membrane |
| 225378_at | VPS37A | vacuolar protein sorting 37 homolog A (S. cerevisiae) | 4.35 | 24.6 | 5.65 | 2.4 x 10^-7^ | transport | nucleus |
| 203331_s_at | INPP5D | inositol polyphosphate-5-phosphatase, 145kDa | 0.33 | 1.9 | 5.65 | 1.7 x 10^-4^ | phosphate metabolic process | cytoplasm |
| 200870_at | STRAP | serine/threonine kinase receptor associated protein | 27.70 | 156.3 | 5.64 | 8.4 x 10^-9^ | mRNA processing | nucleus |
| 1559827_at | LOC401074 | hypothetical LOC401074 | 2.81 | 15.8 | 5.63 | 4.3 x 10^-3^ |  |  |
| 226350_at | CHML | choroideremia-like (Rab escort protein 2) | 2.21 | 12.5 | 5.63 | 3.3 x 10^-6^ | intracellular protein transport | Rab-protein geranylgeranyltransferase complex |
| 242283_at | DNAH14 | dynein, axonemal, heavy chain 14 | 0.92 | 5.2 | 5.62 | 3.2 x 10^-7^ | RNA-dependent DNA replication | microtubule |
| 1554364_at | PPP2R5C | protein phosphatase 2, regulatory subunit B', gamma isoform | 0.20 | 1.1 | 5.62 | 2.4 x 10^-4^ | signal transduction | protein phosphatase type 2A complex |
| 219032_x_at | OPN3 | opsin 3 | 4.73 | 26.6 | 5.62 | 4.7 x 10^-7^ | signal transduction | integral to plasma membrane |
| 244115_at | FAM126A | family with sequence similarity 126, member A | 0.17 | 1.0 | 5.62 | 2.9 x 10^-5^ |  | cytoplasm |
| 225080_at | MYO1C | myosin IC | 4.83 | 27.1 | 5.62 | 2.1 x 10^-10^ | transport | stress fiber |
| 222173_s_at | TBC1D2 | TBC1 domain family, member 2 | 3.46 | 19.4 | 5.62 | 9.0 x 10^-6^ | regulation of Rab GTPase activity | intracellular |
| 224725_at | MIB1 | mindbomb homolog 1 (Drosophila) | 5.60 | 31.4 | 5.62 | 6.2 x 10^-5^ | blood vessel development | cytoplasm |
| 222778_s_at | WHSC1 | Wolf-Hirschhorn syndrome candidate 1 | 1.12 | 6.3 | 5.62 | 1.2 x 10^-4^ | transcription | nucleus |
| 202933_s_at | YES1 | v-yes-1 Yamaguchi sarcoma viral oncogene homolog 1 | 13.05 | 73.1 | 5.60 | 5.4 x 10^-7^ | protein modification process | membrane fraction |
| 201272_at | AKR1B1 | aldo-keto reductase family 1, member B1 (aldose reductase) | 10.48 | 58.7 | 5.60 | 6.1 x 10^-5^ | carbohydrate metabolic process | extracellular space |
| 221437_s_at | MRPS15 | mitochondrial ribosomal protein S15 | 5.39 | 30.2 | 5.60 | 4.7 x 10^-10^ | translation | intracellular |
| 227042_at | YDJC | YdjC homolog (bacterial) | 2.14 | 11.9 | 5.59 | 1.5 x 10^-7^ |  |  |
| 232084_at | SGTB | small glutamine-rich tetratricopeptide repeat (TPR)-containing, beta | 0.33 | 1.8 | 5.59 | 3.3 x 10^-5^ |  |  |
| 32811_at | MYO1C | myosin IC | 2.73 | 15.2 | 5.59 | 8.6 x 10^-11^ | transport | stress fiber |
| 218239_s_at | GTPBP4 | GTP binding protein 4 | 8.64 | 48.3 | 5.59 | 8.5 x 10^-7^ | regulation of cyclin-dependent protein kinase activity | nucleus |
| 224950_at | PTGFRN | prostaglandin F2 receptor negative regulator | 3.39 | 18.9 | 5.58 | 8.4 x 10^-6^ |  | endoplasmic reticulum |
| 212846_at | RRP1B | ribosomal RNA processing 1 homolog B (S. cerevisiae) | 9.36 | 52.3 | 5.58 | 3.6 x 10^-6^ | rRNA processing | nucleus |
| 202684_s_at | RNMT | RNA (guanine-7-) methyltransferase | 0.74 | 4.1 | 5.58 | 5.1 x 10^-4^ | mRNA capping | nucleus |
| 230316_at | SEC14L2 | SEC14-like 2 (S. cerevisiae) | 0.54 | 3.0 | 5.58 | 9.2 x 10^-4^ | transcription | intracellular |
| 212355_at | KIAA0323 | KIAA0323 | 5.20 | 29.0 | 5.58 | 5.6 x 10^-5^ |  |  |
| 217266_at | RPL15P22 | ribosomal protein L15 pseudogene 22 | 7.11 | 39.7 | 5.58 | 6.4 x 10^-7^ |  |  |
| 208802_at | SRP72 | signal recognition particle 72kDa | 4.90 | 27.3 | 5.58 | 1.5 x 10^-6^ | protein amino acid phosphorylation | nucleolus |
| 211846_s_at | PVRL1 | poliovirus receptor-related 1 (herpesvirus entry mediator C) | 0.55 | 3.0 | 5.58 | 4.0 x 10^-6^ | lens morphogenesis in camera-type eye | extracellular region |
| 216483_s_at | C19orf10 | chromosome 19 open reading frame 10 | 8.82 | 49.2 | 5.57 | 1.7 x 10^-5^ | positive regulation of cell proliferation | extracellular region |
| 209787_s_at | HMGN4 | high mobility group nucleosomal binding domain 4 | 13.00 | 72.4 | 5.57 | 6.8 x 10^-10^ |  | chromatin |
| 221156_x_at | CCPG1 | cell cycle progression 1 | 3.58 | 20.0 | 5.57 | 3.8 x 10^-3^ | cell cycle | membrane |
| 218392_x_at | SFXN1 | sideroflexin 1 | 2.80 | 15.6 | 5.57 | 2.8 x 10^-6^ | transport | mitochondrion |
| 201921_at | GNG10 | guanine nucleotide binding protein (G protein), gamma 10 | 19.76 | 110.0 | 5.57 | 1.0 x 10^-8^ | signal transduction | heterotrimeric G-protein complex |
| 1558682_at | HMGA2 | high mobility group AT-hook 2 | 0.30 | 1.7 | 5.57 | 1.3 x 10^-4^ | establishment or maintenance of chromatin architecture | nuclear chromosome |
| 201516_at | SRM | spermidine synthase | 2.95 | 16.4 | 5.56 | 1.4 x 10^-6^ | spermidine biosynthetic process |  |
| 204078_at | SC65 | synaptonemal complex protein SC65 | 0.94 | 5.2 | 5.56 | 1.1 x 10^-7^ | synaptonemal complex assembly | condensed nuclear chromosome |
| 215084_s_at | LRRC42 | leucine rich repeat containing 42 | 4.61 | 25.6 | 5.56 | 4.9 x 10^-8^ |  |  |
| 1570588_at | --- | --- | 0.87 | 4.8 | 5.56 | 1.2 x 10^-6^ |  |  |
| 240517_at | CBS | cystathionine-beta-synthase | 0.25 | 1.4 | 5.56 | 4.4 x 10^-3^ | cysteine metabolic process | nucleus |
| 217995_at | SQRDL | sulfide quinone reductase-like (yeast) | 23.92 | 133.0 | 5.56 | 1.1 x 10^-10^ | oxidation reduction | mitochondrion |
| 218156_s_at | TSR1 | TSR1, 20S rRNA accumulation, homolog (S. cerevisiae) | 3.77 | 20.9 | 5.56 | 1.8 x 10^-5^ | ribosome biogenesis | nucleus |
| 208639_x_at | PDIA6 | protein disulfide isomerase family A, member 6 | 32.60 | 181.0 | 5.55 | 3.4 x 10^-8^ | protein folding | endoplasmic reticulum |
| 207668_x_at | PDIA6 | protein disulfide isomerase family A, member 6 | 35.13 | 195.0 | 5.55 | 5.0 x 10^-9^ | protein folding | endoplasmic reticulum |
| 214121_x_at | PDLIM7 | PDZ and LIM domain 7 (enigma) | 0.59 | 3.3 | 5.55 | 1.2 x 10^-4^ | ossification | cytoplasm |
| 205904_at | MICA | MHC class I polypeptide-related sequence A | 2.50 | 13.9 | 5.55 | 4.8 x 10^-3^ | stimulatory C-type lectin receptor signaling pathway | extracellular space |
| 244623_at | KCNQ5 | potassium voltage-gated channel, KQT-like subfamily, member 5 | 0.34 | 1.9 | 5.55 | 6.4 x 10^-3^ | protein complex assembly | voltage-gated potassium channel complex |
| 215058_at | DENND5B | DENN/MADD domain containing 5B | 0.14 | 0.8 | 5.55 | 6.5 x 10^-3^ |  | membrane |
| 209441_at | RHOBTB2 | Rho-related BTB domain containing 2 | 0.35 | 1.9 | 5.55 | 2.2 x 10^-3^ | small GTPase mediated signal transduction | intracellular |
| 209154_at | TAX1BP3 | Tax1 (human T-cell leukemia virus type I) binding protein 3 | 35.05 | 194.3 | 5.54 | 2.1 x 10^-6^ | Rho protein signal transduction | nucleus |
| 202352_s_at | PSMD12 | proteasome (prosome, macropain) 26S subunit, non-ATPase, 12 | 12.08 | 66.9 | 5.54 | 6.8 x 10^-9^ | anaphase-promoting complex-dependent proteasomal ubiquitin-dependent protein catabolic process | cytosol |
| 238598_s_at | --- | --- | 0.56 | 3.1 | 5.54 | 4.6 x 10^-4^ |  |  |
| 217313_at | --- | --- | 2.06 | 11.4 | 5.54 | 1.6 x 10^-5^ |  |  |
| 202525_at | PRSS8 | protease, serine, 8 | 4.23 | 23.4 | 5.54 | 1.9 x 10^-6^ | proteolysis | extracellular region |
| 201380_at | CRTAP | cartilage associated protein | 2.75 | 15.2 | 5.54 | 2.5 x 10^-8^ |  | extracellular region |
| 212658_at | LHFPL2 | lipoma HMGIC fusion partner-like 2 | 1.95 | 10.8 | 5.54 | 9.0 x 10^-6^ |  | membrane |
| 226799_at | FGD6 | FYVE, RhoGEF and PH domain containing 6 | 0.83 | 4.6 | 5.53 | 6.0 x 10^-4^ | cytoskeleton organization | ruffle |
| 1556834_at | --- | --- | 0.37 | 2.0 | 5.53 | 5.8 x 10^-5^ |  |  |
| 219856_at | C1orf116 | chromosome 1 open reading frame 116 | 5.32 | 29.4 | 5.53 | 8.2 x 10^-8^ |  | cytoplasm |
| 225388_at | TSPAN5 | tetraspanin 5 | 1.09 | 6.0 | 5.53 | 1.4 x 10^-4^ |  | membrane |
| 217559_at | RPL10L | ribosomal protein L10-like | 0.35 | 1.9 | 5.53 | 3.0 x 10^-4^ | translation | intracellular |
| 218421_at | CERK | ceramide kinase | 2.85 | 15.8 | 5.53 | 1.1 x 10^-7^ | ceramide metabolic process | integral to membrane of membrane fraction |
| 226214_at | GDE1 | glycerophosphodiester phosphodiesterase 1 | 12.73 | 70.3 | 5.53 | 6.3 x 10^-5^ | glycerol metabolic process | cytoplasm |
| 222465_at | RSL24D1 | ribosomal L24 domain containing 1 | 19.95 | 110.1 | 5.52 | 4.1 x 10^-6^ | translation | intracellular |
| 243446_at | JUB | Jub, ajuba homolog (Xenopus laevis) | 0.26 | 1.4 | 5.52 | 2.0 x 10^-3^ | cell cycle | nucleus |
| 218131_s_at | GATAD2A | GATA zinc finger domain containing 2A | 3.77 | 20.8 | 5.51 | 2.2 x 10^-9^ | blood vessel development | nucleus |
| 223368_s_at | METTL11A | methyltransferase like 11A | 2.79 | 15.4 | 5.51 | 1.1 x 10^-5^ |  |  |
| 223520_s_at | KIF13A | kinesin family member 13A | 0.34 | 1.9 | 5.51 | 2.7 x 10^-5^ | transport | microtubule |
| 240135_x_at | --- | --- | 0.16 | 0.9 | 5.51 | 5.2 x 10^-3^ |  |  |
| 219938_s_at | PSTPIP2 | proline-serine-threonine phosphatase interacting protein 2 | 1.26 | 6.9 | 5.51 | 8.4 x 10^-7^ |  | cytoplasm |
| 1555733_s_at | AP1S3 | adaptor-related protein complex 1, sigma 3 subunit | 2.34 | 12.9 | 5.50 | 4.3 x 10^-6^ | transport | Golgi apparatus |
| 204616_at | UCHL3 | ubiquitin carboxyl-terminal esterase L3 (ubiquitin thiolesterase) | 15.13 | 83.2 | 5.50 | 2.0 x 10^-10^ | ubiquitin-dependent protein catabolic process | intracellular |
| 202735_at | EBP | emopamil binding protein (sterol isomerase) | 5.06 | 27.8 | 5.50 | 4.6 x 10^-5^ | skeletal system development | endoplasmic reticulum |
| 200952_s_at | CCND2 | cyclin D2 | 0.58 | 3.2 | 5.50 | 5.9 x 10^-5^ | G1 | cyclin-dependent protein kinase holoenzyme complex |
| 204331_s_at | MRPS12 | mitochondrial ribosomal protein S12 | 5.51 | 30.3 | 5.50 | 5.1 x 10^-6^ | translation | intracellular |
| 213843_x_at | SLC6A8 | solute carrier family 6 (neurotransmitter transporter, creatine), member 8 | 3.48 | 19.2 | 5.50 | 2.8 x 10^-5^ | transport | integral to plasma membrane |
| 1553021_s_at | BICD2 | bicaudal D homolog 2 (Drosophila) | 0.65 | 3.6 | 5.50 | 3.5 x 10^-5^ | transport | cytoplasm |
| 201013_s_at | PAICS | phosphoribosylaminoimidazole carboxylase, phosphoribosylaminoimidazole succinocarboxamide synthetase | 12.52 | 68.8 | 5.50 | 1.8 x 10^-5^ | purine nucleotide biosynthetic process | phosphoribosylaminoimidazole carboxylase complex |
| 208727_s_at | CDC42 | cell division cycle 42 (GTP binding protein, 25kDa) | 19.31 | 106.1 | 5.50 | 1.7 x 10^-3^ | nuclear migration | intracellular |
| 216564_at | --- | --- | 0.29 | 1.6 | 5.50 | 4.7 x 10^-4^ |  |  |
| 201963_at | ACSL1 | acyl-CoA synthetase long-chain family member 1 | 9.19 | 50.5 | 5.49 | 3.1 x 10^-5^ | lipid metabolic process | mitochondrion |
| 215199_at | CALD1 | caldesmon 1 | 0.24 | 1.3 | 5.49 | 3.9 x 10^-3^ | cell motion | membrane fraction |
| 203574_at | NFIL3 | nuclear factor, interleukin 3 regulated | 8.77 | 48.2 | 5.49 | 3.0 x 10^-7^ | transcription | nucleus |
| 226858_at | CSNK1E | casein kinase 1, epsilon | 3.58 | 19.7 | 5.49 | 3.1 x 10^-6^ | DNA repair | nucleus |
| 210916_s_at | CD44 | CD44 molecule (Indian blood group) | 3.78 | 20.7 | 5.49 | 5.8 x 10^-5^ | regulation of cell growth | cytoplasm |
| 236913_at | --- | --- | 0.76 | 4.2 | 5.49 | 3.3 x 10^-3^ |  |  |
| 202842_s_at | DNAJB9 | DnaJ (Hsp40) homolog, subfamily B, member 9 | 16.26 | 89.2 | 5.49 | 1.8 x 10^-4^ | protein folding | nucleus |
| 224559_at | MALAT1 | metastasis associated lung adenocarcinoma transcript 1 (non-protein coding) | 19.52 | 107.1 | 5.49 | 7.8 x 10^-3^ |  |  |
| 208925_at | CLDND1 | claudin domain containing 1 | 9.90 | 54.3 | 5.48 | 3.4 x 10^-8^ |  | membrane |
| 209052_s_at | WHSC1 | Wolf-Hirschhorn syndrome candidate 1 | 0.84 | 4.6 | 5.48 | 5.3 x 10^-5^ | transcription | nucleus |
| 225300_at | C15orf23 | chromosome 15 open reading frame 23 | 2.73 | 15.0 | 5.48 | 2.0 x 10^-3^ |  | nucleus |
| 209524_at | HDGFRP3 | hepatoma-derived growth factor, related protein 3 | 6.78 | 37.1 | 5.48 | 3.1 x 10^-7^ | cell proliferation | nucleus |
| 216548_x_at | HMGB3L1 | high-mobility group box 3-like 1 | 2.06 | 11.3 | 5.48 | 5.0 x 10^-5^ |  | nucleus |
| 37966_at | PARVB | parvin, beta | 0.25 | 1.4 | 5.48 | 5.0 x 10^-6^ | cell adhesion | cytoplasm |
| 201019_s_at | EIF1AP1 /// EIF1AX | eukaryotic translation initiation factor 1A pseudogene 1 /// eukaryotic translation initiation factor 1A, X-linked | 21.86 | 119.7 | 5.48 | 2.0 x 10^-5^ | translation | cytoplasm |
| 218669_at | RAP2C | RAP2C, member of RAS oncogene family | 6.69 | 36.6 | 5.47 | 5.8 x 10^-8^ | small GTPase mediated signal transduction | intracellular |
| 222548_s_at | MAP4K4 | mitogen-activated protein kinase kinase kinase kinase 4 | 1.58 | 8.6 | 5.47 | 4.1 x 10^-8^ | protein amino acid phosphorylation |  |
| 217294_s_at | ENO1 | enolase 1, (alpha) | 22.42 | 122.5 | 5.46 | 5.1 x 10^-4^ | negative regulation of transcription from RNA polymerase II promoter | phosphopyruvate hydratase complex |
| 235022_at | C18orf19 | chromosome 18 open reading frame 19 | 1.76 | 9.6 | 5.46 | 1.0 x 10^-10^ |  | membrane |
| 208679_s_at | ARPC2 | actin related protein 2/3 complex, subunit 2, 34kDa | 72.53 | 396.2 | 5.46 | 1.2 x 10^-7^ | cell motion | cytoplasm |
| 1568623_a_at | SLC35E4 | solute carrier family 35, member E4 | 0.28 | 1.5 | 5.46 | 7.0 x 10^-6^ |  | membrane |
| 217963_s_at | NGFRAP1 | nerve growth factor receptor (TNFRSF16) associated protein 1 | 21.74 | 118.7 | 5.46 | 7.3 x 10^-9^ | apoptosis | nucleus |
| 200889_s_at | SSR1 | signal sequence receptor, alpha | 10.19 | 55.6 | 5.46 | 1.0 x 10^-6^ | cotranslational protein targeting to membrane | endoplasmic reticulum |
| 214214_s_at | C1QBP | complement component 1, q subcomponent binding protein | 20.92 | 114.2 | 5.46 | 1.1 x 10^-6^ | immune response | mitochondrion |
| 208961_s_at | KLF6 | Kruppel-like factor 6 | 11.36 | 62.0 | 5.46 | 7.5 x 10^-5^ | transcription | intracellular |
| 223315_at | NTN4 | netrin 4 | 6.87 | 37.5 | 5.46 | 1.2 x 10^-5^ | neuron remodeling | extracellular region |
| 244242_at | --- | --- | 0.79 | 4.3 | 5.45 | 1.9 x 10^-7^ |  |  |
| 230143_at | RNF165 | ring finger protein 165 | 0.60 | 3.3 | 5.45 | 1.1 x 10^-3^ |  |  |
| 206307_s_at | FOXD1 | forkhead box D1 | 1.23 | 6.7 | 5.45 | 3.8 x 10^-4^ | transcription | nucleus |
| 210986_s_at | TPM1 | tropomyosin 1 (alpha) | 16.55 | 90.2 | 5.45 | 8.9 x 10^-6^ | in utero embryonic development | stress fiber |
| 201122_x_at | EIF5A | eukaryotic translation initiation factor 5A | 6.27 | 34.1 | 5.44 | 5.5 x 10^-4^ | mRNA export from nucleus | nucleus |
| 230972_at | ANKRD9 | ankyrin repeat domain 9 | 4.89 | 26.6 | 5.44 | 9.8 x 10^-6^ |  |  |
| 201848_s_at | BNIP3 | BCL2/adenovirus E1B 19kDa interacting protein 3 | 7.01 | 38.1 | 5.44 | 4.3 x 10^-10^ | response to hypoxia | nucleus |
| 218777_at | REEP4 | receptor accessory protein 4 | 2.85 | 15.5 | 5.44 | 6.9 x 10^-9^ |  | membrane |
| 239106_at | CA5BP | Carbonic anhydrase VB pseudogene | 2.37 | 12.9 | 5.43 | 3.2 x 10^-3^ | one-carbon compound metabolic process | mitochondrion |
| 209896_s_at | PTPN11 | protein tyrosine phosphatase, non-receptor type 11 | 8.00 | 43.4 | 5.43 | 5.0 x 10^-8^ | protein amino acid dephosphorylation | cytoplasm |
| 1555906_s_at | C3orf23 | chromosome 3 open reading frame 23 | 4.11 | 22.3 | 5.43 | 2.4 x 10^-5^ |  | mitochondrion |
| 217437_s_at | TACC1 | transforming, acidic coiled-coil containing protein 1 | 1.75 | 9.5 | 5.43 | 1.5 x 10^-5^ | microtubule cytoskeleton organization | nucleus |
| 240788_at | --- | --- | 0.67 | 3.6 | 5.43 | 5.7 x 10^-4^ |  |  |
| 224763_at | RPL37 | Ribosomal protein L37 | 4.33 | 23.5 | 5.42 | 6.2 x 10^-6^ | translation | intracellular |
| 234608_at | LAMA3 | laminin, alpha 3 | 0.62 | 3.4 | 5.42 | 1.8 x 10^-3^ | cell adhesion | extracellular region |
| 220610_s_at | LRRFIP2 | leucine rich repeat (in FLII) interacting protein 2 | 4.05 | 21.9 | 5.42 | 2.9 x 10^-7^ | Wnt receptor signaling pathway |  |
| 205195_at | AP1S1 | adaptor-related protein complex 1, sigma 1 subunit | 0.72 | 3.9 | 5.41 | 1.1 x 10^-4^ | transport | Golgi apparatus |
| 202131_s_at | RIOK3 | RIO kinase 3 (yeast) | 8.42 | 45.5 | 5.41 | 5.5 x 10^-4^ | protein amino acid phosphorylation | nucleus |
| 204854_at | LEPREL2 | leprecan-like 2 | 0.25 | 1.4 | 5.41 | 3.5 x 10^-5^ | protein metabolic process | endoplasmic reticulum |
| 235563_at | --- | --- | 0.30 | 1.6 | 5.41 | 6.4 x 10^-5^ |  |  |
| 1552648_a_at | TNFRSF10A | tumor necrosis factor receptor superfamily, member 10a | 1.10 | 5.9 | 5.41 | 2.8 x 10^-7^ | apoptosis | membrane |
| 241418_at | LOC344887 | similar to hCG2041270 | 0.57 | 3.1 | 5.40 | 2.2 x 10^-3^ |  |  |
| 220255_at | FANCE | Fanconi anemia, complementation group E | 1.96 | 10.6 | 5.40 | 1.1 x 10^-9^ | DNA repair | nucleus |
| 1564307_a_at | A2ML1 | alpha-2-macroglobulin-like 1 | 0.47 | 2.5 | 5.40 | 4.3 x 10^-4^ | regulation of endopeptidase activity | extracellular region |
| 226891_at | C3orf21 | chromosome 3 open reading frame 21 | 0.34 | 1.8 | 5.39 | 9.9 x 10^-6^ |  | membrane |
| 243754_at | --- | --- | 1.03 | 5.6 | 5.39 | 6.6 x 10^-3^ |  |  |
| 37408_at | MRC2 | mannose receptor, C type 2 | 2.09 | 11.2 | 5.39 | 1.2 x 10^-4^ | endocytosis | membrane |
| 217738_at | NAMPT | nicotinamide phosphoribosyltransferase | 10.82 | 58.3 | 5.39 | 1.7 x 10^-5^ | signal transduction | cytoplasm |
| 1554086_at | TUBGCP3 | tubulin, gamma complex associated protein 3 | 0.37 | 2.0 | 5.38 | 1.5 x 10^-6^ | microtubule cytoskeleton organization | spindle pole |
| 213061_s_at | NTAN1 | N-terminal asparagine amidase | 3.52 | 19.0 | 5.38 | 2.0 x 10^-9^ | memory | nucleus |
| 203023_at | NOP16 | NOP16 nucleolar protein homolog (yeast) | 2.42 | 13.0 | 5.38 | 2.6 x 10^-7^ |  | nucleus |
| 223077_at | TMOD3 | tropomodulin 3 (ubiquitous) | 10.92 | 58.7 | 5.38 | 1.3 x 10^-6^ |  | cytoplasm |
| 242997_at | --- | --- | 0.39 | 2.1 | 5.38 | 3.9 x 10^-5^ |  |  |
| 224207_x_at | MMP28 | matrix metallopeptidase 28 | 0.82 | 4.4 | 5.38 | 2.2 x 10^-4^ | proteolysis | extracellular region |
| 221830_at | RAP2A | RAP2A, member of RAS oncogene family | 6.88 | 37.0 | 5.37 | 6.3 x 10^-5^ | signal transduction | intracellular |
| 213812_s_at | CAMKK2 | calcium/calmodulin-dependent protein kinase kinase 2, beta | 5.97 | 32.1 | 5.37 | 5.4 x 10^-8^ | MAPKKK cascade | intracellular |
| 208161_s_at | ABCC3 | ATP-binding cassette, sub-family C (CFTR/MRP), member 3 | 3.28 | 17.6 | 5.37 | 5.0 x 10^-4^ | transport | membrane fraction |
| 216449_x_at | HSP90B1 | heat shock protein 90kDa beta (Grp94), member 1 | 27.61 | 148.2 | 5.37 | 8.4 x 10^-6^ | response to hypoxia | endoplasmic reticulum |
| 213901_x_at | RBM9 | RNA binding motif protein 9 | 4.72 | 25.3 | 5.37 | 4.6 x 10^-10^ | mRNA processing | nucleus |
| 230127_at | --- | --- | 0.78 | 4.2 | 5.36 | 2.4 x 10^-6^ |  |  |
| 231808_at | LOC729082 | hypothetical protein LOC729082 | 3.80 | 20.4 | 5.36 | 8.5 x 10^-4^ |  |  |
| 210854_x_at | SLC6A8 | solute carrier family 6 (neurotransmitter transporter, creatine), member 8 | 3.55 | 19.0 | 5.35 | 2.9 x 10^-6^ | transport | integral to plasma membrane |
| 37462_i_at | SF3A2 | splicing factor 3a, subunit 2, 66kDa | 1.42 | 7.6 | 5.35 | 3.2 x 10^-4^ | nuclear mRNA 3'-splice site recognition | intracellular |
| 221753_at | SSH1 | slingshot homolog 1 (Drosophila) | 3.97 | 21.3 | 5.35 | 6.5 x 10^-9^ | cell morphogenesis | cytoplasm |
| 220685_at | FAM120C | family with sequence similarity 120C | 0.31 | 1.7 | 5.35 | 6.1 x 10^-5^ |  |  |
| 216375_s_at | ETV5 | ets variant 5 | 0.59 | 3.2 | 5.35 | 1.1 x 10^-5^ | regulation of transcription, DNA-dependent | nucleus |
| 222752_s_at | TMEM206 | transmembrane protein 206 | 1.98 | 10.6 | 5.35 | 2.8 x 10^-8^ |  | membrane |
| 214126_at | --- | --- | 1.91 | 10.2 | 5.35 | 7.8 x 10^-5^ |  |  |
| 209371_s_at | SH3BP2 | SH3-domain binding protein 2 | 0.71 | 3.8 | 5.35 | 6.0 x 10^-6^ | signal transduction |  |
| 213757_at | --- | --- | 9.84 | 52.6 | 5.35 | 4.9 x 10^-4^ |  |  |
| 225750_at | --- | --- | 13.48 | 72.1 | 5.34 | 4.5 x 10^-9^ |  |  |
| 222872_x_at | OBFC2A | oligonucleotide/oligosaccharide-binding fold containing 2A | 3.74 | 20.0 | 5.34 | 1.4 x 10^-6^ |  | nucleus |
| 200885_at | RHOC | ras homolog gene family, member C | 18.77 | 100.3 | 5.34 | 5.5 x 10^-7^ | small GTPase mediated signal transduction | intracellular |
| 212985_at | APBB2 | amyloid beta (A4) precursor protein-binding, family B, member 2 | 2.17 | 11.6 | 5.34 | 3.8 x 10^-6^ | neuron migration | nucleus |
| 208260_at | AVPR1B | arginine vasopressin receptor 1B | 0.36 | 1.9 | 5.34 | 2.0 x 10^-6^ | regulation of systemic arterial blood pressure by vasopressin | endosome |
| 217988_at | CCNB1IP1 | cyclin B1 interacting protein 1 | 9.52 | 50.8 | 5.33 | 1.3 x 10^-10^ | modification-dependent protein catabolic process | nucleus |
| 203105_s_at | DNM1L | dynamin 1-like | 6.15 | 32.8 | 5.33 | 6.0 x 10^-10^ | mitochondrial membrane organization | cytoplasm |
| 229815_at | --- | --- | 0.47 | 2.5 | 5.33 | 5.6 x 10^-5^ |  |  |
| 1569149_at | PDLIM7 | PDZ and LIM domain 7 (enigma) | 0.26 | 1.4 | 5.33 | 3.2 x 10^-4^ | ossification | cytoplasm |
| 234512_x_at | RPL7AP36 | ribosomal protein L7a pseudogene 36 | 29.91 | 159.4 | 5.33 | 1.1 x 10^-3^ |  |  |
| 1557094_at | LOC653110 | hypothetical LOC653110 | 1.58 | 8.4 | 5.32 | 3.0 x 10^-5^ |  |  |
| 204244_s_at | DBF4 | DBF4 homolog (S. cerevisiae) | 3.02 | 16.1 | 5.32 | 4.6 x 10^-3^ | G1 | intracellular |
| 223960_s_at | C16orf5 | chromosome 16 open reading frame 5 | 1.12 | 5.9 | 5.32 | 1.7 x 10^-6^ | apoptosis | nucleus |
| 213532_at | ADAM17 | ADAM metallopeptidase domain 17 | 5.77 | 30.7 | 5.32 | 5.3 x 10^-9^ | response to hypoxia | cytoplasm |
| 218775_s_at | WWC2 | WW and C2 domain containing 2 | 1.62 | 8.6 | 5.32 | 2.9 x 10^-5^ |  | plasma membrane |
| 202224_at | CRK | v-crk sarcoma virus CT10 oncogene homolog (avian) | 6.50 | 34.6 | 5.32 | 1.6 x 10^-9^ | regulation of transcription from RNA polymerase II promoter | nucleus |
| 218046_s_at | MRPS16 | mitochondrial ribosomal protein S16 | 7.41 | 39.4 | 5.32 | 1.2 x 10^-6^ | translation | intracellular |
| 225943_at | NLN | neurolysin (metallopeptidase M3 family) | 1.43 | 7.6 | 5.32 | 5.4 x 10^-8^ | proteolysis | cytoplasm |
| 216693_x_at | HDGFRP3 | hepatoma-derived growth factor, related protein 3 | 5.74 | 30.5 | 5.31 | 5.7 x 10^-8^ | cell proliferation | nucleus |
| 205174_s_at | QPCT | glutaminyl-peptide cyclotransferase | 0.91 | 4.8 | 5.31 | 4.6 x 10^-4^ | protein modification process |  |
| 212018_s_at | RSL1D1 | ribosomal L1 domain containing 1 | 13.15 | 69.8 | 5.31 | 3.4 x 10^-7^ | RNA processing | intracellular |
| 203332_s_at | INPP5D | inositol polyphosphate-5-phosphatase, 145kDa | 1.49 | 7.9 | 5.30 | 2.3 x 10^-5^ | phosphate metabolic process | cytoplasm |
| 1552476_s_at | PLCD3 | phospholipase C, delta 3 | 0.72 | 3.8 | 5.30 | 7.9 x 10^-6^ | angiogenesis | cytoplasm |
| 204528_s_at | NAP1L1 | nucleosome assembly protein 1-like 1 | 21.55 | 114.3 | 5.30 | 1.3 x 10^-5^ | DNA replication | nucleus |
| 210740_s_at | ITPK1 | inositol 1,3,4-triphosphate 5/6 kinase | 5.20 | 27.6 | 5.30 | 3.6 x 10^-8^ | signal transduction | intracellular |
| 209457_at | DUSP5 | dual specificity phosphatase 5 | 14.33 | 76.0 | 5.30 | 3.5 x 10^-4^ | protein amino acid dephosphorylation | nucleus |
| 31846_at | RHOD | ras homolog gene family, member D | 8.44 | 44.7 | 5.30 | 2.2 x 10^-7^ | small GTPase mediated signal transduction | intracellular |
| 1553333_at | C1orf161 | chromosome 1 open reading frame 161 | 0.16 | 0.8 | 5.30 | 9.3 x 10^-3^ |  |  |
| 229212_at | --- | --- | 0.51 | 2.7 | 5.30 | 8.7 x 10^-5^ |  |  |
| 206467_x_at | RTEL1 /// TNFRSF6B | regulator of telomere elongation helicase 1 /// tumor necrosis factor receptor superfamily, member 6b, decoy | 1.11 | 5.9 | 5.29 | 8.2 x 10^-3^ | telomere maintenance | extracellular region |
| 217790_s_at | SSR3 | signal sequence receptor, gamma (translocon-associated protein gamma) | 2.88 | 15.2 | 5.29 | 1.7 x 10^-5^ | cotranslational protein targeting to membrane | endoplasmic reticulum |
| 238452_at | FCRLB | Fc receptor-like B | 0.15 | 0.8 | 5.29 | 1.7 x 10^-3^ |  | cytoplasm |
| 202442_at | AP3S1 | adaptor-related protein complex 3, sigma 1 subunit | 21.83 | 115.5 | 5.29 | 3.1 x 10^-9^ | transport | Golgi apparatus |
| 228050_at | UTP15 | UTP15, U3 small nucleolar ribonucleoprotein, homolog (S. cerevisiae) | 1.52 | 8.0 | 5.29 | 1.4 x 10^-5^ | rRNA processing | nucleus |
| 217150_s_at | NF2 | neurofibromin 2 (merlin) | 0.80 | 4.2 | 5.29 | 7.4 x 10^-5^ | mesoderm formation | ruffle |
| 200690_at | HSPA9 | heat shock 70kDa protein 9 (mortalin) | 4.09 | 21.6 | 5.29 | 7.0 x 10^-5^ | protein folding | cytoplasm |
| 204119_s_at | ADK | adenosine kinase | 8.54 | 45.2 | 5.29 | 8.2 x 10^-7^ | purine ribonucleoside salvage | nucleus |
| 217173_s_at | LDLR | low density lipoprotein receptor | 2.37 | 12.5 | 5.28 | 1.3 x 10^-4^ | protein amino acid O-linked glycosylation | endosome |
| 243287_s_at | OSTM1 | osteopetrosis associated transmembrane protein 1 | 0.59 | 3.1 | 5.28 | 3.9 x 10^-6^ | osteoclast differentiation | cytosol |
| 212821_at | PLEKHG3 | pleckstrin homology domain containing, family G (with RhoGef domain) member 3 | 0.48 | 2.5 | 5.28 | 7.6 x 10^-5^ | regulation of Rho protein signal transduction | intracellular |
| 217564_s_at | CPS1 | carbamoyl-phosphate synthetase 1, mitochondrial | 0.32 | 1.7 | 5.27 | 1.7 x 10^-4^ | urea cycle | intracellular |
| 220755_s_at | C6orf48 | chromosome 6 open reading frame 48 | 22.57 | 118.7 | 5.26 | 2.3 x 10^-5^ |  |  |
| 210298_x_at | FHL1 | four and a half LIM domains 1 | 1.24 | 6.5 | 5.26 | 6.2 x 10^-4^ | multicellular organismal development | nucleus |
| 232331_at | --- | --- | 0.59 | 3.1 | 5.26 | 2.4 x 10^-5^ |  |  |
| 242329_at | LOC401317 | hypothetical LOC401317 | 0.44 | 2.3 | 5.25 | 7.1 x 10^-5^ |  |  |
| 207172_s_at | CDH11 | cadherin 11, type 2, OB-cadherin (osteoblast) | 1.01 | 5.3 | 5.25 | 1.7 x 10^-5^ | skeletal system development | cytoplasm |
| 228005_at | ZXDB | zinc finger, X-linked, duplicated B | 2.97 | 15.6 | 5.25 | 1.4 x 10^-3^ | transcription | intracellular |
| 205264_at | CD3EAP | CD3e molecule, epsilon associated protein | 1.42 | 7.5 | 5.25 | 1.4 x 10^-6^ | transcription | RNA polymerase I transcription factor complex |
| 227757_at | CUL4A | cullin 4A | 0.56 | 2.9 | 5.25 | 6.1 x 10^-4^ | G1 | cullin-RING ubiquitin ligase complex |
| 219933_at | GLRX2 | glutaredoxin 2 | 4.76 | 25.0 | 5.25 | 7.4 x 10^-10^ | protein thiol-disulfide exchange | nucleus |
| 228059_x_at | MRPS22 | mitochondrial ribosomal protein S22 | 5.90 | 31.0 | 5.25 | 1.7 x 10^-7^ |  | mitochondrion |
| 208084_at | ITGB6 | integrin, beta 6 | 0.90 | 4.7 | 5.24 | 5.7 x 10^-3^ | inflammatory response | integrin complex |
| 227068_at | PGK1 | phosphoglycerate kinase 1 | 6.24 | 32.7 | 5.24 | 8.8 x 10^-4^ | glycolysis | cytoplasm |
| 230778_at | --- | --- | 2.60 | 13.7 | 5.24 | 4.4 x 10^-8^ |  |  |
| 215918_s_at | SPTBN1 | spectrin, beta, non-erythrocytic 1 | 0.56 | 3.0 | 5.24 | 4.6 x 10^-8^ | common-partner SMAD protein phosphorylation | nucleus |
| 1555895_at | DNM2 | dynamin 2 | 0.47 | 2.5 | 5.24 | 7.0 x 10^-5^ | G2 | cytoplasm |
| 203597_s_at | WBP4 | WW domain binding protein 4 (formin binding protein 21) | 2.26 | 11.8 | 5.24 | 3.4 x 10^-8^ | mRNA processing | nucleus |
| 225066_at | --- | --- | 1.07 | 5.6 | 5.24 | 4.3 x 10^-3^ | signal transduction | protein phosphatase type 2A complex |
| 202712_s_at | CKMT1A /// CKMT1B | creatine kinase, mitochondrial 1A /// creatine kinase, mitochondrial 1B | 9.45 | 49.5 | 5.24 | 5.6 x 10^-5^ | creatine metabolic process | mitochondrion |
| 215082_at | ELOVL5 | ELOVL family member 5, elongation of long chain fatty acids (FEN1/Elo2, SUR4/Elo3-like, yeast) | 0.62 | 3.2 | 5.23 | 4.8 x 10^-6^ | fatty acid biosynthetic process | endoplasmic reticulum |
| 204462_s_at | SLC16A2 | solute carrier family 16, member 2 (monocarboxylic acid transporter 8) | 1.22 | 6.4 | 5.23 | 1.5 x 10^-5^ | transport | membrane fraction |
| 1554667_s_at | METTL8 | methyltransferase like 8 | 3.23 | 16.9 | 5.22 | 1.5 x 10^-7^ | skeletal muscle tissue development | nucleus |
| 224937_at | PTGFRN | prostaglandin F2 receptor negative regulator | 10.12 | 52.8 | 5.22 | 1.1 x 10^-4^ |  | endoplasmic reticulum |
| 202613_at | CTPS | CTP synthase | 1.98 | 10.3 | 5.22 | 2.0 x 10^-4^ | nucleobase, nucleoside, nucleotide and nucleic acid metabolic process | cytosol |
| 218602_s_at | HAUS6 | HAUS augmin-like complex, subunit 6 | 0.93 | 4.9 | 5.22 | 9.0 x 10^-5^ | cell cycle | cytoplasm |
| 226712_at | SSR1 | signal sequence receptor, alpha | 2.80 | 14.6 | 5.22 | 1.4 x 10^-4^ | cotranslational protein targeting to membrane | endoplasmic reticulum |
| 207717_s_at | PKP2 | plakophilin 2 | 4.84 | 25.3 | 5.22 | 3.7 x 10^-7^ | cell adhesion | nucleus |
| 231932_at | TRAF3IP3 | TRAF3 interacting protein 3 | 0.90 | 4.7 | 5.22 | 3.1 x 10^-3^ |  | membrane |
| 209187_at | DR1 | down-regulator of transcription 1, TBP-binding (negative cofactor 2) | 9.78 | 51.0 | 5.21 | 6.8 x 10^-9^ | negative regulation of transcription from RNA polymerase II promoter | intracellular |
| 218732_at | PTRH2 | peptidyl-tRNA hydrolase 2 | 5.55 | 28.9 | 5.21 | 5.8 x 10^-6^ | translation | chromosome, centromeric region |
| 218537_at | HCFC1R1 | host cell factor C1 regulator 1 (XPO1 dependent) | 0.59 | 3.1 | 5.21 | 1.8 x 10^-3^ |  | nucleus |
| 216867_s_at | PDGFA | platelet-derived growth factor alpha polypeptide | 0.65 | 3.4 | 5.21 | 3.5 x 10^-5^ | cell activation | extracellular region |
| 231579_s_at | TIMP2 | TIMP metallopeptidase inhibitor 2 | 3.16 | 16.5 | 5.21 | 2.8 x 10^-5^ | negative regulation of cell proliferation | extracellular region |
| 221654_s_at | USP3 | ubiquitin specific peptidase 3 | 14.17 | 73.8 | 5.21 | 5.4 x 10^-7^ | ubiquitin-dependent protein catabolic process |  |
| 218823_s_at | KCTD9 | potassium channel tetramerisation domain containing 9 | 4.83 | 25.1 | 5.21 | 2.8 x 10^-8^ | potassium ion transport | voltage-gated potassium channel complex |
| 200730_s_at | PTP4A1 | protein tyrosine phosphatase type IVA, member 1 | 11.14 | 58.0 | 5.20 | 2.3 x 10^-6^ | protein amino acid dephosphorylation | nucleus |
| 226802_s_at | LOC96610 | BMS1 homolog, ribosome assembly protein (yeast) pseudogene | 1.84 | 9.6 | 5.20 | 7.0 x 10^-6^ |  |  |
| 226280_at | BNIP2 | BCL2/adenovirus E1B 19kDa interacting protein 2 | 4.11 | 21.4 | 5.20 | 4.0 x 10^-7^ | apoptosis | nuclear envelope |
| 200615_s_at | AP2B1 | adaptor-related protein complex 2, beta 1 subunit | 7.24 | 37.7 | 5.20 | 9.5 x 10^-8^ | intracellular protein transport | cytosol |
| 212250_at | MTDH | metadherin | 15.48 | 80.5 | 5.20 | 4.6 x 10^-6^ | negative regulation of transcription from RNA polymerase II promoter | nucleus |
| 237415_at | --- | --- | 0.59 | 3.1 | 5.20 | 3.3 x 10^-5^ |  |  |
| 237145_at | EIF2AK4 | eukaryotic translation initiation factor 2 alpha kinase 4 | 0.25 | 1.3 | 5.20 | 1.4 x 10^-3^ | translation | intracellular |
| 239614_x_at | --- | --- | 0.48 | 2.5 | 5.20 | 3.6 x 10^-3^ |  |  |
| 241431_at | --- | --- | 0.32 | 1.6 | 5.20 | 1.9 x 10^-5^ |  |  |
| 209786_at | HMGN4 | high mobility group nucleosomal binding domain 4 | 9.04 | 47.0 | 5.20 | 2.4 x 10^-8^ |  | chromatin |
| 200700_s_at | KDELR2 | KDEL (Lys-Asp-Glu-Leu)endoplasmic reticulum protein retention receptor 2 | 25.43 | 132.0 | 5.19 | 7.5 x 10^-6^ | protein retention in ER lumen | endoplasmic reticulum |
| 202720_at | TES | testis derived transcript (3 LIM domains) | 22.33 | 115.9 | 5.19 | 2.9 x 10^-9^ |  |  |
| 222265_at | TNS4 | tensin 4 | 0.81 | 4.2 | 5.19 | 6.9 x 10^-6^ | apoptosis | cytoplasm |
| 227117_at | --- | --- | 6.12 | 31.8 | 5.19 | 9.7 x 10^-8^ |  |  |
| 226223_at | --- | --- | 8.78 | 45.6 | 5.19 | 1.6 x 10^-6^ |  |  |
| 233044_at | --- | --- | 1.28 | 6.7 | 5.19 | 3.0 x 10^-3^ |  |  |
| 200625_s_at | CAP1 | CAP, adenylate cyclase-associated protein 1 (yeast) | 36.38 | 188.7 | 5.19 | 1.5 x 10^-6^ | cell morphogenesis | cytoplasm |
| 225585_at | RAP2A | RAP2A, member of RAS oncogene family | 3.47 | 18.0 | 5.19 | 2.9 x 10^-7^ | signal transduction | intracellular |
| 34187_at | RBMS2 | RNA binding motif, single stranded interacting protein 2 | 2.17 | 11.3 | 5.18 | 3.3 x 10^-5^ | RNA processing | nucleus |
| 204958_at | PLK3 | polo-like kinase 3 (Drosophila) | 0.91 | 4.7 | 5.18 | 1.5 x 10^-7^ | protein amino acid phosphorylation | membrane |
| 225830_at | PDZD8 | PDZ domain containing 8 | 1.81 | 9.4 | 5.18 | 2.1 x 10^-5^ | intracellular signaling cascade |  |
| 223075_s_at | AIF1L | allograft inflammatory factor 1-like | 0.63 | 3.3 | 5.18 | 5.3 x 10^-6^ |  | focal adhesion |
| 216396_s_at | EI24 | etoposide induced 2.4 mRNA | 7.22 | 37.4 | 5.17 | 5.2 x 10^-6^ | induction of apoptosis | membrane |
| 203910_at | ARHGAP29 | Rho GTPase activating protein 29 | 7.23 | 37.4 | 5.17 | 1.6 x 10^-5^ | signal transduction | intracellular |
| 202231_at | EIF3M | eukaryotic translation initiation factor 3, subunit M | 32.55 | 168.3 | 5.17 | 1.0 x 10^-6^ | translation | cytoplasm |
| 1558046_x_at | LOC441528 | hypothetical protein LOC441528 | 0.40 | 2.1 | 5.17 | 4.2 x 10^-4^ |  |  |
| 212612_at | RCOR1 | REST corepressor 1 | 7.38 | 38.2 | 5.17 | 5.6 x 10^-6^ | transcription | nucleus |
| 225831_at | LUZP1 | leucine zipper protein 1 | 3.21 | 16.6 | 5.17 | 2.0 x 10^-5^ |  | nucleus |
| 214168_s_at | TJP1 | tight junction protein 1 (zona occludens 1) | 4.36 | 22.5 | 5.16 | 1.9 x 10^-6^ | blastocyst formation | nucleus |
| 208511_at | PTTG3 | pituitary tumor-transforming 3 | 0.53 | 2.7 | 5.16 | 7.9 x 10^-3^ | DNA metabolic process | nucleus |
| 200606_at | DSP | desmoplakin | 69.56 | 359.2 | 5.16 | 9.1 x 10^-7^ | epidermis development | cornified envelope |
| 205349_at | GNA15 | guanine nucleotide binding protein (G protein), alpha 15 (Gq class) | 5.59 | 28.9 | 5.16 | 5.0 x 10^-7^ | protein amino acid ADP-ribosylation | heterotrimeric G-protein complex |
| 200853_at | H2AFZ | H2A histone family, member Z | 38.27 | 197.3 | 5.16 | 1.5 x 10^-6^ | nucleosome assembly | nucleosome |
| 241014_at | LOC339400 | hypothetical protein LOC339400 | 0.30 | 1.5 | 5.16 | 2.8 x 10^-3^ |  |  |
| 212610_at | PTPN11 | protein tyrosine phosphatase, non-receptor type 11 | 24.27 | 125.1 | 5.16 | 1.1 x 10^-7^ | protein amino acid dephosphorylation | cytoplasm |
| 1553574_at | IFNE | interferon, epsilon | 0.46 | 2.4 | 5.15 | 2.4 x 10^-3^ | defense response | extracellular region |
| 219293_s_at | OLA1 | Obg-like ATPase 1 | 27.43 | 141.3 | 5.15 | 8.2 x 10^-8^ | ATP catabolic process | intracellular |
| 201189_s_at | ITPR3 | inositol 1,4,5-triphosphate receptor, type 3 | 12.41 | 63.9 | 5.15 | 1.7 x 10^-8^ | transport | nucleus |
| 213523_at | CCNE1 | cyclin E1 | 0.71 | 3.7 | 5.15 | 1.9 x 10^-8^ | G1 | nucleus |
| 222759_at | SUV420H1 | suppressor of variegation 4-20 homolog 1 (Drosophila) | 3.86 | 19.9 | 5.15 | 1.4 x 10^-4^ | transcription | nucleus |
| 209100_at | IFRD2 | interferon-related developmental regulator 2 | 4.94 | 25.4 | 5.15 | 3.1 x 10^-5^ | cell proliferation |  |
| 201064_s_at | PABPC4 | poly(A) binding protein, cytoplasmic 4 (inducible form) | 21.95 | 112.9 | 5.14 | 4.4 x 10^-6^ | RNA processing | cytoplasm |
| 204053_x_at | PTEN | phosphatase and tensin homolog | 10.07 | 51.8 | 5.14 | 2.0 x 10^-4^ | regulation of cyclin-dependent protein kinase activity | nucleus |
| 1553768_a_at | DCBLD1 | discoidin, CUB and LCCL domain containing 1 | 1.08 | 5.6 | 5.14 | 2.9 x 10^-4^ | cell adhesion | membrane |
| 202787_s_at | MAPKAPK3 | mitogen-activated protein kinase-activated protein kinase 3 | 1.87 | 9.6 | 5.14 | 6.2 x 10^-5^ | protein amino acid phosphorylation | nucleus |
| 201980_s_at | RSU1 | Ras suppressor protein 1 | 9.84 | 50.6 | 5.14 | 9.9 x 10^-8^ | signal transduction |  |
| 205579_at | HRH1 | histamine receptor H1 | 0.45 | 2.3 | 5.14 | 6.4 x 10^-5^ | inflammatory response | plasma membrane |
| 207017_at | RAB27B | RAB27B, member RAS oncogene family | 0.70 | 3.6 | 5.14 | 6.0 x 10^-3^ | small GTPase mediated signal transduction | Golgi apparatus |
| 239794_at | --- | --- | 1.66 | 8.5 | 5.14 | 3.0 x 10^-5^ |  |  |
| 218238_at | GTPBP4 | GTP binding protein 4 | 5.48 | 28.1 | 5.13 | 1.3 x 10^-6^ | regulation of cyclin-dependent protein kinase activity | nucleus |
| 227475_at | FOXQ1 | forkhead box Q1 | 16.14 | 82.7 | 5.13 | 2.6 x 10^-6^ | transcription | nucleus |
| 231221_at | CLEC16A | C-type lectin domain family 16, member A | 0.25 | 1.3 | 5.12 | 8.4 x 10^-5^ |  |  |
| 239352_at | SLC6A15 | solute carrier family 6 (neutral amino acid transporter), member 15 | 0.51 | 2.6 | 5.12 | 3.0 x 10^-7^ | transport | integral to plasma membrane |
| 201114_x_at | PSMA7 | proteasome (prosome, macropain) subunit, alpha type, 7 | 25.78 | 132.0 | 5.12 | 8.0 x 10^-9^ | ubiquitin-dependent protein catabolic process | proteasome complex |
| 217776_at | RDH11 | retinol dehydrogenase 11 (all-trans/9-cis/11-cis) | 15.26 | 78.2 | 5.12 | 2.2 x 10^-5^ | transport | intracellular |
| 231775_at | TNFRSF10A | tumor necrosis factor receptor superfamily, member 10a | 3.66 | 18.7 | 5.11 | 1.3 x 10^-6^ | apoptosis | membrane |
| 222740_at | ATAD2 | ATPase family, AAA domain containing 2 | 1.52 | 7.8 | 5.11 | 7.0 x 10^-3^ | transcription | nucleus |
| 209146_at | SC4MOL | sterol-C4-methyl oxidase-like | 41.81 | 213.6 | 5.11 | 1.1 x 10^-7^ | fatty acid metabolic process | endoplasmic reticulum |
| 200756_x_at | CALU | calumenin | 4.26 | 21.8 | 5.11 | 4.7 x 10^-7^ |  | extracellular region |
| 1553292_s_at | FLJ25006 | uncharacterized serine/threonine-protein kinase SgK494 | 0.71 | 3.6 | 5.11 | 1.1 x 10^-3^ | protein amino acid phosphorylation |  |
| 228783_at | BVES | blood vessel epicardial substance | 0.34 | 1.7 | 5.10 | 4.7 x 10^-8^ | muscle organ development | membrane |
| 204928_s_at | SLC10A3 | solute carrier family 10 (sodium/bile acid cotransporter family), member 3 | 2.01 | 10.2 | 5.10 | 1.4 x 10^-7^ | transport | membrane |
| 201393_s_at | IGF2R | insulin-like growth factor 2 receptor | 11.33 | 57.8 | 5.10 | 2.3 x 10^-6^ | transport | membrane fraction |
| 209544_at | RIPK2 | receptor-interacting serine-threonine kinase 2 | 0.50 | 2.5 | 5.10 | 1.3 x 10^-6^ | protein amino acid phosphorylation | intracellular |
| 221739_at | C19orf10 | chromosome 19 open reading frame 10 | 11.11 | 56.7 | 5.10 | 1.1 x 10^-4^ | positive regulation of cell proliferation | extracellular region |
| 240419_at | SLC6A15 | solute carrier family 6 (neutral amino acid transporter), member 15 | 0.31 | 1.6 | 5.10 | 3.5 x 10^-6^ | transport | integral to plasma membrane |
| 201695_s_at | NP | nucleoside phosphorylase | 5.14 | 26.2 | 5.10 | 1.8 x 10^-6^ | nucleobase, nucleoside, nucleotide and nucleic acid metabolic process | intracellular |
| 200743_s_at | TPP1 | tripeptidyl peptidase I | 10.24 | 52.2 | 5.10 | 7.3 x 10^-6^ | proteolysis | soluble fraction |
| 216041_x_at | GRN | granulin | 9.97 | 50.8 | 5.10 | 1.7 x 10^-7^ | signal transduction | extracellular region |
| 205807_s_at | TUFT1 | tuftelin 1 | 8.06 | 41.0 | 5.10 | 5.4 x 10^-7^ | ossification | extracellular region |
| 223001_at | OSTC | oligosaccharyltransferase complex subunit | 23.25 | 118.4 | 5.09 | 5.4 x 10^-6^ |  | membrane |
| 212024_x_at | FLII | flightless I homolog (Drosophila) | 10.00 | 51.0 | 5.09 | 1.6 x 10^-6^ | transcription | nucleus |
| 208893_s_at | DUSP6 | dual specificity phosphatase 6 | 8.77 | 44.7 | 5.09 | 3.5 x 10^-6^ | inactivation of MAPK activity | soluble fraction |
| 1569190_at | SCLT1 | sodium channel and clathrin linker 1 | 0.38 | 1.9 | 5.09 | 2.2 x 10^-7^ | clustering of voltage-gated sodium channels | cytoplasm |
| 1554158_at | ZMYND11 | zinc finger, MYND domain containing 11 | 0.17 | 0.9 | 5.09 | 9.0 x 10^-3^ | negative regulation of transcription from RNA polymerase II promoter | nucleus |
| 221931_s_at | SEH1L | SEH1-like (S. cerevisiae) | 7.99 | 40.6 | 5.09 | 3.0 x 10^-8^ | transport | nucleus |
| 213793_s_at | HOMER1 | homer homolog 1 (Drosophila) | 5.23 | 26.6 | 5.09 | 4.5 x 10^-6^ | skeletal muscle contraction | membrane fraction |
| 201577_at | NME1 | non-metastatic cells 1, protein (NM23A) expressed in | 11.17 | 56.8 | 5.08 | 2.1 x 10^-4^ | GTP biosynthetic process | ruffle |
| 210059_s_at | MAPK13 | mitogen-activated protein kinase 13 | 2.82 | 14.3 | 5.08 | 2.3 x 10^-6^ | protein amino acid phosphorylation |  |
| 215812_s_at | LOC653562 /// SLC6A10P /// SLC6A8 | hypothetical LOC653562 /// solute carrier family 6 (neurotransmitter transporter, creatine), member 10 (pseudogene) /// solute carrier family 6 (neurotransmitter transporter, creatine), member 8 | 2.16 | 11.0 | 5.07 | 8.6 x 10^-5^ | transport | integral to plasma membrane |
| 204319_s_at | RGS10 | regulator of G-protein signaling 10 | 4.70 | 23.8 | 5.07 | 6.6 x 10^-8^ | negative regulation of signal transduction |  |
| 1565162_s_at | MGST1 | microsomal glutathione S-transferase 1 | 43.64 | 221.4 | 5.07 | 7.2 x 10^-10^ | glutathione metabolic process | mitochondrion |
| 210970_s_at | IBTK | inhibitor of Bruton agammaglobulinemia tyrosine kinase | 14.48 | 73.4 | 5.07 | 1.4 x 10^-4^ | negative regulation of protein amino acid phosphorylation | cytoplasm |
| 45828_at | ATP5SL | ATP5S-like | 5.30 | 26.8 | 5.07 | 1.4 x 10^-8^ |  |  |
| 203414_at | MMD | monocyte to macrophage differentiation-associated | 2.42 | 12.3 | 5.07 | 6.4 x 10^-8^ | cytolysis | membrane fraction |
| 206688_s_at | CPSF4 | cleavage and polyadenylation specific factor 4, 30kDa | 4.05 | 20.5 | 5.07 | 6.3 x 10^-8^ | mRNA processing | nucleus |
| 228587_at | FAM83G | family with sequence similarity 83, member G | 0.91 | 4.6 | 5.07 | 2.3 x 10^-6^ |  |  |
| 215411_s_at | TRAF3IP2 | TRAF3 interacting protein 2 | 6.22 | 31.5 | 5.06 | 1.7 x 10^-8^ | intracellular signaling cascade |  |
| 224010_at | ANAPC11 | anaphase promoting complex subunit 11 | 0.40 | 2.0 | 5.06 | 6.6 x 10^-4^ | cell cycle | nucleus |
| 236663_at | --- | --- | 0.53 | 2.7 | 5.06 | 1.4 x 10^-3^ |  |  |
| 216971_s_at | PLEC1 | plectin 1, intermediate filament binding protein 500kDa | 5.22 | 26.4 | 5.06 | 4.4 x 10^-4^ |  | cytoplasm |
| 203550_s_at | C1orf2 | chromosome 1 open reading frame 2 | 3.75 | 19.0 | 5.06 | 1.4 x 10^-6^ |  | membrane |
| 208886_at | H1F0 | H1 histone family, member 0 | 18.88 | 95.5 | 5.06 | 7.6 x 10^-7^ | nucleosome assembly | nucleosome |
| 214782_at | CTTN | cortactin | 0.86 | 4.3 | 5.06 | 1.4 x 10^-3^ | receptor-mediated endocytosis | ruffle |
| 214097_at | RPS21 | ribosomal protein S21 | 5.12 | 25.9 | 5.06 | 8.4 x 10^-5^ | translation | intracellular |
| 210406_s_at | RAB6A /// RAB6C | RAB6A, member RAS oncogene family /// RAB6C, member RAS oncogene family | 27.48 | 138.9 | 5.06 | 5.9 x 10^-8^ | transport | Golgi membrane |
| 234989_at | NCRNA00084 | non-protein coding RNA 84 | 37.20 | 187.9 | 5.05 | 1.7 x 10^-4^ |  |  |
| 225032_at | FNDC3B | fibronectin type III domain containing 3B | 12.39 | 62.5 | 5.04 | 3.8 x 10^-10^ | positive regulation of fat cell differentiation | endoplasmic reticulum |
| 209096_at | UBE2V2 | ubiquitin-conjugating enzyme E2 variant 2 | 13.01 | 65.6 | 5.04 | 3.9 x 10^-6^ | protein polyubiquitination | nucleus |
| 202158_s_at | CUGBP2 | CUG triplet repeat, RNA binding protein 2 | 1.95 | 9.8 | 5.04 | 5.2 x 10^-4^ | mRNA splice site selection | soluble fraction |
| 215074_at | MYO1B | myosin IB | 0.68 | 3.4 | 5.04 | 3.9 x 10^-5^ |  | myosin complex |
| 222451_s_at | ZDHHC9 | zinc finger, DHHC-type containing 9 | 4.47 | 22.6 | 5.04 | 1.3 x 10^-6^ |  | Golgi membrane |
| 226414_s_at | ANAPC11 /// LOC100131844 | anaphase promoting complex subunit 11 /// similar to anaphase promoting complex subunit 11 | 21.05 | 106.1 | 5.04 | 7.0 x 10^-6^ | cell cycle | nucleus |
| 201181_at | GNAI3 | guanine nucleotide binding protein (G protein), alpha inhibiting activity polypeptide 3 | 3.86 | 19.4 | 5.04 | 7.4 x 10^-7^ | transport | cytoplasm |
| 243276_at | ALS2CL | ALS2 C-terminal like | 1.76 | 8.9 | 5.04 | 4.2 x 10^-5^ | endosome organization | cytoplasm |
| 244864_at | --- | --- | 0.45 | 2.3 | 5.04 | 8.4 x 10^-3^ |  |  |
| 1569154_a_at | --- | --- | 0.27 | 1.4 | 5.03 | 1.7 x 10^-4^ |  |  |
| 217913_at | VPS4A | vacuolar protein sorting 4 homolog A (S. cerevisiae) | 5.25 | 26.4 | 5.03 | 2.9 x 10^-7^ | cytokinesis | ESCRT III complex |
| 1562415_a_at | SPOCD1 | SPOC domain containing 1 | 0.43 | 2.2 | 5.03 | 1.4 x 10^-3^ | transcription |  |
| 201976_s_at | MYO10 | myosin X | 11.88 | 59.7 | 5.03 | 1.2 x 10^-5^ | signal transduction | cytoskeleton |
| 218982_s_at | MRPS17 /// ZNF713 | mitochondrial ribosomal protein S17 /// zinc finger protein 713 | 8.31 | 41.8 | 5.03 | 4.0 x 10^-5^ | transcription | intracellular |
| 208824_x_at | PCTK1 | PCTAIRE protein kinase 1 | 3.99 | 20.1 | 5.03 | 6.6 x 10^-5^ | protein amino acid phosphorylation | cytoplasm |
| 201144_s_at | EIF2S1 | eukaryotic translation initiation factor 2, subunit 1 alpha, 35kDa | 15.20 | 76.3 | 5.02 | 2.5 x 10^-7^ | translation | nucleus |
| 218744_s_at | PACSIN3 | protein kinase C and casein kinase substrate in neurons 3 | 0.77 | 3.9 | 5.02 | 5.6 x 10^-5^ | endocytosis | cytoplasm |
| 212265_at | QKI | quaking homolog, KH domain RNA binding (mouse) | 16.05 | 80.6 | 5.02 | 8.0 x 10^-11^ | mRNA processing | nucleus |
| 212563_at | BOP1 | block of proliferation 1 | 1.11 | 5.6 | 5.01 | 8.3 x 10^-4^ | maturation of LSU-rRNA from tricistronic rRNA transcript (SSU-rRNA, 5.8S rRNA, LSU-rRNA) | nucleus |
| 219631_at | LRP12 | low density lipoprotein-related protein 12 | 2.03 | 10.2 | 5.01 | 1.9 x 10^-5^ | endocytosis | integral to plasma membrane |
| 209433_s_at | PPAT | phosphoribosyl pyrophosphate amidotransferase | 2.76 | 13.8 | 5.01 | 3.0 x 10^-5^ | purine nucleotide biosynthetic process |  |
| 218700_s_at | RAB7L1 | RAB7, member RAS oncogene family-like 1 | 1.59 | 8.0 | 5.01 | 1.2 x 10^-3^ | transport | plasma membrane |
| 201573_s_at | ETF1 | eukaryotic translation termination factor 1 | 9.18 | 46.0 | 5.01 | 1.4 x 10^-6^ | translation | cytoplasm |
| 216505_x_at | RPS10P5 | ribosomal protein S10 pseudogene 5 | 12.65 | 63.4 | 5.01 | 1.5 x 10^-6^ |  | ribosome |
| 205083_at | AOX1 | aldehyde oxidase 1 | 0.74 | 3.7 | 5.01 | 2.9 x 10^-5^ | oxygen and reactive oxygen species metabolic process | cytoplasm |
| 221994_at | PDLIM5 | PDZ and LIM domain 5 | 0.39 | 1.9 | 5.00 | 2.9 x 10^-3^ |  | membrane fraction |
| 212702_s_at | BICD2 | bicaudal D homolog 2 (Drosophila) | 3.91 | 19.6 | 5.00 | 1.1 x 10^-6^ | transport | cytoplasm |
| 242677_at | --- | --- | 0.34 | 1.7 | 5.00 | 4.4 x 10^-4^ |  |  |
| 205361_s_at | PFDN4 | prefoldin subunit 4 | 7.80 | 39.0 | 5.00 | 1.2 x 10^-8^ | protein folding | cytosol |

^1^ The list consist of the 1,828 probe sets (1,161 genes) that were significantly (p<0.01) overexpressed >5-fold in basal cells (n=5) compared to differentiated epithelium (n=12) listed in desecending order of basal cell / differentiated epithelium expression ratio.

^2^ p value following Benjamini-Hochberg correction.

^3^ First Gene Ontology molecular function assignment.

^4^ First Gene Ontology cellular compartment assignment.
