# Supplementary material for: The Human Airway Epithelial Basal Cell Transcriptome
Source: PLoS One. 2011 May 4;6(5):e18378. doi: 10.1371/journal.pone.0018378 (PMC3087716; doi:10.1371/journal.pone.0018378)
Supplement: Table S2 — Basal Cell-specific Genes Shared by Human and Mouse. (DOC) [file pone.0018378.s002.doc]

| **Gene symbol** | **Gene title** | **ProbesetID** | **Basal / differentiated epithelium expression ratio** |
| --- | --- | --- | --- |
|  |  |  |  |
| AHNAK2 | AHNAK nucleoprotein 2 | 212992_at | 6.9 |
| AMOTL1 | angiomotin-like 1 | 225459_at | 24.5 |
| ANKRD29 | ankyrin repeat domain 29 | 238332_at | 9.1 |
| ARNTL2 | aryl hydrocarbon receptor nuclear translocator-like 2 | 224204_x_at | 44.9 |
| ARPC2 | actin related protein 2/3 complex, subunit 2, 34kDa | 207988_s_at | 6.8 |
| ARPC5L | actin related protein 2/3 complex, subunit 5-like | 226915_s_at | 5.8 |
| ARSJ | arylsulfatase family, member J | 219973_at | 12.7 |
| ARTN | artemin | 210237_at | 21.7 |
| AXL | AXL receptor tyrosine kinase | 202686_s_at | 6.0 |
| BMP1 | bone morphogenetic protein 1 | 205574_x_at | 12.3 |
| BNC1 | basonuclin 1 | 1552487_a_at | 69.7 |
| BNIP2 | BCL2/adenovirus E1B 19kDa interacting protein 2 | 209308_s_at | 6.7 |
| CALD1 | caldesmon 1 | 212077_at | 109.6 |
| CAPG | capping protein (actin filament), gelsolin-like | 201850_at | 9.9 |
| CAV1 | caveolin 1, caveolae protein, 22kDa | 212097_at | 169.5 |
| CCDC3 | coiled-coil domain containing 3 | 223316_at | 7.1 |
| CCDC88A | coiled-coil domain containing 88A | 225045_at | 13.2 |
| CD109 | CD109 molecule | 229900_at | 24.4 |
| CDH13 | cadherin 13, H-cadherin (heart) | 204726_at | 16.3 |
| COL17A1 | collagen, type XVII, alpha 1 | 204636_at | 353.0 |
| COL4A2 | collagen, type IV, alpha 2 | 211964_at | 7.2 |
| COL7A1 | collagen, type VII, alpha 1 | 217312_s_at | 15.1 |
| CTH | cystathionase (cystathionine gamma-lyase) | 217127_at | 18.5 |
| DKK3 | dickkopf homolog 3 (*Xenopus laevis*) | 230508_at | 27.9 |
| DNAJB9 | DnaJ (Hsp40) homolog, subfamily B, member 9 | 202843_at | 6.7 |
| DNAJC21 | DnaJ (Hsp40) homolog, subfamily C, member 21 | 238336_s_at | 5.9 |
| DSC2 | desmocollin 2 | 226817_at | 35.1 |
| DSC3 | desmocollin 3 | 206032_at | 16.9 |
| DST | dystonin | 216918_s_at | 30.2 |
| DUSP7 | dual specificity phosphatase 7 | 213848_at | 33.1 |
| EHD2 | EH-domain containing 2 | 221870_at | 123.7 |
| ELOVL4 | elongation of very long chain fatty acids (FEN1/Elo2, SUR4/Elo3, yeast)-like 4 | 219532_at | 19.6 |
| ELOVL6 | ELOVL family member 6, elongation of long chain fatty acids (FEN1/Elo2, SUR4/Elo3-like, yeast) | 204256_at | 23.2 |
| ELOVL7 | ELOVL family member 7, elongation of long chain fatty acids (yeast) | 227180_at | 12.2 |
| EPHB4 | EPH receptor B4 | 202894_at | 6.0 |
| ETF1 | eukaryotic translation termination factor 1 | 201573_s_at | 5.0 |
| ETV4 | ets variant 4 | 1554576_a_at | 10.8 |
| FADS1 | fatty acid desaturase 1 | 208962_s_at | 20.3 |
| FADS2 | fatty acid desaturase 2 | 202218_s_at | 18.9 |
| FAM110C | family with sequence similarity 110, member C | 226863_at | 7.1 |
| FAM126A | family with sequence similarity 126, member A | 223625_at | 12.7 |
| FBLN1 | #N/A | 201787_at | 13.2 |
| FBN2 | fibrillin 2 | 203184_at | 5.9 |
| FLNA | filamin A, alpha (actin binding protein 280) | 213746_s_at | 19.1 |
| FLNB | filamin B, beta (actin binding protein 278) | 208613_s_at | 7.7 |
| FOSL1 | FOS-like antigen 1 | 204420_at | 30.7 |
| FOXA2 | forkhead box A2 | 40284_at | 11.4 |
| FSCN1 | fascin homolog 1, actin-bundling protein (*Strongylocentrotus purpuratus*) | 201564_s_at | 89.0 |
| FSTL3 | follistatin-like 3 (secreted glycoprotein) | 203592_s_at | 25.4 |
| GPC1 | glypican 1 | 202755_s_at | 7.8 |
| GPR126 | G protein-coupled receptor 126 | 213094_at | 10.2 |
| GPR87 | G protein-coupled receptor 87 | 219936_s_at | 8.7 |
| HDGFRP3 | hepatoma-derived growth factor, related protein 3 | 209526_s_at | 6.5 |
| HSPC159 | galectin-related protein | 219998_at | 77.6 |
| IL1RN | interleukin 1 receptor antagonist | 212657_s_at | 28.7 |
| IL20RB | interleukin 20 receptor beta | 228575_at | 18.1 |
| INF2 | inverted formin, FH2 and WH2 domain containing | 224469_s_at | 15.8 |
| ITGA3 | integrin, alpha 3 (antigen CD49C, alpha 3 subunit of VLA-3 receptor) | 201474_s_at | 77.6 |
| ITGB1 | integrin, beta 1 (fibronectin receptor, beta polypeptide, antigen CD29 includes MDF2, MSK12) | 1553530_a_at | 12.8 |
| JUB | jub, ajuba homolog (*Xenopus laevis*) | 225806_at | 10.0 |
| JUP | junction plakoglobin | 201015_s_at | 5.9 |
| KIAA1949 | KIAA1949 | 224927_at | 13.5 |
| KLHL29 | kelch-like 29 (Drosophila) | 1554262_s_at | 10.6 |
| KRT17 | keratin 17 | 205157_s_at | 50.3 |
| KRT5 | keratin 5 | 201820_at | 8.6 |
| LAMA3 | laminin, alpha 3 | 203726_s_at | 27.6 |
| LAMB3 | laminin, beta 3 | 209270_at | 18.7 |
| LEPR x L1 | leprecan-like 1 | 218717_s_at | 57.0 |
| LZIC | leucine zipper and CTNNBIP1 domain containing | 226087_at | 5.7 |
| MAP4K4 | mitogen-activated protein kinase kinase kinase kinase 4 | 206571_s_at | 7.8 |
| MMD | monocyte to macrophage differentiation-associated | 203414_at | 5.1 |
| MREG | melanoregulin | 219648_at | 8.6 |
| MSN | moesin | 200600_at | 10.5 |
| MTHFD1L | methylenetetrahydrofolate dehydrogenase (NADP+ dependent) 1-like | 231094_s_at | 22.5 |
| MYO5A | myosin VA (heavy chain 12, myoxin) | 204527_at | 8.9 |
| NDRG1 | N-myc downstream regulated 1 | 200632_s_at | 50.3 |
| NEDD4 | neural precursor cell expressed, developmentally down-regulated 4 | 213012_at | 8.5 |
| NTN4 | netrin 4 | 223315_at | 5.5 |
| PARD6G | par-6 partitioning defective 6 homolog gamma (*C. elegans*) | 232132_at | 9.5 |
| PDGFC | platelet derived growth factor C | 218718_at | 6.2 |
| PHGDH | phosphoglycerate dehydrogenase | 201397_at | 8.2 |
| PITPNC1 | phosphatidylinositol transfer protein, cytoplasmic 1 | 219155_at | 6.3 |
| PLCH2 | phospholipase C, eta 2 | 206080_at | 8.4 |
| PMEPA1 | prostate transmembrane protein, androgen induced 1 | 222449_at | 32.1 |
| PRAGMIN | homolog of rat pragma of Rnd2 | 235085_at | 15.7 |
| PRKCDBP | protein kinase C, delta binding protein | 213010_at | 6.0 |
| PROCR | protein C receptor, endothelial (EPCR) | 203650_at | 33.1 |
| PTPN14 | protein tyrosine phosphatase, non-receptor type 14 | 205503_at | 6.2 |
| PVRL1 | poliovirus receptor-related 1 (herpesvirus entry mediator C) | 225211_at | 6.5 |
| QKI | quaking homolog, KH domain RNA binding (mouse) | 212263_at | 7.3 |
| RAB38 | RAB38, member RAS oncogene family | 219412_at | 13.8 |
| RAP2A | RAP2A, member of RAS oncogene family | 221830_at | 5.4 |
| RAP2B | RAP2B, member of RAS oncogene family | 213923_at | 8.5 |
| RAP2C | RAP2C, member of RAS oncogene family | 218668_s_at | 6.8 |
| REEP4 | receptor accessory protein 4 | 218777_at | 5.4 |
| RGS10 | regulator of G-protein signaling 10 | 204319_s_at | 5.1 |
| S100A10 | S100 calcium binding protein A10 | 200872_at | 8.3 |
| SACS | spastic ataxia of Charlevoix-Saguenay (sacsin) | 213262_at | 6.7 |
| SCEL | sciellin | 206884_s_at | 194.6 |
| SCHIP1 | schwannomin interacting protein 1 | 204030_s_at | 6.3 |
| SERPINB5 | serpin peptidase inhibitor, clade B (ovalbumin), member 5 | 204855_at | 20.1 |
| SERPINE2 | serpin peptidase inhibitor, clade E (nexin, plasminogen activator inhibitor type 1), member 2 | 212190_at | 91.4 |
| SH3RF2 | SH3 domain containing ring finger 2 | 228892_at | 11.6 |
| SHMT2 | serine hydroxymethyltransferase 2 (mitochondrial) | 214095_at | 24.9 |
| SLC2A9 | Solute carrier family 2 (facilitated glucose transporter), member 9 | 232678_at | 6.2 |
| SLC6A8 | solute carrier family 6 (neurotransmitter transporter, creatine), member 8 | 213843_x_at | 5.5 |
| SNAI2 | snail homolog 2 (Drosophila) | 213139_at | 12.0 |
| SNCG | synuclein, gamma (breast cancer-specific protein 1) | 209877_at | 7.1 |
| STARD4 | StAR-related lipid transfer (START) domain containing 4 | 226390_at | 12.0 |
| TGFB1 | transforming growth factor, beta 1 | 203085_s_at | 5.7 |
| TGFB1I1 | transforming growth factor beta 1 induced transcript 1 | 209651_at | 14.6 |
| TGFBI | transforming growth factor, beta-induced, 68kDa | 201506_at | 36.1 |
| TINAGL1 | tubulointerstitial nephritis antigen-like 1 | 219058_x_at | 20.5 |
| TNC | tenascin C | 201645_at | 13.7 |
| TP63 | tumor protein p63 | 209863_s_at | 8.9 |
| TPBG | trophoblast glycoprotein | 203476_at | 10.2 |
| TUBB6 | tubulin, beta 6 | 209191_at | 127.8 |
| UNC5B | Unc-5 homolog B (*C. elegans*) | 213100_at | 7.2 |
| YIF1B | Yip1 interacting factor homolog B (*S. cerevisiae*) | 231211_s_at | 6.2 |

^1^ Overlap of human airway basal cell-enriched genome (p< 0.01, fold-change >5) with mouse basal cell genome (Table S5 of Rock et al [1]) was assessed, and the top human probe set representing each gene along with the basal cell / differentiation epithelium expression ratio is tabulated.

**Reference List**

1. Rock JR, Onaitis MW, Rawlins EL, Lu Y, Clark CP, Xue Y, Randell SH, Hogan BL (2009) Basal cells as stem cells of the mouse trachea and human airway epithelium. Proc Natl Acad Sci U S A 106: 12771-12775.
