# Supplementary material for: The Human Airway Epithelial Basal Cell Transcriptome
Source: PLoS One. 2011 May 4;6(5):e18378. doi: 10.1371/journal.pone.0018378 (PMC3087716; doi:10.1371/journal.pone.0018378)
Supplement: Table S3 — Genes Comprising the Significant GATHER KEGG Categories of the Human Basal Cell Signature. (DOC) [file pone.0018378.s003.doc]

| **ProbeSetID** | **Gene symbol** | **Gene title** | **Mean expression in differentiated epithelium** | **Mean expression in basal cells** | **Basal/ differentiated epithelium ratio** | **p value^2^** |
| --- | --- | --- | --- | --- | --- | --- |
|  | |  |  |  |  |  |
| **Focal adhesion** | |  |  |  |  |  |
| 211160_x_at | ACTN1 | actinin, alpha 1 | 4.2 | 70.8 | 17.0 | 5.8 x 10^-3^ |
| 212607_at | AKT3 | v-akt murine thymoma viral oncogene homolog 3 (protein kinase B, gamma) | 1.5 | 9.3 | 6.2 | 6.8 x 10^-7^ |
| 212097_at | CAV1 | caveolin 1, caveolae protein, 22kDa | 1.0 | 177.4 | 169.5 | 1.0 x 10^-3^ |
| 203324_s_at | CAV2 | caveolin 2 | 10.3 | 183.9 | 17.9 | 9.9 x 10^-11^ |
| 200951_s_at | CCND2 | cyclin D2 | 0.5 | 12.4 | 26.2 | 2.5 x 10^-7^ |
| 208727_s_at | CDC42 | cell division cycle 42 (GTP binding protein, 25kDa) | 19.3 | 106.1 | 5.5 | 1.7 x 10^-3^ |
| 211964_at | COL4A2 | collagen, type IV, alpha 2 | 0.7 | 5.1 | 7.2 | 6.7 x 10^-3^ |
| 213992_at | COL4A6 | collagen, type IV, alpha 6 | 5.1 | 39.3 | 7.6 | 6.6 x 10^-8^ |
| 202224_at | CRK | v-crk sarcoma virus CT10 oncogene homolog (avian) | 6.5 | 34.6 | 5.3 | 1.6 x 10^-9^ |
| 209190_s_at | DIAPH1 | diaphanous homolog 1 (Drosophila) | 8.2 | 47.4 | 5.8 | 4.2 x 10^-6^ |
| 201984_s_at | EGFR | epidermal growth factor receptor (erythroblastic leukemia viral (v-erb-b) oncogene homolog, avian) | 9.2 | 93.6 | 10.2 | 9.1 x 10^-7^ |
| 206412_at | FER | fer (fps/fes related) tyrosine kinase | 1.5 | 8.6 | 5.7 | 5.0 x 10^-7^ |
| 213746_s_at | FLNA | filamin A, alpha (actin binding protein 280) | 2.0 | 38.0 | 19.1 | 2.1 x 10^-5^ |
| 208613_s_at | FLNB | filamin B, beta (actin binding protein 278) | 13.4 | 103.5 | 7.7 | 6.9 x 10^-6^ |
| 210105_s_at | FYN | FYN oncogene related to SRC, FGR, Y x S | 4.1 | 24.5 | 5.9 | 7.6 x 10^-6^ |
| 201474_s_at | ITGA3 | integrin, alpha 3 (antigen CD49C, alpha 3 subunit of VLA-3 receptor) | 3.2 | 50.1 | 15.8 | 5.0 x 10^-9^ |
| 201389_at | ITGA5 | integrin, alpha 5 (fibronectin receptor, alpha polypeptide) | 0.7 | 18.3 | 27.3 | 2.9 x 10^-6^ |
| 201656_at | ITGA6 | integrin, alpha 6 | 2.6 | 129.8 | 50.8 | 5.9 x 10^-9^ |
| 1553530_a_at | ITGB1 | integrin, beta 1 (fibronectin receptor, beta polypeptide, antigen CD29 includes MDF2, MSK12) | 16.9 | 216.8 | 12.8 | 7.4 x 10^-7^ |
| 204990_s_at | ITGB4 | integrin, beta 4 | 11.0 | 106.2 | 9.6 | 3.0 x 10^-7^ |
| 226535_at | ITGB6 | integrin, beta 6 | 5.4 | 44.6 | 8.3 | 6.3 x 10^-3^ |
| 203726_s_at | LAMA3 | laminin, alpha 3 | 5.4 | 148.4 | 27.6 | 1.3 x 10^-5^ |
| 201505_at | LAMB1 | laminin, beta 1 | 1.8 | 31.3 | 17.3 | 2.6 x 10^-5^ |
| 209270_at | LAMB3 | laminin, beta 3 | 15.9 | 297.3 | 18.7 | 2.8 x 10^-9^ |
| 200770_s_at | LAMC1 | laminin, gamma 1 (formerly LAMB2) | 3.5 | 45.0 | 12.9 | 5.5 x 10^-8^ |
| 202267_at | LAMC2 | laminin, gamma 2 | 11.8 | 417.2 | 35.2 | 1.6 x 10^-6^ |
| 202670_at | MAP2K1 | mitogen-activated protein kinase kinase 1 | 8.4 | 58.5 | 7.0 | 1.5 x 10^-9^ |
| 203510_at | MET | met proto-oncogene (hepatocyte growth factor receptor) | 30.9 | 195.3 | 6.3 | 3.8 x 10^-7^ |
| 202647_s_at | NRAS | neuroblastoma RAS viral (v-ras) oncogene homolog | 5.1 | 32.2 | 6.3 | 5.1 x 10^-6^ |
| 37966_at | PARVB | parvin, beta | 0.3 | 1.4 | 5.5 | 5.0 x 10^-6^ |
| 218718_at | PDGFC | platelet derived growth factor C | 12.7 | 78.6 | 6.2 | 2.6 x 10^-7^ |
| 209652_s_at | PGF | placental growth factor | 0.4 | 7.3 | 17.9 | 7.2 x 10^-7^ |
| 204053_x_at | PT EN | phosphatase and tensin homolog | 10.1 | 51.8 | 5.1 | 2.0 x 10^-4^ |
| 206482_at | PTK6 | PTK6 protein tyrosine kinase 6 | 2.0 | 13.0 | 6.7 | 7.8 x 10^-6^ |
| 213603_s_at | RAC2 | ras-related C3 botulinum toxin substrate 2 (rho family, small GTP-binding protein Rac2) | 6.9 | 50.2 | 7.3 | 1.8 x 10^-4^ |
| 212647_at | RRAS | related RAS viral (r-ras) oncogene homolog | 1.9 | 59.5 | 30.8 | 1.5 x 10^-7^ |
| 208456_s_at | RRAS2 | related RAS viral (r-ras) oncogene homolog 2 | 6.0 | 44.0 | 7.3 | 5.9 x 10^-6^ |
| 214853_s_at | SHC1 | SHC (Src homology 2 domain containing) transforming protein 1 | 14.9 | 204.0 | 13.7 | 3.3 x 10^-8^ |
| 201109_s_at | THBS1 | thrombospondin 1 | 0.5 | 59.4 | 110.4 | 7.3 x 10^-3^ |
| 201645_at | TNC | tenascin C | 8.6 | 118.0 | 13.7 | 3.2 x 10^-6^ |
| 200931_s_at | VCL | vinculin | 9.6 | 55.8 | 5.8 | 1.5 x 10^-6^ |
| 211527_x_at | VEGFA | vascular endothelial growth factor A | 4.2 | 35.1 | 8.3 | 1.6 x 10^-5^ |
| 209946_at | VEGFC | vascular endothelial growth factor C | 1.3 | 9.1 | 7.2 | 2.2 x 10^-5^ |
| 200808_s_at | ZYX | zyxin | 3.1 | 26.2 | 8.3 | 2.6 x 10^-6^ |
|  |  |  |  |  |  |  |
| **ECM-receptor interactions** | | |  |  |  |  |
| 201109_s_at | THBS1 | thrombospondin 1 | 0.5 | 59.4 | 110.4 | 7.3 x 10^-3^ |
| 201656_at | ITGA6 | integrin, alpha 6 | 2.6 | 129.8 | 50.8 | 5.9 x 10^-9^ |
| 202267_at | LAMC2 | laminin, gamma 2 | 11.8 | 417.2 | 35.2 | 1.6 x 10^-6^ |
| 203726_s_at | LAMA3 | laminin, alpha 3 | 5.4 | 148.4 | 27.6 | 1.3 x 10^-5^ |
| 201389_at | ITGA5 | integrin, alpha 5 (fibronectin receptor, alpha polypeptide) | 0.7 | 18.3 | 27.3 | 2.9 x 10^-6^ |
| 209270_at | LAMB3 | laminin, beta 3 | 15.9 | 297.3 | 18.7 | 2.8 x 10^-9^ |
| 201505_at | LAMB1 | laminin, beta 1 | 1.8 | 31.3 | 17.3 | 2.6 x 10^-5^ |
| 201474_s_at | ITGA3 | integrin, alpha 3 (antigen CD49C, alpha 3 subunit of VLA-3 receptor) | 3.2 | 50.1 | 15.8 | 5.0 x 10^-9^ |
| 201645_at | TNC | tenascin C | 8.6 | 118.0 | 13.7 | 3.2 x 10^-6^ |
| 200770_s_at | LAMC1 | laminin, gamma 1 (formerly LAMB2) | 3.5 | 45.0 | 12.9 | 5.5 x 10^-8^ |
| 1553530_a_at | ITGB1 | integrin, beta 1 (fibronectin receptor, beta polypeptide, antigen CD29 includes MDF2, MSK12) | 16.9 | 216.8 | 12.8 | 7.4 x 10^-7^ |
| 204490_s_at | CD44 | CD44 molecule (Indian blood group) | 5.9 | 58.2 | 9.9 | 1.4 x 10^-8^ |
| 204990_s_at | ITGB4 | integrin, beta 4 | 11.0 | 106.2 | 9.6 | 3.0 x 10^-7^ |
| 201286_at | SDC1 | syndecan 1 | 12.0 | 110.9 | 9.3 | 9.5 x 10^-8^ |
| 226535_at | ITGB6 | integrin, beta 6 | 5.4 | 44.6 | 8.3 | 6.3 x 10^-3^ |
| 213992_at | COL4A6 | collagen, type IV, alpha 6 | 5.1 | 39.3 | 7.6 | 6.6 x 10^-8^ |
| 211964_at | COL4A2 | collagen, type IV, alpha 2 | 0.7 | 5.1 | 7.2 | 6.7 x 10^-3^ |
| 201655_s_at | HSPG2 | heparan sulfate proteoglycan 2 | 4.5 | 26.8 | 6.0 | 1.4 x 10^-5^ |
|  |  |  |  |  |  |  |
| **Adherens junctions** | | |  |  |  |  |
| 204165_at | WASF1 | WAS protein family, member 1 | 0.6 | 12.1 | 20.5 | 2.4 x 10^-7^ |
| 211160_x_at | ACTN1 | actinin, alpha 1 | 4.2 | 70.8 | 17.0 | 5.8 x 10^-3^ |
| 213139_at | SNAI2 | snail homolog 2 (Drosophila) | 6.0 | 71.9 | 12.0 | 2.0 x 10^-4^ |
| 201984_s_at | EGFR | epidermal growth factor receptor (erythroblastic leukemia viral (v-erb-b) oncogene homolog, avian) | 9.2 | 93.6 | 10.2 | 9.1 x 10^-7^ |
| 224793_s_at | TGFBR1 | transforming growth factor, beta receptor 1 | 7.3 | 54.4 | 7.5 | 7.4 x 10^-8^ |
| 212072_s_at | CSNK2A1 | casein kinase 2, alpha 1 polypeptide | 12.7 | 93.5 | 7.3 | 5.9 x 10^-7^ |
| 213603_s_at | RAC2 | ras-related C3 botulinum toxin substrate 2 (rho family, small GTP-binding protein Rac2) | 6.9 | 50.2 | 7.3 | 2.0 x 10^-4^ |
| 225211_at | PVRL1 | poliovirus receptor-related 1 (herpesvirus entry mediator C) | 3.3 | 21.6 | 6.5 | 3.6 x 10^-5^ |
| 203510_at | MET | met proto-oncogene (hepatocyte growth factor receptor) | 30.9 | 195.3 | 6.3 | 3.8 x 10^-7^ |
| 210105_s_at | FYN | FYN oncogene related to SRC, FGR, Y x S | 4.1 | 24.5 | 5.9 | 7.6 x 10^-6^ |
| 207121_s_at | MAPK6 | mitogen-activated protein kinase 6 | 27.7 | 163.3 | 5.9 | 3.8 x 10^-11^ |
| 200931_s_at | VCL | vinculin | 9.6 | 55.8 | 5.8 | 1.5 x 10^-6^ |
| 206412_at | FER | fer (fps/fes related) tyrosine kinase | 1.5 | 8.6 | 5.7 | 5.0 x 10^-7^ |
| 202933_s_at | YES1 | v-yes-1 Yamaguchi sarcoma viral oncogene homolog 1 | 13.1 | 73.1 | 5.6 | 5.4 x 10^-7^ |
| 208727_s_at | CDC42 | cell division cycle 42 (GTP-binding protein, 25kDa) | 19.3 | 106.1 | 5.5 | 1.7 x 10^-3^ |
| 214168_s_at | TJP1 | tight junction protein 1 (zona occludens 1) | 4.4 | 22.5 | 5.2 | 1.9 x 10^-6^ |
|  |  |  |  |  |  |  |
| **MAPK signaling pathway** | | |  |  |  |  |
| 210984_x_at | EGFR | epidermal growth factor receptor (erythroblastic leukemia viral (v-erb-b) oncogene homolog, avian) | 2.4 | 15.1 | 6.4 | 2.4 x 10^-3^ |
| 212607_at | AKT3 | v-akt murine thymoma viral oncogene homolog 3 (protein kinase B, gamma) | 1.5 | 9.3 | 6.2 | 6.8 x 10^-7^ |
| 200779_at | ATF4 | activating transcription factor 4 (tax-responsive enhancer element B67) | 42.6 | 301.5 | 7.1 | 1.3 x 10^-10^ |
| 201853_s_at | CDC25B | cell division cycle 25 homolog B (S. pombe) | 2.3 | 21.6 | 9.3 | 5.2 x 10^-6^ |
| 208727_s_at | CDC42 | cell division cycle 42 (GTP-binding protein, 25kDa) | 19.3 | 106.1 | 5.5 | 1.7 x 10^-3^ |
| 202224_at | CRK | v-crk sarcoma virus CT10 oncogene homolog (avian) | 6.5 | 34.6 | 5.3 | 1.6 x 10^-9^ |
| 209457_at | DUSP5 | dual specificity phosphatase 5 | 14.3 | 76.0 | 5.3 | 3.5 x 10^-4^ |
| 208892_s_at | DUSP6 | dual specificity phosphatase 6 | 13.6 | 87.4 | 6.4 | 3.0 x 10^-7^ |
| 214793_at | DUSP7 | dual specificity phosphatase 7 | 0.2 | 4.8 | 20.2 | 3.1 x 10^-7^ |
| 227271_at | FGF11 | fibroblast growth factor 11 | 1.3 | 8.3 | 6.2 | 2.5 x 10^-6^ |
| 204422_s_at | FGF2 | fibroblast growth factor 2 (basic) | 0.2 | 1.8 | 7.7 | 2.7 x 10^-3^ |
| 213746_s_at | FLNA | filamin A, alpha (actin binding protein 280) | 2.0 | 38.0 | 19.1 | 2.1 x 10^-5^ |
| 208613_s_at | FLNB | filamin B, beta (actin binding protein 278) | 13.4 | 103.5 | 7.7 | 6.9 x 10^-6^ |
| 203725_at | GADD45A | growth arrest and DNA-damage-inducible, alpha | 13.3 | 135.4 | 10.2 | 4.4 x 10^-10^ |
| 211936_at | HSPA5 | heat shock 70kDa protein 5 (glucose-regulated protein, 78kDa) | 92.4 | 565.1 | 6.1 | 1.5 x 10^-6^ |
| 200690_at | HSPA9 | heat shock 70kDa protein 9 (mortalin) | 4.1 | 21.6 | 5.3 | 7.0 x 10^-5^ |
| 205067_at | IL1B | interleukin 1, beta | 1.8 | 20.9 | 11.7 | 6.3 x 10^-3^ |
| 202670_at | MAP2K1 | mitogen-activated protein kinase kinase 1 | 8.4 | 58.5 | 7.0 | 1.5 x 10^-9^ |
| 206571_s_at | MAP4K4 | mitogen-activated protein kinase kinase kinase kinase 4 | 3.4 | 26.5 | 7.8 | 1.3 x 10^-6^ |
| 210058_at | MAPK13 | mitogen-activated protein kinase 13 | 4.0 | 24.8 | 6.2 | 1.4 x 10^-5^ |
| 207121_s_at | MAPK6 | mitogen-activated protein kinase 6 | 27.7 | 163.3 | 5.9 | 3.8 x 10^-11^ |
| 202787_s_at | MAPKAPK3 | mitogen-activated protein kinase-activated protein kinase 3 | 1.9 | 9.6 | 5.1 | 6.2 x 10^-5^ |
| 202431_s_at | MYC | v-myc myelocytomatosis viral oncogene homolog (avian) | 9.6 | 90.1 | 9.4 | 8.1 x 10^-5^ |
| 202647_s_at | NRAS | neuroblastoma RAS viral (v-ras) oncogene homolog | 5.1 | 32.2 | 6.3 | 5.1 x 10^-6^ |
| 221027_s_at | PLA2G12A | phospholipase A2, group XIIA | 4.2 | 30.1 | 7.1 | 2.2 x 10^-3^ |
| 213603_s_at | RAC2 | ras-related C3 botulinum toxin substrate 2 (rho family, small GTP binding protein Rac2) | 6.9 | 50.2 | 7.3 | 1.8 x 10^-4^ |
| 212647_at | RRAS | related RAS viral (r-ras) oncogene homolog | 1.9 | 59.5 | 30.8 | 1.5 x 10^-7^ |
| 208456_s_at | RRAS2 | related RAS viral (r-ras) oncogene homolog 2 | 6.0 | 44.0 | 7.3 | 5.9 x 10^-6^ |
| 203085_s_at | TGFB1 | transforming growth factor, beta 1 | 2.6 | 14.9 | 5.7 | 1.2 x 10^-4^ |
| 209909_s_at | TGFB2 | transforming growth factor, beta 2 | 0.8 | 4.6 | 6.0 | 2.9 x 10^-4^ |
| 224793_s_at | TGFBR1 | transforming growth factor, beta receptor 1 | 7.3 | 54.4 | 7.5 | 7.4 x 10^-8^ |
| 225662_at | ZAK | sterile alpha motif and leucine zipper containing kinase AZK | 3.4 | 37.8 | 11.2 | 3.6 x 10^-7^ |
|  | |  |  |  |  |  |
| **Regulation of actin cytoskeleton** | | |  |  |  |  |
| 211160_x_at | ACTN1 | actinin, alpha 1 | 4.2 | 70.8 | 17.0 | 5.8 x 10^-3^ |
| 207988_s_at | ARPC2 | actin related protein 2/3 complex, subunit 2, 34kDa | 27.7 | 187.1 | 6.8 | 3.3 x 10^-8^ |
| 226915_s_at | ARPC5L | actin related protein 2/3 complex, subunit 5-like | 9.0 | 52.2 | 5.8 | 4.8 x 10^-10^ |
| 208727_s_at | CDC42 | cell division cycle 42 (GTP binding protein, 25kDa) | 19.3 | 106.1 | 5.5 | 1.7 x 10^-3^ |
| 202224_at | CRK | v-crk sarcoma virus CT10 oncogene homolog (avian) | 6.5 | 34.6 | 5.3 | 1.6 x 10^-9^ |
| 209190_s_at | DIAPH1 | diaphanous homolog 1 (Drosophila) | 8.2 | 47.4 | 5.8 | 4.2 x 10^-6^ |
| 201984_s_at | EGFR | epidermal growth factor receptor (erythroblastic leukemia viral (v-erb-b) oncogene homolog, avian) | 9.2 | 93.6 | 10.2 | 9.1 x 10^-7^ |
| 204819_at | FGD1 | FYV x , RhoG x F and PH domain containing 1 | 0.6 | 4.1 | 6.5 | 3.2 x 10^-5^ |
| 227271_at | FGF11 | fibroblast growth factor 11 | 1.3 | 8.3 | 6.2 | 2.5 x 10^-6^ |
| 204422_s_at | FGF2 | fibroblast growth factor 2 (basic) | 0.2 | 1.8 | 7.7 | 2.7 x 10^-3^ |
| 201474_s_at | ITGA3 | integrin, alpha 3 (antigen CD49C, alpha 3 subunit of VLA-3 receptor) | 3.2 | 50.1 | 15.8 | 5.0 x 10^-9^ |
| 201389_at | ITGA5 | integrin, alpha 5 (fibronectin receptor, alpha polypeptide) | 0.7 | 18.3 | 27.3 | 2.9 x 10^-6^ |
| 201656_at | ITGA6 | integrin, alpha 6 | 2.6 | 129.8 | 50.8 | 5.9 x 10^-9^ |
| 1553530_a_at | ITGB1 | integrin, beta 1 (fibronectin receptor, beta polypeptide, antigen CD29 includes MDF2, MSK12) | 16.9 | 216.8 | 12.8 | 7.4 x 10^-7^ |
| 204990_s_at | ITGB4 | integrin, beta 4 | 11.0 | 106.2 | 9.6 | 3.0 x 10^-7^ |
| 226535_at | ITGB6 | integrin, beta 6 | 5.4 | 44.6 | 8.3 | 6.3 x 10^-3^ |
| 202670_at | MAP2K1 | mitogen-activated protein kinase kinase 1 | 8.4 | 58.5 | 7.0 | 1.5 x 10^-9^ |
| 207121_s_at | MAPK6 | mitogen-activated protein kinase 6 | 27.7 | 163.3 | 5.9 | 3.8 x 10^-11^ |
| 200600_at | MSN | moesin | 8.3 | 87.2 | 10.5 | 2.6 x 10^-5^ |
| 207738_s_at | NCKAP1 | NCK-associated protein 1 | 26.8 | 152.6 | 5.7 | 1.4 x 10^-6^ |
| 202647_s_at | NRAS | neuroblastoma RAS viral (v-ras) oncogene homolog | 5.1 | 32.2 | 6.3 | 5.1 x 10^-6^ |
| 200634_at | PFN1 | profilin 1 | 33.0 | 240.8 | 7.3 | 5.1 x 10^-6^ |
| 213603_s_at | RAC2 | ras-related C3 botulinum toxin substrate 2 (rho family, small GTP binding protein Rac2) | 6.9 | 50.2 | 7.3 | 1.8 x 10^-4^ |
| 212647_at | RRAS | related RAS viral (r-ras) oncogene homolog | 1.9 | 59.5 | 30.8 | 1.5 x 10^-7^ |
| 208456_s_at | RRAS2 | related RAS viral (r-ras) oncogene homolog 2 | 6.0 | 44.0 | 7.3 | 5.9 x 10^-6^ |
| 209453_at | SLC9A1 | solute carrier family 9 (sodium/hydrogen exchanger), member 1 | 2.8 | 16.9 | 5.9 | 7.1 x 10^-5^ |
| 221753_at | SSH1 | slingshot homolog 1 (Drosophila) | 4.0 | 21.3 | 5.4 | 6.5 x 10^-9^ |
| 200931_s_at | VCL | vinculin | 9.6 | 55.8 | 5.8 | 1.5 x 10^-6^ |
| 204165_at | WASF1 | WAS protein family, member 1 | 0.6 | 12.1 | 20.5 | 2.4 x 10^-7^ |

1. For all significant KEGG pathways identified by GATHER analysis (Table 2) the genes overlapping with the basal cell signature were identified, and the basal and differentiated epithelium expression ratios were extracted.
2. p value following Benjamini-Hochberg correction.
