# Supplementary material for: The Human Airway Epithelial Basal Cell Transcriptome
Source: PLoS One. 2011 May 4;6(5):e18378. doi: 10.1371/journal.pone.0018378 (PMC3087716; doi:10.1371/journal.pone.0018378)
Supplement: Table S4 — All Significant Gene Ontology Categories of the Human Airway Basal Cell Signature. (DOC) [file pone.0018378.s004.doc]

| **GO Category** | **GO level** | **GO category title** | **Overlap with basal genes** | **Total genes** | **Percentage** | **p value** | **Bayes factor** | | **Genes** | | |
| --- | --- | --- | --- | --- | --- | --- | --- | --- | --- | --- | --- |
|  |  |  |  |  |  |  | |  | |  |  |
| GO:0007398 | 6 | ectoderm development | 22 | 218 | 10.1 | 7 x 10^-12^ | | 17.1 | | BNC1 COL7A1 CRABP2 DSP EMP1 FABP5 KLK5 KLK7 KRT16 KRT17 KRT5 KRT6A KRT6B LAMA3 LAMB3 LAMC2 PTHLH SCEL SPRR1A SPRR1B SPRR2B TFAP2A |  |
| GO:0008544 | 7 | epidermis development | 19 | 202 | 9.4 | 2 x 10^-9^ | | 13.8 | | BNC1 COL7A1 CRABP2 DSP EMP1 FABP5 KLK5 KLK7 KRT16 KRT17 KRT5 LAMA3 LAMB3 LAMC2 PTHLH SCEL SPRR1A SPRR1B SPRR2B |  |
| GO:0000074 | 6 | regulation of cell cycle | 52 | 448 | 11.6 | 4 x 10^-8^ | | 11.0 | | ALOX15B APBB2 AXL BCL10 CCNA1 CCND2 CCNE1 CDC25B CDC6 CDK6 CDK8 CDKN1A CDKN3 CHEK1 CUL4A DST DUSP6 E2F3 E2F7 EGFR EREG FGF2 G0S2 GADD45A HK2 IL1B KLK10 LATS2 MAD2L2 MCC MN1 MPHOSPH6 MYC NF2 NME1 NRAS PCTK1 PDGFA PGF PLK3 SESN2 SFN SHC1 TBRG4 TGFA TGFB1 TGFB2 UBE2V2 VEGF VEGFC ZAK ZMYND11 |  |
| GO:0009887 | 4 | organogenesis | 90 | 650 | 13.8 | 9 x 10^-7^ | | 8.1 | | ALOX15B ANGPTL4 APBB2 ARTN BMP1 BMP2 BNC1 CALD1 CDC42 CDH11 COL12A1 COL7A1 CRABP2 CSRP2 CUGBP2 CYP1B1 DBN1 DPYSL4 DSP EBP EMP1 EPHB2 EPHB4 EREG FABP5 FEZ1 FGD1 FGF11 FGF2 FHL1 FHL3 FLNA FLNB GJA1 HBEGF IFRD1 IL18 JAG1 JARID2 KLF6 KLK5 KLK6 KLK7 KLK8 KRT16 KRT17 KRT5 KRT6A KRT6B LAMA3 LAMB3 LAMC1 LAMC2 LMNA MATN3 MKKS NCKAP1 NPAS2 NRG1 NRP1 NTNG1 PDGFC PDLIM5 PGF PHGDH PTHLH ROBO3 SCEL SDCBP2 SEMA3B SERPINE2 SGCG SNAI2 SOX15 SPRR1A SPRR1B SPRR2B TEAD4 TFAP2A THBS1 TLE1 TNFRSF12A TNNT1 TPM1 TPM4 TPP1 TUFT1 TWIST2 VEGF VEGFC |  |
| GO:0009653 | 3 | morphogenesis | 106 | 1338 | 7.9 | 1 x 10^-6^ | | 7.9 | | ALOX15B ANGPTL4 APBB2 ARTN BMP1 BMP2 BNC1 CALD1 CAP1 CDC42 CDH11 COL12A1 COL7A1 CPM CRABP2 CSRP2 CUGBP2 CYP1B1 DBN1 DCBLD2 DKK3 DPYSL4 DSP EBP EMP1 EPHB2 EPHB4 EREG FABP5 FBN2 FEZ1 FGD1 FGF11 FGF2 FHL1 FHL3 FLNA FLNB FXYD5 GJA1 HBEGF IER3 IFRD1 IGFBP6 IL18 JAG1 JARID2 KLF6 KLK5 KLK6 KLK7 KLK8 KRT16 KRT17 KRT5 KRT6A KRT6B LAMA3 LAMB3 LAMC1 LAMC2 LMNA MATN3 MCAM MKKS NCKAP1 NPAS2 NRG1 NRP1 NTNG1 PDGFC PDLIM5 PGF PHGDH PTHLH ROBO3 SCEL SDCBP2 SEMA3B SERPINE2 SGCG SHC1 SLC3A2 SNAI2 SOCS2 SOX15 SPRR1A SPRR1B SPRR2B TEAD4 TFAP2A TGFB1 TGFB2 THBS1 TLE1 TNFRSF12A TNNT1 TPM1 TPM4 TPP1 TUFT1 TWIST2 VEGF VEGFC WHSC1 WNT7A |  |
| GO:0008283 | 4 | cell proliferation | 98 | 1169 | 8.4 | 3 x 10^-6^ | | 7.1 | | ADAMTS1 ALOX15B ANAPC11 APBB2 AREG ARTN AXL BCAT1 BCL10 BNC1 CCNA1 CCND2 CCNE1 CDC25B CDC6 CDK6 CDK8 CDKN1A CDKN3 CHEK1 CSRP2 CUL4A DNM2 DST DUSP6 E2F3 E2F7 EDN1 EGFR EMP1 EPHB4 EREG ETS1 FGF2 FOSL1 FSCN1 G0S2 GADD45A GRN HBEGF HDGFRP3 HK2 HMGA1 IFRD2 IGFBP6 IL18 IL1B JAG1 KLK10 KPNA2 KRT16 LAMP3 LATS2 MAD2L2 MAPK13 MAPK6 MCC MET MN1 MPHOSPH6 MXD1 MYC NAP1L1 NF2 NME1 NPM1 NRAS NRP1 OSMR PARD6G PAWR PCTK1 PDGFA PGF PLK3 PTHLH PTTG3 PYY RBM9 RIOK3 SC65 SESN2 SFN SHC1 SSR1 TACC1 TBRG4 TGFA TGFB1 TGFB1I1 TGFB2 TGFBI TIMP1 UBE2V2 VEGF VEGFC ZAK ZMYND11 |  |
| GO:0009113 | 7 | purine base biosynthesis | 4 | 8 | 50.0 | 1 x 10^-5^ | | 4.8 | | GART GMPS PAICS PPAT |  |
| GO:0030216 | 4 | keratinocyte differentiation | 5 | 75 | 6.7 | 3 x 10^-5^ | | 3.8 | | IVL JAG1 LAMA3 PTGS2 SPRR2B |  |
| GO:0006144 | 7 | purine base metabolism | 4 | 15 | 26.7 | 6 x 10^-5^ | | 3.3 | | GART GMPS PAICS PPAT |  |
| GO:0007049 | 5 | cell cycle | 66 | 1000 | 6.6 | 1 x 10^-4^ | | 3.2 | | ALOX15B ANAPC11 APBB2 AXL BCAT1 BCL10 CCNA1 CCND2 CCNE1 CDC25B CDC6 CDK6 CDK8 CDKN1A CDKN3 CHEK1 CUL4A DNM2 DST DUSP6 E2F3 E2F7 EGFR EREG FGF2 G0S2 GADD45A HK2 HMGA1 IL1B KLK10 KPNA2 LATS2 MAD2L2 MAPK13 MAPK6 MCC MN1 MPHOSPH6 MYC NAP1L1 NF2 NME1 NPM1 NRAS PARD6G PCTK1 PDGFA PGF PLK3 PTTG3 RIOK3 SC65 SESN2 SFN SHC1 TACC1 TBRG4 TGFA TGFB1 TGFB2 UBE2V2 VEGF VEGFC ZAK ZMYND11 |  |
| GO:0007250 | 6 | activation of NF-κB-inducing kinase | 5 | 13 | 38.5 | 1 x 10^-4^ | | 2.4 | | CARD10 IRAK1 MALT1 TNFRSF10A TNFRSF10B |  |
| GO:0046112 | 6 | nucleobase biosynthesis | 5 | 12 | 41.7 | 1 x 10^-4^ | | 2.4 | | CPS1 GART GMPS PAICS PPAT |  |
| GO:0006520 | 6 | amino acid metabolism | 27 | 232 | 11.6 | 3 x 10^-4^ | | 2.0 | | AK3 ARG2 ASNS BCAT1 CBS CPS1 CTH CTPS GARS GLS GMPS MARS NAGS NDST1 PHGDH PPAT PSAT1 PYCR1 SARS SDSL SHMT2 SLC25A15 SLC7A1 SLC7A5 TARS UST YARS |  |
| GO:0007010 | 6 | cytoskeleton organization and biogenesis | 35 | 534 | 6.6 | 4 x 10^-4^ | | 2.0 | | APBB2 ARPC2 ARPC5L CAP1 CAPG CDC42 CRK DBN1 DIAPH1 DST FGD1 FLNA FLNB FSCN1 KIF13A KIF1B KIF21B KPNA2 KRT6B KRT7 MAP2 MAP4 MYO5A NPM1 PFDN4 PFN1 PLEC1 PLEK2 PYY RHOF SGCG TRIP10 TUBB6 TUBGCP3 WASF1 |  |
| GO:0009064 | 7 | glutamine family amino acid metabolism | 9 | 51 | 17.6 | 3 x 10^-4^ | | 1.7 | | ARG2 ASNS CPS1 CTPS GLS GMPS NAGS PPAT PYCR1 |  |
| GO:0019752 | 6 | carboxylic acid metabolism | 41 | 615 | 6.7 | 6 x 10^-4^ | | 1.6 | | ACSL1 ACSL4 AK3 ALOX15B ALOXE3 ARG2 ASNS BCAT1 CBS CPS1 CTH CTPS ELOVL4 FADS1 FADS2 FADS3 GARS GLS GMPS MARS ME1 MTHFD1L MTHFD2 NAGS NDST1 PHGDH PPAT PSAT1 PTGS2 PYCR1 SARS SC4MOL SCD SDSL SHMT2 SLC25A15 SLC7A1 SLC7A5 TARS UST YARS |  |
| GO:0006082 | 5 | organic acid metabolism | 41 | 622 | 6.6 | 7 x 10^-4^ | | 1.5 | | ACSL1 ACSL4 AK3 ALOX15B ALOXE3 ARG2 ASNS BCAT1 CBS CPS1 CTH CTPS ELOVL4 FADS1 FADS2 FADS3 GARS GLS GMPS MARS ME1 MTHFD1L MTHFD2 NAGS NDST1 PHGDH PPAT PSAT1 PTGS2 PYCR1 SARS SC4MOL SCD SDSL SHMT2 SLC25A15 SLC7A1 SLC7A5 TARS UST YARS |  |
| GO:0009112 | 6 | nucleobase metabolism | 5 | 23 | 21.7 | 4 x 10^-4^ | | 1.4 | | CPS1 GART GMPS PAICS PPAT |  |
| GO:0009607 | 4 | response to biotic stimulus | 35 | 470 | 7.4 | 5 x 10^-4^ | | 1.4 | | ADA AOX1 C1QBP CAMP CAPG DEFB126 EDEM1 ETS1 FOSL1 GLRX2 GPX3 HRH1 HSPB8 IL18 IL1B IL1RN IL24 IRAK1 ITGB1 KLF6 MALT1 MICA MICB NFIL3 OXSR1 PABPC4 PROCR PTGS2 PVRL1 RIPK2 RNASE7 S100A9 SERPINH1 TICAM2 ULBP2 |  |
| GO:0007155 | 4 | cell adhesion | 53 | 824 | 6.4 | 9 x 10^-4^ | | 1.3 | | CD44 CDH11 CDH13 CDH3 CDH4 CEECAM1 CNTN1 COL12A1 COL4A6 COL7A1 CSPG4 DCBLD1 DCBLD2 DSC2 DSC3 DSG2 DSG3 DST FEZ1 FXYD5 HSPG2 IL18 ITGA3 ITGA5 ITGA6 ITGB1 ITGB4 ITGB6 JUP LAMA3 LAMB1 LAMB3 LAMC1 LAMC2 LOXL2 MCAM MICA MICB NRP1 PARVB PKP1 PKP2 PVRL1 SCARB1 SRPX TGFB1I1 TGFBI THBS1 TNC TNFRSF12A TPBG VCL ZYX |  |
| GO:0007154 | 3 | cell communication | 239 | 1675 | 14.3 | 2 x 10^-3^ | | 1.2 | | ADAM17 ADAM9 ADAMTS1 ADCY7 ADM ADRB2 AKAP12 AKT3 ANGPTL4 AP3S1 APBB2 AREG ARNTL2 ARTN AVPR1B AXL BCL10 BMP2 CAMKK2 CAP1 CARD10 CBL CCK CD44 CDC42 CDH11 CDH13 CDH3 CDH4 CEECAM1 CERK CNTN1 COL12A1 COL4A6 COL7A1 CORO1C CRABP2 CRK CSNK1E CSPG4 DCBLD1 DCBLD2 DKK1 DKK3 DLGAP4 DNM1L DNM2 DSC2 DSC3 DSG2 DSG3 DST DUSP6 ECM1 EDN1 EGFR EPHB2 EPHB4 EREG FADS1 FER FEZ1 FGD1 FGF11 FGF2 FLNA FLNB FST FXYD5 FYN GAL GDF15 GJA1 GJB2 GJB3 GJB6 GLRX2 GNA15 GNAI3 GNB1 GNG10 GOLT1B GPCR5A GPR115 GPR126 GPR153 GPR37 GPR87 GPSM1 GRB10 GRB14 GRN GTPBP4 HBEGF HIF1A HRH1 HSPG2 HTR7 IGF2R IGFBP6 IL18 IL1B IL1RL1 INPP4B INPP5D IRAK1 IRS1 ITGA3 ITGA5 ITGA6 ITGB1 ITGB4 ITGB6 ITPK1 JAG1 JUP KCNQ5 KIF1B KLK6 LAMA3 LAMB1 LAMB3 LAMC1 LAMC2 LATS2 LEPR LGALS1 LOXL2 LRP12 MALT1 MAP2K1 MAP4K4 MAPK13 MAPK6 MAPKAPK3 MCAM MCC MET MFHAS1 MICA MICB MKNK2 MYO10 NMU NPAS2 NRAS NRP1 NUDT11 OPN3 OSMR PARVB PDE5A PDGFA PDK1 PGF PITPNC1 PKP1 PKP2 PLAU PLAUR PLCD3 PLEK2 PPARG PPP2R5C PTHLH PTK6 PTPLA PTPN11 PTPRG PVRL1 PYY RAB27B RAB32 RAB38 RAB7L1 RAC2 RALA RAP2A RAP2B RAP2C RBM9 RGS10 RGS16 RGS20 RHEB RHOBTB2 RHOC RHOD RHOF RIPK2 RRAS RRAS2 RSU1 RTKN S100A9 SCARB1 SDCBP2 SEMA3B SFN SH3BP2 SHC1 SLC6A8 SNX24 SNX5 SOCS2 SPRY4 SQSTM1 SRP72 SRPX STC2 STK17A TAX1BP3 TFAP2A TGFA TGFB1 TGFB1I1 TGFBI TGFBR1 THBS1 TICAM2 TLE1 TMEPAI TNC TNFRSF10A TNFRSF10B TNFRSF12A TPBG TRIB3 TRIP10 UNC5B VCL VDR VEGF VEGFC WNT7A WSB2 YES1 ZAK ZYX |  |
| GO:0006519 | 5 | amino acid and derivative metabolism | 29 | 374 | 7.8 | 9 x 10^-4^ | | 1.1 | | AK3 ARG2 ASNS BCAT1 CBS CPS1 CTH CTPS GARS GLS GMPS MARS NAGS NDST1 ODC1 PHGDH PPAT PSAT1 PYCR1 SARS SDSL SHMT2 SLC25A15 SLC7A1 SLC7A5 SRM TARS UST YARS |  |
| GO:0006541 | 8 | glutamine metabolism | 6 | 17 | 35.3 | 6 x 10^-4^ | | 1.1 | | ASNS CTPS GLS GMPS PPAT PYCR1 |  |
| GO:0008652 | 7 | amino acid biosynthesis | 10 | 51 | 19.6 | 6 x 10^-4^ | | 1.1 | | AK3 ASNS BCAT1 CBS CPS1 CTH NAGS PHGDH PSAT1 PYCR1 |  |
| GO:0015849 | 5 | organic acid transport | 12 | 160 | 7.5 | 1 x 10^-3^ | | 0.7 | | SLC16A1 SLC16A2 SLC16A3 SLC16A4 SLC25A15 SLC38A1 SLC38A2 SLC38A5 SLC3A2 SLC7A1 SLC7A11 SLC7A5 |  |
| GO:0046942 | 6 | carboxylic acid transport | 12 | 159 | 7.5 | 1 x 10^-3^ | | 0.7 | | SLC16A1 SLC16A2 SLC16A3 SLC16A4 SLC25A15 SLC38A1 SLC38A2 SLC38A5 SLC3A2 SLC7A1 SLC7A11 SLC7A5 |  |
| GO:0009309 | 6 | amine biosynthesis | 12 | 86 | 14.0 | 1 x 10^-3^ | | 0.7 | | AK3 ASNS BCAT1 CBS CPS1 CTH NAGS ODC1 PHGDH PSAT1 PYCR1 SRM |  |
| GO:0007249 | 7 | I-κB kinase/NF-κB cascade | 15 | 173 | 8.7 | 2 x 10^-3^ | | 0.3 | | BCL10 CARD10 ECM1 FLNA GJA1 GOLT1B IRAK1 LGALS1 MALT1 RHOC RIPK2 SQSTM1 TICAM2 TNFRSF10A TNFRSF10B |  |
| GO:0000082 | 7 | G1/S transition of mitotic cell cycle | 10 | 52 | 19.2 | 2 x 10^-3^ | | 0.2 | | BCAT1 CCNA1 CCNE1 CDC6 CDKN1A CDKN3 CHEK1 CUL4A GADD45A LATS2 |  |

^1^ GATHER (Gene Annotation Tool to Help Explain Relationships, http://gather.genome.duke.edu/) was used to assist in functional annotation of the human basal signature. Input data consisted of the unique named genes from probe sets with basal / large airway epithelium expression ratio >5, p<0.0, following Benjamini-Hochberg multiple test correction. All significant overrepresented Gene Ontology categories are shown.

^2^ Number of genes in that category represented in the human airway basal cell signature.

^3^ A measure of false discovery rate.
