# Supplementary material for: The Human Airway Epithelial Basal Cell Transcriptome
Source: PLoS One. 2011 May 4;6(5):e18378. doi: 10.1371/journal.pone.0018378 (PMC3087716; doi:10.1371/journal.pone.0018378)
Supplement: Table S5 — Genes Comprising Top Gene Ontology Categories in the Human Basal Cell Signature. (DOC) [file pone.0018378.s005.doc]

| **ProbeSetID** | **Gene symbol** | **Gene title** | **Mean expression in differentiated epithelium** | **Mean expression in basal cells** | **Basal/differentiated epithelium expression ratio** | **p value^2^** |
| --- | --- | --- | --- | --- | --- | --- |
|  |  |  |  |  |  |  |
| **Ectoderm development** | | |  |  |  |  |
| 1552487_a_at | BNC1 | basonuclin 1 | 0.5 | 35.1 | 69.7 | 9.4 x 10^-5^ |
| 217312_s_at | COL7A1 | collagen, type VII, alpha 1 | 1.1 | 15.9 | 15.1 | 9.4 x 10^-6^ |
| 202575_at | CRABP2 | cellular retinoic acid binding protein 2 | 2.0 | 36.4 | 17.8 | 1.9 x 10^-4^ |
| 200606_at | DSP | desmoplakin | 69.6 | 359.2 | 5.2 | 9.1 x 10^-7^ |
| 201324_at | EMP1 | epithelial membrane protein 1 | 5.4 | 97.9 | 18.1 | 2.5 x 10^-5^ |
| 202345_s_at | FABP5 | fatty acid binding protein 5 (psoriasis-associated) | 16.0 | 90.4 | 5.7 | 1.5 x 10^-3^ |
| 222242_s_at | KLK5 | kallikrein-related peptidase 5 | 0.5 | 9.8 | 19.3 | 2.7 x 10^-4^ |
| 205778_at | KLK7 | kallikrein-related peptidase 7 | 0.1 | 24.2 | 221.3 | 3.6 x 10^-6^ |
| 209800_at | KRT16 | keratin 16 | 0.1 | 88.7 | 635.4 | 2.7 x 10^-8^ |
| 205157_s_at | KRT17 | keratin 17 | 10.7 | 537.9 | 50.3 | 1.9 x 10^-8^ |
| 201820_at | KRT5 | keratin 5 | 44.5 | 382.6 | 8.6 | 1.6 x 10^-5^ |
| 209125_at | KRT6A | keratin 6A | 1.1 | 724.4 | 667.2 | 3.2 x 10^-9^ |
| 209126_x_at | KRT6B | keratin 6B | 1.8 | 353.0 | 196.2 | 3.6 x 10^-10^ |
| 203726_s_at | LAMA3 | laminin, alpha 3 | 5.4 | 148.4 | 27.6 | 1.3 x 10^-5^ |
| 209270_at | LAMB3 | laminin, beta 3 | 15.9 | 297.3 | 18.7 | 2.8 x 10^-9^ |
| 202267_at | LAMC2 | laminin, gamma 2 | 11.8 | 417.2 | 35.2 | 1.6 x 10^-6^ |
| 210355_at | PTHLH | parathyroid hormone-like hormone | 0.6 | 18.6 | 32.3 | 2.8 x 10^-4^ |
| 206884_s_at | SCEL | sciellin | 0.4 | 73.3 | 194.6 | 2.0 x 10^-7^ |
| 213796_at | SPRR1A | small proline-rich protein 1A | 0.2 | 83.9 | 371.4 | 2.2 x 10^-6^ |
| 205064_at | SPRR1B | small proline-rich protein 1B (cornifin) | 0.7 | 234.3 | 345.5 | 5.4 x 10^-8^ |
| 208539_x_at | SPRR2B | small proline-rich protein 2B | 0.9 | 30.4 | 35.2 | 3.1 x 10^-4^ |
| 204653_at | TFAP2A | transcription factor AP-2 alpha (activating enhancer binding protein 2 alpha) | 9.9 | 64.2 | 6.5 | 2.8 x 10^-9^ |
| **Epidermis development** | | |  |  |  |  |
| 1552487_a_at | BNC1 | basonuclin 1 | 0.5 | 35.1 | 69.7 | 9.4 x 10^-5^ |
| 217312_s_at | COL7A1 | collagen, type VII, alpha 1 | 1.1 | 15.9 | 15.1 | 9.4 x 10^-6^ |
| 202575_at | CRABP2 | cellular retinoic acid binding protein 2 | 2.0 | 36.4 | 17.8 | 1.9 x 10^-4^ |
| 200606_at | DSP | desmoplakin | 69.6 | 359.2 | 5.2 | 9.1 x 10^-7^ |
| 201324_at | EMP1 | epithelial membrane protein 1 | 5.4 | 97.9 | 18.1 | 2.5 x 10^-5^ |
| 202345_s_at | FABP5 | fatty acid binding protein 5 (psoriasis-associated) | 16.0 | 90.4 | 5.7 | 1.5 x 10^-3^ |
| 222242_s_at | KLK5 | kallikrein-related peptidase 5 | 0.5 | 9.8 | 19.3 | 2.7 x 10^-4^ |
| 205778_at | KLK7 | kallikrein-related peptidase 7 | 0.1 | 24.2 | 221.3 | 3.6 x 10^-6^ |
| 209800_at | KRT16 | keratin 16 | 0.1 | 88.7 | 635.4 | 2.7 x 10^-8^ |
| 205157_s_at | KRT17 | keratin 17 | 10.7 | 537.9 | 50.3 | 1.9 x 10^-8^ |
| 201820_at | KRT5 | keratin 5 | 44.5 | 382.6 | 8.6 | 1.6 x 10^-5^ |
| 203726_s_at | LAMA3 | laminin, alpha 3 | 5.4 | 148.4 | 27.6 | 1.3 x 10^-5^ |
| 209270_at | LAMB3 | laminin, beta 3 | 15.9 | 297.3 | 18.7 | 2.8 x 10^-9^ |
| 202267_at | LAMC2 | laminin, gamma 2 | 11.8 | 417.2 | 35.2 | 1.6 x 10^-6^ |
| 210355_at | PTHLH | parathyroid hormone-like hormone | 0.6 | 18.6 | 32.3 | 2.8 x 10^-4^ |
| 206884_s_at | SCEL | sciellin | 0.4 | 73.3 | 194.6 | 2.0 x 10^-7^ |
| 213796_at | SPRR1A | small proline-rich protein 1A | 0.2 | 83.9 | 371.4 | 2.2 x 10^-6^ |
| 205064_at | SPRR1B | small proline-rich protein 1B (cornifin) | 0.7 | 234.3 | 345.5 | 5.4 x 10^-8^ |
| 208539_x_at | SPRR2B | small proline-rich protein 2B | 0.9 | 30.4 | 35.2 | 3.1 x 10^-4^ |
| **Regulation of cell cycle** | | |  |  |  |  |
| 206714_at | ALOX15B | arachidonate 15-lipoxygenase, type B | 0.4 | 2.8 | 6.8 | 2.2 x 10^-3^ |
| 213419_at | APBB2 | amyloid beta (A4) precursor protein-binding, family B, member 2 | 0.9 | 8.0 | 8.8 | 5.9 x 10^-7^ |
| 202686_s_at | AXL | AXL receptor tyrosine kinase | 5.7 | 34.6 | 6.0 | 4.5 x 10^-5^ |
| 1557257_at | BCL10 | B-cell CLL/lymphoma 10 | 0.8 | 6.5 | 8.4 | 3.0 x 10^-3^ |
| 205899_at | CCNA1 | cyclin A1 | 4.4 | 42.8 | 9.7 | 6.2 x 10^-3^ |
| 200951_s_at | CCND2 | cyclin D2 | 0.5 | 12.4 | 26.2 | 2.5 x 10^-7^ |
| 213523_at | CCNE1 | cyclin E1 | 0.7 | 3.7 | 5.1 | 1.9 x 10^-8^ |
| 201853_s_at | CDC25B | cell division cycle 25 homolog B (S. pombe) | 2.3 | 21.6 | 9.3 | 5.2 x 10^-6^ |
| 203967_at | CDC6 | cell division cycle 6 homolog (S. cerevisiae) | 0.2 | 4.4 | 18.8 | 7.8 x 10^-3^ |
| 224851_at | CDK6 | cyclin-dependent kinase 6 | 7.8 | 53.2 | 6.8 | 1.1 x 10^-6^ |
| 1553113_s_at | CDK8 | cyclin-dependent kinase 8 | 2.5 | 15.7 | 6.2 | 4.6 x 10^-7^ |
| 202284_s_at | CDKN1A | cyclin-dependent kinase inhibitor 1A (p21, Cip1) | 10.7 | 167.8 | 15.7 | 2.0 x 10^-6^ |
| 1555758_a_at | CDKN3 | cyclin-dependent kinase inhibitor 3 | 0.4 | 17.0 | 39.7 | 4.8 x 10^-3^ |
| 205393_s_at | CHEK1 | CHK1 checkpoint homolog (S. pombe) | 0.5 | 4.3 | 8.5 | 8.8 x 10^-4^ |
| 227757_at | CUL4A | cullin 4A | 0.6 | 2.9 | 5.2 | 6.1 x 10^-4^ |
| 216918_s_at | DST | dystonin | 3.3 | 99.3 | 30.2 | 2.0 x 10^-8^ |
| 208892_s_at | DUSP6 | dual specificity phosphatase 6 | 13.6 | 87.4 | 6.4 | 3.0 x 10^-7^ |
| 203693_s_at | E2F3 | E2F transcription factor 3 | 1.0 | 8.5 | 8.1 | 4.6 x 10^-10^ |
| 228033_at | E2F7 | E2F transcription factor 7 | 2.5 | 20.7 | 8.2 | 1.2 x 10^-4^ |
| 201984_s_at | EGFR | epidermal growth factor receptor (erythroblastic leukemia viral (v-erb-b) oncogene homolog, avian) | 9.2 | 93.6 | 10.2 | 9.1 x 10^-7^ |
| 205767_at | EREG | epiregulin | 0.1 | 34.6 | 246.0 | 8.0 x 10^-7^ |
| 204422_s_at | FGF2 | fibroblast growth factor 2 (basic) | 0.2 | 1.8 | 7.7 | 2.7 x 10^-3^ |
| 213524_s_at | G0S2 | G0/G1switch 2 | 3.5 | 99.0 | 28.1 | 2.0 x 10^-4^ |
| 203725_at | GADD45A | growth arrest and DNA-damage-inducible, alpha | 13.3 | 135.4 | 10.2 | 4.4 x 10^-10^ |
| 202934_at | HK2 | hexokinase 2 | 4.0 | 41.4 | 10.3 | 2.9 x 10^-5^ |
| 205067_at | IL1B | interleukin 1, beta | 1.8 | 20.9 | 11.7 | 6.3 x 10^-3^ |
| 209792_s_at | KLK10 | kallikrein-related peptidase 10 | 9.2 | 77.4 | 8.4 | 3.8 x 10^-5^ |
| 230348_at | LATS2 | LATS, large tumor suppressor, homolog 2 (Drosophila) | 0.2 | 1.5 | 8.9 | 3.3 x 10^-4^ |
| 223234_at | MAD2L2 | MAD2 mitotic arrest deficient-like 2 (yeast) | 2.4 | 16.5 | 6.8 | 4.5 x 10^-9^ |
| 226225_at | MCC | mutated in colorectal cancers | 3.9 | 24.6 | 6.3 | 8.8 x 10^-6^ |
| 205330_at | MN1 | meningioma (disrupted in balanced translocation) 1 | 0.5 | 5.6 | 12.2 | 8.6 x 10^-8^ |
| 203740_at | MPHOSPH6 | M-phase phosphoprotein 6 | 10.2 | 63.9 | 6.3 | 2.4 x 10^-9^ |
| 202431_s_at | MYC | v-myc myelocytomatosis viral oncogene homolog (avian) | 9.6 | 90.1 | 9.4 | 8.1 x 10^-5^ |
| 217150_s_at | NF2 | neurofibromin 2 (merlin) | 0.8 | 4.2 | 5.3 | 7.4 x 10^-5^ |
| 201577_at | NME1 | non-metastatic cells 1, protein (NM23A) expressed in | 11.2 | 56.8 | 5.1 | 2.1 x 10^-4^ |
| 202647_s_at | NRAS | neuroblastoma RAS viral (v-ras) oncogene homolog | 5.1 | 32.2 | 6.3 | 5.1 x 10^-6^ |
| 208824_x_at | PCTK1 | PCTAIRE protein kinase 1 | 4.0 | 20.1 | 5.0 | 6.6 x 10^-5^ |
| 205463_s_at | PDGFA | platelet-derived growth factor alpha polypeptide | 3.0 | 19.4 | 6.4 | 5.8 x 10^-6^ |
| 209652_s_at | PGF | placental growth factor | 0.4 | 7.3 | 17.9 | 7.2 x 10^-7^ |
| 204958_at | PLK3 | polo-like kinase 3 (Drosophila) | 0.9 | 4.7 | 5.2 | 1.5 x 10^-7^ |
| 223195_s_at | SESN2 | sestrin 2 | 2.3 | 29.8 | 12.7 | 1.1 x 10^-4^ |
| 209260_at | SFN | stratifin | 4.0 | 141.0 | 35.4 | 2.2 x 10^-4^ |
| 214853_s_at | SHC1 | SHC (Src homology 2 domain containing) transforming protein 1 | 14.9 | 204.0 | 13.7 | 3.3 x 10^-8^ |
| 220789_s_at | TBRG4 | transforming growth factor beta regulator 4 | 1.3 | 10.5 | 7.9 | 9.0 x 10^-5^ |
| 205016_at | TGFA | transforming growth factor, alpha | 7.4 | 59.7 | 8.1 | 4.5 x 10^-11^ |
| 203085_s_at | TGFB1 | transforming growth factor, beta 1 | 2.6 | 14.9 | 5.7 | 1.2 x 10^-4^ |
| 209909_s_at | TGFB2 | transforming growth factor, beta 2 | 0.8 | 4.6 | 6.0 | 2.9 x 10^-4^ |
| 209096_at | UBE2V2 | ubiquitin-conjugating enzyme E2 variant 2 | 13.0 | 65.6 | 5.0 | 3.9 x 10^-6^ |
| 211527_x_at | VEGFA | vascular endothelial growth factor A | 4.2 | 35.1 | 8.3 | 1.6 x 10^-5^ |
| 209946_at | VEGFC | vascular endothelial growth factor C | 1.3 | 9.1 | 7.2 | 2.2 x 10^-5^ |
| 225662_at | ZAK | sterile alpha motif and leucine zipper containing kinase AZK | 3.4 | 37.8 | 11.2 | 3.6 x 10^-7^ |
| 1554158_at | ZMYND11 | zinc finger, MYND domain containing 11 | 0.2 | 0.9 | 5.1 | 9.0 x 10^-3^ |
| **Organogenesis** | | |  |  |  |  |
| 206714_at | ALOX15B | arachidonate 15-lipoxygenase, type B | 0.4 | 2.8 | 6.8 | 2.2 x 10^-3^ |
| 221009_s_at | ANGPTL4 | angiopoietin-like 4 | 0.8 | 4.6 | 5.8 | 5.2 x 10^-3^ |
| 213419_at | APBB2 | amyloid beta (A4) precursor protein-binding, family B, member 2 | 0.9 | 8.0 | 8.8 | 5.9 x 10^-7^ |
| 210237_at | ARTN | artemin | 0.4 | 9.0 | 21.7 | 3.4 x 10^-4^ |
| 205574_x_at | BMP1 | bone morphogenetic protein 1 | 0.4 | 5.2 | 12.3 | 1.7 x 10^-5^ |
| 205289_at | BMP2 | bone morphogenetic protein 2 | 0.3 | 8.2 | 30.7 | 1.2 x 10^-9^ |
| 1552487_a_at | BNC1 | basonuclin 1 | 0.5 | 35.1 | 69.7 | 9.4 x 10^-5^ |
| 212077_at | CALD1 | caldesmon 1 | 1.2 | 135.8 | 109.6 | 1.2 x 10^-6^ |
| 208727_s_at | CDC42 | cell division cycle 42 (GTP binding protein, 25kDa) | 19.3 | 106.1 | 5.5 | 1.7 x 10^-3^ |
| 207173_x_at | CDH11 | cadherin 11, type 2, OB-cadherin (osteoblast) | 2.7 | 23.1 | 8.5 | 1.8 x 10^-5^ |
| 231766_s_at | COL12A1 | collagen, type XII, alpha 1 | 0.4 | 4.0 | 9.7 | 2.9 x 10^-4^ |
| 217312_s_at | COL7A1 | collagen, type VII, alpha 1 | 1.1 | 15.9 | 15.1 | 9.4 x 10^-6^ |
| 202575_at | CRABP2 | cellular retinoic acid binding protein 2 | 2.0 | 36.4 | 17.8 | 1.9 x 10^-4^ |
| 207030_s_at | CSRP2 | cysteine and glycine-rich protein 2 | 2.3 | 51.6 | 22.1 | 7.4 x 10^-7^ |
| 202158_s_at | CUGBP2 | CUG triplet repeat, RNA binding protein 2 | 1.9 | 9.8 | 5.0 | 5.2 x 10^-4^ |
| 202437_s_at | CYP1B1 | cytochrome P450, family 1, subfamily B, polypeptide 1 | 0.7 | 20.4 | 27.4 | 5.9 x 10^-3^ |
| 202806_at | DBN1 | drebrin 1 | 1.5 | 19.1 | 12.4 | 5.9 x 10^-8^ |
| 205493_s_at | DPYSL4 | dihydropyrimidinase-like 4 | 0.2 | 1.8 | 9.0 | 6.6 x 10^-3^ |
| 200606_at | DSP | desmoplakin | 69.6 | 359.2 | 5.2 | 9.1 x 10^-7^ |
| 202735_at | EBP | emopamil binding protein (sterol isomerase) | 5.1 | 27.8 | 5.5 | 4.6 x 10^-5^ |
| 201324_at | EMP1 | epithelial membrane protein 1 | 5.4 | 97.9 | 18.1 | 2.5 x 10^-5^ |
| 209589_s_at | EPHB2 | EPH receptor B2 | 0.8 | 6.0 | 7.7 | 2.5 x 10^-4^ |
| 202894_at | EPHB4 | EPH receptor B4 | 3.2 | 19.1 | 6.0 | 9.8 x 10^-8^ |
| 205767_at | EREG | epiregulin | 0.1 | 34.6 | 246.0 | 8.0 x 10^-7^ |
| 202345_s_at | FABP5 | fatty acid binding protein 5 (psoriasis-associated) | 16.0 | 90.4 | 5.7 | 1.5 x 10^-3^ |
| 203562_at | FEZ1 | fasciculation and elongation protein zeta 1 (zygin I) | 1.3 | 53.4 | 42.2 | 2.2 x 10^-6^ |
| 204819_at | FGD1 | FYVE, RhoGEF and PH domain containing 1 | 0.6 | 4.1 | 6.5 | 3.2 x 10^-5^ |
| 227271_at | FGF11 | fibroblast growth factor 11 | 1.3 | 8.3 | 6.2 | 2.5 x 10^-6^ |
| 204422_s_at | FGF2 | fibroblast growth factor 2 (basic) | 0.2 | 1.8 | 7.7 | 2.7 x 10^-3^ |
| 201540_at | FHL1 | four and a half LIM domains 1 | 5.1 | 55.9 | 10.9 | 1.9 x 10^-4^ |
| 218818_at | FHL3 | four and a half LIM domains 3 | 0.6 | 4.1 | 7.0 | 7.2 x 10^-4^ |
| 213746_s_at | FLNA | filamin A, alpha (actin binding protein 280) | 2.0 | 38.0 | 19.1 | 2.1 x 10^-5^ |
| 208613_s_at | FLNB | filamin B, beta (actin binding protein 278) | 13.4 | 103.5 | 7.7 | 6.9 x 10^-6^ |
| 201667_at | GJA1 | gap junction protein, alpha 1, 43kDa | 2.6 | 83.8 | 32.9 | 5.2 x 10^-4^ |
| 38037_at | HBEGF | heparin-binding EGF-like growth factor | 3.8 | 65.4 | 17.3 | 6.0 x 10^-7^ |
| 202147_s_at | IFRD1 | interferon-related developmental regulator 1 | 3.9 | 28.4 | 7.3 | 2.9 x 10^-4^ |
| 206295_at | IL18 | interleukin 18 (interferon-gamma-inducing factor) | 4.0 | 26.5 | 6.6 | 5.2 x 10^-9^ |
| 216268_s_at | JAG1 | jagged 1 (Alagille syndrome) | 23.3 | 249.3 | 10.7 | 4.0 x 10^-7^ |
| 203298_s_at | JARID2 | jumonji, AT rich interactive domain 2 | 2.7 | 19.2 | 7.1 | 1.4 x 10^-7^ |
| 208961_s_at | KLF6 | Kruppel-like factor 6 | 11.4 | 62.0 | 5.5 | 7.5 x 10^-5^ |
| 222242_s_at | KLK5 | kallikrein-related peptidase 5 | 0.5 | 9.8 | 19.3 | 2.7 x 10^-4^ |
| 204733_at | KLK6 | kallikrein-related peptidase 6 | 0.4 | 16.2 | 38.1 | 8.2 x 10^-5^ |
| 205778_at | KLK7 | kallikrein-related peptidase 7 | 0.1 | 24.2 | 221.3 | 3.6 x 10^-6^ |
| 206125_s_at | KLK8 | kallikrein-related peptidase 8 | 0.7 | 8.4 | 11.9 | 2.8 x 10^-6^ |
| 209800_at | KRT16 | keratin 16 | 0.1 | 88.7 | 635.4 | 2.7 x 10^-8^ |
| 205157_s_at | KRT17 | keratin 17 | 10.7 | 537.9 | 50.3 | 1.9 x 10^-8^ |
| 201820_at | KRT5 | keratin 5 | 44.5 | 382.6 | 8.6 | 1.6 x 10^-5^ |
| 209125_at | KRT6A | keratin 6A | 1.1 | 724.4 | 667.2 | 3.2 x 10^-9^ |
| 209126_x_at | KRT6B | keratin 6B | 1.8 | 353.0 | 196.2 | 3.6 x 10^-10^ |
| 203726_s_at | LAMA3 | laminin, alpha 3 | 5.4 | 148.4 | 27.6 | 1.3 x 10^-5^ |
| 209270_at | LAMB3 | laminin, beta 3 | 15.9 | 297.3 | 18.7 | 2.8 x 10^-9^ |
| 200770_s_at | LAMC1 | laminin, gamma 1 (formerly LAMB2) | 3.5 | 45.0 | 12.9 | 5.5 x 10^-8^ |
| 202267_at | LAMC2 | laminin, gamma 2 | 11.8 | 417.2 | 35.2 | 1.6 x 10^-6^ |
| 212086_x_at | LMNA | lamin A/C | 7.5 | 45.8 | 6.1 | 3.4 x 10^-6^ |
| 206091_at | MATN3 | matrilin 3 | 0.2 | 1.0 | 6.2 | 3.5 x 10^-6^ |
| 222530_s_at | MKKS | McKusick-Kaufman syndrome | 6.7 | 41.1 | 6.2 | 1.2 x 10^-6^ |
| 207738_s_at | NCKAP1 | NCK-associated protein 1 | 26.8 | 152.6 | 5.7 | 1.4 x 10^-6^ |
| 205460_at | NPAS2 | neuronal PAS domain protein 2 | 0.4 | 4.7 | 10.7 | 1.2 x 10^-3^ |
| 206343_s_at | NRG1 | neuregulin 1 | 0.4 | 19.8 | 53.4 | 7.4 x 10^-7^ |
| 210510_s_at | NRP1 | neuropilin 1 | 0.2 | 2.7 | 13.0 | 2.4 x 10^-7^ |
| 236088_at | NTNG1 | netrin G1 | 0.4 | 2.3 | 6.4 | 8.3 x 10^-5^ |
| 218718_at | PDGFC | platelet derived growth factor C | 12.7 | 78.6 | 6.2 | 2.6 x 10^-7^ |
| 221994_at | PDLIM5 | PDZ and LIM domain 5 | 0.4 | 1.9 | 5.0 | 2.9 x 10^-3^ |
| 209652_s_at | PGF | placental growth factor | 0.4 | 7.3 | 17.9 | 7.2 x 10^-7^ |
| 201397_at | PHGDH | phosphoglycerate dehydrogenase | 4.1 | 33.4 | 8.2 | 2.3 x 10^-9^ |
| 210355_at | PTHLH | parathyroid hormone-like hormone | 0.6 | 18.6 | 32.3 | 2.8 x 10^-4^ |
| 219550_at | ROBO3 | roundabout, axon guidance receptor, homolog 3 (Drosophila) | 0.6 | 4.2 | 7.4 | 9.4 x 10^-6^ |
| 206884_s_at | SCEL | sciellin | 0.4 | 73.3 | 194.6 | 2.0 x 10^-7^ |
| 233565_s_at | SDCBP2 | syndecan binding protein (syntenin) 2 | 3.8 | 24.0 | 6.3 | 8.6 x 10^-6^ |
| 203071_at | SEMA3B | sema domain, immunoglobulin domain (Ig), short basic domain, secreted, (semaphorin) 3B | 0.3 | 3.6 | 11.8 | 2.3 x 10^-5^ |
| 212190_at | SERPINE2 | serpin peptidase inhibitor, clade E (nexin, plasminogen activator inhibitor type 1), member 2 | 0.9 | 85.0 | 91.4 | 1.9 x 10^-9^ |
| 207302_at | SGCG | sarcoglycan, gamma (35kDa dystrophin-associated glycoprotein) | 0.1 | 0.7 | 10.5 | 2.3 x 10^-4^ |
| 213139_at | SNAI2 | snail homolog 2 (Drosophila) | 6.0 | 71.9 | 12.0 | 2.0 x 10^-4^ |
| 206122_at | SOX15 | SRY (sex determining region Y)-box 15 | 1.9 | 27.0 | 14.0 | 2.9 x 10^-8^ |
| 213796_at | SPRR1A | small proline-rich protein 1A | 0.2 | 83.9 | 371.4 | 2.2 x 10^-6^ |
| 205064_at | SPRR1B | small proline-rich protein 1B (cornifin) | 0.7 | 234.3 | 345.5 | 5.4 x 10^-8^ |
| 208539_x_at | SPRR2B | small proline-rich protein 2B | 0.9 | 30.4 | 35.2 | 3.1 x 10^-4^ |
| 41037_at | TEAD4 | TEA domain family member 4 | 0.4 | 2.7 | 6.0 | 4.3 x 10^-7^ |
| 204653_at | TFAP2A | transcription factor AP-2 alpha (activating enhancer binding protein 2 alpha) | 9.9 | 64.2 | 6.5 | 2.8 x 10^-9^ |
| 201109_s_at | THBS1 | thrombospondin 1 | 0.5 | 59.4 | 110.4 | 7.3 x 10^-3^ |
| 228284_at | TLE1 | transducin-like enhancer of split 1 (E(sp1) homolog, Drosophila) | 1.7 | 18.4 | 11.0 | 2.0 x 10^-11^ |
| 218368_s_at | TNFRSF12A | tumor necrosis factor receptor superfamily, member 12A | 2.7 | 69.4 | 25.5 | 1.3 x 10^-5^ |
| 213201_s_at | TNNT1 | troponin T type 1 (skeletal, slow) | 0.5 | 4.5 | 9.9 | 1.3 x 10^-4^ |
| 210986_s_at | TPM1 | tropomyosin 1 (alpha) | 16.5 | 90.2 | 5.4 | 8.9 x 10^-6^ |
| 1567107_s_at | TPM4 | tropomyosin 4 | 1.1 | 24.3 | 22.7 | 1.1 x 10^-6^ |
| 200743_s_at | TPP1 | tripeptidyl peptidase I | 10.2 | 52.2 | 5.1 | 7.3 x 10^-6^ |
| 205807_s_at | TUFT1 | tuftelin 1 | 8.1 | 41.0 | 5.1 | 5.4 x 10^-7^ |
| 229404_at | TWIST2 | twist homolog 2 (Drosophila) | 0.5 | 3.7 | 7.7 | 4.0 x 10^-3^ |
| 211527_x_at | VEGFA | vascular endothelial growth factor A | 4.2 | 35.1 | 8.3 | 1.6 x 10^-5^ |
| 209946_at | VEGFC | vascular endothelial growth factor C | 1.3 | 9.1 | 7.2 | 2.2 x 10^-5^ |
| **Morphogenesis** | | |  |  |  |  |
| 206714_at | ALOX15B | arachidonate 15-lipoxygenase, type B | 0.4 | 2.8 | 6.8 | 2.2 x 10^-3^ |
| 221009_s_at | ANGPTL4 | angiopoietin-like 4 | 0.8 | 4.6 | 5.8 | 5.2 x 10^-3^ |
| 213419_at | APBB2 | amyloid beta (A4) precursor protein-binding, family B, member 2 | 0.9 | 8.0 | 8.8 | 5.9 x 10^-7^ |
| 210237_at | ARTN | artemin | 0.4 | 9.0 | 21.7 | 3.4 x 10^-4^ |
| 205574_x_at | BMP1 | bone morphogenetic protein 1 | 0.4 | 5.2 | 12.3 | 1.7 x 10^-5^ |
| 205289_at | BMP2 | bone morphogenetic protein 2 | 0.3 | 8.2 | 30.7 | 1.2 x 10^-9^ |
| 1552487_a_at | BNC1 | basonuclin 1 | 0.5 | 35.1 | 69.7 | 9.4 x 10^-5^ |
| 212077_at | CALD1 | caldesmon 1 | 1.2 | 135.8 | 109.6 | 1.2 x 10^-6^ |
| 213798_s_at | CAP1 | CAP, adenylate cyclase-associated protein 1 (yeast) | 28.0 | 174.5 | 6.2 | 3.8 x 10^-9^ |
| 208727_s_at | CDC42 | cell division cycle 42 (GTP binding protein, 25kDa) | 19.3 | 106.1 | 5.5 | 1.7 x 10^-3^ |
| 207173_x_at | CDH11 | cadherin 11, type 2, OB-cadherin (osteoblast) | 2.7 | 23.1 | 8.5 | 1.8 x 10^-5^ |
| 231766_s_at | COL12A1 | collagen, type XII, alpha 1 | 0.4 | 4.0 | 9.7 | 2.9 x 10^-4^ |
| 217312_s_at | COL7A1 | collagen, type VII, alpha 1 | 1.1 | 15.9 | 15.1 | 9.4 x 10^-6^ |
| 206100_at | CPM | carboxypeptidase M | 0.4 | 12.2 | 28.8 | 1.2 x 10^-4^ |
| 202575_at | CRABP2 | cellular retinoic acid binding protein 2 | 2.0 | 36.4 | 17.8 | 1.9 x 10^-4^ |
| 207030_s_at | CSRP2 | cysteine and glycine-rich protein 2 | 2.3 | 51.6 | 22.1 | 7.4 x 10^-7^ |
| 202158_s_at | CUGBP2 | CUG triplet repeat, RNA binding protein 2 | 1.9 | 9.8 | 5.0 | 5.2 x 10^-4^ |
| 202437_s_at | CYP1B1 | cytochrome P450, family 1, subfamily B, polypeptide 1 | 0.7 | 20.4 | 27.4 | 5.9 x 10^-3^ |
| 202806_at | DBN1 | drebrin 1 | 1.5 | 19.1 | 12.4 | 5.9 x 10^-8^ |
| 213865_at | DCBLD2 | discoidin, CUB and LCCL domain containing 2 | 0.4 | 5.6 | 14.5 | 3.9 x 10^-6^ |
| 230508_at | DKK3 | dickkopf homolog 3 (Xenopus laevis) | 0.3 | 7.3 | 27.9 | 2.5 x 10^-6^ |
| 205493_s_at | DPYSL4 | dihydropyrimidinase-like 4 | 0.2 | 1.8 | 9.0 | 6.6 x 10^-3^ |
| 200606_at | DSP | desmoplakin | 69.6 | 359.2 | 5.2 | 9.1 x 10^-7^ |
| 202735_at | EBP | emopamil binding protein (sterol isomerase) | 5.1 | 27.8 | 5.5 | 4.6 x 10^-5^ |
| 201324_at | EMP1 | epithelial membrane protein 1 | 5.4 | 97.9 | 18.1 | 2.5 x 10^-5^ |
| 209589_s_at | EPHB2 | EPH receptor B2 | 0.8 | 6.0 | 7.7 | 2.5 x 10^-4^ |
| 202894_at | EPHB4 | EPH receptor B4 | 3.2 | 19.1 | 6.0 | 9.8 x 10^-8^ |
| 205767_at | EREG | epiregulin | 0.1 | 34.6 | 246.0 | 8.0 x 10^-7^ |
| 202345_s_at | FABP5 | fatty acid binding protein 5 (psoriasis-associated) | 16.0 | 90.4 | 5.7 | 1.5 x 10^-3^ |
| 203184_at | FBN2 | fibrillin 2 | 1.7 | 10.2 | 5.9 | 4.5 x 10^-3^ |
| 203562_at | FEZ1 | fasciculation and elongation protein zeta 1 (zygin I) | 1.3 | 53.4 | 42.2 | 2.2 x 10^-6^ |
| 204819_at | FGD1 | FYVE, RhoGEF and PH domain containing 1 | 0.6 | 4.1 | 6.5 | 3.2 x 10^-5^ |
| 227271_at | FGF11 | fibroblast growth factor 11 | 1.3 | 8.3 | 6.2 | 2.5 x 10^-6^ |
| 204422_s_at | FGF2 | fibroblast growth factor 2 (basic) | 0.2 | 1.8 | 7.7 | 2.7 x 10^-3^ |
| 201540_at | FHL1 | four and a half LIM domains 1 | 5.1 | 55.9 | 10.9 | 1.9 x 10^-4^ |
| 218818_at | FHL3 | four and a half LIM domains 3 | 0.6 | 4.1 | 7.0 | 7.2 x 10^-4^ |
| 213746_s_at | FLNA | filamin A, alpha (actin binding protein 280) | 2.0 | 38.0 | 19.1 | 2.1 x 10^-5^ |
| 208613_s_at | FLNB | filamin B, beta (actin binding protein 278) | 13.4 | 103.5 | 7.7 | 6.9 x 10^-6^ |
| 218084_x_at | FXYD5 | FXYD domain containing ion transport regulator 5 | 3.8 | 45.6 | 12.0 | 6.5 x 10^-8^ |
| 201667_at | GJA1 | gap junction protein, alpha 1, 43kDa | 2.6 | 83.8 | 32.9 | 5.2 x 10^-4^ |
| 38037_at | HBEGF | heparin-binding EGF-like growth factor | 3.8 | 65.4 | 17.3 | 6.0 x 10^-7^ |
| 201631_s_at | IER3 | immediate early response 3 | 43.6 | 424.8 | 9.8 | 6.3 x 10^-9^ |
| 202147_s_at | IFRD1 | interferon-related developmental regulator 1 | 3.9 | 28.4 | 7.3 | 2.9 x 10^-4^ |
| 203851_at | IGFBP6 | insulin-like growth factor binding protein 6 | 1.6 | 13.8 | 8.5 | 1.7 x 10^-4^ |
| 206295_at | IL18 | interleukin 18 (interferon-gamma-inducing factor) | 4.0 | 26.5 | 6.6 | 5.2 x 10^-9^ |
| 216268_s_at | JAG1 | jagged 1 (Alagille syndrome) | 23.3 | 249.3 | 10.7 | 4.0 x 10^-7^ |
| 203298_s_at | JARID2 | jumonji, AT rich interactive domain 2 | 2.7 | 19.2 | 7.1 | 1.4 x 10^-7^ |
| 208961_s_at | KLF6 | Kruppel-like factor 6 | 11.4 | 62.0 | 5.5 | 7.5 x 10^-5^ |
| 222242_s_at | KLK5 | kallikrein-related peptidase 5 | 0.5 | 9.8 | 19.3 | 2.7 x 10^-4^ |
| 204733_at | KLK6 | kallikrein-related peptidase 6 | 0.4 | 16.2 | 38.1 | 8.2 x 10^-5^ |
| 205778_at | KLK7 | kallikrein-related peptidase 7 | 0.1 | 24.2 | 221.3 | 3.6 x 10^-6^ |
| 206125_s_at | KLK8 | kallikrein-related peptidase 8 | 0.7 | 8.4 | 11.9 | 2.8 x 10^-6^ |
| 209800_at | KRT16 | keratin 16 | 0.1 | 88.7 | 635.4 | 2.7 x 10^-8^ |
| 205157_s_at | KRT17 | keratin 17 | 10.7 | 537.9 | 50.3 | 1.9 x 10^-8^ |
| 201820_at | KRT5 | keratin 5 | 44.5 | 382.6 | 8.6 | 1.6 x 10^-5^ |
| 209125_at | KRT6A | keratin 6A | 1.1 | 724.4 | 667.2 | 3.2 x 10^-9^ |
| 209126_x_at | KRT6B | keratin 6B | 1.8 | 353.0 | 196.2 | 3.6 x 10^-10^ |
| 203726_s_at | LAMA3 | laminin, alpha 3 | 5.4 | 148.4 | 27.6 | 1.3 x 10^-5^ |
| 209270_at | LAMB3 | laminin, beta 3 | 15.9 | 297.3 | 18.7 | 2.8 x 10^-9^ |
| 200770_s_at | LAMC1 | laminin, gamma 1 (formerly LAMB2) | 3.5 | 45.0 | 12.9 | 5.5 x 10^-8^ |
| 202267_at | LAMC2 | laminin, gamma 2 | 11.8 | 417.2 | 35.2 | 1.6 x 10^-6^ |
| 212086_x_at | LMNA | lamin A/C | 7.5 | 45.8 | 6.1 | 3.4 x 10^-6^ |
| 206091_at | MATN3 | matrilin 3 | 0.2 | 1.0 | 6.2 | 3.5 x 10^-6^ |
| 210869_s_at | MCAM | melanoma cell adhesion molecule | 0.6 | 14.8 | 23.3 | 9.2 x 10^-3^ |
| 222530_s_at | MKKS | McKusick-Kaufman syndrome | 6.7 | 41.1 | 6.2 | 1.2 x 10^-6^ |
| 207738_s_at | NCKAP1 | NCK-associated protein 1 | 26.8 | 152.6 | 5.7 | 1.4 x 10^-6^ |
| 205460_at | NPAS2 | neuronal PAS domain protein 2 | 0.4 | 4.7 | 10.7 | 1.2 x 10^-3^ |
| 206343_s_at | NRG1 | neuregulin 1 | 0.4 | 19.8 | 53.4 | 7.4 x 10^-7^ |
| 210510_s_at | NRP1 | neuropilin 1 | 0.2 | 2.7 | 13.0 | 2.4 x 10^-7^ |
| 236088_at | NTNG1 | netrin G1 | 0.4 | 2.3 | 6.4 | 8.3 x 10^-5^ |
| 218718_at | PDGFC | platelet derived growth factor C | 12.7 | 78.6 | 6.2 | 2.6 x 10^-7^ |
| 221994_at | PDLIM5 | PDZ and LIM domain 5 | 0.4 | 1.9 | 5.0 | 2.9 x 10^-3^ |
| 209652_s_at | PGF | placental growth factor | 0.4 | 7.3 | 17.9 | 7.2 x 10^-7^ |
| 201397_at | PHGDH | phosphoglycerate dehydrogenase | 4.1 | 33.4 | 8.2 | 2.3 x 10^-9^ |
| 210355_at | PTHLH | parathyroid hormone-like hormone | 0.6 | 18.6 | 32.3 | 2.8 x 10^-4^ |
| 219550_at | ROBO3 | roundabout, axon guidance receptor, homolog 3 (Drosophila) | 0.6 | 4.2 | 7.4 | 9.4 x 10^-6^ |
| 206884_s_at | SCEL | sciellin | 0.4 | 73.3 | 194.6 | 2.0 x 10^-7^ |
| 233565_s_at | SDCBP2 | syndecan binding protein (syntenin) 2 | 3.8 | 24.0 | 6.3 | 8.6 x 10^-6^ |
| 203071_at | SEMA3B | sema domain, immunoglobulin domain (Ig), short basic domain, secreted, (semaphorin) 3B | 0.3 | 3.6 | 11.8 | 2.3 x 10^-5^ |
| 212190_at | SERPINE2 | serpin peptidase inhibitor, clade E (nexin, plasminogen activator inhibitor type 1), member 2 | 0.9 | 85.0 | 91.4 | 1.9 x 10^-9^ |
| 207302_at | SGCG | sarcoglycan, gamma (35kDa dystrophin-associated glycoprotein) | 0.1 | 0.7 | 10.5 | 2.3 x 10^-4^ |
| 214853_s_at | SHC1 | SHC (Src homology 2 domain containing) transforming protein 1 | 14.9 | 204.0 | 13.7 | 3.3 x 10^-8^ |
| 200924_s_at | SLC3A2 | solute carrier family 3 (activators of dibasic and neutral amino acid transport), member 2 | 2.9 | 84.6 | 29.1 | 2.4 x 10^-8^ |
| 213139_at | SNAI2 | snail homolog 2 (Drosophila) | 6.0 | 71.9 | 12.0 | 2.0 x 10^-4^ |
| 203373_at | SOCS2 | suppressor of cytokine signaling 2 | 1.5 | 10.0 | 6.5 | 1.2 x 10^-5^ |
| 206122_at | SOX15 | SRY (sex determining region Y)-box 15 | 1.9 | 27.0 | 14.0 | 2.9 x 10^-8^ |
| 213796_at | SPRR1A | small proline-rich protein 1A | 0.2 | 83.9 | 371.4 | 2.2 x 10^-6^ |
| 205064_at | SPRR1B | small proline-rich protein 1B (cornifin) | 0.7 | 234.3 | 345.5 | 5.4 x 10^-8^ |
| 208539_x_at | SPRR2B | small proline-rich protein 2B | 0.9 | 30.4 | 35.2 | 3.1 x 10^-4^ |
| 41037_at | TEAD4 | TEA domain family member 4 | 0.4 | 2.7 | 6.0 | 4.3 x 10^-7^ |
| 204653_at | TFAP2A | transcription factor AP-2 alpha (activating enhancer binding protein 2 alpha) | 9.9 | 64.2 | 6.5 | 2.8 x 10^-9^ |
| 203085_s_at | TGFB1 | transforming growth factor, beta 1 | 2.6 | 14.9 | 5.7 | 1.2 x 10^-4^ |
| 209909_s_at | TGFB2 | transforming growth factor, beta 2 | 0.8 | 4.6 | 6.0 | 2.9 x 10^-4^ |
| 201109_s_at | THBS1 | thrombospondin 1 | 0.5 | 59.4 | 110.4 | 7.3 x 10^-3^ |
| 228284_at | TLE1 | transducin-like enhancer of split 1 (E(sp1) homolog, Drosophila) | 1.7 | 18.4 | 11.0 | 2.0 x 10^-11^ |
| 218368_s_at | TNFRSF12A | tumor necrosis factor receptor superfamily, member 12A | 2.7 | 69.4 | 25.5 | 1.3 x 10^-5^ |
| 213201_s_at | TNNT1 | troponin T type 1 (skeletal, slow) | 0.5 | 4.5 | 9.9 | 1.3 x 10^-4^ |
| 210986_s_at | TPM1 | tropomyosin 1 (alpha) | 16.5 | 90.2 | 5.4 | 8.9 x 10^-6^ |
| 1567107_s_at | TPM4 | tropomyosin 4 | 1.1 | 24.3 | 22.7 | 1.1 x 10^-6^ |
| 200743_s_at | TPP1 | tripeptidyl peptidase I | 10.2 | 52.2 | 5.1 | 7.3 x 10^-6^ |
| 205807_s_at | TUFT1 | tuftelin 1 | 8.1 | 41.0 | 5.1 | 5.4 x 10^-7^ |
| 229404_at | TWIST2 | twist homolog 2 (Drosophila) | 0.5 | 3.7 | 7.7 | 4.0 x 10^-3^ |
| 211527_x_at | VEGFA | vascular endothelial growth factor A | 4.2 | 35.1 | 8.3 | 1.6 x 10^-5^ |
| 209946_at | VEGFC | vascular endothelial growth factor C | 1.3 | 9.1 | 7.2 | 2.2 x 10^-5^ |
| 209053_s_at | WHSC1 | Wolf-Hirschhorn syndrome candidate 1 | 1.4 | 15.7 | 11.0 | 4.9 x 10^-6^ |
| 210248_at | WNT7A | wingless-type MMTV integration site family, member 7A | 0.2 | 2.9 | 15.7 | 1.0 x 10^-5^ |
| **Cell proliferation** | | |  |  |  |  |
| 222162_s_at | ADAMTS1 | ADAM metallopeptidase with thrombospondin type 1 motif, 1 | 0.1 | 43.1 | 327.7 | 4.8 x 10^-6^ |
| 206714_at | ALOX15B | arachidonate 15-lipoxygenase, type B | 0.4 | 2.8 | 6.8 | 2.2 x 10^-3^ |
| 224010_at | ANAPC11 | anaphase promoting complex subunit 11 | 0.4 | 2.0 | 5.1 | 6.6 x 10^-4^ |
| 213419_at | APBB2 | amyloid beta (A4) precursor protein-binding, family B, member 2 | 0.9 | 8.0 | 8.8 | 5.9 x 10^-7^ |
| 205239_at | AREG | amphiregulin | 2.7 | 354.7 | 133.9 | 8.5 x 10^-10^ |
| 210237_at | ARTN | artemin | 0.4 | 9.0 | 21.7 | 3.4 x 10^-4^ |
| 202686_s_at | AXL | AXL receptor tyrosine kinase | 5.7 | 34.6 | 6.0 | 4.5 x 10^-5^ |
| 226517_at | BCAT1 | branched chain aminotransferase 1, cytosolic | 0.6 | 9.8 | 17.2 | 8.7 x 10^-5^ |
| 1557257_at | BCL10 | B-cell CLL/lymphoma 10 | 0.8 | 6.5 | 8.4 | 3.0 x 10^-3^ |
| 1552487_a_at | BNC1 | basonuclin 1 | 0.5 | 35.1 | 69.7 | 9.4 x 10^-5^ |
| 205899_at | CCNA1 | cyclin A1 | 4.4 | 42.8 | 9.7 | 6.2 x 10^-3^ |
| 200951_s_at | CCND2 | cyclin D2 | 0.5 | 12.4 | 26.2 | 2.5 x 10^-7^ |
| 213523_at | CCNE1 | cyclin E1 | 0.7 | 3.7 | 5.1 | 1.9 x 10^-8^ |
| 201853_s_at | CDC25B | cell division cycle 25 homolog B (S. pombe) | 2.3 | 21.6 | 9.3 | 5.2 x 10^-6^ |
| 203967_at | CDC6 | cell division cycle 6 homolog (S. cerevisiae) | 0.2 | 4.4 | 18.8 | 7.8 x 10^-3^ |
| 224851_at | CDK6 | cyclin-dependent kinase 6 | 7.8 | 53.2 | 6.8 | 1.1 x 10^-6^ |
| 1553113_s_at | CDK8 | cyclin-dependent kinase 8 | 2.5 | 15.7 | 6.2 | 4.6 x 10^-7^ |
| 202284_s_at | CDKN1A | cyclin-dependent kinase inhibitor 1A (p21, Cip1) | 10.7 | 167.8 | 15.7 | 2.0 x 10^-6^ |
| 1555758_a_at | CDKN3 | cyclin-dependent kinase inhibitor 3 | 0.4 | 17.0 | 39.7 | 4.8 x 10^-3^ |
| 205393_s_at | CHEK1 | CHK1 checkpoint homolog (S. pombe) | 0.5 | 4.3 | 8.5 | 8.8 x 10^-4^ |
| 207030_s_at | CSRP2 | cysteine and glycine-rich protein 2 | 2.3 | 51.6 | 22.1 | 7.4 x 10^-7^ |
| 227757_at | CUL4A | cullin 4A | 0.6 | 2.9 | 5.2 | 6.1 x 10^-4^ |
| 1555895_at | DNM2 | dynamin 2 | 0.5 | 2.5 | 5.2 | 7.0 x 10^-5^ |
| 216918_s_at | DST | dystonin | 3.3 | 99.3 | 30.2 | 2.0 x 10^-8^ |
| 208892_s_at | DUSP6 | dual specificity phosphatase 6 | 13.6 | 87.4 | 6.4 | 3.0 x 10^-7^ |
| 203693_s_at | E2F3 | E2F transcription factor 3 | 1.0 | 8.5 | 8.1 | 4.6 x 10^-10^ |
| 228033_at | E2F7 | E2F transcription factor 7 | 2.5 | 20.7 | 8.2 | 1.2 x 10^-4^ |
| 1564630_at | EDN1 | endothelin 1 | 0.2 | 7.5 | 31.7 | 5.2 x 10^-5^ |
| 201984_s_at | EGFR | epidermal growth factor receptor (erythroblastic leukemia viral (v-erb-b) oncogene homolog, avian) | 9.2 | 93.6 | 10.2 | 9.1 x 10^-7^ |
| 201324_at | EMP1 | epithelial membrane protein 1 | 5.4 | 97.9 | 18.1 | 2.5 x 10^-5^ |
| 202894_at | EPHB4 | EPH receptor B4 | 3.2 | 19.1 | 6.0 | 9.8 x 10^-8^ |
| 205767_at | EREG | epiregulin | 0.1 | 34.6 | 246.0 | 8.0 x 10^-7^ |
| 224833_at | ETS1 | v-ets erythroblastosis virus E26 oncogene homolog 1 (avian) | 6.4 | 94.6 | 14.8 | 3.1 x 10^-6^ |
| 204422_s_at | FGF2 | fibroblast growth factor 2 (basic) | 0.2 | 1.8 | 7.7 | 2.7 x 10^-3^ |
| 204420_at | FOSL1 | FOS-like antigen 1 | 0.7 | 22.9 | 30.7 | 6.1 x 10^-4^ |
| 201564_s_at | FSCN1 | fascin homolog 1, actin-bundling protein (Strongylocentrotus purpuratus) | 0.3 | 26.9 | 89.0 | 1.3 x 10^-5^ |
| 213524_s_at | G0S2 | G0/G1switch 2 | 3.5 | 99.0 | 28.1 | 2.0 x 10^-4^ |
| 203725_at | GADD45A | growth arrest and DNA-damage-inducible, alpha | 13.3 | 135.4 | 10.2 | 4.4 x 10^-10^ |
| 211284_s_at | GRN | granulin | 6.2 | 37.6 | 6.1 | 9.4 x 10^-7^ |
| 38037_at | HBEGF | heparin-binding EGF-like growth factor | 3.8 | 65.4 | 17.3 | 6.0 x 10^-7^ |
| 209526_s_at | HDGFRP3 | hepatoma-derived growth factor, related protein 3 | 5.4 | 35.1 | 6.5 | 3.2 x 10^-8^ |
| 202934_at | HK2 | hexokinase 2 | 4.0 | 41.4 | 10.3 | 2.9 x 10^-5^ |
| 206074_s_at | HMGA1 | high mobility group AT-hook 1 | 7.6 | 95.0 | 12.4 | 1.3 x 10^-6^ |
| 209100_at | IFRD2 | interferon-related developmental regulator 2 | 4.9 | 25.4 | 5.1 | 3.1 x 10^-5^ |
| 203851_at | IGFBP6 | insulin-like growth factor binding protein 6 | 1.6 | 13.8 | 8.5 | 1.7 x 10^-4^ |
| 206295_at | IL18 | interleukin 18 (interferon-gamma-inducing factor) | 4.0 | 26.5 | 6.6 | 5.2 x 10^-9^ |
| 205067_at | IL1B | interleukin 1, beta | 1.8 | 20.9 | 11.7 | 6.3 x 10^-3^ |
| 216268_s_at | JAG1 | jagged 1 (Alagille syndrome) | 23.3 | 249.3 | 10.7 | 4.0 x 10^-7^ |
| 209792_s_at | KLK10 | kallikrein-related peptidase 10 | 9.2 | 77.4 | 8.4 | 3.8 x 10^-5^ |
| 201088_at | KPNA2 | karyopherin alpha 2 (RAG cohort 1, importin alpha 1) | 13.3 | 82.7 | 6.2 | 2.2 x 10^-3^ |
| 209800_at | KRT16 | keratin 16 | 0.1 | 88.7 | 635.4 | 2.7 x 10^-8^ |
| 205569_at | LAMP3 | lysosomal-associated membrane protein 3 | 9.6 | 63.1 | 6.5 | 1.9 x 10^-3^ |
| 230348_at | LATS2 | LATS, large tumor suppressor, homolog 2 (Drosophila) | 0.2 | 1.5 | 8.9 | 3.3 x 10^-4^ |
| 223234_at | MAD2L2 | MAD2 mitotic arrest deficient-like 2 (yeast) | 2.4 | 16.5 | 6.8 | 4.5 x 10^-9^ |
| 210058_at | MAPK13 | mitogen-activated protein kinase 13 | 4.0 | 24.8 | 6.2 | 1.4 x 10^-5^ |
| 207121_s_at | MAPK6 | mitogen-activated protein kinase 6 | 27.7 | 163.3 | 5.9 | 3.8 x 10^-11^ |
| 226225_at | MCC | mutated in colorectal cancers | 3.9 | 24.6 | 6.3 | 8.8 x 10^-6^ |
| 203510_at | MET | met proto-oncogene (hepatocyte growth factor receptor) | 30.9 | 195.3 | 6.3 | 3.8 x 10^-7^ |
| 205330_at | MN1 | meningioma (disrupted in balanced translocation) 1 | 0.5 | 5.6 | 12.2 | 8.6 x 10^-8^ |
| 203740_at | MPHOSPH6 | M-phase phosphoprotein 6 | 10.2 | 63.9 | 6.3 | 2.4 x 10^-9^ |
| 228846_at | MXD1 | MAX dimerization protein 1 | 3.3 | 35.4 | 10.7 | 2.6 x 10^-6^ |
| 202431_s_at | MYC | v-myc myelocytomatosis viral oncogene homolog (avian) | 9.6 | 90.1 | 9.4 | 8.1 x 10^-5^ |
| 204528_s_at | NAP1L1 | nucleosome assembly protein 1-like 1 | 21.6 | 114.3 | 5.3 | 1.3 x 10^-5^ |
| 217150_s_at | NF2 | neurofibromin 2 (merlin) | 0.8 | 4.2 | 5.3 | 7.4 x 10^-5^ |
| 201577_at | NME1 | non-metastatic cells 1, protein (NM23A) expressed in | 11.2 | 56.8 | 5.1 | 2.1 x 10^-4^ |
| 221923_s_at | NPM1 | nucleophosmin (nucleolar phosphoprotein B23, numatrin) | 20.0 | 120.8 | 6.0 | 9.0 x 10^-8^ |
| 202647_s_at | NRAS | neuroblastoma RAS viral (v-ras) oncogene homolog | 5.1 | 32.2 | 6.3 | 5.1 x 10^-6^ |
| 210510_s_at | NRP1 | neuropilin 1 | 0.2 | 2.7 | 13.0 | 2.4 x 10^-7^ |
| 1554008_at | OSMR | oncostatin M receptor | 1.4 | 16.2 | 12.0 | 3.0 x 10^-6^ |
| 232132_at | PARD6G | par-6 partitioning defective 6 homolog gamma (C. elegans) | 0.2 | 1.5 | 9.5 | 8.6 x 10^-6^ |
| 204005_s_at | PAWR | PRKC, apoptosis, WT1, regulator | 5.2 | 47.8 | 9.2 | 3.1 x 10^-6^ |
| 208824_x_at | PCTK1 | PCTAIRE protein kinase 1 | 4.0 | 20.1 | 5.0 | 6.6 x 10^-5^ |
| 205463_s_at | PDGFA | platelet-derived growth factor alpha polypeptide | 3.0 | 19.4 | 6.4 | 5.8 x 10^-6^ |
| 209652_s_at | PGF | placental growth factor | 0.4 | 7.3 | 17.9 | 7.2 x 10^-7^ |
| 204958_at | PLK3 | polo-like kinase 3 (Drosophila) | 0.9 | 4.7 | 5.2 | 1.5 x 10^-7^ |
| 210355_at | PTHLH | parathyroid hormone-like hormone | 0.6 | 18.6 | 32.3 | 2.8 x 10^-4^ |
| 208511_at | PTTG3 | pituitary tumor-transforming 3 | 0.5 | 2.7 | 5.2 | 7.9 x 10^-3^ |
| 207080_s_at | PYY | peptide YY | 0.1 | 1.0 | 7.1 | 7.6 x 10^-5^ |
| 212104_s_at | RBM9 | RNA binding motif protein 9 | 6.7 | 40.9 | 6.1 | 8.1 x 10^-10^ |
| 202131_s_at | RIOK3 | RIO kinase 3 (yeast) | 8.4 | 45.5 | 5.4 | 5.5 x 10^-4^ |
| 204078_at | SC65 | synaptonemal complex protein SC65 | 0.9 | 5.2 | 5.6 | 1.1 x 10^-7^ |
| 223195_s_at | SESN2 | sestrin 2 | 2.3 | 29.8 | 12.7 | 1.1 x 10^-4^ |
| 209260_at | SFN | stratifin | 4.0 | 141.0 | 35.4 | 2.2 x 10^-4^ |
| 214853_s_at | SHC1 | SHC (Src homology 2 domain containing) transforming protein 1 | 14.9 | 204.0 | 13.7 | 3.3 x 10^-8^ |
| 200889_s_at | SSR1 | signal sequence receptor, alpha | 10.2 | 55.6 | 5.5 | 1.0 x 10^-6^ |
| 217437_s_at | TACC1 | transforming, acidic coiled-coil containing protein 1 | 1.7 | 9.5 | 5.4 | 1.5 x 10^-5^ |
| 220789_s_at | TBRG4 | transforming growth factor beta regulator 4 | 1.3 | 10.5 | 7.9 | 9.0 x 10^-5^ |
| 205016_at | TGFA | transforming growth factor, alpha | 7.4 | 59.7 | 8.1 | 4.5 x 10^-11^ |
| 203085_s_at | TGFB1 | transforming growth factor, beta 1 | 2.6 | 14.9 | 5.7 | 1.2 x 10^-4^ |
| 209651_at | TGFB1I1 | transforming growth factor beta 1 induced transcript 1 | 0.7 | 10.9 | 14.6 | 6.1 x 10^-5^ |
| 209909_s_at | TGFB2 | transforming growth factor, beta 2 | 0.8 | 4.6 | 6.0 | 2.9 x 10^-4^ |
| 201506_at | TGFBI | transforming growth factor, beta-induced, 68kDa | 6.8 | 243.7 | 36.1 | 1.9 x 10^-4^ |
| 201666_at | TIMP1 | TIMP metallopeptidase inhibitor 1 | 25.2 | 215.4 | 8.6 | 1.8 x 10^-6^ |
| 209096_at | UBE2V2 | ubiquitin-conjugating enzyme E2 variant 2 | 13.0 | 65.6 | 5.0 | 3.9 x 10^-6^ |
| 211527_x_at | VEGFA | vascular endothelial growth factor A | 4.2 | 35.1 | 8.3 | 1.6 x 10^-5^ |
| 209946_at | VEGFC | vascular endothelial growth factor C | 1.3 | 9.1 | 7.2 | 2.2 x 10^-5^ |
| 225662_at | ZAK | sterile alpha motif and leucine zipper containing kinase AZK | 3.4 | 37.8 | 11.2 | 3.6 x 10^-7^ |
| 1554158_at | ZMYND11 | zinc finger, MYND domain containing 11 | 0.2 | 0.9 | 5.1 | 9.0 x 10^-3^ |
| **Purine base biosynthesis** | | |  |  |  |  |
| 210005_at | GART | phosphoribosylglycinamide formyltransferase, phosphoribosylglycinamide synthetase, phosphoribosylaminoimidazole synthetase | 1.2 | 7.1 | 5.8 | 6.2 x 10^-8^ |
| 214431_at | GMPS | guanine monphosphate synthetase | 4.1 | 30.2 | 7.3 | 1.3 x 10^-6^ |
| 201014_s_at | PAICS | phosphoribosylaminoimidazole carboxylase, phosphoribosylaminoimidazole succinocarboxamide synthetase | 5.4 | 38.1 | 7.0 | 1.6 x 10^-10^ |
| 209433_s_at | PPAT | phosphoribosyl pyrophosphate amidotransferase | 2.8 | 13.8 | 5.0 | 3.0 x 10^-5^ |
| **Keratinocyte differentiation** | | |  |  |  |  |
| 214599_at | IVL | involucrin | 0.2 | 5.4 | 22.7 | 2.8 x 10^-3^ |
| 216268_s_at | JAG1 | jagged 1 (Alagille syndrome) | 23.3 | 249.3 | 10.7 | 4.0 x 10^-7^ |
| 203726_s_at | LAMA3 | laminin, alpha 3 | 5.4 | 148.4 | 27.6 | 1.3 x 10^-5^ |
| 204748_at | PTGS2 | prostaglandin-endoperoxide synthase 2 (prostaglandin G/H synthase and cyclooxygenase) | 2.8 | 44.1 | 15.9 | 2.9 x 10^-4^ |
| 208539_x_at | SPRR2B | small proline-rich protein 2B | 0.9 | 30.4 | 35.2 | 3.1 x 10^-4^ |
| **Purine base metabolism** | | |  |  |  |  |
| 210005_at | GART | phosphoribosylglycinamide formyltransferase, phosphoribosylglycinamide synthetase, phosphoribosylaminoimidazole synthetase | 1.2 | 7.1 | 5.8 | 6.2 x 10^-8^ |
| 214431_at | GMPS | guanine monphosphate synthetase | 4.1 | 30.2 | 7.3 | 1.3 x 10^-6^ |
| 201014_s_at | PAICS | phosphoribosylaminoimidazole carboxylase, phosphoribosylaminoimidazole succinocarboxamide synthetase | 5.4 | 38.1 | 7.0 | 1.6 x 10^-10^ |
| 209433_s_at | PPAT | phosphoribosyl pyrophosphate amidotransferase | 2.8 | 13.8 | 5.0 | 3.0 x 10^-5^ |
| **Cell cycle** |  |  |  |  |  |  |
| 206714_at | ALOX15B | arachidonate 15-lipoxygenase, type B | 0.4 | 2.8 | 6.8 | 2.2 x 10^-3^ |
| 224010_at | ANAPC11 | anaphase promoting complex subunit 11 | 0.4 | 2.0 | 5.1 | 6.6 x 10^-4^ |
| 213419_at | APBB2 | amyloid beta (A4) precursor protein-binding, family B, member 2 | 0.9 | 8.0 | 8.8 | 5.9 x 10^-7^ |
| 202686_s_at | AXL | AXL receptor tyrosine kinase | 5.7 | 34.6 | 6.0 | 4.5 x 10^-5^ |
| 226517_at | BCAT1 | branched chain aminotransferase 1, cytosolic | 0.6 | 9.8 | 17.2 | 8.7 x 10^-5^ |
| 1557257_at | BCL10 | B-cell CLL/lymphoma 10 | 0.8 | 6.5 | 8.4 | 3.0 x 10^-3^ |
| 205899_at | CCNA1 | cyclin A1 | 4.4 | 42.8 | 9.7 | 6.2 x 10^-3^ |
| 200951_s_at | CCND2 | cyclin D2 | 0.5 | 12.4 | 26.2 | 2.5 x 10^-7^ |
| 213523_at | CCNE1 | cyclin E1 | 0.7 | 3.7 | 5.1 | 1.9 x 10^-8^ |
| 201853_s_at | CDC25B | cell division cycle 25 homolog B (S. pombe) | 2.3 | 21.6 | 9.3 | 5.2 x 10^-6^ |
| 203967_at | CDC6 | cell division cycle 6 homolog (S. cerevisiae) | 0.2 | 4.4 | 18.8 | 7.8 x 10^-3^ |
| 224851_at | CDK6 | cyclin-dependent kinase 6 | 7.8 | 53.2 | 6.8 | 1.1 x 10^-6^ |
| 1553113_s_at | CDK8 | cyclin-dependent kinase 8 | 2.5 | 15.7 | 6.2 | 4.6 x 10^-7^ |
| 202284_s_at | CDKN1A | cyclin-dependent kinase inhibitor 1A (p21, Cip1) | 10.7 | 167.8 | 15.7 | 2.0 x 10^-6^ |
| 1555758_a_at | CDKN3 | cyclin-dependent kinase inhibitor 3 | 0.4 | 17.0 | 39.7 | 4.8 x 10^-3^ |
| 205393_s_at | CHEK1 | CHK1 checkpoint homolog (S. pombe) | 0.5 | 4.3 | 8.5 | 8.8 x 10^-4^ |
| 227757_at | CUL4A | cullin 4A | 0.6 | 2.9 | 5.2 | 6.1 x 10^-4^ |
| 1555895_at | DNM2 | dynamin 2 | 0.5 | 2.5 | 5.2 | 7.0 x 10^-5^ |
| 216918_s_at | DST | dystonin | 3.3 | 99.3 | 30.2 | 2.0 x 10^-8^ |
| 208892_s_at | DUSP6 | dual specificity phosphatase 6 | 13.6 | 87.4 | 6.4 | 3.0 x 10^-7^ |
| 203693_s_at | E2F3 | E2F transcription factor 3 | 1.0 | 8.5 | 8.1 | 4.6 x 10^-10^ |
| 228033_at | E2F7 | E2F transcription factor 7 | 2.5 | 20.7 | 8.2 | 1.2 x 10^-4^ |
| 201984_s_at | EGFR | epidermal growth factor receptor (erythroblastic leukemia viral (v-erb-b) oncogene homolog, avian) | 9.2 | 93.6 | 10.2 | 9.1 x 10^-7^ |
| 205767_at | EREG | epiregulin | 0.1 | 34.6 | 246.0 | 8.0 x 10^-7^ |
| 204422_s_at | FGF2 | fibroblast growth factor 2 (basic) | 0.2 | 1.8 | 7.7 | 2.7 x 10^-3^ |
| 213524_s_at | G0S2 | G0/G1switch 2 | 3.5 | 99.0 | 28.1 | 2.0 x 10^-4^ |
| 203725_at | GADD45A | growth arrest and DNA-damage-inducible, alpha | 13.3 | 135.4 | 10.2 | 4.4 x 10^-10^ |
| 202934_at | HK2 | hexokinase 2 | 4.0 | 41.4 | 10.3 | 2.9 x 10^-5^ |
| 206074_s_at | HMGA1 | high mobility group AT-hook 1 | 7.6 | 95.0 | 12.4 | 1.3 x 10^-6^ |
| 205067_at | IL1B | interleukin 1, beta | 1.8 | 20.9 | 11.7 | 6.3 x 10^-3^ |
| 209792_s_at | KLK10 | kallikrein-related peptidase 10 | 9.2 | 77.4 | 8.4 | 3.8 x 10^-5^ |
| 201088_at | KPNA2 | karyopherin alpha 2 (RAG cohort 1, importin alpha 1) | 13.3 | 82.7 | 6.2 | 2.2 x 10^-3^ |
| 230348_at | LATS2 | LATS, large tumor suppressor, homolog 2 (Drosophila) | 0.2 | 1.5 | 8.9 | 3.3 x 10^-4^ |
| 223234_at | MAD2L2 | MAD2 mitotic arrest deficient-like 2 (yeast) | 2.4 | 16.5 | 6.8 | 4.5 x 10^-9^ |
| 210058_at | MAPK13 | mitogen-activated protein kinase 13 | 4.0 | 24.8 | 6.2 | 1.4 x 10^-5^ |
| 207121_s_at | MAPK6 | mitogen-activated protein kinase 6 | 27.7 | 163.3 | 5.9 | 3.8 x 10^-11^ |
| 226225_at | MCC | mutated in colorectal cancers | 3.9 | 24.6 | 6.3 | 8.8 x 10^-6^ |
| 205330_at | MN1 | meningioma (disrupted in balanced translocation) 1 | 0.5 | 5.6 | 12.2 | 8.6 x 10^-8^ |
| 203740_at | MPHOSPH6 | M-phase phosphoprotein 6 | 10.2 | 63.9 | 6.3 | 2.4 x 10^-9^ |
| 202431_s_at | MYC | v-myc myelocytomatosis viral oncogene homolog (avian) | 9.6 | 90.1 | 9.4 | 8.1 x 10^-5^ |
| 204528_s_at | NAP1L1 | nucleosome assembly protein 1-like 1 | 21.6 | 114.3 | 5.3 | 1.3 x 10^-5^ |
| 217150_s_at | NF2 | neurofibromin 2 (merlin) | 0.8 | 4.2 | 5.3 | 7.4 x 10^-5^ |
| 201577_at | NME1 | non-metastatic cells 1, protein (NM23A) expressed in | 11.2 | 56.8 | 5.1 | 2.1 x 10^-4^ |
| 221923_s_at | NPM1 | nucleophosmin (nucleolar phosphoprotein B23, numatrin) | 20.0 | 120.8 | 6.0 | 9.0 x 10^-8^ |
| 202647_s_at | NRAS | neuroblastoma RAS viral (v-ras) oncogene homolog | 5.1 | 32.2 | 6.3 | 5.1 x 10^-6^ |
| 232132_at | PARD6G | par-6 partitioning defective 6 homolog gamma (C. elegans) | 0.2 | 1.5 | 9.5 | 8.6 x 10^-6^ |
| 208824_x_at | PCTK1 | PCTAIRE protein kinase 1 | 4.0 | 20.1 | 5.0 | 6.6 x 10^-5^ |
| 205463_s_at | PDGFA | platelet-derived growth factor alpha polypeptide | 3.0 | 19.4 | 6.4 | 5.8 x 10^-6^ |
| 209652_s_at | PGF | placental growth factor | 0.4 | 7.3 | 17.9 | 7.2 x 10^-7^ |
| 204958_at | PLK3 | polo-like kinase 3 (Drosophila) | 0.9 | 4.7 | 5.2 | 1.5 x 10^-7^ |
| 208511_at | PTTG3 | pituitary tumor-transforming 3 | 0.5 | 2.7 | 5.2 | 7.9 x 10^-3^ |
| 202131_s_at | RIOK3 | RIO kinase 3 (yeast) | 8.4 | 45.5 | 5.4 | 5.5 x 10^-4^ |
| 204078_at | SC65 | synaptonemal complex protein SC65 | 0.9 | 5.2 | 5.6 | 1.1 x 10^-7^ |
| 223195_s_at | SESN2 | sestrin 2 | 2.3 | 29.8 | 12.7 | 1.1 x 10^-4^ |
| 209260_at | SFN | stratifin | 4.0 | 141.0 | 35.4 | 2.2 x 10^-4^ |
| 214853_s_at | SHC1 | SHC (Src homology 2 domain containing) transforming protein 1 | 14.9 | 204.0 | 13.7 | 3.3 x 10^-8^ |
| 217437_s_at | TACC1 | transforming, acidic coiled-coil containing protein 1 | 1.7 | 9.5 | 5.4 | 1.5 x 10^-5^ |
| 220789_s_at | TBRG4 | transforming growth factor beta regulator 4 | 1.3 | 10.5 | 7.9 | 9.0 x 10^-5^ |
| 205016_at | TGFA | transforming growth factor, alpha | 7.4 | 59.7 | 8.1 | 4.5 x 10^-11^ |
| 203085_s_at | TGFB1 | transforming growth factor, beta 1 | 2.6 | 14.9 | 5.7 | 1.2 x 10^-4^ |
| 209909_s_at | TGFB2 | transforming growth factor, beta 2 | 0.8 | 4.6 | 6.0 | 2.9 x 10^-4^ |
| 209096_at | UBE2V2 | ubiquitin-conjugating enzyme E2 variant 2 | 13.0 | 65.6 | 5.0 | 3.9 x 10^-6^ |
| 211527_x_at | VEGFA | vascular endothelial growth factor A | 4.2 | 35.1 | 8.3 | 1.6 x 10^-5^ |
| 209946_at | VEGFC | vascular endothelial growth factor C | 1.3 | 9.1 | 7.2 | 2.2 x 10^-5^ |
| 225662_at | ZAK | sterile alpha motif and leucine zipper containing kinase AZK | 3.4 | 37.8 | 11.2 | 3.6 x 10^-7^ |
| 1554158_at | ZMYND11 | zinc finger, MYND domain containing 11 | 0.2 | 0.9 | 5.1 | 9.0 x 10^-3^ |

1. For all significant Gene Ontology categories identified by GATHER analysis (Table 3) the genes overlapping with the basal cell signature were identified, and the basal and differentiated epithelium expression ratios were extracted.
2. p value following Benjamini-Hochberg correction.
