# Supplementary material for: The Human Airway Epithelial Basal Cell Transcriptome
Source: PLoS One. 2011 May 4;6(5):e18378. doi: 10.1371/journal.pone.0018378 (PMC3087716; doi:10.1371/journal.pone.0018378)
Supplement: Table S6 — Genes Comprising the Significant Canonical Pathways of the Human Basal Cell Signature. (DOC) [file pone.0018378.s006.doc]

| **ProbeSetID** | **Gene symbol** | **Gene title** | **Mean expression in differentiated epithelium** | **Mean expression in basal cells** | **Basal/differentiated epithelium expression ratio** | **p value^2^** |
| --- | --- | --- | --- | --- | --- | --- |
|  | | |  |  |  |  |
| **Neuregulin signaling** | | |  |  |  |  |
| 213532_at | ADAM17 | ADAM metallopeptidase domain 17 | 5.8 | 30.7 | 5.3 | 5.3 x 10^-9^ |
| 212607_at | AKT3 | v-akt murine thymoma viral oncogene homolog 3 (protein kinase B, gamma) | 1.5 | 9.3 | 6.2 | 6.8 x 10^-7^ |
| 205239_at | AREG | amphiregulin | 2.7 | 354.7 | 133.9 | 8.5 x 10^-10^ |
| 202224_at | CRK | v-crk sarcoma virus CT10 oncogene homolog (avian) | 6.5 | 34.6 | 5.3 | 1.6 x 10^-9^ |
| 201984_s_at | EGFR | epidermal growth factor receptor (erythroblastic leukemia viral (v-erb-b) oncogene homolog, avian) | 9.2 | 93.6 | 10.2 | 9.1 x 10^-7^ |
| 205767_at | EREG | epiregulin | 0.1 | 34.6 | 246.0 | 8.0 x 10^-7^ |
| 224657_at | ERRFI1 | ERBB receptor feedback inhibitor 1 | 16.3 | 280.0 | 17.2 | 4.4 x 10^-11^ |
| 38037_at | HBEGF | heparin-binding EGF-like growth factor | 3.8 | 65.4 | 17.3 | 6.0 x 10^-7^ |
| 201474_s_at | ITGA3 | integrin, alpha 3 (antigen CD49C, alpha 3 subunit of VLA-3 receptor) | 3.2 | 50.1 | 15.8 | 5.0 x 10^-9^ |
| 201389_at | ITGA5 | integrin, alpha 5 (fibronectin receptor, alpha polypeptide) | 0.7 | 18.3 | 27.3 | 2.9 x 10^-6^ |
| 1553530_a_at | ITGB1 | integrin, beta 1 (fibronectin receptor, beta polypeptide, antigen CD29 includes MDF2, MSK12) | 16.9 | 216.8 | 12.8 | 7.4 x 10^-7^ |
| 202670_at | MAP2K1 | mitogen-activated protein kinase kinase 1 | 8.4 | 58.5 | 7.0 | 1.5 x 10^-9^ |
| 202431_s_at | MYC | v-myc myelocytomatosis viral oncogene homolog (avian) | 9.6 | 90.1 | 9.4 | 8.1 x 10^-5^ |
| 202647_s_at | NRAS | neuroblastoma RAS viral (v-ras) oncogene homolog | 5.1 | 32.2 | 6.3 | 5.1 x 10^-6^ |
| 206343_s_at | NRG1 | neuregulin 1 | 0.4 | 19.8 | 53.4 | 7.4 x 10^-7^ |
| 204053_x_at | PTEN | phosphatase and tensin homolog | 10.1 | 51.8 | 5.1 | 2.0 x 10^-4^ |
| 209896_s_at | PTPN11 | protein tyrosine phosphatase, non-receptor type 11 | 8.0 | 43.4 | 5.4 | 5.0 x 10^-8^ |
| 213603_s_at | RAC2 | ras-related C3 botulinum toxin substrate 2 (rho family, small GTP binding protein Rac2) | 6.9 | 50.2 | 7.3 | 1.8 x 10^-4^ |
| 212647_at | RRAS | related RAS viral (r-ras) oncogene homolog | 1.9 | 59.5 | 30.8 | 1.5 x 10^-7^ |
| 208456_s_at | RRAS2 | related RAS viral (r-ras) oncogene homolog 2 | 6.0 | 44.0 | 7.3 | 5.9 x 10^-6^ |
| 214853_s_at | SHC1 | SHC (Src homology 2 domain containing) transforming protein 1 | 14.9 | 204.0 | 13.7 | 3.3 x 10^-8^ |
| 205016_at | TGFA | transforming growth factor, alpha | 7.4 | 59.7 | 8.1 | 4.5 x 10^-11^ |
| **Integrin signaling** | | |  |  |  |  |
| 211160_x_at | ACTN1 | actinin, alpha 1 | 4.2 | 70.8 | 17.0 |  |
| 212607_at | AKT3 | v-akt murine thymoma viral oncogene homolog 3 (protein kinase B, gamma) | 1.5 | 9.3 | 6.2 | 5.8 x 10^-3^ |
| 207988_s_at | ARPC2 | actin related protein 2/3 complex, subunit 2, 34kDa | 27.7 | 187.1 | 6.8 | 6.8 x 10^-7^ |
| 226915_s_at | ARPC5L | actin related protein 2/3 complex, subunit 5-like | 9.0 | 52.2 | 5.8 | 3.3 x 10^-8^ |
| 224790_at | ASAP1 | ArfGAP with SH3 domain, ankyrin repeat and PH domain 1 | 1.0 | 9.5 | 9.8 | 4.8 x 10^-10^ |
| 212097_at | CAV1 | caveolin 1, caveolae protein, 22kDa | 1.0 | 177.4 | 169.5 | 2.5 x 10^-9^ |
| 208727_s_at | CDC42 | cell division cycle 42 (GTP binding protein, 25kDa) | 19.3 | 106.1 | 5.5 | 1.0 x 10^-3^ |
| 210105_s_at | FYN | FYN oncogene related to SRC, FGR, YES | 4.1 | 24.5 | 5.9 | 1.7 x 10^-3^ |
| 201474_s_at | ITGA3 | integrin, alpha 3 (antigen CD49C, alpha 3 subunit of VLA-3 receptor) | 3.2 | 50.1 | 15.8 | 7.6 x 10^-6^ |
| 201389_at | ITGA5 | integrin, alpha 5 (fibronectin receptor, alpha polypeptide) | 0.7 | 18.3 | 27.3 | 5.0 x 10^-9^ |
| 201656_at | ITGA6 | integrin, alpha 6 | 2.6 | 129.8 | 50.8 | 2.9 x 10^-6^ |
| 1553530_a_at | ITGB1 | integrin, beta 1 (fibronectin receptor, beta polypeptide, antigen CD29 includes MDF2, MSK12) | 16.9 | 216.8 | 12.8 | 5.9 x 10^-9^ |
| 204990_s_at | ITGB4 | integrin, beta 4 | 11.0 | 106.2 | 9.6 | 7.4 x 10^-7^ |
| 226535_at | ITGB6 | integrin, beta 6 | 5.4 | 44.6 | 8.3 | 3.0 x 10^-7^ |
| 202670_at | MAP2K1 | mitogen-activated protein kinase kinase 1 | 8.4 | 58.5 | 7.0 | 6.3 x 10^-3^ |
| 201319_at | MYL12A | myosin, light chain 12A, regulatory, non-sarcomeric | 19.7 | 114.7 | 5.8 | 1.5 x 10^-9^ |
| 202647_s_at | NRAS | neuroblastoma RAS viral (v-ras) oncogene homolog | 5.1 | 32.2 | 6.3 | 4.3 x 10^-6^ |
| 37966_at | PARVB | parvin, beta | 0.3 | 1.4 | 5.5 | 5.1 x 10^-6^ |
| 204053_x_at | PTEN | phosphatase and tensin homolog | 10.1 | 51.8 | 5.1 | 5.0 x 10^-6^ |
| 213603_s_at | RAC2 | ras-related C3 botulinum toxin substrate 2 (rho family, small GTP binding protein Rac2) | 6.9 | 50.2 | 7.3 | 2.0 x 10^-4^ |
| 214435_x_at | RALA | v-ral simian leukemia viral oncogene homolog A (ras related) | 5.5 | 47.9 | 8.6 | 1.8 x 10^-4^ |
| 221830_at | RAP2A | RAP2A, member of RAS oncogene family | 6.9 | 37.0 | 5.4 | 1.3 x 10^-6^ |
| 213923_at | RAP2B | RAP2B, member of RAS oncogene family | 6.5 | 55.8 | 8.5 | 6.3 x 10^-5^ |
| 200885_at | RHOC | ras homolog gene family, member C | 18.8 | 100.3 | 5.3 | 2.7 x 10^-7^ |
| 209885_at | RHOD | ras homolog gene family, member D | 2.5 | 32.6 | 13.0 | 5.5 x 10^-7^ |
| 222812_s_at | RHOF | ras homolog gene family, member F (in filopodia) | 0.3 | 3.1 | 11.5 | 9.2 x 10^-8^ |
| 212647_at | RRAS | related RAS viral (r-ras) oncogene homolog | 1.9 | 59.5 | 30.8 | 5.2 x 10^-8^ |
| 208456_s_at | RRAS2 | related RAS viral (r-ras) oncogene homolog 2 | 6.0 | 44.0 | 7.3 | 1.5 x 10^-7^ |
| 214853_s_at | SHC1 | SHC (Src homology 2 domain containing) transforming protein 1 | 14.9 | 204.0 | 13.7 | 5.9 x 10^-6^ |
| 209264_s_at | TSPAN4 | tetraspanin 4 | 1.0 | 9.4 | 9.5 | 3.3 x 10^-8^ |
| 225387_at | TSPAN5 | tetraspanin 5 | 0.9 | 10.1 | 10.9 | 1.3 x 10^-4^ |
| 200931_s_at | VCL | vinculin | 9.6 | 55.8 | 5.8 | 4.9 x 10^-4^ |
| 200808_s_at | ZYX | zyxin | 3.1 | 26.2 | 8.3 | 1.5 x 10^-6^ |
| **ILK signaling** | | |  |  |  |  |
| 211160_x_at | ACTN1 | actinin, alpha 1 | 4.2 | 70.8 | 17.0 |  |
| 212607_at | AKT3 | v-akt murine thymoma viral oncogene homolog 3 (protein kinase B, gamma) | 1.5 | 9.3 | 6.2 |  |
| 200779_at | ATF4 | activating transcription factor 4 (tax-responsive enhancer element B67) | 42.6 | 301.5 | 7.1 | 5.8 x 10^-3^ |
| 208727_s_at | CDC42 | cell division cycle 42 (GTP binding protein, 25kDa) | 19.3 | 106.1 | 5.5 | 6.8 x 10^-7^ |
| 229228_at | CREB5 | cAMP responsive element binding protein 5 | 0.4 | 3.4 | 7.9 | 1.3 x 10^-10^ |
| 200606_at | DSP | desmoplakin | 69.6 | 359.2 | 5.2 | 1.7 x 10^-3^ |
| 1554795_a_at | FBLIM1 | filamin binding LIM protein 1 | 0.6 | 9.2 | 14.9 | 1.1 x 10^-4^ |
| 209209_s_at | FERMT2 | fermitin family homolog 2 (Drosophila) | 2.1 | 13.6 | 6.5 | 9.1 x 10^-7^ |
| 213746_s_at | FLNA | filamin A, alpha (actin binding protein 280) | 2.0 | 38.0 | 19.1 | 1.2 x 10^-6^ |
| 208613_s_at | FLNB | filamin B, beta (actin binding protein 278) | 13.4 | 103.5 | 7.7 | 5.0 x 10^-5^ |
| 200989_at | HIF1A | hypoxia inducible factor 1, alpha subunit (basic helix-loop-helix transcription factor) | 30.0 | 258.6 | 8.6 | 2.1 x 10^-5^ |
| 204686_at | IRS1 | insulin receptor substrate 1 | 1.8 | 43.2 | 24.1 | 6.9 x 10^-6^ |
| 1553530_a_at | ITGB1 | integrin, beta 1 (fibronectin receptor, beta polypeptide, antigen CD29 includes MDF2, MSK12) | 16.9 | 216.8 | 12.8 | 8.1 x 10^-10^ |
| 204990_s_at | ITGB4 | integrin, beta 4 | 11.0 | 106.2 | 9.6 | 1.2 x 10^-5^ |
| 226535_at | ITGB6 | integrin, beta 6 | 5.4 | 44.6 | 8.3 | 7.4 x 10^-7^ |
| 202431_s_at | MYC | v-myc myelocytomatosis viral oncogene homolog (avian) | 9.6 | 90.1 | 9.4 | 3.0 x 10^-7^ |
| 37966_at | PARVB | parvin, beta | 0.3 | 1.4 | 5.5 | 6.3 x 10^-3^ |
| 209652_s_at | PGF | placental growth factor | 0.4 | 7.3 | 17.9 | 8.1 x 10^-5^ |
| 212680_x_at | PPP1R14B | protein phosphatase 1, regulatory (inhibitor) subunit 14B | 3.8 | 47.3 | 12.4 | 5.0 x 10^-6^ |
| 1554364_at | PPP2R5C | protein phosphatase 2, regulatory subunit B', gamma isoform | 0.2 | 1.1 | 5.6 | 7.2 x 10^-7^ |
| 204053_x_at | PTEN | phosphatase and tensin homolog | 10.1 | 51.8 | 5.1 | 1.1 x 10^-8^ |
| 204748_at | PTGS2 | prostaglandin-endoperoxide synthase 2 (prostaglandin G/H synthase and cyclooxygenase) | 2.8 | 44.1 | 15.9 | 2.4 x 10^-4^ |
| 213603_s_at | RAC2 | ras-related C3 botulinum toxin substrate 2 (rho family, small GTP binding protein Rac2) | 6.9 | 50.2 | 7.3 | 2.0 x 10^-4^ |
| 200885_at | RHOC | ras homolog gene family, member C | 18.8 | 100.3 | 5.3 | 2.9 x 10^-4^ |
| 209885_at | RHOD | ras homolog gene family, member D | 2.5 | 32.6 | 13.0 | 1.8 x 10^-4^ |
| 222812_s_at | RHOF | ras homolog gene family, member F (in filopodia) | 0.3 | 3.1 | 11.5 | 5.5 x 10^-7^ |
| 201980_s_at | RSU1 | Ras suppressor protein 1 | 9.8 | 50.6 | 5.1 | 9.2 x 10^-8^ |
| 213139_at | SNAI2 | snail homolog 2 (Drosophila) | 6.0 | 71.9 | 12.0 | 5.2 x 10^-8^ |
| 209651_at | TGFB1I1 | transforming growth factor beta 1 induced transcript 1 | 0.7 | 10.9 | 14.6 | 9.9 x 10^-8^ |
| 211527_x_at | VEGFA | vascular endothelial growth factor A | 4.2 | 35.1 | 8.3 | 2.0 x 10^-4^ |
| 209946_at | VEGFC | vascular endothelial growth factor C | 1.3 | 9.1 | 7.2 | 6.1 x 10^-5^ |
| **Ephrin receptor signaling** | | |  |  |  |  |
| 212607_at | AKT3 | v-akt murine thymoma viral oncogene homolog 3 (protein kinase B, gamma) | 1.5 | 9.3 | 6.2 | 2.2 x 10^-5^ |
| 207988_s_at | ARPC2 | actin related protein 2/3 complex, subunit 2, 34kDa | 27.7 | 187.1 | 6.8 |  |
| 226915_s_at | ARPC5L | actin related protein 2/3 complex, subunit 5-like | 9.0 | 52.2 | 5.8 |  |
| 200779_at | ATF4 | activating transcription factor 4 (tax-responsive enhancer element B67) | 42.6 | 301.5 | 7.1 | 6.8 x 10^-7^ |
| 208727_s_at | CDC42 | cell division cycle 42 (GTP binding protein, 25kDa) | 19.3 | 106.1 | 5.5 | 3.3 x 10^-8^ |
| 229228_at | CREB5 | cAMP responsive element binding protein 5 | 0.4 | 3.4 | 7.9 | 4.8 x 10^-10^ |
| 202224_at | CRK | v-crk sarcoma virus CT10 oncogene homolog (avian) | 6.5 | 34.6 | 5.3 | 1.3 x 10^-10^ |
| 209589_s_at | EPHB2 | EPH receptor B2 | 0.8 | 6.0 | 7.7 | 1.7 x 10^-3^ |
| 202894_at | EPHB4 | EPH receptor B4 | 3.2 | 19.1 | 6.0 | 1.1 x 10^-4^ |
| 210105_s_at | FYN | FYN oncogene related to SRC, FGR, YES | 4.1 | 24.5 | 5.9 | 1.6 x 10^-9^ |
| 205349_at | GNA15 | guanine nucleotide binding protein (G protein), alpha 15 (Gq class) | 5.6 | 28.9 | 5.2 | 2.5 x 10^-4^ |
| 201181_at | GNAI3 | guanine nucleotide binding protein (G protein), alpha inhibiting activity polypeptide 3 | 3.9 | 19.4 | 5.0 | 9.8 x 10^-8^ |
| 200744_s_at | GNB1 | guanine nucleotide binding protein (G protein), beta polypeptide 1 | 14.7 | 91.0 | 6.2 | 7.6 x 10^-6^ |
| 201921_at | GNG10 | guanine nucleotide binding protein (G protein), gamma 10 | 19.8 | 110.0 | 5.6 | 5.0 x 10^-7^ |
| 201474_s_at | ITGA3 | integrin, alpha 3 (antigen CD49C, alpha 3 subunit of VLA-3 receptor) | 3.2 | 50.1 | 15.8 | 7.4 x 10^-7^ |
| 201389_at | ITGA5 | integrin, alpha 5 (fibronectin receptor, alpha polypeptide) | 0.7 | 18.3 | 27.3 | 3.5 x 10^-7^ |
| 1553530_a_at | ITGB1 | integrin, beta 1 (fibronectin receptor, beta polypeptide, antigen CD29 includes MDF2, MSK12) | 16.9 | 216.8 | 12.8 | 1.0 x 10^-8^ |
| 202670_at | MAP2K1 | mitogen-activated protein kinase kinase 1 | 8.4 | 58.5 | 7.0 | 5.0 x 10^-9^ |
| 206571_s_at | MAP4K4 | mitogen-activated protein kinase kinase kinase kinase 4 | 3.4 | 26.5 | 7.8 | 2.9 x 10^-6^ |
| 202647_s_at | NRAS | neuroblastoma RAS viral (v-ras) oncogene homolog | 5.1 | 32.2 | 6.3 | 7.4 x 10^-7^ |
| 205463_s_at | PDGFA | platelet-derived growth factor alpha polypeptide | 3.0 | 19.4 | 6.4 | 1.5 x 10^-9^ |
| 218718_at | PDGFC | platelet derived growth factor C | 12.7 | 78.6 | 6.2 | 1.3 x 10^-6^ |
| 209652_s_at | PGF | placental growth factor | 0.4 | 7.3 | 17.9 | 5.1 x 10^-6^ |
| 209896_s_at | PTPN11 | protein tyrosine phosphatase, non-receptor type 11 | 8.0 | 43.4 | 5.4 | 5.8 x 10^-6^ |
| 213603_s_at | RAC2 | ras-related C3 botulinum toxin substrate 2 (rho family, small GTP binding protein Rac2) | 6.9 | 50.2 | 7.3 | 2.6 x 10^-7^ |
| 212647_at | RRAS | related RAS viral (r-ras) oncogene homolog | 1.9 | 59.5 | 30.8 | 7.2 x 10^-7^ |
| 208456_s_at | RRAS2 | related RAS viral (r-ras) oncogene homolog 2 | 6.0 | 44.0 | 7.3 | 5.0 x 10^-8^ |
| 214853_s_at | SHC1 | SHC (Src homology 2 domain containing) transforming protein 1 | 14.9 | 204.0 | 13.7 | 1.8 x 10^-4^ |
| 211527_x_at | VEGFA | vascular endothelial growth factor A | 4.2 | 35.1 | 8.3 | 1.5 x 10^-7^ |
| 209946_at | VEGFC | vascular endothelial growth factor C | 1.3 | 9.1 | 7.2 | 5.9 x 10^-6^ |
| **Viral entry visa endocytotic pathways** | | |  |  |  |  |
| 200615_s_at | AP2B1 | adaptor-related protein complex 2, beta 1 subunit | 7.2 | 37.7 | 5.2 | 1.6 x 10^-5^ |
| 208074_s_at | AP2S1 | adaptor-related protein complex 2, sigma 1 subunit | 11.6 | 79.5 | 6.8 | 2.2 x 10^-5^ |
| 212097_at | CAV1 | caveolin 1, caveolae protein, 22kDa | 1.0 | 177.4 | 169.5 |  |
| 208727_s_at | CDC42 | cell division cycle 42 (GTP binding protein, 25kDa) | 19.3 | 106.1 | 5.5 |  |
| 211043_s_at | CLTB | clathrin, light chain (Lcb) | 7.0 | 61.5 | 8.8 | 9.5 x 10^-8^ |
| 1555895_at | DNM2 | dynamin 2 | 0.5 | 2.5 | 5.2 | 9.3 x 10^-8^ |
| 213746_s_at | FLNA | filamin A, alpha (actin binding protein 280) | 2.0 | 38.0 | 19.1 | 1.0 x 10^-3^ |
| 208613_s_at | FLNB | filamin B, beta (actin binding protein 278) | 13.4 | 103.5 | 7.7 | 1.7 x 10^-3^ |
| 210105_s_at | FYN | FYN oncogene related to SRC, FGR, YES | 4.1 | 24.5 | 5.9 | 9.0 x 10^-5^ |
| 201474_s_at | ITGA3 | integrin, alpha 3 (antigen CD49C, alpha 3 subunit of VLA-3 receptor) | 3.2 | 50.1 | 15.8 | 7.0 x 10^-5^ |
| 201389_at | ITGA5 | integrin, alpha 5 (fibronectin receptor, alpha polypeptide) | 0.7 | 18.3 | 27.3 | 2.1 x 10^-5^ |
| 201656_at | ITGA6 | integrin, alpha 6 | 2.6 | 129.8 | 50.8 | 6.9 x 10^-6^ |
| 1553530_a_at | ITGB1 | integrin, beta 1 (fibronectin receptor, beta polypeptide, antigen CD29 includes MDF2, MSK12) | 16.9 | 216.8 | 12.8 | 7.6 x 10^-6^ |
| 204990_s_at | ITGB4 | integrin, beta 4 | 11.0 | 106.2 | 9.6 | 5.0 x 10^-9^ |
| 226535_at | ITGB6 | integrin, beta 6 | 5.4 | 44.6 | 8.3 | 2.9 x 10^-6^ |
| 202647_s_at | NRAS | neuroblastoma RAS viral (v-ras) oncogene homolog | 5.1 | 32.2 | 6.3 | 5.9 x 10^-9^ |
| 213603_s_at | RAC2 | ras-related C3 botulinum toxin substrate 2 (rho family, small GTP binding protein Rac2) | 6.9 | 50.2 | 7.3 | 7.4 x 10^-7^ |
| 212647_at | RRAS | related RAS viral (r-ras) oncogene homolog | 1.9 | 59.5 | 30.8 | 3.0 x 10^-7^ |
| 208456_s_at | RRAS2 | related RAS viral (r-ras) oncogene homolog 2 | 6.0 | 44.0 | 7.3 | 6.3 x 10^-3^ |
| **Glioblastome multiforme signaling** | | |  |  |  |  |
| 212607_at | AKT3 | v-akt murine thymoma viral oncogene homolog 3 (protein kinase B, gamma) | 1.5 | 9.3 | 6.2 | 1.8 x 10^-4^ |
| 213523_at | CCNE1 | cyclin E1 | 0.7 | 3.7 | 5.1 | 1.5 x 10^-7^ |
| 208727_s_at | CDC42 | cell division cycle 42 (GTP binding protein, 25kDa) | 19.3 | 106.1 | 5.5 | 5.9 x 10^-6^ |
| 224851_at | CDK6 | cyclin-dependent kinase 6 | 7.8 | 53.2 | 6.8 |  |
| 202284_s_at | CDKN1A | cyclin-dependent kinase inhibitor 1A (p21, Cip1) | 10.7 | 167.8 | 15.7 |  |
| 203693_s_at | E2F3 | E2F transcription factor 3 | 1.0 | 8.5 | 8.1 | 6.8 x 10^-7^ |
| 201984_s_at | EGFR | epidermal growth factor receptor (erythroblastic leukemia viral (v-erb-b) oncogene homolog, avian) | 9.2 | 93.6 | 10.2 | 1.9 x 10^-8^ |
| 201189_s_at | ITPR3 | inositol 1,4,5-triphosphate receptor, type 3 | 12.4 | 63.9 | 5.1 | 1.7 x 10^-3^ |
| 202670_at | MAP2K1 | mitogen-activated protein kinase kinase 1 | 8.4 | 58.5 | 7.0 | 1.1 x 10^-6^ |
| 202431_s_at | MYC | v-myc myelocytomatosis viral oncogene homolog (avian) | 9.6 | 90.1 | 9.4 | 2.0 x 10^-6^ |
| 217150_s_at | NF2 | neurofibromin 2 (merlin) | 0.8 | 4.2 | 5.3 | 4.6 x 10^-10^ |
| 202647_s_at | NRAS | neuroblastoma RAS viral (v-ras) oncogene homolog | 5.1 | 32.2 | 6.3 | 9.1 x 10^-7^ |
| 205463_s_at | PDGFA | platelet-derived growth factor alpha polypeptide | 3.0 | 19.4 | 6.4 | 1.7 x 10^-8^ |
| 218718_at | PDGFC | platelet derived growth factor C | 12.7 | 78.6 | 6.2 | 1.5 x 10^-9^ |
| 234971_x_at | PLCD3 | phospholipase C, delta 3 | 0.6 | 5.4 | 9.3 | 8.1 x 10^-5^ |
| 206080_at | PLCH2 | phospholipase C, eta 2 | 0.5 | 4.1 | 8.4 | 7.4 x 10^-5^ |
| 204053_x_at | PTEN | phosphatase and tensin homolog | 10.1 | 51.8 | 5.1 | 5.1 x 10^-6^ |
| 213603_s_at | RAC2 | ras-related C3 botulinum toxin substrate 2 (rho family, small GTP binding protein Rac2) | 6.9 | 50.2 | 7.3 | 5.8 x 10^-6^ |
| 200885_at | RHOC | ras homolog gene family, member C | 18.8 | 100.3 | 5.3 | 2.6 x 10^-7^ |
| 209885_at | RHOD | ras homolog gene family, member D | 2.5 | 32.6 | 13.0 | 2.2 x 10^-8^ |
| 222812_s_at | RHOF | ras homolog gene family, member F (in filopodia) | 0.3 | 3.1 | 11.5 | 5.2 x 10^-6^ |
| 212647_at | RRAS | related RAS viral (r-ras) oncogene homolog | 1.9 | 59.5 | 30.8 | 2.0 x 10^-4^ |
| 208456_s_at | RRAS2 | related RAS viral (r-ras) oncogene homolog 2 | 6.0 | 44.0 | 7.3 | 1.8 x 10^-4^ |
| 214853_s_at | SHC1 | SHC (Src homology 2 domain containing) transforming protein 1 | 14.9 | 204.0 | 13.7 | 5.5 x 10^-7^ |
| 209152_s_at | TCF3 | transcription factor 3 (E2A immunoglobulin enhancer binding factors E12/E47) | 1.0 | 6.7 | 6.6 | 9.2 x 10^-8^ |
| 210248_at | WNT7A | wingless-type MMTV integration site family, member 7A | 0.2 | 2.9 | 15.7 | 5.2 x 10^-8^ |
| **HER2 signaling** | | |  |  |  |  |
| 212607_at | AKT3 | v-akt murine thymoma viral oncogene homolog 3 (protein kinase B, gamma) | 1.5 | 9.3 | 6.2 | 5.9 x 10^-6^ |
| 205239_at | AREG | amphiregulin | 2.7 | 354.7 | 133.9 | 3.3 x 10^-8^ |
| 213523_at | CCNE1 | cyclin E1 | 0.7 | 3.7 | 5.1 | 2.6 x 10^-7^ |
| 208727_s_at | CDC42 | cell division cycle 42 (GTP binding protein, 25kDa) | 19.3 | 106.1 | 5.5 | 1.0 x 10^-5^ |
| 224851_at | CDK6 | cyclin-dependent kinase 6 | 7.8 | 53.2 | 6.8 |  |
| 202284_s_at | CDKN1A | cyclin-dependent kinase inhibitor 1A (p21, Cip1) | 10.7 | 167.8 | 15.7 |  |
| 201984_s_at | EGFR | epidermal growth factor receptor (erythroblastic leukemia viral (v-erb-b) oncogene homolog, avian) | 9.2 | 93.6 | 10.2 | 6.8 x 10^-7^ |
| 1553530_a_at | ITGB1 | integrin, beta 1 (fibronectin receptor, beta polypeptide, antigen CD29 includes MDF2, MSK12) | 16.9 | 216.8 | 12.8 | 8.5 x 10^-10^ |
| 204990_s_at | ITGB4 | integrin, beta 4 | 11.0 | 106.2 | 9.6 | 1.9 x 10^-8^ |
| 226535_at | ITGB6 | integrin, beta 6 | 5.4 | 44.6 | 8.3 | 1.7 x 10^-3^ |
| 201069_at | MMP2 | matrix metallopeptidase 2 (gelatinase A, 72kDa gelatinase, 72kDa type IV collagenase) | 0.8 | 6.9 | 9.0 | 1.1 x 10^-6^ |
| 202647_s_at | NRAS | neuroblastoma RAS viral (v-ras) oncogene homolog | 5.1 | 32.2 | 6.3 | 2.0 x 10^-6^ |
| 206343_s_at | NRG1 | neuregulin 1 | 0.4 | 19.8 | 53.4 | 9.1 x 10^-7^ |
| 232132_at | PARD6G | par-6 partitioning defective 6 homolog gamma (C. elegans) | 0.2 | 1.5 | 9.5 | 7.4 x 10^-7^ |
| 213603_s_at | RAC2 | ras-related C3 botulinum toxin substrate 2 (rho family, small GTP binding protein Rac2) | 6.9 | 50.2 | 7.3 | 3.0 x 10^-7^ |
| 212647_at | RRAS | related RAS viral (r-ras) oncogene homolog | 1.9 | 59.5 | 30.8 | 6.3 x 10^-3^ |
| 208456_s_at | RRAS2 | related RAS viral (r-ras) oncogene homolog 2 | 6.0 | 44.0 | 7.3 | 2.5 x 10^-3^ |
| **Renal cell carcinoma signaling** | | |  |  |  |  |
| 212607_at | AKT3 | v-akt murine thymoma viral oncogene homolog 3 (protein kinase B, gamma) | 1.5 | 9.3 | 6.2 | 7.4 x 10^-7^ |
| 208727_s_at | CDC42 | cell division cycle 42 (GTP binding protein, 25kDa) | 19.3 | 106.1 | 5.5 | 8.6 x 10^-6^ |
| 202224_at | CRK | v-crk sarcoma virus CT10 oncogene homolog (avian) | 6.5 | 34.6 | 5.3 | 1.8 x 10^-4^ |
| 224833_at | ETS1 | v-ets erythroblastosis virus E26 oncogene homolog 1 (avian) | 6.4 | 94.6 | 14.8 | 1.5 x 10^-7^ |
| 200989_at | HIF1A | hypoxia inducible factor 1, alpha subunit (basic helix-loop-helix transcription factor) | 30.0 | 258.6 | 8.6 | 5.9 x 10^-6^ |
| 202670_at | MAP2K1 | mitogen-activated protein kinase kinase 1 | 8.4 | 58.5 | 7.0 |  |
| 203510_at | MET | met proto-oncogene (hepatocyte growth factor receptor) | 30.9 | 195.3 | 6.3 |  |
| 202647_s_at | NRAS | neuroblastoma RAS viral (v-ras) oncogene homolog | 5.1 | 32.2 | 6.3 | 6.8 x 10^-7^ |
| 209896_s_at | PTPN11 | protein tyrosine phosphatase, non-receptor type 11 | 8.0 | 43.4 | 5.4 | 1.7 x 10^-3^ |
| 213603_s_at | RAC2 | ras-related C3 botulinum toxin substrate 2 (rho family, small GTP binding protein Rac2) | 6.9 | 50.2 | 7.3 | 1.6 x 10^-9^ |
| 212647_at | RRAS | related RAS viral (r-ras) oncogene homolog | 1.9 | 59.5 | 30.8 | 3.1 x 10^-6^ |
| 208456_s_at | RRAS2 | related RAS viral (r-ras) oncogene homolog 2 | 6.0 | 44.0 | 7.3 | 8.1 x 10^-10^ |
| 201249_at | SLC2A1 | solute carrier family 2 (facilitated glucose transporter), member 1 | 0.3 | 5.5 | 17.7 | 1.5 x 10^-9^ |
| 205016_at | TGFA | transforming growth factor, alpha | 7.4 | 59.7 | 8.1 | 3.8 x 10^-7^ |
| 203085_s_at | TGFB1 | transforming growth factor, beta 1 | 2.6 | 14.9 | 5.7 | 5.1 x 10^-6^ |
| 211527_x_at | VEGFA | vascular endothelial growth factor A | 4.2 | 35.1 | 8.3 | 5.0 x 10^-8^ |
| **Clatharin-mediated endocytosis-mediated signaling** | | |  |  |  |  |
| 225522_at | AAK1 | AP2 associated kinase 1 | 5.8 | 36.4 | 6.3 | 1.5 x 10^-7^ |
| 200615_s_at | AP2B1 | adaptor-related protein complex 2, beta 1 subunit | 7.2 | 37.7 | 5.2 | 5.9 x 10^-6^ |
| 208074_s_at | AP2S1 | adaptor-related protein complex 2, sigma 1 subunit | 11.6 | 79.5 | 6.8 | 3.9 x 10^-7^ |
| 207988_s_at | ARPC2 | actin related protein 2/3 complex, subunit 2, 34kDa | 27.7 | 187.1 | 6.8 | 4.5 x 10^-11^ |
| 226915_s_at | ARPC5L | actin related protein 2/3 complex, subunit 5-like | 9.0 | 52.2 | 5.8 | 1.2 x 10^-4^ |
| 243475_at | CBL | Cas-Br-M (murine) ecotropic retroviral transforming sequence | 0.4 | 2.3 | 6.0 | 1.6 x 10^-5^ |
| 208727_s_at | CDC42 | cell division cycle 42 (GTP binding protein, 25kDa) | 19.3 | 106.1 | 5.5 |  |
| 211043_s_at | CLTB | clathrin, light chain (Lcb) | 7.0 | 61.5 | 8.8 |  |
| 212072_s_at | CSNK2A1 | casein kinase 2, alpha 1 polypeptide | 12.7 | 93.5 | 7.3 | 1.6 x 10^-5^ |
| 214073_at | CTTN | cortactin | 1.4 | 13.0 | 9.3 | 9.5 x 10^-8^ |
| 1555895_at | DNM2 | dynamin 2 | 0.5 | 2.5 | 5.2 | 9.3 x 10^-8^ |
| 203105_s_at | DNM1L | dynamin 1-like | 6.1 | 32.8 | 5.3 | 3.3 x 10^-8^ |
| 209589_s_at | EPHB2 | EPH receptor B2 | 0.8 | 6.0 | 7.7 | 4.8 x 10^-10^ |
| 204422_s_at | FGF2 | fibroblast growth factor 2 (basic) | 0.2 | 1.8 | 7.7 | 1.7 x 10^-7^ |
| 227271_at | FGF11 | fibroblast growth factor 11 | 1.3 | 8.3 | 6.2 | 1.7 x 10^-3^ |
| 201389_at | ITGA5 | integrin, alpha 5 (fibronectin receptor, alpha polypeptide) | 0.7 | 18.3 | 27.3 | 9.0 x 10^-5^ |
| 1553530_a_at | ITGB1 | integrin, beta 1 (fibronectin receptor, beta polypeptide, antigen CD29 includes MDF2, MSK12) | 16.9 | 216.8 | 12.8 | 5.9 x 10^-7^ |
| 204990_s_at | ITGB4 | integrin, beta 4 | 11.0 | 106.2 | 9.6 | 1.2 x 10^-6^ |
| 226535_at | ITGB6 | integrin, beta 6 | 5.4 | 44.6 | 8.3 | 7.0 x 10^-5^ |
| 202068_s_at | LDLR | low density lipoprotein receptor | 21.1 | 138.2 | 6.6 | 6.0 x 10^-10^ |
| 203510_at | MET | met proto-oncogene (hepatocyte growth factor receptor) | 30.9 | 195.3 | 6.3 | 2.5 x 10^-4^ |
| 205463_s_at | PDGFA | platelet-derived growth factor alpha polypeptide | 3.0 | 19.4 | 6.4 | 2.7 x 10^-3^ |
| 218718_at | PDGFC | platelet derived growth factor C | 12.7 | 78.6 | 6.2 | 2.5 x 10^-6^ |
| 209652_s_at | PGF | placental growth factor | 0.4 | 7.3 | 17.9 | 2.9 x 10^-6^ |
| 211527_x_at | VEGFA | vascular endothelial growth factor A | 4.2 | 35.1 | 8.3 | 7.4 x 10^-7^ |
| 209946_at | VEGFC | vascular endothelial growth factor C | 1.3 | 9.1 | 7.2 | 3.0 x 10^-7^ |
| **Agrin interactions at neuromuscular junction** | | |  |  |  |  |
| 208727_s_at | CDC42 | cell division cycle 42 (GTP binding protein, 25kDa) | 19.3 | 106.1 | 5.5 | 2.1 x 10^-8^ |
| 214073_at | CTTN | cortactin | 1.4 | 13.0 | 9.3 | 3.8 x 10^-7^ |
| 201984_s_at | EGFR | epidermal growth factor receptor (erythroblastic leukemia viral (v-erb-b) oncogene homolog, avian) | 9.2 | 93.6 | 10.2 | 5.8 x 10^-6^ |
| 206173_x_at | GABPB1 | GA binding protein transcription factor, beta subunit 1 | 1.2 | 7.3 | 6.1 | 2.6 x 10^-7^ |
| 201474_s_at | ITGA3 | integrin, alpha 3 (antigen CD49C, alpha 3 subunit of VLA-3 receptor) | 3.2 | 50.1 | 15.8 | 7.2 x 10^-7^ |
| 201389_at | ITGA5 | integrin, alpha 5 (fibronectin receptor, alpha polypeptide) | 0.7 | 18.3 | 27.3 | 1.6 x 10^-5^ |
| 201656_at | ITGA6 | integrin, alpha 6 | 2.6 | 129.8 | 50.8 | 2.2 x 10^-5^ |
| 1553530_a_at | ITGB1 | integrin, beta 1 (fibronectin receptor, beta polypeptide, antigen CD29 includes MDF2, MSK12) | 16.9 | 216.8 | 12.8 |  |
| 201505_at | LAMB1 | laminin, beta 1 | 1.8 | 31.3 | 17.3 |  |
| 200770_s_at | LAMC1 | laminin, gamma 1 (formerly LAMB2) | 3.5 | 45.0 | 12.9 | 1.7 x 10^-3^ |
| 202647_s_at | NRAS | neuroblastoma RAS viral (v-ras) oncogene homolog | 5.1 | 32.2 | 6.3 | 1.2 x 10^-6^ |
| 206343_s_at | NRG1 | neuregulin 1 | 0.4 | 19.8 | 53.4 | 9.1 x 10^-7^ |
| 213603_s_at | RAC2 | ras-related C3 botulinum toxin substrate 2 (rho family, small GTP binding protein Rac2) | 6.9 | 50.2 | 7.3 | 1.5 x 10^-8^ |
| 212647_at | RRAS | related RAS viral (r-ras) oncogene homolog | 1.9 | 59.5 | 30.8 | 5.0 x 10^-9^ |
| 208456_s_at | RRAS2 | related RAS viral (r-ras) oncogene homolog 2 | 6.0 | 44.0 | 7.3 | 2.9 x 10^-6^ |

^1^ For the top canonical pathways identified by Ingenuity Pathway Analysis (Table 4) the genes overlapping with the basal cell signature were identified, and the basal and differentiated epithelium expression ratios were extracted.

^2^ p value following Benjamini-Hochberg correction.
